# Protein tyrosine phosphatase inactivation by electrophilic tyrosine modification

**DOI:** 10.1039/d5sc07398g

**Published:** 2026-01-19

**Authors:** Madeleine L. Ware, David M. Leace, Zihan Qu, Quentin Schaefer, Sagar D. Vaidya, Mikayla L. Horvath, Zhihong Li, Yunpeng Bai, Zhong-Yin Zhang, Ku-Lung Hsu

**Affiliations:** a Department of Chemistry, University of Texas at Austin Austin TX 78712 USA ken.hsu@austin.utexas.edu; b Department of Pharmacology, University of Virginia School of Medicine Charlottesville VA 22908 USA; c James Tarpo Jr. and Margaret Tarpo Department of Chemistry USA zhang-zy@purdue.edu; d Borch Department of Medicinal Chemistry and Molecular Pharmacology, Purdue University West Lafayette IN 47907 USA

## Abstract

Covalent protein tyrosine phosphatase (PTP) inhibitors principally target the catalytic cysteine, which is highly conserved and presents challenges for achieving selectivity across the PTP family. Here, we identified a tyrosine-reactive covalent inhibitor for SHP2 (DML189) with secondary molecular glue activity *via* a ligand induced protein tethering (LIPT) mechanism. We detected ligand binding at Y279, which is in proximity to the catalytic cysteine on SHP2 and has known functional and pathogenic properties. Covalent SHP2 modification by DML189 induced reversible disulfide tethering and monomer loss that was not observed to the same extent on PTP1B, LYP, or SHP1. Crosslinking mass spectrometry detected unique tethering events involving regulatory cysteines after DML189 modification on SHP2. Together, we discovered a tyrosine reactive inhibitor that targets functional sites on SHP2 and exhibits molecular glue activity through LIPT.

## Abstract 2

Here, we develop covalent inhibitors that inactivate protein tyrosine phosphatases (PTPs) *via* electrophilic modification of functional tyrosines.

## Graphical abstract

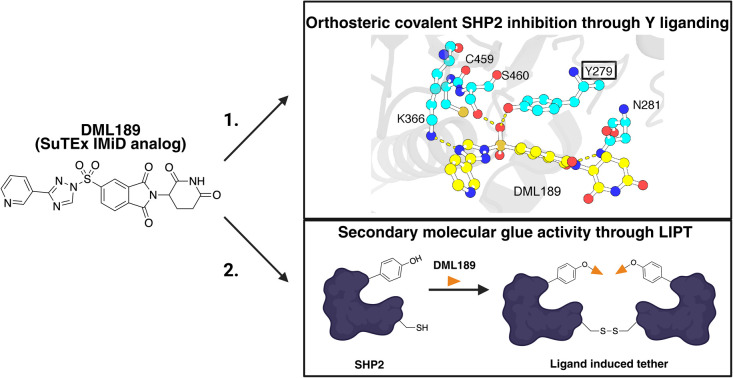

## Introduction

**Fig. 1:**
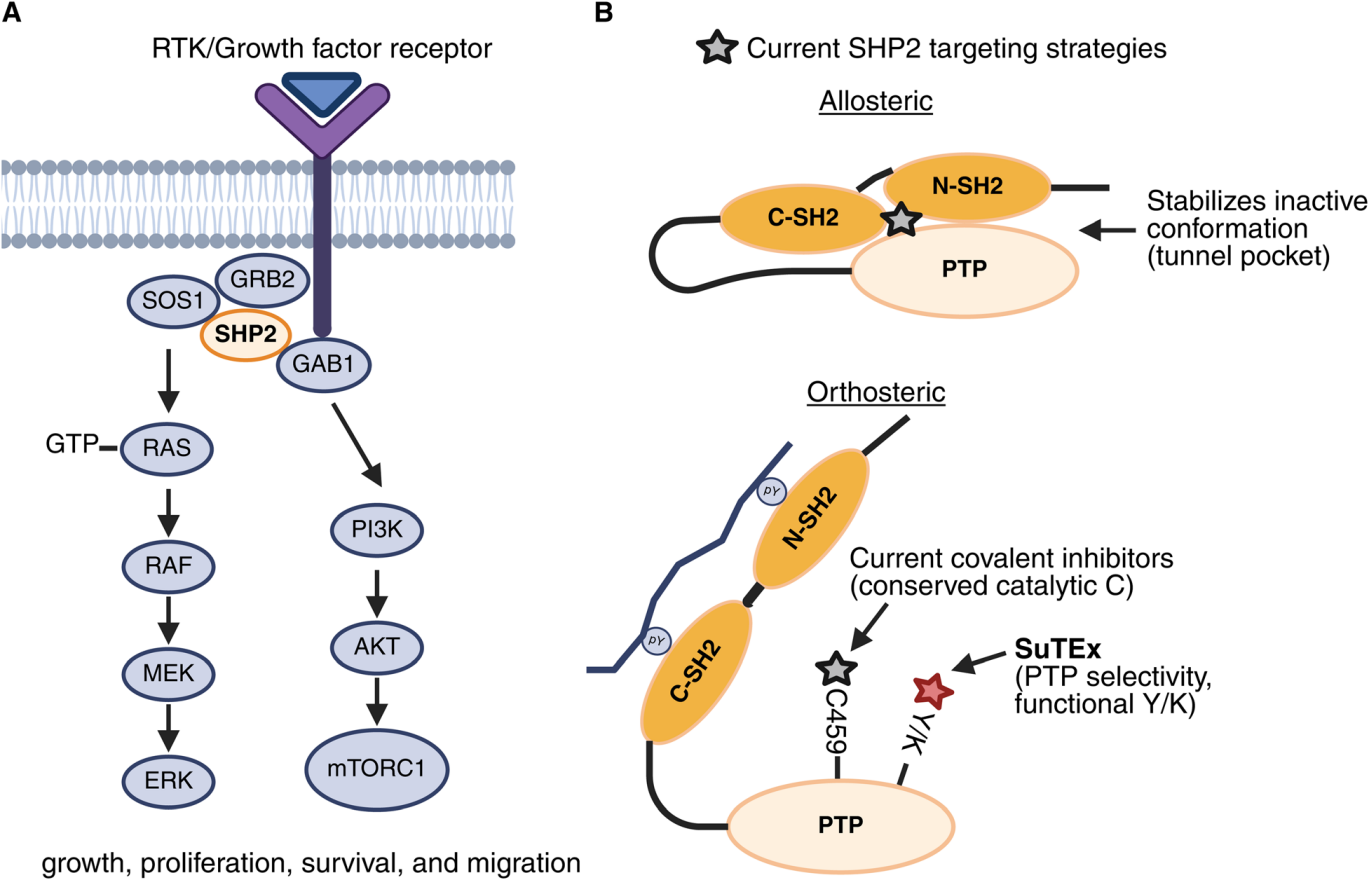
SHP2 is a key regulator of cellular signaling and an emerging drug target. (A) SHP2 serves as a phosphatase and scaffolding protein for propagating RAS-MAPK and PI3K-AKT signaling. (B) Current enzymatic inhibitors of SHP2 include: (i) allosteric inhibitors such as SHP099 that bind SHP2's “tunnel” pocket to lock its autoinhibited conformation and (ii) covalent orthosteric inhibitors targeting the conserved catalytic cysteine (C459) in SHP2's PTP domain. This work discloses covalent SuTEx molecules that expand the targetable residues in the PTP domain to include lysine and tyrosine.

Protein tyrosine phosphatase non-receptor type 11 (PTPN11), which encodes SHP2, is a critical mediator of receptor tyrosine kinase (RTK) signaling. SHP2 contains a catalytic PTP domain and tandem SH2 domains that transition between an autoinhibited ‘closed’ conformation and an ‘open’ active state upon binding to phosphorylated tyrosine motifs.^1–3^ Through this mechanism, SHP2 acts as a positive regulator of RAS-ERK signaling by relieving RasGAP-mediated inhibition and by functioning as a scaffold for pathway activation (Fig. 1A).^4,5^ Oncogenic SHP2 mutations are recurrent in juvenile myelomonocytic leukemia^6^ and its activity is also required for tumor growth and therapy resistance in diverse solid cancers.^7,8^ In addition, SHP2 is recruited by the immune checkpoint receptor PD-1 to dephosphorylate CD28, thereby dampening T-cell co-stimulation.^9,10^ These biological roles make SHP2 a compelling therapeutic target in both oncology and immunotherapy.

Despite this validation, efforts to pharmacologically modulate SHP2 have faced longstanding challenges.^11,12^ The highly conserved and polar PTP active site has hindered development of selective orthosteric inhibitors. Allosteric inhibitors such as SHP099 overcame these barriers by binding a tunnel-like pocket formed at the interface of the N-SH2, C-SH2, and PTP domains to stabilize the autoinhibited ‘closed’ conformation (Fig. 1B).^13–15^ This chemical class has generated multiple clinical candidates, including TNO155 (batoprotafib), RMC-4630 (vociprotafib), JAB-3312 (sitneprotafib), and BBP-398, most of which are being evaluated in combination with inhibitors of RTKs or KRAS mutants.^16^ However, their efficacy as monotherapies has been limited, and resistance mutations that bias SHP2 toward the open conformation reduce allosteric inhibitor binding.^17^ Moreover, SHP2 allosteric inhibitors also display off-target autophagy inhibition^18^ adding additional complexity when deploying them as chemical probes and potential therapeutics.

Emerging strategies aim to address limitations of allosteric inhibition. Orthosteric active-site binders, including covalent inhibitors targeting the catalytic cysteine, are being developed to directly block catalysis even in constitutively active mutants (Fig. 1B).^19^ In parallel, proteolysis-targeting chimeras (PROTACs) capable of degrading SHP2 have demonstrated preclinical activity and may abrogate both catalytic and scaffolding functions.^20–23^ Together, these approaches expand the toolkit for interrogating SHP2 biology and tackling mechanisms of resistance. Despite these advances, alternative chemical strategies are needed for modulating SHP2 function with high potency and selectivity while overcoming the liabilities of existing allosteric scaffolds.

Here, we screened a library of tyrosine-reactive sulfonyl-triazole (SuTEx) ligands to discover a thalidomide analog, DML189 that blocks biochemical activity of SHP2 with selectivity against additional PTPs (SHP1, LYP, PTP1B) tested. DML189 covalently engages SHP2 Y279 and induces reversible disulfide tethering to form a dimer *via* a ligand induced protein tethering (LIPT) mechanism. Crosslinking mass spectrometry revealed LIPT between the catalytic cysteine (C459) and a backdoor cysteine (C367). Our findings expand the repertoire of orthosteric SHP2 inhibitors to include tyrosine-reactive SuTEx ligands with secondary molecular glue activity.

## Results

### Discovery of sulfonyl-triazole PTP inhibitors

PTPs utilize a highly nucleophilic and conserved catalytic cysteine for biochemical function.^2^ Consequently, electrophilic compounds are prone to target this nucleophilic site, which can be challenging for achieving PTP selectivity. Engaging non-cysteine residues can expose less-conserved regions as a more suitable starting point for developing PTP-selective inhibitors. Sulfonyl-azole chemistry has recently emerged as an electrophilic class for expanding covalent targeting beyond cysteine to include functional tyrosine, lysine and histidine sites on proteins.^24^

**Fig. 2:**
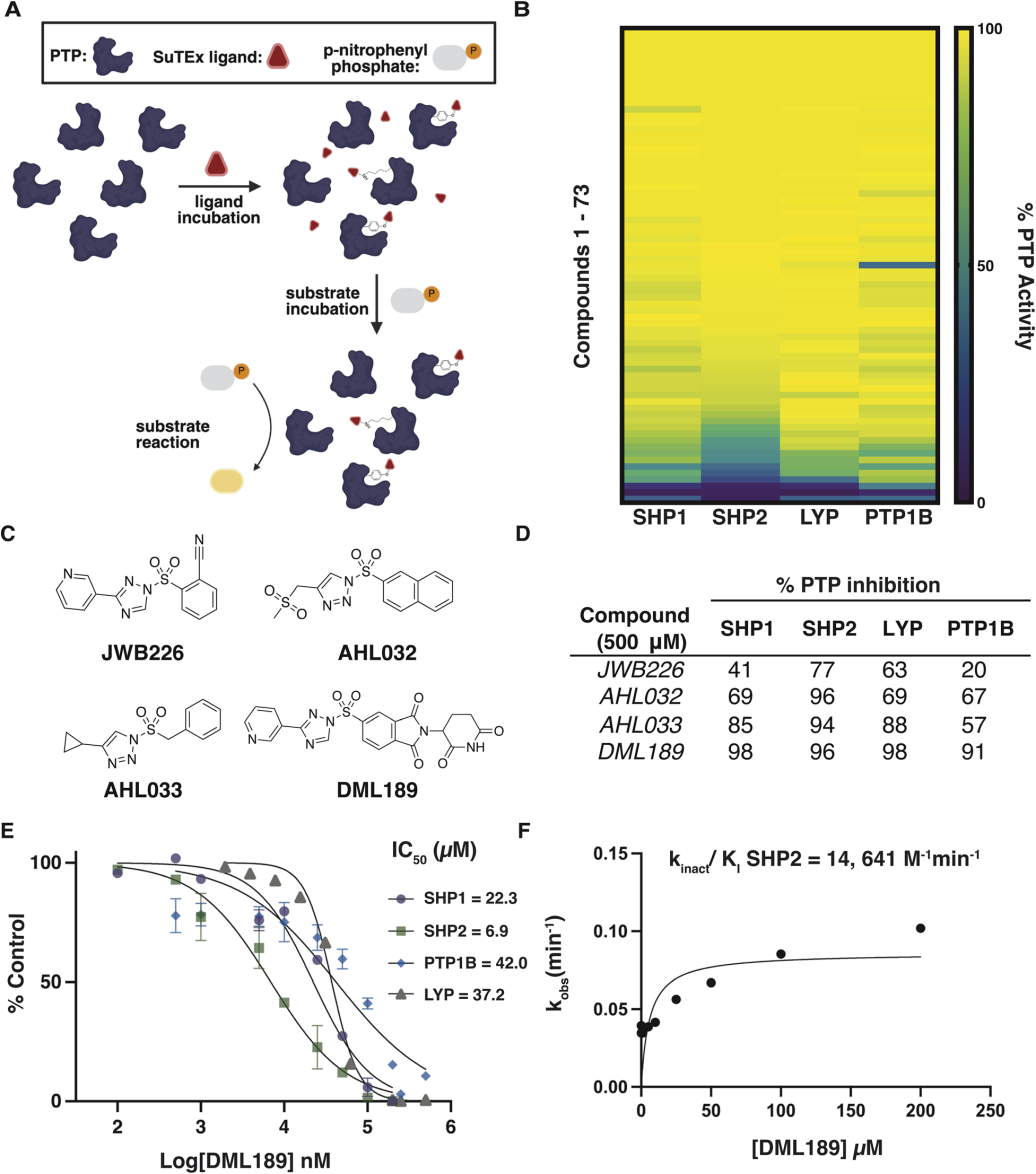
Discovery of sulfonyl-triazole PTP inhibitors. (A) High throughput screening workflow to evaluate PTP activity following treatment with SuTEx fragments. The figure was generated using BioRender. (B) Heat map depicting inhibitory activity (detected as reduction in % PTP activity) of SuTEx compounds (500 µM, 30 min) tested against respective PTPs. (C) Chemical structures for SuTEx compounds (DML189, AHL032, AHL033, JWB226) showing >70% inhibition against at least 1 PTP. (D) % Inhibition of SHP1, SHP2, PTP1B, and LYP following treatment with DML189, AHL032, AHL033, JWB226 (500 µM, 30 min). (E) Concentration-dependent inactivation of SHP1, SHP2, PTP1B, and LYP with DML189 treatment (1 h). Data are shown as mean ± SD of % control absorbance values. (F) DML189-SHP2 *k*_inact_/*K*_I_ was derived from plotting *k*_obs_ at various concentrations of DML189 and fitting the line to *k*_obs_ = *k*_inact_ × [I]/(*K*_I_ + [I]).

To test whether these electrophiles could be applied to PTP inhibitor development, we deployed an activity-based assay to screen a diverse sulfonyl triazole (SuTEx) electrophile library against the catalytic domains of SHP1, SHP2, LYP, and PTP1B (73 compounds, 500 µM, 30 min; Fig. 2A, B and Table S1) using *p*-nitrophenyl phosphate (pNPP) as a substrate. We identified 4 ligands that showed >70% inhibitory activity against one or more PTPs (Fig. 2C and D). From these hits, we selected DML189 for further studies because of its prominent activity against PTPs (>90% inhibition) and distinct adduct group containing thalidomide, a drug with established molecular glue activity^25,26^ (Fig. 2C and D).

We performed dose response studies, which provided initial evidence that DML189 exhibited highest potency for SHP2 (IC_50_ = 6.9 µM) and substantially reduced activity (3–6-fold) against SHP1, PTP1B, and LYP (Fig. 2E). A covalent binding mechanism for DML189 was supported by time-dependent SHP2 inactivation and a lack of inhibitory activity with thalidomide alone (0–500 µM, 1 h; Fig. S1A and B). We measured a *k*_inact_/*K*_I_ of 14 641 M^−1^ min^−1^ for DML189 against SHP2 (Fig. 2F and S1C). Given that covalent lenalidomide containing molecules have been shown to induce CRBN mediated neosubstrate degradation,^27^ we tested for but did not observe degradative activity of DML189 against SHP2, PTP1B, LYP or SHP1 in live cells (Fig. S1D and E).

### DML189 binds functional tyrosines in the SHP2 active site

**Fig. 3:**
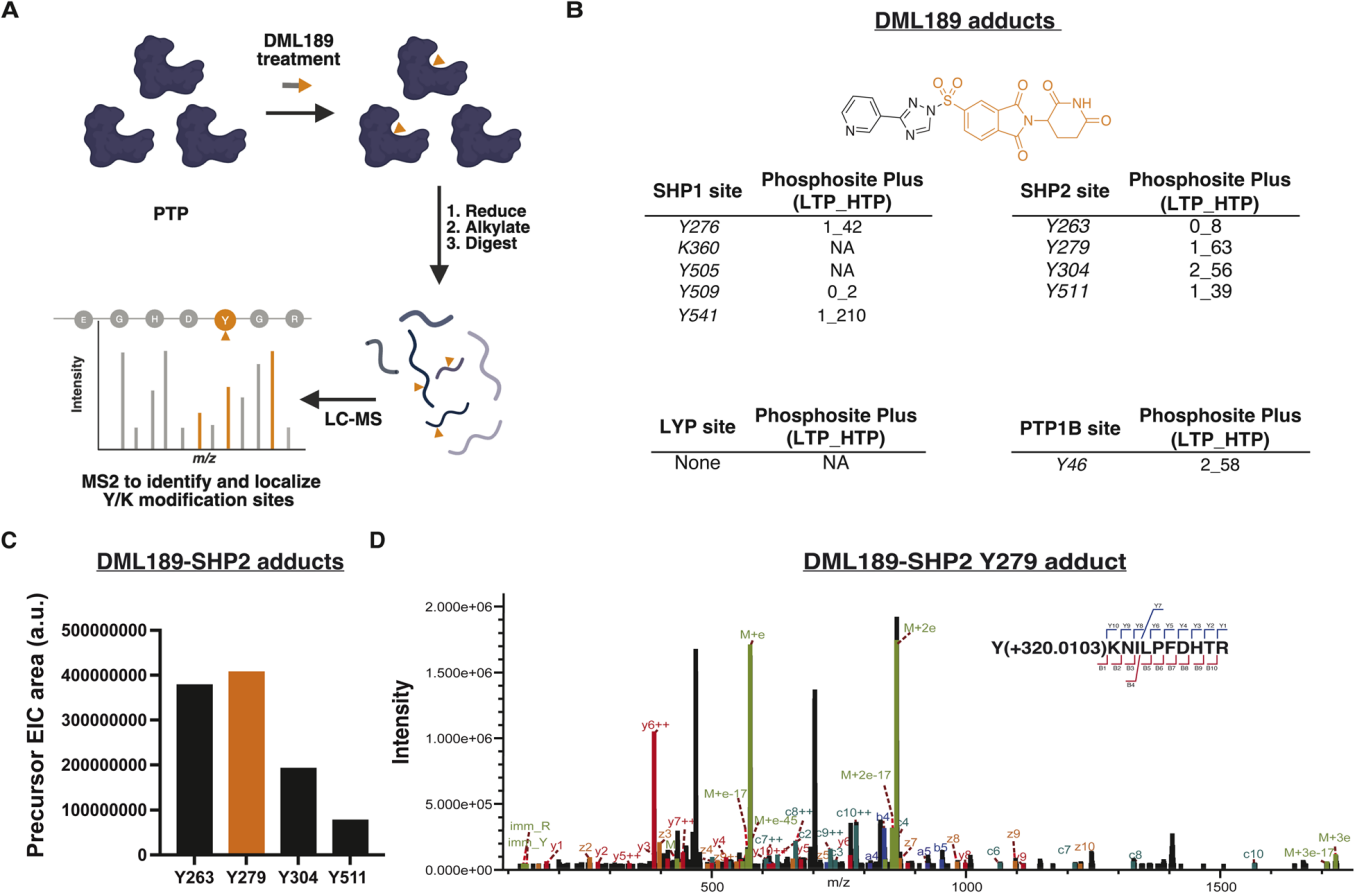
DML189 binds functional tyrosine sites in the SHP2 catalytic domain. (A) Workflow for detection of DML189 modification sites on PTPs *via* LC-MS/MS. Figure was generated using BioRender. (B) DML189 (+320.0103 Da) modification sites detected on SHP1, SHP2, PTP1B, and LYP. Peptide isoforms met the following quality control criteria: Byonic score ≥300, −5 ≤ ppm err ≤ 5, delta mod score ≥ 20. Phosphosite Plus (https://www.phosphosite.org) was used to identify sites with known post-translational phosphorylation. (C) Bar graph showing integrated precursor areas of DML189-SHP2 peptide adducts detected in the same injection. Data was analyzed using Byonic and Proteome Discoverer 3.0 (D) EThcD spectrum of DML189-SHP2 adduct localized on Y279.

To verify DML189 covalent binding, we performed LC-MS/MS to directly detect covalent adducts of DML189 on SHP1, SHP2, LYP, and PTP1B catalytic domains (Fig. 3A and Table S2). Sequence coverage for all PTPs evaluated by mass spectrometry was high (≥80%, Fig. S2A). We detected general binding to SHP1 (5 total sites), minimal binding on PTP1B (1 site), and no adducts on LYP (Fig. 3B). Using a combination of HCD and EThcD fragmentation, we localized DML189 adducts (+320.0103 Da) on SHP2 to Y263, Y279, Y304, and Y511 (Fig. 3B and S2B–D). Interestingly, the majority of DML189-modified tyrosines are also annotated phosphosites (Phosphosite Plus^28^ HTP > 10) with established roles in regulating SHP2 activity.^29–31^ We did not detect DML189 adducts on cysteine, lysine, or histidine sites on SHP2.

The principal sites on SHP2 modified by DML189 were Y263 and Y279 as determined by comparing MS1 EIC integrations (Fig. 3C and S2E). We deprioritized Y263 because of its low phosphosite annotation and validation (LTP = 0, HTP < 10). The Y279 residue forms part of the active site (*i.e.*, pTyr binding pocket) and plays a role in positioning a substrate pTyr for hydrolysis.^32^ Y279 is also in proximity to the catalytic cysteine (C459, 8.2 Å; PDB:3ZM0), and mutations at this tyrosine are inactivating and commonly found in individuals with LEOPARD syndrome.^33,34^ DML189 also modified the analogous Y279 positions in the SHP1 (Y276) and PTP1B (Y46) catalytic domains. Interestingly, mutations of the corresponding Y46 residue in PTP1B also impair catalytic activity.^35^

**Fig. 4:**
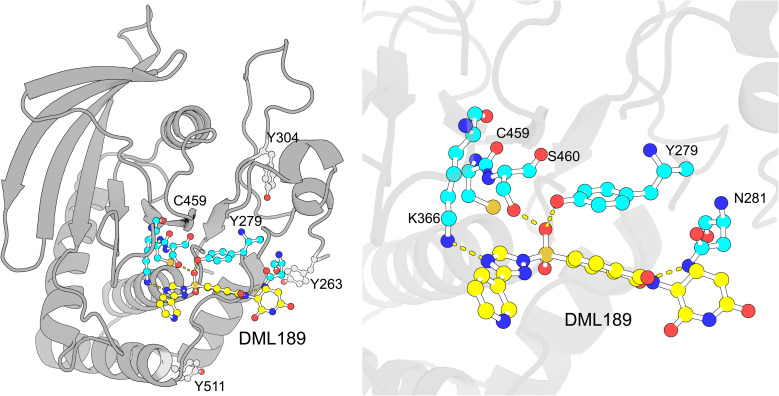
Docking predicts key DML189-SHP2 catalytic domain interactions. (Left) Lowest energy pose of DML189 docked at SHP2 Y279 using GOLD. Catalytic cysteine C459, Y263, Y304, and Y511 are highlighted. (Right) DML189 H-bonding interactions with K366, S460, and N281 in the catalytic pocket. Oxygen = red. Nitrogen = blue. Sulfur = orange. DML189 carbons = yellow. SHP2 residue carbons = cyan.

Since DML189 primarily binds Y279 near the SHP2 catalytic cysteine, we performed docking studies at this site to further probe the mechanism of inactivation. The lowest energy pose (−28.2 kcal mol^−1^) revealed multiple interactions of DML189 with key active site residues (Fig. 4 and Table S3). Specifically, the sulfonyl electrophile is positioned less than 3.9 Å from nucleophilic Y279 and forms hydrogen bonds (H-bond) with S460 (P-loop) and K366 (E-loop).^3^ The S460 residue has been shown to be critical for catalysis by interacting with incoming substrates *via* H-bonding to position them for nucleophilic attack by C459.^36^ Docking also revealed a predicted H-bond between the phthalimide nitrogen of DML189 and SHP2 N381. The docking studies at Y279 position DML189's electrophilic center closer to C459 (7.0 Å) compared to other solvent exposed and ligand modified positions (Y304 and Y511) on SHP2 (Fig. 4).

Based on the prior mutagenesis studies and the above modeling analyses, we conclude that modification of Y279 by DML189 likely inactivates SHP2 by altering the conformation of essential active site residues involved in substrate hydrolysis.

### SHP2 is sensitive to ligand induced protein tethering

**Fig. 5:**
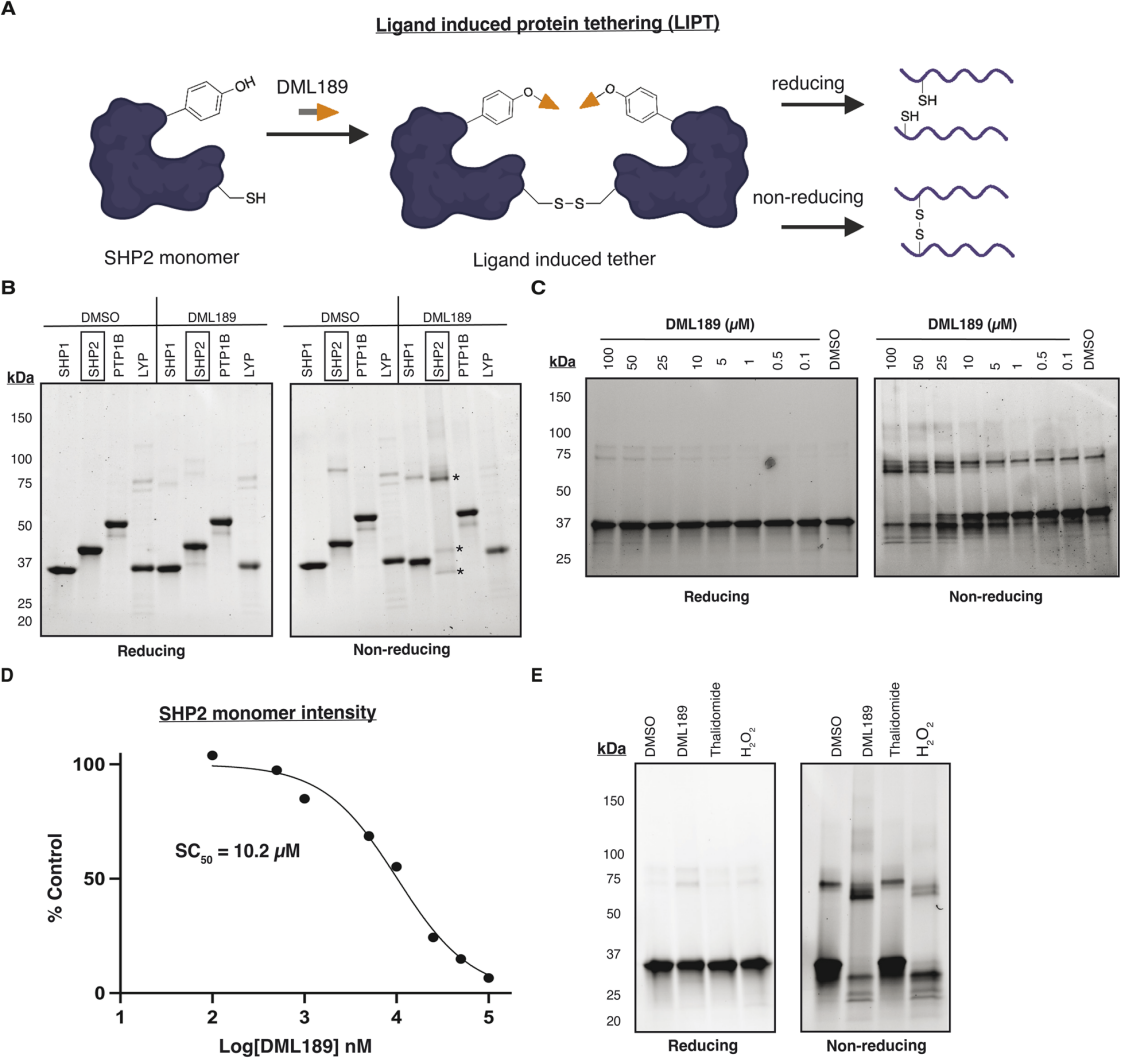
SHP2 is sensitive to DML189-mediated LIPT in a PTP-specific manner. (A) Workflow for evaluating PTP disulfide tethering with ligand treatment under reducing/non-reducing SDS-PAGE. Figure was generated using BioRender. (B) Evaluation of DML189 (150 µM, 1 h) dependent LIPT response on purified PTPs (SHP1, SHP2, PTP1B, LYP) using reducing/non-reducing SDS-PAGE. Asterisk indicates SHP2 inter- and intra-molecular disulfide linked species. (C) DML189 (0.1–100 µM, 1 h) LIPT dose response on SHP2. (D) Quantification of the integrated band intensity of SHP2 monomer to determine the stabilization concentration (SC_50_) of SHP2-DML189 LIPT dose response. (E) Reducing/non-reducing SDS-PAGE analysis of SHP2 disulfide tethering in response to DML189 (100 µM, 1 h), thalidomide (100 µM, 1 h), and H_2_O_2_ (500 µM, 1 h).

SHP2 undergoes reversible oxidation of its catalytic cysteine *via* disulfide bond formation between “backdoor cysteines” C333 and C367, which protects it from irreversible oxidation to sulfonic acid.^37^ Several studies also show that SHP2 dimerization is inactivating and can be mediated by disulfide bridges.^38,39^ We recently discovered that tyrosine-reactive SuTEx ligands can act as molecular glues *via* reversible disulfide tethering to stabilize and re-localize protein complexes in live cells.^40^ We therefore hypothesized that the proximity of the Y279 DML189 adduct to both the catalytic and backdoor cysteines could direct ligand induced protein tethering (LIPT) on SHP2 (Fig. 5A).

We therefore performed non-reducing (-BME) and reducing (+BME) SDS-PAGE analysis of purified PTPs treated with DML189 to detect LIPT events. DML189-treated SHP2 showed near complete loss of the catalytic monomer and subsequent formation of bands corresponding to a disulfide-tethered dimer and -oxidized monomer. In contrast, DML189 produced minimal LIPT effect on SHP1 and negligible effects on LYP or PTP1B (Fig. 5B). DML189-mediated LIPT on SHP2 was dose and time dependent with an estimated stabilization concentration (SC_50_) comparable to its biochemical potency (∼10 µM for measured IC_50_ and SC_50_, Fig. 5C, D and S3B). SHP2 concentration did not impact disulfide bond formation after treatment with DML189 (25 µM, 1 h) and DMSO alone did not induce tethering (Fig. S3A–C).

To test whether LIPT events were distinct from classical oxidative inactivation, we treated SHP2 with hydrogen peroxide. In contrast to DML189, the LIPT profile using hydrogen peroxide resulted in prominent intramolecular disulfide bonds and reduced tethered dimer formation (Fig. 5E). Additionally, we tested whether the parent thalidomide molecule produced the same LIPT effects on SHP2 but did not observe detectable activity (Fig. 5E). We evaluated whether reversal of LIPT using reducing agent also impacted enzymatic inhibition with DML189. Biochemical inactivation of both SHP proteins by DML189 was decreased in the presence of DTT (5 mM, 30 min) with SHP2 showing a more dramatic reversal compared to SHP1 (∼11- *vs.* 3-fold change in the observed IC_50_ values with DTT, respectively; Fig. S3D).

### DML189 induces unique tethering events on SHP2

**Fig. 6:**
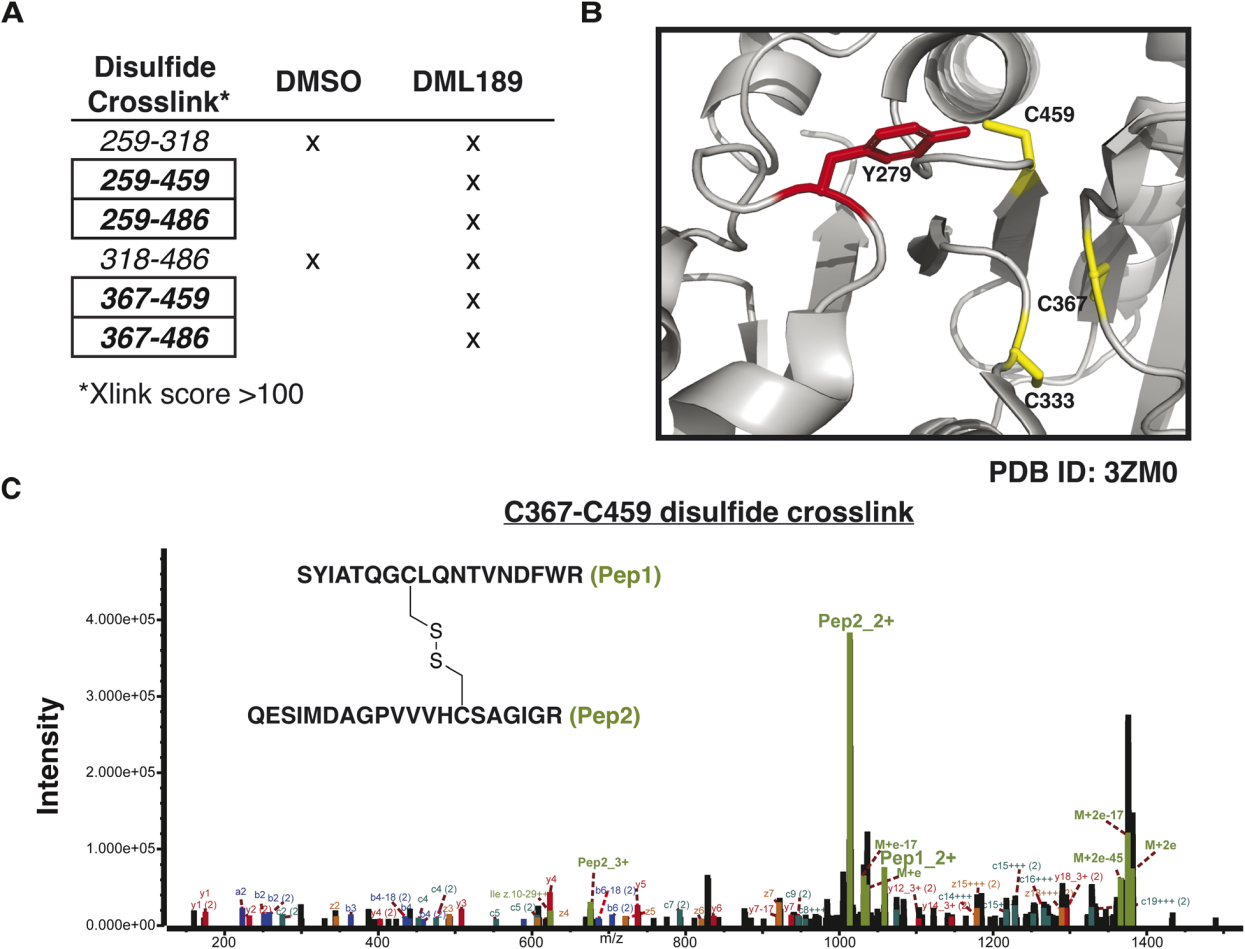
DML189 induces specific disulfide tethers on SHP2. (A) Detected disulfide bonds on SHP2 that met the following quality criteria: Byonic score ≥ 300, −5 ≤ ppm error ≤ 5, and Xlink score ≥ 100. Boxes indicate crosslinks unique to DML189 treated SHP2. (B) Crystal structure of SHP2 catalytic domain showing the proximity of Y279 to both the catalytic and backdoor regulatory cysteines. Figure generated using Pymol: PDB ID: 3ZM0 (C) EThcD spectrum of a DML189 specific disulfide tether on SHP2 involving the backdoor and catalytic cysteines, C367–C459.

To identify disulfide bonds mediating LIPT on SHP2, we performed non-reducing (-DTT), crosslinking LC-MS/MS. After DML189 treatment (75 µM, 1 h), SHP2 protein was immediately exposed to iodoacetamide to prevent disulfide reshuffling^41^ followed by sample processing. EThcD fragmentation was used to break disulfide linkages and fragment peptides sufficiently for spectral matching. Xlink analysis (Byonic) enabled disulfide linkage assignment (Table S4). DML189 induced four unique disulfide tethers on SHP2, including two with the catalytic cysteine (C459; Fig. 6A and B). Additional linkages involving the backdoor cysteine C367 were also detected (Fig. 6B and C). We searched for DML189 adducts and confirmed covalent modification of Y279 on the tethered SHP2 protein (Table S4). In congruence with our gel-based studies, the effect was specific to SHP2 and no detectable LIPT was identified on SHP1 *via* crosslinking LC-MS/MS.

## Discussion

PTPs are an emerging class of drug targets that could benefit from covalent strategies to achieve selectivity between related family members. The reliance on targeting the catalytic cysteine poses challenges for achieving selectivity given the high conservation of this catalytic mechanism across the PTP family. Here, we screened a library of tyrosine-reactive SuTEx ligands against purified PTPs to discover a SHP2 inhibitor with secondary molecular glue activity that stabilizes a SHP2 dimer *via* LIPT.

A distinguishing feature of the SHP2 inhibitor DML189 among the library members screened was the inclusion of a thalidomide binding element. Thalidomide and other immunomodulatory drugs (IMiDs) are known molecular glues that bind the E3 ligase cereblon (CRBN) to recruit neosubstrates for targeted protein degradation.^26^ To the best of our knowledge, IMiDs have not been reported to directly interact with SHP2, which we confirmed by the absence of biochemical inhibition and LIPT activity using free thalidomide (Fig. 5 and S1). Our studies combined with recent reports using a covalent lenalidomide^27^ support the ability to redirect IMiD substrate scope by inclusion of sulfonyl electrophiles into this drug modality.

DML189 showed increased potency (IC_50_) for SHP2 compared to other PTPs evaluated (Fig. 2). Furthermore, we detected direct covalent adducts of DML189 on SHP2 that were more localized compared to general SHP1 labeling; PTP1B and LYP modifications were reduced or absent, respectively. We identified Y279 as the primary site of DML189-SHP2 adduction as determined by mass spectrometry (Fig. 3). Docking studies at this SHP2 site showed key interactions between active site residues and DML189. Notably, we identified predicted H-bonds between S460 (P-loop) and K366 (E loop) and the pyridyl leaving group of DML189. S460 is key for catalysis through H-bond interactions to position substrates for nucleophilic attack by C459 (Fig. 4).^36^

Our recent discovery that SuTEx ligands can exhibit secondary molecular glue activity through LIPT motivated deeper investigations into DML189 binding mechanism. Interestingly, we found that SHP2 showed enhanced susceptibility to DML189-mediated LIPT, which could help explain the observed biochemical selectivity (Fig. 5). Crosslinking mass spectrometry identified disulfide tethering events that included the catalytic (C459) and backdoor cysteine (C367) to provide further insights into DML189-mediated inactivation of SHP2 (Fig. 6). DML189 was less effective as a biochemical inhibitor against SHP1, which could be explained partly by the weak but detectable LIPT activity against this PTP (Fig. 5). Additional studies are warranted to determine the relative contributions of LIPT *versus* covalent binding underlying the observed inhibitory activity of DML189 against PTPs.

We believe the current findings support a broader strategy for targeting the PTP family using tyrosine-reactive ligands. For example, DML189 covalently engages PTP1B Y46, a key residue that when mutated leads to reduced catalytic activity^35^ and increased substrate trapping (Y46F).^42^ Y46 forms side chain interactions with the main-chain atoms and the aromatic ring of pTyr.^32^ A hydrogen bond between the phenol group of Y46 and the side chain of S16 stabilizes the conformation of Y46 and its interaction with substrates.

While promising, our studies are currently limited to evaluation of purified PTP catalytic domains. Future studies should evaluate whether the biochemical and LIPT activity of DML189 is recapitulated against full length SHP2 and in more physiologically relevant environments. These studies are not trivial given the intricate regulation of PTP biology and the need to develop DML189 analogs with sufficient potency, selectivity and stability for cell biological evaluation. The stoichiometry of SHP2 dimerization and whether invoking dimerization is required for SHP2 inactivation using SuTEx ligands are also important questions for future investigations.

In summary, we demonstrate covalent targeting of tyrosines in the SHP2 PTP domain is a viable path for developing biochemical inhibitors that operate in part *via* a disulfide-tethering molecular glue mechanism.

## Author contributions

Conceptualization, MLW, DML, Z-YZ, and K-LH; data curation, MLW, DML, ZL, ZQ, SDV; formal analysis, MLW, DML, ZL, ZQ, SDV; investigation, MLW, DML, ZQ, QS, MLH, YB; methodology, MLW, DML, and ZQ; visualization, MLW, DML, QS, and ZL; writing, MLW, DML, K-LH, and Z-YZ; funding acquisition, Z-YZ and K-LH; resources, Z-YZ and K-LH; project administration, Z-YZ and K-LH; supervision, Z-YZ and K-LH.

## Conflicts of interest

K.-L.H. is a founder and scientific advisory board member of Hyku Biosciences.

## Supplementary Material

SC-017-D5SC07398G-s001

SC-017-D5SC07398G-s002

SC-017-D5SC07398G-s003

SC-017-D5SC07398G-s004

SC-017-D5SC07398G-s005

SC-017-D5SC07398G-s006

## Data Availability

The data supporting this article have been included as part of the supplementary information (SI). This paper does not report original code. Supplementary information is available. See DOI: https://doi.org/10.1039/d5sc07398g.
