# Supplementary material for: Protein tyrosine phosphatase inactivation by electrophilic tyrosine modification
Source: Chem Sci. 2026 Jan 19;17(10):5163–71. doi: 10.1039/d5sc07398g (PMC12814959; doi:10.1039/d5sc07398g)
Supplement: SC-017-D5SC07398G-s001 [file SC-017-D5SC07398G-s001.pdf]

Supplementary Information (SI) for Chemical Science.
This journal is © The Royal Society of Chemistry 2026

SUPPORTING INFORMATION

Protein tyrosine phosphatase inactivation by electrophilic tyrosine modification

Madeleine L. Ware[1], David M. Leace[1,2], Zihan Qu[3], Quentin Schaefer[1], Sagar D. Vaidya[1], Mikayla L. Horvath[1], Zhihong Li[1], Yunpeng Bai[4], Zhong-Yin Zhang*[3,4] and Ku-Lung Hsu*[1]

[1]Department of Chemistry, University of Texas at Austin, Austin, TX, 78712, USA
[2]Department of Pharmacology, University of Virginia School of Medicine, Charlottesville, VA, 22908, USA.
[3]James Tarpo Jr. and Margaret Tarpo Department of Chemistry, [4]Borch Department of Medicinal Chemistry and Molecular Pharmacology, Purdue University, West Lafayette, IN, 47907, USA

*Corresponding author. Email: zhang-zy@purdue.edu (Z.-Y.Z.); ken.hsu@austin.utexas.edu (K.-L.H.)

Contents

1. Supporting Figures
2. Chemical Synthesis
3. Biological Methods
4. References
5. Appendix

1. SUPPORTING FIGURES

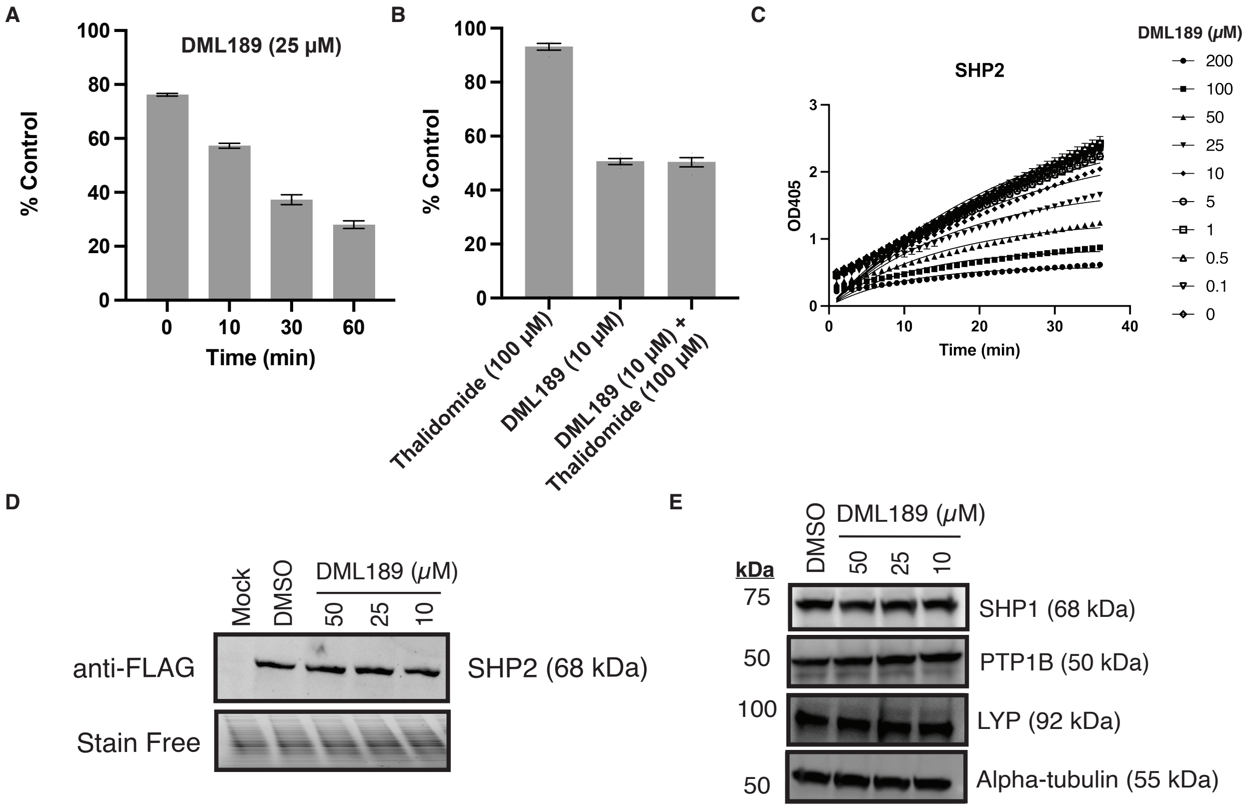

Supplementary Figure 1. (A) Time dependent inhibition (% vehicle control) of SHP2 by DML189 (25 µM). Data shown as mean ± SEM of % control from n =3 replicates. (B) SHP2 activity after treatment with thalidomide alone (100 µM, 1 h) and after co-incubation with DML189 (10 µM) and thalidomide (100 µM, 1 h). Data shown as mean ± SEM of % control from n =4 replicates. (C) SHP2 and pNPP (20 mM) reaction progress curves with DML189 (200-0.1 µM) treatment. Data are from n=3 replicates. (D) Western blot analysis of full length SHP2 protein levels after DML189 dose response (0-50 µM, 24 h) in live HEK 293T cells overexpressing recombinant SHP2. (E) Western blot analysis of SHP1, PTP1B, and LYP protein levels after DML189 treatment (0-50 µM, 24 h) in live Jurkat cells.

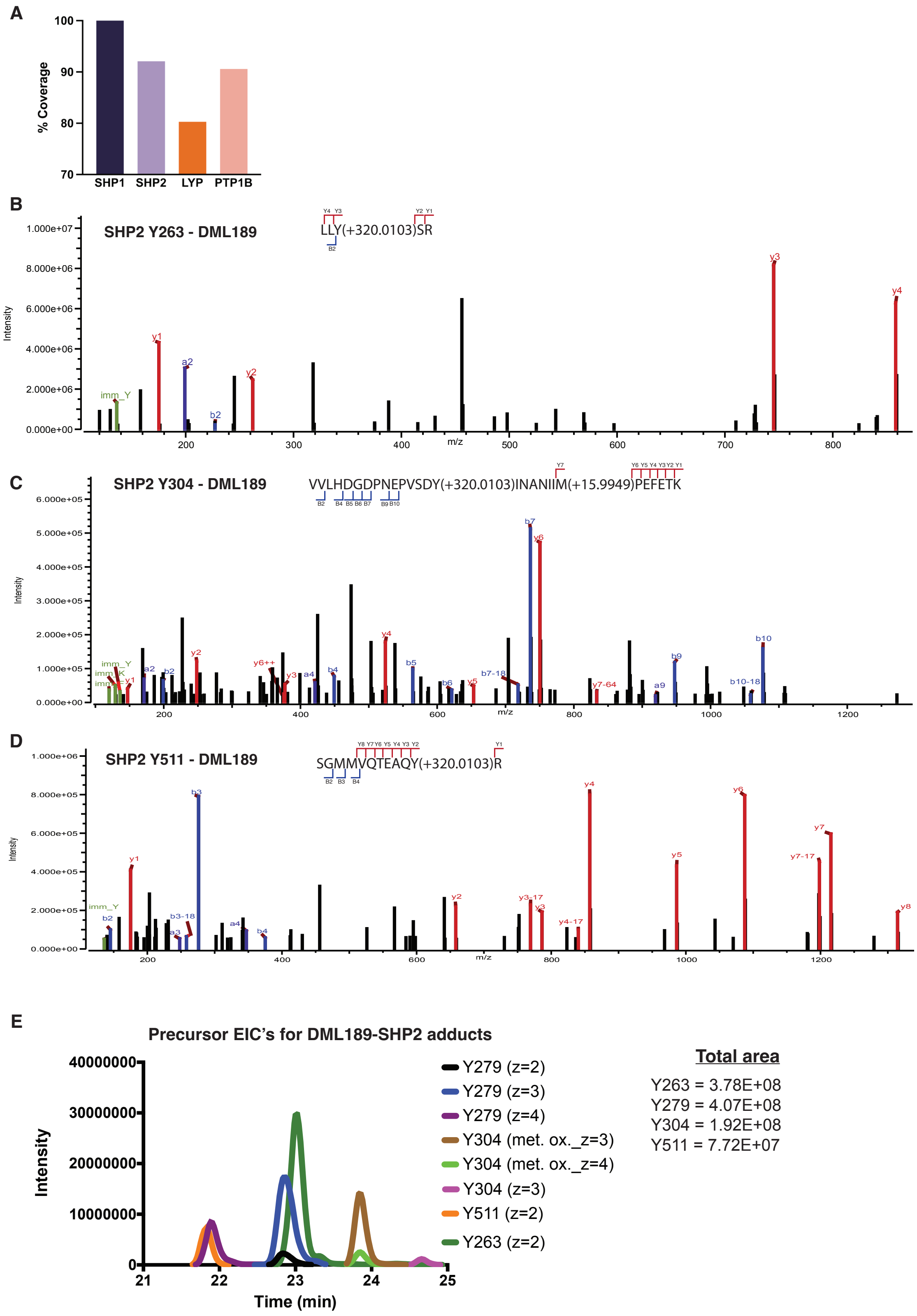
A
% Coverage
SHP1
SHP2
LYP
PTP1B
B
SHP2 Y263 - DML189
LLY(+320.0103)SR
Intensity
m/z
C
SHP2 Y304 - DML189
VVLHDGDPNEPVSDY(+320.0103)INANIIM(+15.9949)PEFETK
D
SHP2 Y511 - DML189
SGMMVQTEAQY(+320.0103)R
E
Precursor EIC's for DML189-SHP2 adducts
Intensity
Time (min)
Y279 (z=2)
Y279 (z=3)
Y279 (z=4)
Y304 (met. ox._z=3)
Y304 (met. ox._z=4)
Y304 (z=3)
Y511 (z=2)
Y263 (z=2)
Total area
Y263 = 3.78E+08
Y279 = 4.07E+08
Y304 = 1.92E+08
Y511 = 7.72E+07

Supplementary Figure 2. (A) Bar plot of % protein sequence coverage of PTP catalytic domains after trypsin digest and bottom-up LC-MS/MS analysis. (B-D) HCD spectra for SHP2-DML189 adducts at Y263, Y304 and Y511. Peptide spectra met the following quality control; Byonic score ≥ 300, $-5 \leq$ ppm err ≤ 5, delta mod score ≥ 20. (E) Extracted ion chromatograms (EICs) of detected DML189-SHP2 precursor adducts from a single sample injection. Precursor areas were integrated using Proteome Discoverer 3.0.

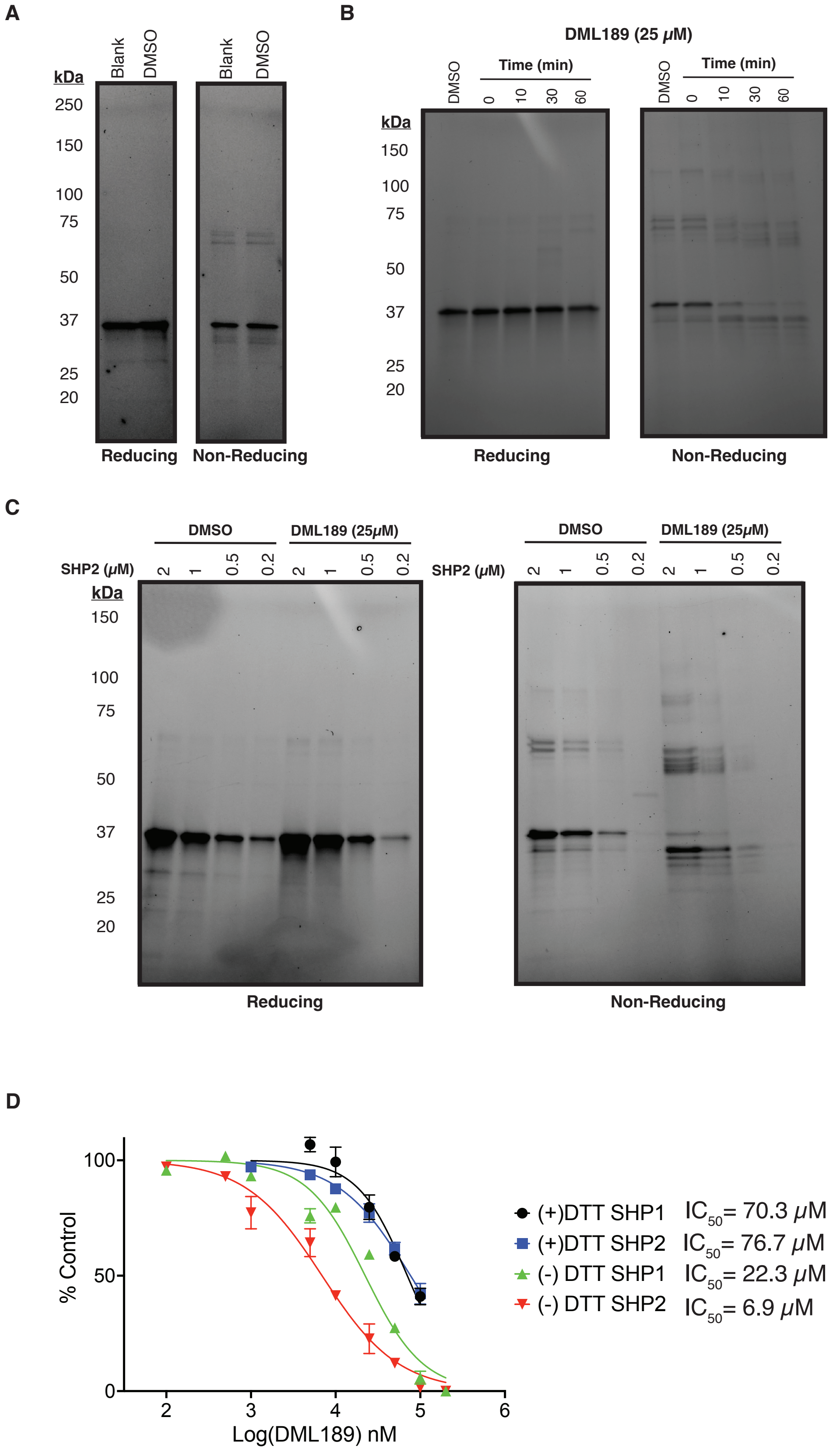
A
Blank
DMSO
Blank
DMSO
kDa
250
150
100
75
50
37
25
20
Reducing
Non-Reducing
B
DML189 (25 μM)
DMSO
Time (min)
0
10
30
60
DMSO
Time (min)
0
10
30
60
kDa
150
100
75
50
37
25
20
Reducing
Non-Reducing
C
DMSO
DML189 (25μM)
SHP2 (μM)
2
1
0.5
0.2
2
1
0.5
0.2
kDa
150
100
75
50
37
25
20
Reducing
DMSO
DML189 (25μM)
SHP2 (μM)
2
1
0.5
0.2
2
1
0.5
0.2
Non-Reducing
D
% Control
100
50
0
2
3
4
5
6
Log(DML189) nM
(+)DTT SHP1 IC50= 70.3 μM
(+)DTT SHP2 IC50= 76.7 μM
(-) DTT SHP1 IC50= 22.3 μM
(-) DTT SHP2 IC50= 6.9 μM

Supplementary Figure 3. (A) SHP2 was incubated with and without DMSO for 1 h at room temperature and analyzed by reducing and non-reducing SDS-PAGE. (B) Reducing and non-reducing SDS-PAGE analysis of DML189 (25 μM) incubated with SHP2 for 0, 10, 30, and 60 min. (C) Reducing and non-reducing SDS-PAGE analysis of DML189 (25 μM) incubated with the indicated concentrations of SHP2. (D) Reversal of DML189 inhibitory activity against SHP1 and SHP2 after treatment with DTT (5 mM, 30 min) as measured by pNPP substrate assay.

2. CHEMICAL SYNTHESIS

General synthetic procedure for 1, 2, 3 sulfonyl triazoles

Synthesis was performed as previously described.[1]

General synthetic procedure for 1, 2, 4 sulfonyl triazoles

Synthesis was performed as previously described.[2]

<u>Scheme 1. Synthesis of DML189</u>

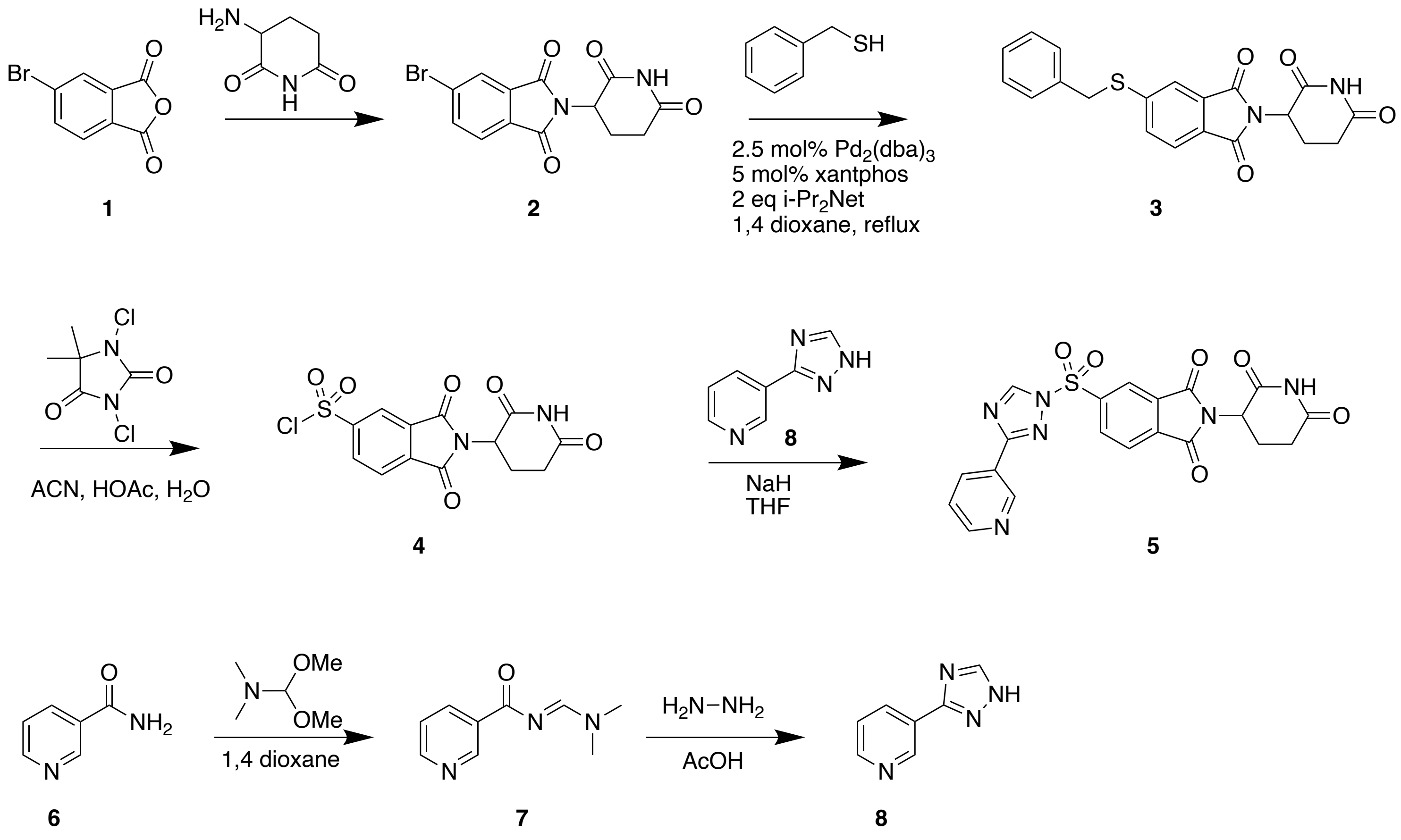

5-Bromo-2-(2,6-dioxo-3-piperidinyl)-1*H*-isoindole-1,3(2*H*)-dione (2)

10 g (60.8 mmol, 1.2 equiv) of aminoglutarimide HCl was dissolved in 550 mL of glacial acetic acid followed by addition of 11.5 g (50.7 mmol, 1 equiv) of bromo phthalic anhydride **1.** Next, 17.8 mL (127.7 mmol, 2.1 equiv) of triethylamine was added slowly, and the mixture was heated to reflux for 2h and monitored for starting material depletion. At completion, the reaction was cooled and poured onto ice. The precipitate was filtered through a glass frit, washed with cold water, and dried to yield 15.3 g of a purple powder (75%).

1*H*-Isoindole-1,3(2*H*)-dione, 2-(2,6-dioxo-3-piperidinyl)-5-[(phenylmethyl)thio]- (3)[3]

2 (4.37g, 13 mmol, 1 equiv)**,** benzyl mercaptan (1.53 mL, 91 mmol, 7 equiv), $Pd_2(dba)_3$ (299 mg, 0.325 mmol, 0.025 equiv), xantphos (377 mg, 0.65 mmol, 0.05 equiv), and i-Pr_2Net (4.6 mL, 26 mmol, 2 equiv) were dissolved in 27 mL of 1,4 dioxane and heated to reflux overnight. The next morning, the reaction mixture was cooled and dried on celite. The material was loaded on a biotage column and eluted with 0-50% EtOAc/DCM. The product was collected, precipitated, and washed with DCM to yield an off white solid (3.37g, 66% yield).

1*H*-Isoindole-5-sulfonyl chloride, 2-(2,6-dioxo-3-piperidinyl)-2,3-dihydro-1,3-dioxo- (4)[4]

3 (1g, 2.5 mmol, 1 equiv), acetic acid (0.94 mL), and water (0.625 mL) were added to 25 mL of ACN and cooled to -10 °C. DCDMH (0.99g, 5 mmol, 2 equiv) was then added slowly. After 1 hour the reaction mixture was diluted with 25 mL of DCM and cooled to 0°C. 25 mL $NaHCO_3$ was then added, and the solution was stirred for 10 minutes. The organic layer was then extracted, washed with brine, dried over Na_2SO_4, concentrated, precipitated, and filtered. After washing with cold DCM, the product was obtained as a bright white powder. H-NMR showed the product with presence of the hydrolyzed sulfonic acid.

3-(1*H*-1,2,4-Triazol-5-yl)pyridine (8)

Synthesis was performed as previously described.[5]

2-(2,6-Dioxopiperidin-3-yl)-5-((3-(pyridine-3-yl)-1*H*-1,2,4-triazol-1-yl)sulfonyl)isoindoline-1,3-dione (5, DML189)

To a flame dried flask was dissolved 1, 2, 4 triazole **8** (82 mg, 0.56 mmol, 1 equiv) in 2.5 mL of dry THF. The solution was cooled to 0° C and NaH (22.4 mg, 0.56 mmol, 1 equiv) was added. The reaction was stirred for 30 minutes after which sulfonyl chloride **4 (**200 mg, 0.56 mmol, 1 equiv) was added and the reaction was brought to room temperature and stirred overnight. The next morning, the precipitate was filtered, loaded onto celite, and purified with 0-20% MeOH/DCM on a biotage to yield a white solid (170 mg, 65% yield).

3. BIOLOGICAL METHODS

Purification of PTP catalytic domains

The cDNA encoding the catalytic domain of SHP1 (residues 245-543) was amplified by PCR and subcloned into pET21a(+) bacterial expression vector to allow expression of N-terminal His-tagged proteins. E. coli BL21(DE3) (Novagen) was used as an expression host and the induction of protein expression was carried out in LB Broth with 1 mM IPTG at 18 °C overnight. Cell pellets were stored at −80 °C for subsequent protein purification. Frozen cell pellets were lysed by sonication in 40 mL cold lysis buffer (50 mM Tris-HCl, pH 8.0, 150 mM NaCl, 5 mM imidazole, and 1 mM PMSF) per liter cell pellet. Cell lysates were clarified by centrifugation using a Beckman JA-18 rotor for 15 min at 5308 x g. The supernatant was incubated with HisPur Ni-NTA resin (Thermo Scientific) for 2 h and then packed onto a column and washed with 50 resin volume of buffer A (50 mM Tris-HCl, pH 8.0, 500 mM NaCl, 5 mM imidazole). The His-tagged protein was eluted with Buffer B (50 mM Tris-HCl, pH=8.0, 500 mM NaCl, 300 mM imidazole). Pooled His-protein-containing fractions were concentrated, loaded onto a HiLoad 26/600 Superdex 75 column (GE Healthcare Biosciences) and eluted with storage buffer (50 mM Tris-HCl, pH 8.0, 150 mM NaCl, 1 mM DTT, 10% glycerol). The purity of the protein was determined to be >95% by SDS-PAGE and Coomassie staining. The protein was aliquoted and stored at −80 °C. The catalytic domain of SHP2, PTP1B, and LYP were expressed and purified as described previously[6-9].

PTP activity assay for SuTEx ligand screen

SuTEx compounds were dissolved in DMSO to make 50 mM stock and transferred to assay plate. Ligands (500 µM) were incubated with SHP1/SHP2/PTP1B/LYP (400 nM) in assay buffer (50 mM 3,3-dimethylglutaric acid, pH 7.0, 18 mM NaCl, 1 mM EDTA, 0.01% Triton X-100) for 30 min to allow covalent labeling. Excessive substrate para-nitrophenol phosphate (pNPP, 20 mM) was added to the mixture and incubated for 2/2/0.5/10 min, respectively to monitor remaining enzyme activity.

DML189 dose responses and time dependence assays

0.025 nmols of PTP catalytic domain in assay buffer (50 mM Tris, 150 mM NaCl, pH 7.5) was treated with ligands (0-200 µM, 1h). pNPP (20 mM) was then added and the enzyme activity monitored by reading absorbance at 405nm. The above assay was performed with SHP2 treated with DML189 (25µM) where enzyme activity was monitored at 0, 10, 30, and 60 min.

DML189-SHP2 k_{inact}/KI calculation

DML189 (0-500 µM), PTP catalytic domain (0.025 nmols), and PNPP (20 mM) were incubated together in assay buffer (50 mM Tris, 150 mM NaCl, pH 7.5) and monitored for product formation using a plate reader for at least 30 min. Reaction progress curves were then fitted to the equation $Y=(V_0/k_{obs})(1-\exp(-k_{obs}X))$ in order to obtain k_{obs} values for each concentration of DML189. These k_{obs} values are then plotted against compound concentration. k_{inact}/K_I was calculated using the equation $k_{obs} = k_{inact}*[I]/(K_I+[I])$.

Assessment of thalidomide activity against SHP2

0.025 nmols of SHP2 catalytic domain in assay buffer (50 mM Tris, 150 mM NaCl, pH 7.5) was treated with thalidomide (100 µM, 1h) and enzyme activity was evaluated using pNPP (20 mM) as a substrate. Additionally, the above assay was performed after SHP2 was pre-incubated with thalidomide (100 µM) followed by DML189 (10 µM) for 1h at RT.

Testing DML189 degradative activity against PTPs

HEK293T cells were transiently transfected with full length SHP2 using polyethylenimine (PEI). After 48h, cells were treated with DML189 (50, 25, 10 µM) in serum free media for 24h. Cells were harvested and washed 3x in 1x DPBS. Jurkat cells were treated (DML189 (50, 25, 10 µM), 10^6/mL) and harvested to test for LYP, SHP1, and PTP1B degradation. Following whole cell lysis (50 mM Tris, 150 mM NaCl, pH 7.5, 1% Triton), lysates were normalized to 2 mg/mL and analyzed for PTP degradation via western blot: SHP2 (anti-FLAG, Sigma, Rabbit, 1:2500)), SHP1 (proteintech, Cat No. 24546-1-AP, 1:2000), PTP1B (proteintech, Cat No. 11334-1-AP), LYP (proteintech, Cat No. 11783-1-AP, 1:500).

Docking

A structure of SHP2 (PDB ID: 3ZM0) was obtained from PDB website. Residues within 10 Å of the binding site were defined as the docking pocket. Subsequently, compound was docked into this defined pocket using GOLD 5.1 with default parameters and 100 genetic algorithm (GA) runs. For each GA run, a maximum of 125,000 operations were performed. Docking was terminated when the top ten solutions had RMSD values within 1.5 Å. The resulting protein–ligand complexes were visualized using the PyMOL Molecular Graphics System (version 2.3.0).

Non-reducing / reducing SDS-PAGE of PTP catalytic domains

Catalytic domains of PTPs (SHP1, SHP2, PTP1B or LYP (0.5 mg/mL) in assay buffer (50 mM Tris, 150 mM NaCl, pH 7.5) were treated for 1h at RT with DML189 (150 µM) or DMSO. The samples were split in half and quenched with BME-containing or BME-free loading buffer and analyzed by stain free gel.

DML189 LIPT dose response on SHP2

SHP2 in assay buffer (50 mM Tris, 150 mM NaCl, pH 7.5) at 0.132 mg/mL was treated for 1h at RT with DML189 (100-0.1 µM) or DMSO. The samples were split in half and quenched with BME-containing or BME-free loading buffer and analyzed by stain free gel.

SHP2 dilution LIPT analysis with DML189

Various concentrations of SHP2 (2-0.2 µM) were treated with DMSO or DML189 (25 µM) and analyzed by reducing/non-reducing SDS-PAGE followed by stain free imaging.

SHP2 thalidomide and H_2O_2 LIPT response

SHP2 in assay buffer (50 mM Tris, 150 mM NaCl, pH 7.5) at 0.132 mg/mL was treated at RT with thalidomide (100 µM, 1h) or H_2O_2 (500 µM, 1h). The samples were split in half and quenched with BME-containing or BME-free loading buffer and analyzed by stain free gel

DTT reversibility assay

0.025 nmols SHP1 and SHP2 catalytic domain were treated with DML189 (0-100 µM, 1h) in assay buffer (50 mM Tris, 150 mM NaCl, pH 7.5). After DML189 incubation, samples were treated with DTT (5 mM, 30 min) followed by PTP activity readout using pNPP (20 mM).

Sample preparation for DML189 adduct detection by LC-MS/MS

1 nmol of SHP1, SHP2, LYP, and PTP1B catalytic domains were treated with DML189 (100 µM, 1h, RT) in assay buffer (50 mM Tris, 150 mM NaCl, pH 7.4). Following treatment, samples were loaded onto a 10,000 Da MWCO filter and washed 3x with 25mM ammonium bicarbonate. After transfer to Eppendorf tubes, samples were reduced (DTT, 20 mM, 65°C, 15 min), alkylated (iodoacetamide, 40 mM, RT, 30 min), and trypsinized (3h, 37°C). Trypsin was quenched with 1µL acetic acid prior to injection (150 ng) on the LC-MS.

Sample preparation for crosslinking LC-MS/MS

SHP1 and SHP2 (1 nmol) were treated with DML189 (75 µM, 1h, RT) or DMSO and then immediately quenched with iodoacetamide (40mM, 30 min, RT) to prevent disulfide reshuffling. Samples were loaded onto a 10,000 Da MWCO filter and washed 3x with 25 mM ammonium bicarbonate. After washing, samples were trypsinized (1.5h, 37°C) and quenched with 1µL formic acid before injection onto the LC-MS.

LC-MS/MS acquisition and analysis for DML189 adduct detection on PTP catalytic domains

Peptides from DML189 treated PTP's were evaluated by LC-MS/MS using a Vanquish Neo UHPLC coupled to an Orbitrap Eclipse Tribrid mass spectrometer. Peptides were separated on a 30 min gradient by reverse phase LC using 3 µm C18 (20 cm) as follows: (A: 0.1% formic acid/H2O; B: 80% ACN, 0.1% formic acid in H2O): 0–1 min 15% B, 450 nL/min; 1–8.5 min 40% B, 300 nL/min; 8.5–16 min 60% B, 300 nL/min; 16–20 min 99% B, 300 nL/min; 20–30 min 1% B. Data were acquired using a top 20 ddMS2 method. MS1 spectra were acquired at 120K resolution with a max IT of 20 ms and MS2 spectra were taken with a normalized AGC of 50%, quadrupole isolation width of 1.6 m/z, and max IT of 100 ms. DML189 (+320.0181 Da) adducts on PTP domains were searched as modifications on tyrosine, lysine, histidine, and cysteine using Byonic. Peptides met the following quality criteria: PMI-Byonic Score ≥ 300, delta ppm err. 5, delta mod score ≥ 20.

Data acquisition and analysis for crosslinking LC-MS/MS.

Non-reduced peptides from DML189 treated PTP's were evaluated by LC-MS/MS using a Vanquish Neo UHPLC coupled to an Orbitrap Eclipse Tribrid mass spectrometer. Peptides were separated on a 30 min gradient by reverse phase LC using 3 µm C18 (20 cm) as follows: (A: 0.1% formic acid/H2O; B: 80% ACN, 0.1% formic acid in H2O): 0–1 min 15% B, 450 nL/min; 1–8.5 min 40% B, 300 nL/min; 8.5–16 min 60% B, 300 nL/min; 16–20 min 99% B, 300 nL/min; 20–30 min 1% B. Calibrated charge-state dependent ETD activation followed by a supplemental HCD at 30% CE was used to fragment disulfide-bound peptides. MS2 spectra were taken at 15k resolution, an absolute AGC of 1E5 and maximum injection time of 200 ms. Disulfide linked peptides were identified using Byonic S-S Xlink node (S-S cleavable linkage, –2.015650 Da) with the following quality control criteria: PMI-Byonic Score ≥ 300, delta ppm err. 5, and Xlink score of >100.

4. REFERENCES

1. A. M. Ciancone, K. W. Seo, M. Chen, A. L. Borne, A. H. Libby, D. L. Bai, R. E. Kleiner and K.-L. Hsu, Global Discovery of Covalent Modulators of Ribonucleoprotein Granules, *J Am Chem Soc*, 2023, **145**, 11056-11066.
2. J. W. Brulet, A. L. Borne, K. Yuan, A. H. Libby and K. L. Hsu, Liganding Functional Tyrosine Sites on Proteins Using Sulfur-Triazole Exchange Chemistry, *J Am Chem Soc*, 2020, **142**, 8270-8280.
3. T. Itoh and T. Mase, A General Palladium-Catalyzed Coupling of Aryl Bromides/Triflates and Thiols, *Org Lett*, 2004, **6**, 4587-4590.
4. Y.-M. Pu, A. Christesen and Y.-Y. Ku, A simple and highly effective oxidative chlorination protocol for the preparation of arenesulfonyl chlorides, *Tetrahedron Lett*, 2010, **51**, 418-421.
5. R. J. Grams, W. J. Wolfe, R. J. Seal, J. Veccia and K.-L. Hsu, Discovery and Optimization of a Covalent AKR1C3 Inhibitor, *J Med Chem*, 2025, **68**, 9465-9478.
6. M. Taing, Y. F. Keng, K. Shen, L. Wu, D. S. Lawrence and Z. Y. Zhang, Potent and highly selective inhibitors of the protein tyrosine phosphatase 1B, *Biochemistry*, 1999, **38**, 3793-3803.
7. Z. H. Yu, R. Y. Zhang, C. D. Walls, L. Chen, S. Zhang, L. Wu, S. Liu and Z. Y. Zhang, Molecular basis of gain-of-function LEOPARD syndrome-associated SHP2 mutations, *Biochemistry*, 2014, **53**, 4136-4151.
8. Y. He, S. Liu, A. Menon, S. Stanford, E. Oppong, A. M. Gunawan, L. Wu, D. J. Wu, A. M. Barrios, N. Bottini, A. C. Cato and Z. Y. Zhang, A potent and selective small-molecule inhibitor for the lymphoid-specific tyrosine phosphatase (LYP), a target associated with autoimmune diseases, *J Med Chem*, 2013, **56**, 4990-5008.
9. S. Grosskopf, C. Eckert, C. Arkona, S. Radetzki, K. Böhm, U. Heinemann, G. Wolber, J.-P. von Kries, W. Birchmeier and J. Rademann, Selective Inhibitors of the Protein Tyrosine Phosphatase SHP2 Block Cellular Motility and Growth of Cancer Cells in vitro and in vivo, *ChemMedChem*, 2015, **10**, 815-826.

5. APPENDIX

A. Published sulfonyl triazole compounds used for PTP screening. Synthesis and characterization are reported in References [1, 2, 5]:

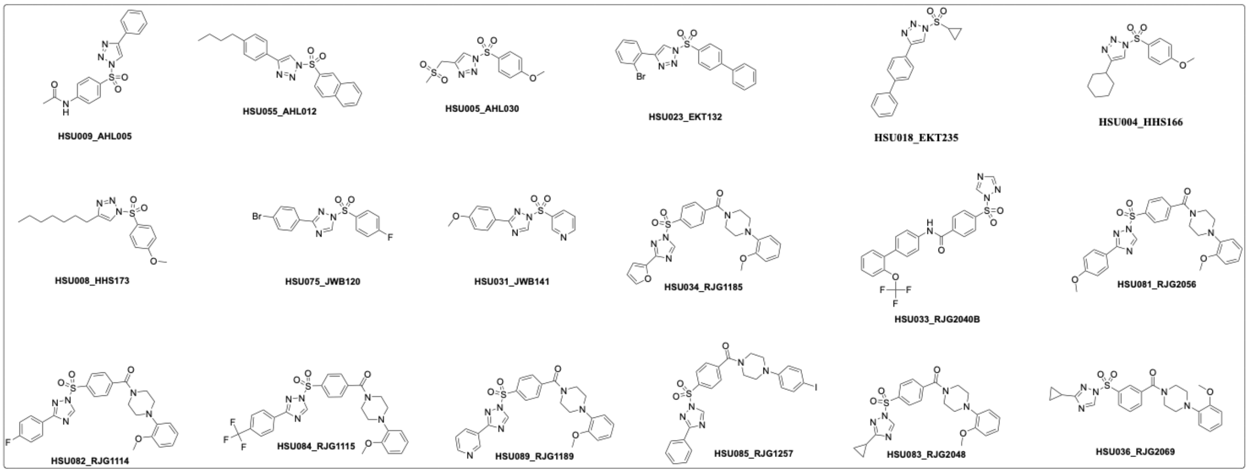

B. Compound characterization of remaining sulfonyl-triazoles used in PTP screen:

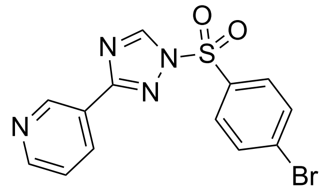

HSU001-JWB179

Chemical Formula: $C_{13}H_{10}BrN_4O_2S$
Exact Mass: 364.9708

^{1}H NMR (800 MHz, DMSO-D6) δ 9.56 (s, 1H), 9.15 (d, *J* = 2.4 Hz, 1H), 8.70 (dd, *J* = 4.8, 1.7 Hz, 1H), 8.33 (dt, *J* = 7.9, 1.9 Hz, 1H), 8.10 – 8.07 (m, 2H), 7.98 – 7.94 (m, 2H), 7.55 (dd, *J* = 7.9, 4.8 Hz, 1H).

^{13}C NMR (201 MHz, DMSO-D6) δ 162.06, 151.36, 148.15, 147.26, 134.34, 134.26, 133.47, 130.74, 130.24, 124.75, 124.23.

HRMS (ESI-TOF) *m/z*: $[M + H]^+$ calculated for $C_{13}H_{11}BrN_4O_2S$ 364.9708, Found, 364.9695.

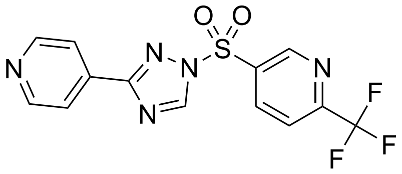

HSU002_JWB222

Chemical Formula: $C_{13}H_8F_3N_5O_2S$
Exact Mass: 355.0351

^{1}H NMR (800 MHz, DMSO-D6) δ 9.63 (s, 1H), 9.51 (d, *J* = 2.3 Hz, 1H), 8.88 (dd, *J* = 8.4, 2.4 Hz, 2H), 8.77 (d, *J* = 6.2 Hz, 2H), 8.28 (dd, *J* = 8.4, 0.8 Hz, 2H), 8.01 (d, *J* = 6.2 Hz, 2H).

^{13}C NMR (201 MHz, DMSO-D6) δ 162.25, 151.35 (q, *J* = 34.8 Hz), 149.84, 149.37, 148.83, 147.17, 139.90, 136.66, 135.19, 122.35, 121.01.

^{19}F NMR (376 MHz, acetone-D6) δ -69.04 (s, 3F).

HRMS (ESI-TOF) *m/z*: $[M + H]^+$ calculated for $C_{13}H_9F_3N_5O_2S$ 356.0424, Found, 356.0432.

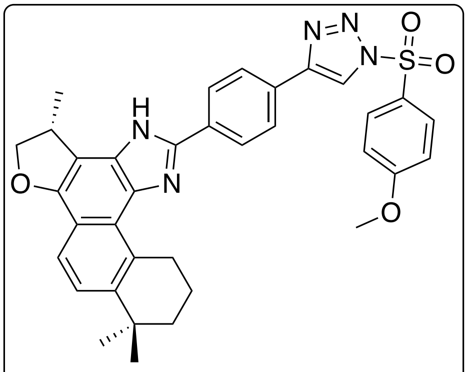

HSU003 (CPT03)

Chemical Formula: $C_{35}H_{33}N_5O_4S$
Exact Mass: 619.2253

^{1}H NMR (800 MHz, Acetone-D6) δ 9.11 (s, 1H), 8.41 (s, 1H), 8.24 (d, *J* = 9.2 Hz, 2H), 8.17 (d, *J* = 8.0 Hz, 2H), 7.92 (s, 1H), 7.61 (d, *J* = 8.6 Hz, 1H), 7.33 (d, *J* = 9.2 Hz, 2H), 4.97 (s, 1H), 4.51 (s, 1H), 4.03 (s, 3H), 2.14 (p, *J* = 2.2 Hz, 2H), 2.10 (m, 3H), 1.92 – 1.85 (m, 2H), 1.58 (d, *J* = 6.9 Hz, 3H), 1.49 (s, 3H), 1.48 (s, 3H).

^{13}C NMR (201 MHz, Acetone-D6) δ 166.62, 153.99, 147.62, 147.01, 144.11, 133.26, 132.04, 132.00, 130.31, 127.92, 127.27, 127.09, 123.93, 123.86, 121.25, 121.18, 120.61, 118.14, 116.34, 116.25, 111.13, 79.84, 79.76, 56.69, 39.75, 37.52, 35.19, 32.33, 31.75, 20.69, 19.99.

HRMS (ESI-TOF) *m/z*: $[M + H]^+$ calculated for $C_{35}H_{34}N_5O_4S$ 620.2332, Found, 620.2302.

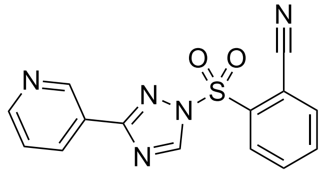

HSU006_JWB226

Chemical Formula: $C_{14}H_{10}N_5O_2S$
Exact Mass: 312.0555

^{1}H NMR (400 MHz, Acetone-D6) δ 9.33 (s, 1H), 9.22 (s, 1H), 8.69 (d, *J* = 4.8 Hz, 1H), 8.55 (d, *J* = 7.6 Hz, 1H), 8.35 (d, *J* = 7.9 Hz, 1H), 8.21 (d, *J* = 7.3 Hz, 1H), 8.13 (m, 2H), 7.50 (dd, *J* = 8.0, 4.8 Hz, 1H).

^{13}C NMR (100 MHz, Acetone-D6) δ 164.00, 152.53, 148.82, 148.81, 137.57, 137.20, 135.21, 134.81, 132.71, 125.95, 124.67, 115.33, 112.11.

HRMS (ESI-TOF) *m/z*: $[M + H]^+$ calculated for $C_{14}H_{11}N_5O_2S$ 312.0555, Found, 312.0559.

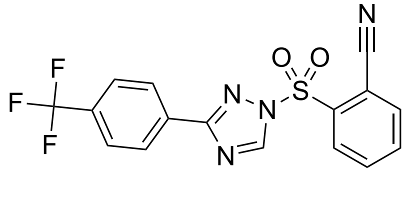

HSU010_JWB228
Chemical Formula: $C_{16}H_{10}F_3N_4O_2S$
Exact Mass: 379.0477

^{1}H NMR (800 MHz, DMSO-D6) δ 8.65 (dd, *J* = 7.1, 3.9 Hz, 1H), 8.29 – 8.19 (m, 2H), 7.92 – 7.83 (m, 3H), 7.77 (dd, *J* = 7.6, 1.3 Hz, 1H), 7.64 (td, *J* = 7.7, 1.3 Hz, 1H), 7.49 (td, *J* = 7.6, 1.3 Hz, 1H).

^{13}C NMR (201 MHz, DMSO-D6) δ 163.06, 150.38, 148.64, 146.04, 133.96, 132.25, 129.01, 127.36, 127.07, 126.48, 125.73(m, 1C), 125.70, 117.67, 108.95.

^{19}F NMR (376 MHz, Acetone-D6) δ -63.44 (s, 3F).

HRMS (ESI-TOF) *m/z*: $[M + H]^+$ calculated for $C_{16}H_{10}F_3N_4O_2S$ 379.0471, Found, 379.0470.

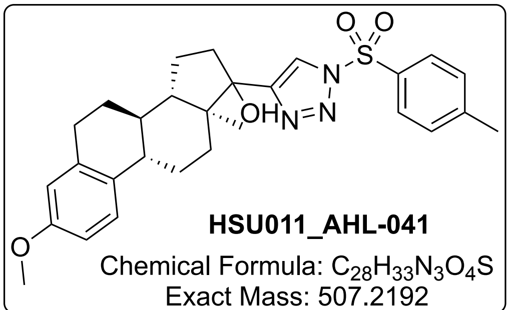

HSU011_AHL-041
Chemical Formula: $C_{28}H_{33}N_3O_4S$
Exact Mass: 507.2192

^{1}H NMR (400 MHz, Acetone-D6) δ 8.34 (s, 1H), 8.04 (d, *J* = 8.3 Hz, 2H), 7.55 (d, *J* = 8.1 Hz, 2H), 7.09 (d, *J* = 8.5 Hz, 1H), 6.64 (dd, *J* = 8.5, 2.7 Hz, 1H), 6.59 (s, 1H), 3.72 (s, 3H), 3.40 (q, *J* = 7.0 Hz, 1H), 3.11 (br s, 1H), 2.80 (m, 1H), 2.45 (s, 3H), 2.43 – 2.26 (m, 1H), 2.21 – 2.06 (m, 2H), 2.00 – 1.79 (m, 3H), 1.80 – 1.67 (m, 1H), 1.63 – 1.51 (m, 1H), 1.45 (q, *J* = 11.4 Hz, 1H), 1.38 – 1.23 (m, 2H), 1.11 (t, *J* = 7.0 Hz, 2H), 1.04 (s, 3H), 0.55 (td, *J* = 12.9, 4.2 Hz, 1H).

^{13}C NMR (100 MHz, Acetone-D6) δ 158.49, 155.97, 148.47, 138.49, 134.43, 133.05, 131.50, 129.25, 126.99, 122.82, 114.39, 112.21, 82.57, 66.11, 55.27, 49.22, 48.19, 44.37, 40.47, 38.41, 33.80, 28.25, 27.06, 24.42, 21.67, 15.61, 14.72.

HRMS (ESI-TOF) *m/z*: $[M + H]^+$ calculated for $C_{28}H_{34}N_3O_4S$ 508.2265, Found, 508.2257.

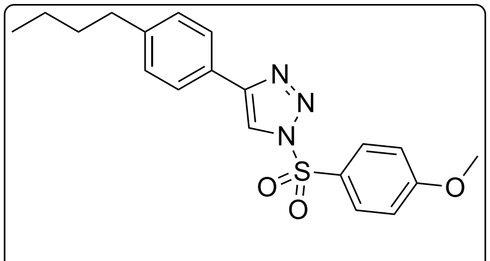

HSU012_AHL-011

Chemical Formula: $C_{19}H_{21}N_3O_3S$
Exact Mass: 371.1304

^{1}H NMR (400 MHz, DMSO-D6) δ 9.31 (s, 1H), 8.10 (d, *J* = 9.1 Hz, 2H), 7.83 (d, *J* = 8.4 Hz, 2H), 7.29 (d, *J* = 8.5 Hz, 2H), 7.25 (d, *J* = 9.1 Hz, 2H), 3.87 (s, 3H), 2.60 (t, *J* = 7.6 Hz, 2H), 1.56 (p, *J* = 7.6 Hz, 2H), 1.29 (dq, *J* = 14.3, 7.2 Hz, 2H), 0.89 (t, *J* = 7.3 Hz, 3H).

^{13}C NMR (151 MHz, DMSO-D6) δ 165.28, 147.03, 143.47, 130.99, 128.99, 126.21, 126.15, 125.79, 120.73, 115.77, 56.26, 34.61, 32.98, 21.74, 13.80.

HRMS (ESI-TOF) *m/z*: $[M + H]^+$ calculated for $C_{19}H_{22}N_3O_3S$ 372.1376, Found, 372.1369.

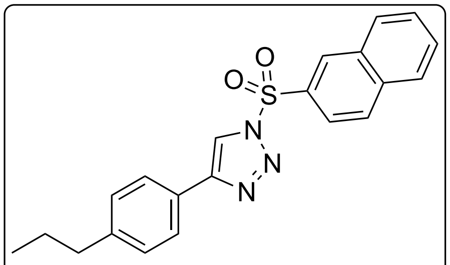

HSU013_AHL-015

Chemical Formula: $C_{21}H_{19}N_3O_2S$
Exact Mass: 377.1198

^{1}H NMR (400 MHz, DMSO-D6) δ 9.41 (s, 1H), 8.99 (d, *J* = 2.1 Hz, 1H), 8.33 (d, *J* = 8.1 Hz, 1H), 8.26 (d, *J* = 8.9 Hz, 1H), 8.16 – 8.08 (m, 1H), 8.05 (dd, *J* = 8.8, 2.1 Hz, 1H), 7.88 – 7.80 (m, 3H), 7.77 (ddd, *J* = 8.1, 7.0, 1.3 Hz, 1H), 7.28 (d, *J* = 8.1 Hz, 2H), 2.56 (t, *J* = 7.5 Hz, 2H), 1.58 (h, *J* = 7.4 Hz, 2H), 0.87 (t, *J* = 7.3 Hz, 3H).

^{13}C NMR (100 MHz, Acetone-D6) δ 148.31, 144.50, 136.90, 134.02, 132.87, 131.88, 131.49, 131.42, 130.75, 129.85, 129.29, 129.03, 127.55, 126.76, 122.85, 120.91, 38.26, 25.14, 13.95.

HRMS (ESI-TOF) *m/z*: $[M + H]^+$ calculated for $C_{21}H_{20}N_3O_2S$ 378.1271, Found, 378.1272.

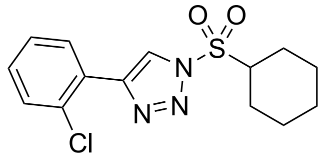

HSU014_EKT116
Chemical Formula: $C_{14}H_{16}ClN_3O_2S$
Exact Mass: 325.0652

^{1}H NMR (400 MHz, DMSO-D6) δ 9.03 (s, 1H), 8.04 (dd, *J* = 6.7, 2.8 Hz, 1H), 7.64 (dd, *J* = 7.2, 2.1 Hz, 1H), 7.57 – 7.46 (m, 2H), 4.10 (tt, *J* = 11.9, 3.6 Hz, 1H), 1.99 (d, *J* = 11.0 Hz, 2H), 1.80 (d, *J* = 13.2 Hz, 2H), 1.60 (d, *J* = 13.0 Hz, 1H), 1.45 (m, 2H), 1.31 (m, 2H), 1.13 (m, 1H).

^{13}C NMR (151 MHz, DMSO-D6) δ 143.17, 131.21, 130.70, 130.46, 130.35, 127.71, 127.46, 124.94, 63.41, 25.46, 24.19, 23.91.

HRMS (ESI-TOF) *m/z*: $[M + H]^+$ calculated for $C_{14}H_{17}ClN_3O_2S$ 326.0725, Found, 326.0721.

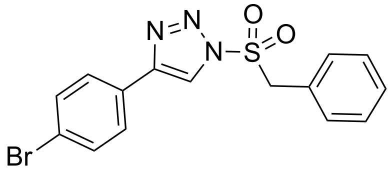

HSU015_EKT151
Chemical Formula: $C_{15}H_{12}BrN_3O_2S$
Exact Mass: 376.9834

^{1}H NMR (400 MHz, $CDCl_3$) δ 7.78 (s, 1H), 7.58 (q, *J* = 8.5 Hz, 4H), 7.36 (t, *J* = 7.4 Hz, 1H), 7.29 (t, *J* = 7.3 Hz, 2H), 7.12 (d, *J* = 7.4 Hz, 2H), 4.87 (s, 2H).

^{13}C NMR (100 MHz, DMSO-D6) δ 145.32, 132.07, 130.88, 129.45, 128.82, 128.07, 127.77, 126.30, 122.79, 122.15, 60.07.

HRMS (ESI-TOF) *m/z*: $[M + H]^+$ calculated for $C_{15}H_{12}BrN_3O_2S$ 377.9906, Found, 377.9888.

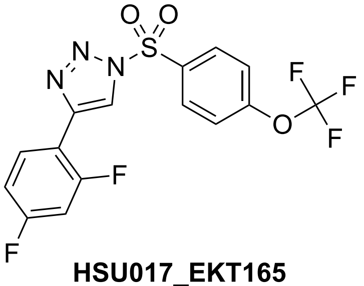

HSU017_EKT165
Chemical Formula: $C_{15}H_8F_5N_3O_3S$
Exact Mass: 405.0207

^{1}H NMR (400 MHz, Acetone-D6) δ 8.82 (d, *J* = 2.0 Hz, 1H), 8.42 (d, *J* = 7.7 Hz, 2H), 8.23 (q, *J* = 8.7 Hz, 1H), 7.72 (d, *J* = 9.0 Hz, 2H), 7.40 – 7.02 (m, 2H).

^{13}C NMR (100 MHz, Acetone-D6) δ 164.08 (d, *J* = 237.5 Hz), 160.47 (dd, *J* = 251.3, 12.0 Hz), 155.07 (d, *J* = 2.0 Hz), 141.23, 135.35, 132.45, 130.31 (dd, *J* = 10.0, 4.6 Hz), 123.33 (d, *J* = 11.7 Hz), 122.82, 121.06 (d, *J* = 258.8 Hz), 114.63 (d, *J* = 13.4 Hz), 113.13 (dd, *J* = 21.9, 3.6 Hz), 105.30 (t, *J* = 26.0 Hz).

^{19}F NMR (376 MHz, Acetone-D6) δ -58.51 (s, 3F), -109.77 – -110.07 (m, 1F), -110.25 – -110.58 (m, 1F).

HRMS (ESI-TOF) *m/z*: $[M + H]^+$ calculated for $C_{15}H_9F_5N_3O_3S$ 406.0279, Found, 406.0281.

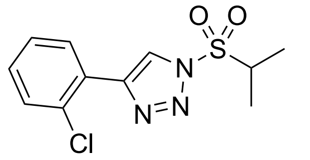

HSU019_EKT122
Chemical Formula: $C_{11}H_{12}ClN_3O_2S$
Exact Mass: 285.0339

^{1}H NMR (400 MHz, $CDCl_3$) δ 8.69 (s, 1H), 8.26 (dd, *J* = 7.8, 2.5 Hz, 1H), 7.50 (d, *J* = 8.0 Hz, 1H), 7.42 (t, *J* = 7.5 Hz, 1H), 7.35 (td, *J* = 7.6, 1.4 Hz, 1H), 3.90 (hept, *J* = 7.2 Hz, 1H), 1.49 (s, 3H), 1.47 (s, 3H).

^{13}C NMR (100 MHz, DMSO-D6) δ 143.20, 131.28, 130.75, 130.53, 130.34, 127.73, 127.49, 125.15, 56.99, 15.58.

HRMS (ESI-TOF) *m/z*: $[M + H]^+$ calculated for $C_{11}H_{13}ClN_3O_2S$ 286.0412, Found, 286.0400.

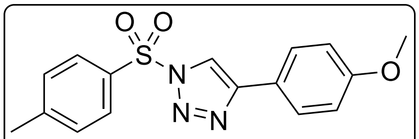

HSU020_EKT080
Chemical Formula: $C_{16}H_{15}N_3O_3S$
Exact Mass: 329.0834

^{1}H NMR (400 MHz, DMSO-D6) δ 8.22 (s, 1H), 7.78 (d, *J* = 8.7 Hz, 2H), 7.48 (d, *J* = 8.1 Hz, 2H), 7.12 (d, *J* = 7.9 Hz, 2H), 7.02 (d, *J* = 8.8 Hz, 2H), 3.79 (s, 3H), 2.29 (s, 3H).

^{13}C NMR (151 MHz, DMSO-D6) δ 160.81, 151.47, 147.06, 137.31, 132.10, 130.65, 128.21, 128.12, 120.05, 114.67, 55.35, 21.21.

HRMS (ESI-TOF) *m/z*: $[M + H]^+$ calculated for $C_{16}H_{16}N_3O_3S$ 364.0907, Found, 364.0907.

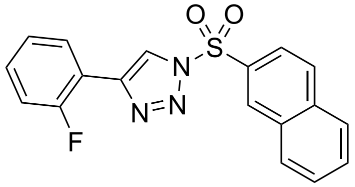

HSU022_EKT101
Chemical Formula: $C_{18}H_{12}FN_3O_2S$
Exact Mass: 353.0634

^{1}H NMR (400 MHz, $CDCl_3$) δ 8.79 (s, 1H), 8.55 (d, *J* = 3.4 Hz, 1H), 8.25 (t, *J* = 7.6 Hz, 1H), 8.02 (m, 3H), 7.92 (d, *J* = 8.3 Hz, 1H), 7.72 (t, *J* = 7.5 Hz, 1H), 7.67 (t, *J* = 7.5 Hz, 1H), 7.34 (q, *J* = 6.8 Hz, 1H), 7.23 (t, *J* = 7.6 Hz, 1H), 7.19 – 7.10 (m, 1H).

^{13}C NMR (100 MHz, DMSO-D6) δ 159.98, 157.50, 140.72 (d, *J* = 2.3 Hz), 135.66, 131.96, 131.54, 131.33, 131.11 (d, *J* = 8.5 Hz), 130.92, 130.75, 130.03, 128.53, 128.14, 125.12 (d, *J* = 3.4 Hz), 123.32 (d, *J* = 10.4 Hz), 121.89, 116.24 (d, *J* = 21.0 Hz).

^{19}F NMR (376 MHz, Acetone-D6) δ -114.71 (dtd, *J* = 11.4, 8.0, 3.8 Hz, 1F).

HRMS (ESI-TOF) *m/z*: $[M + H]^+$ calculated for $C_{18}H_{13}FN_3O_2S$ 354.0707, Found, 354.0709.

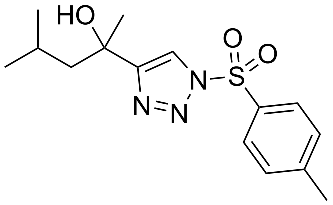

HSU024_AHL-009
Chemical Formula: $C_{15}H_{21}N_3O_3S$
Exact Mass: 323.1304

^{1}H NMR (400 MHz, DMSO-D6) δ 8.59 (s, 1H), 7.67 (s, 1H), 7.48 (d, *J* = 8.1 Hz, 2H), 7.12 (d, *J* = 8.3 Hz, 2H), 2.29 (s, 3H), 1.67 – 1.62 (m, 2H), 1.58 (dt, *J* = 12.7, 6.0 Hz, 1H), 1.45 (s, 3H), 0.80 (d, *J* = 6.5 Hz, 3H), 0.70 (d, *J* = 6.5 Hz, 3H).

^{13}C NMR (151 MHz, DMSO-D6) δ 145.32, 137.90, 128.16, 125.52, 69.66, 51.56, 29.65, 24.38, 24.15, 23.82, 20.81.

HRMS (ESI-TOF) *m/z*: $[M + H]^+$ calculated for $C_{15}H_{22}N_3O_3S$ 324.1376, Found, 324.1377.

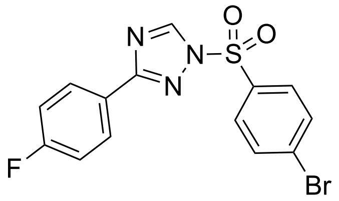

HSU25_JWB164
Chemical Formula: $C_{14}H_{10}BrFN_3O_2S$
Exact Mass: 381.9661

^{1}H NMR (800 MHz, DMSO-D6) δ 8.59 (s, 1H), 8.15 – 7.98 (m, 2H), 7.60 – 7.47 (m, 4H), 7.34 (m, 2H).

^{13}C NMR (201 MHz, DMSO-D6) δ 163.43, 162.22, 147.70, 146.27, 130.61, 128.23, 127.76, 121.57, 115.96, 115.85.

^{19}F NMR (376 MHz, DMSO-D6) δ -113.88 (td, *J* = 8.9, 4.5 Hz, 1F).

HRMS (ESI-TOF) *m/z*: $[M + H]^+$ calculated for $C_{14}H_{11}BrFN_3O_2S$ 382.9656, Found, 382.9648.

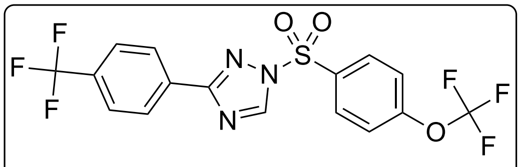

HSU026_JWB154
Chemical Formula: $C_{16}H_9F_6N_3O_3S$
Exact Mass: 437.0269

^{1}H NMR (600 MHz, DMSO-D6) δ 9.57 (s, 1H), 8.43 – 8.28 (m, 2H), 8.19 (d, *J* = 8.1 Hz, 2H), 7.86 (d, *J* = 8.1 Hz, 2H), 7.77 – 7.66 (m, 2H).

^{13}C NMR (201 MHz, DMSO-D6) δ 163.39, 158.41, 153.34, 148.66, 147.95, 146.48, 128.11, 127.80, 127.04, 126.62 (m, 1C), 126.32 (m, 1C), 120.74.

^{19}F NMR (376 MHz, Acetone-D6) δ -58.51 (s, 3F), -63.43 (s, 3F).

HRMS (ESI-TOF) *m/z*: $[M + H]^+$ calculated for $C_{16}H_{10}F_6N_3O_3S$ 438.0342, Found, 438.0343.

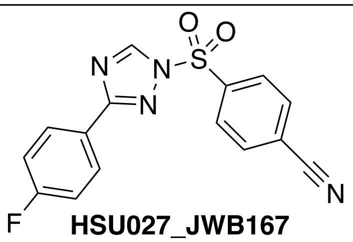

HSU027_JWB167
Chemical Formula: $C_{15}H_9FN_4O_2S$
Exact Mass: 328.0430

^{1}H NMR (800 MHz, DMSO-D6) δ 8.72 (s, 1H), 8.12 – 7.99 (m, 2H), 7.86 – 7.78 (m, 2H), 7.78 – 7.68 (m, 2H), 7.47 – 7.24 (m, 2H).

^{13}**C NMR** (201 MHz, DMSO-D6) δ 163.61, 162.39, 156.82, 152.55, 146.02, 132.10, 128.42 (d, *J* = 8.8 Hz), 126.43, 118.69, 116.02 (d, *J* = 22.0 Hz), 111.09.

^{19}**F NMR** (376 MHz, Acetone-D6) δ -111.70 (ddd, *J* = 13.7, 8.9, 5.3 Hz, 1F).

HRMS (ESI-TOF) *m/z*: $[M + H]^+$ calculated for $C_{15}H_{10}FN_4O_2S$ 329.0503, Found, 329.0498.

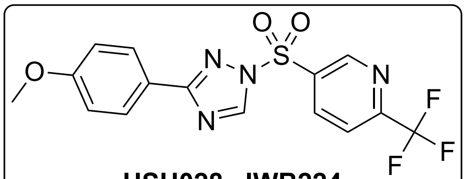

HSU028_JWB224
Chemical Formula: $C_{15}H_{11}F_3N_4O_3S$
Exact Mass: 384.0504

^{1}**H NMR** (400 MHz, Acetone-D6) δ 9.48 (s, 1H), 9.17 (s, 1H), 8.89 (d, *J* = 8.3 Hz, 1H), 8.24 (d, *J* = 8.3 Hz, 1H), 8.02 (d, *J* = 8.9 Hz, 2H), 7.03 (d, *J* = 8.9 Hz, 2H), 3.85 (s, 3H).

^{13}**C NMR** (100 MHz, Acetone-D6) δ 166.13, 162.81, 153.09 (d, *J* = 35.2 Hz), 150.26, 148.01, 140.23, 137.07, 129.38, 122.79 (q, *J* = 2.9 Hz), 122.34, 120.39, 115.07, 55.77.

^{19}**F NMR** (376 MHz, Acetone-D6) δ -69.04 (s, 1F).

HRMS (ESI-TOF) *m/z*: $[M + H]^+$ calculated for $C_{15}H_{12}F_3N_4O_3S$ 385.0577, Found, 385.0590.

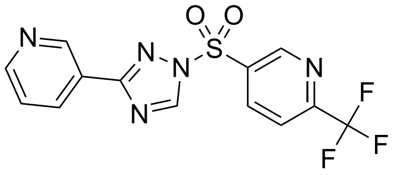

HSU029_JWB221
Chemical Formula: $C_{13}H_8F_3N_5O_2S$
Exact Mass: 355.0351

^{1}**H NMR** (800 MHz, DMSO-D6) δ 9.59 (s, 1H), 9.50 (d, *J* = 2.4 Hz, 1H), 9.18 (d, *J* = 1.2 Hz, 1H), 8.88 (dd, *J* = 8.4, 2.4 Hz, 1H), 8.72 (dd, *J* = 4.8, 1.7 Hz, 1H), 8.39 – 8.32 (m, 1H), 8.28 (d, *J* = 8.4 Hz, 1H), 7.58 – 7.51 (m, 1H).

^{13}**C NMR** (201 MHz, DMSO-D6) δ 162.38, 151.63, 151.29 (d, *J* = 35.0 Hz), 149.27, 148.58, 147.49, 139.80, 135.32, 134.31, 124.62, 124.20, 122.33 (q, *J* = 2.9 Hz), 121.31, 119.94.
^{19}F NMR (376 MHz, acetone) δ -69.04 (s, 3F).

HRMS (ESI-TOF) *m/z*: $[M + H]^+$ calculated for $C_{13}H_9F_3N_5O_2S$ 356.0424, Found, 356.0419.

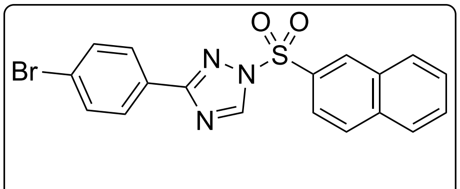

HSU030_JWB123
Chemical Formula: $C_{18}H_{12}BrN_3O_2S$
Exact Mass: 412.9834

^{1}H NMR (800 MHz, DMSO-D6) δ 8.61 (s, 1H), 8.14 (d, *J* = 0.8 Hz, 1H), 7.99 – 7.94 (m, 3H), 7.92 – 7.88 (m, 1H), 7.86 (d, *J* = 8.3 Hz, 1H), 7.73 – 7.69 (m, 3H), 7.54 – 7.50 (m, 2H).

^{13}C NMR (201 MHz, DMSO-D6) δ 146.20, 145.58, 132.72, 132.15, 131.89, 128.46, 127.93, 127.45, 127.31, 126.43, 126.29, 124.04, 123.97, 122.73.

HRMS (ESI-TOF) *m/z*: $[M + H]^+$ calculated for $C_{18}H_{13}BrN_3O_2S$ 413.9906, Found, 413.9907.

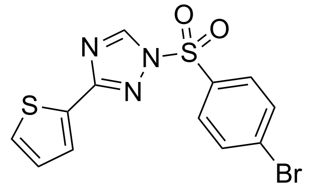

HSU032_JWB209
Chemical Formula: $C_{12}H_8BrN_3O_2S_2$
Exact Mass: 368.9241

^{1}H NMR (400 MHz, DMSO-D6) δ 9.45 (s, 1H), 8.06 (d, *J* = 8.8 Hz, 2H), 7.97 (d, *J* = 9.0 Hz, 2H), 7.76 (dd, *J* = 5.0, 1.2 Hz, 1H), 7.71 (dd, *J* = 3.7, 1.2 Hz, 1H), 7.18 (dd, *J* = 5.0, 3.7 Hz, 1H).

^{13}C NMR (100 MHz, DMSO-D6) δ 160.38, 148.03, 134.40, 133.55, 130.76, 130.23, 129.82, 128.77, 128.53, 127.83.

HRMS (ESI-TOF) *m/z*: $[M + H]^+$ calculated for $C_{12}H_9BrN_3O_2S_2$ 369.9314, Found, 369.9309.

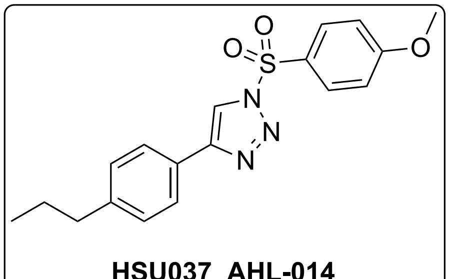

HSU037_AHL-014
Chemical Formula: $C_{18}H_{19}N_3O_3S$
Exact Mass: 357.1147

^{1}H NMR (400 MHz, DMSO-D6) δ 9.32 (s, 1H), 8.10 (d, *J* = 9.1 Hz, 2H), 7.83 (d, *J* = 8.3 Hz, 2H), 7.29 (d, *J* = 8.6 Hz, 2H), 7.25 (d, *J* = 9.1 Hz, 2H), 3.87 (s, 3H), 2.58 (t, *J* = 7.5 Hz, 2H), 1.59 (h, *J* = 7.4 Hz, 2H), 0.88 (t, *J* = 7.3 Hz, 3H).

^{13}**C NMR** (100 MHz, DMSO-D6) δ 165.22, 146.98, 143.18, 130.94, 128.99, 126.23, 126.12, 125.72, 120.72, 115.72, 56.21, 36.97, 23.91, 13.57.

HRMS (ESI-TOF) *m/z*: $[M + H]^+$ calculated for $C_{18}H_{20}N_3O_3S$ 358.1220, Found, 358.1220.

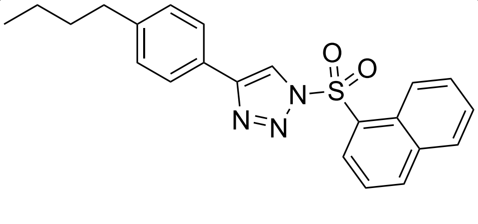

HSU038_AHL-017
Chemical Formula: $C_{22}H_{21}N_3O_2S$
Exact Mass: 391.1354

^{1}**H NMR** (400 MHz, DMSO-D6) δ 9.53 (s, 1H), 8.65 (t, *J* = 7.3 Hz, 2H), 8.51 (d, *J* = 8.3 Hz, 1H), 8.17 (d, *J* = 7.3 Hz, 1H), 7.85 (t, *J* = 7.9 Hz, 1H), 7.79 (d, *J* = 8.2 Hz, 3H), 7.71 (t, *J* = 7.5 Hz, 1H), 7.25 (d, *J* = 8.3 Hz, 2H), 2.56 (t, *J* = 7.7 Hz, 2H), 1.52 (p, *J* = 7.6 Hz, 2H), 1.26 (h, *J* = 7.3 Hz, 2H), 0.85 (t, *J* = 7.3 Hz, 3H).

^{13}**C NMR** (100 MHz, DMSO-d6) δ 146.90, 143.51, 138.16, 133.82, 132.86, 129.94, 128.95, 127.90, 127.08, 126.00, 125.77, 125.10, 122.95, 121.26, 34.57, 32.94, 21.69, 13.76.

HRMS (ESI-TOF) *m/z*: $[M + H]^+$ calculated for $C_{22}H_{22}N_3O_2S$ 392.1427, Found, 392.1427.

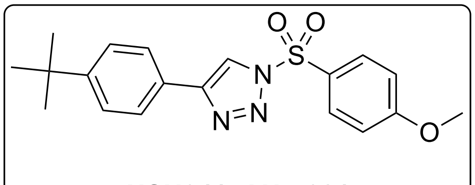

HSU041_AHL-021
Chemical Formula: $C_{19}H_{21}N_3O_3S$
Exact Mass: 371.1304

^{1}**H NMR** (400 MHz, DMSO-D6) δ 9.33 (s, 1H), 8.10 (d, *J* = 9.1 Hz, 2H), 7.85 (d, *J* = 8.5 Hz, 2H), 7.49 (d, *J* = 8.5 Hz, 2H), 7.25 (d, *J* = 9.1 Hz, 2H), 3.88 (s, 3H), 1.29 (s, 9H).

^{13}**C NMR** (100 MHz, DMSO-D6) δ 165.25, 151.64, 146.88, 130.96, 126.10, 125.98, 125.83, 125.59, 120.79, 115.75, 56.23, 34.49, 31.00.

HRMS (ESI-TOF) *m/z*: $[M + H]^+$ calculated for $C_{19}H_{22}N_3O_3S$ 372.1376, Found, 372.1377.

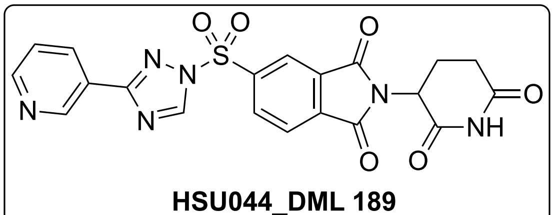

HSU044_DML 189
Chemical Formula: $C_{20}H_{14}N_6O_6S$
Exact Mass: 466.0696

^{1}H NMR (400 MHz, Acetone-D6) δ 10.04 (s, 1H), 9.35 (s, 1H), 9.25 (s, 1H), 8.81 – 8.64 (m, 2H), 8.59 (s, 1H), 8.42 (d, *J* = 8.0 Hz, 1H), 8.25 (d, *J* = 8.0 Hz, 1H), 7.56 (dd, *J* = 8.0, 4.9 Hz, 1H), 5.23 (dd, *J* = 12.9, 5.4 Hz, 1H), 2.98 (ddd, *J* = 17.6, 14.4, 5.4 Hz, 1H), 2.84 – 2.68 (m, 2H), 2.25 (ddt, *J* = 13.0, 5.7, 2.7 Hz, 2H).

^{13}C NMR (100 MHz, Acetone-D6) δ 172.50, 169.55, 166.24, 166.02, 163.78, 151.90, 148.55, 148.26, 142.24, 138.13, 136.12, 135.47, 134.00, 132.98, 125.89, 124.95, 124.13, 50.79, 31.84, 22.98.

HRMS (ESI-TOF) *m/z*: $[M + H]^+$ calculated for $C_{20}H_{15}N_6O_6S$ 467.0768, Found, 467.0768.

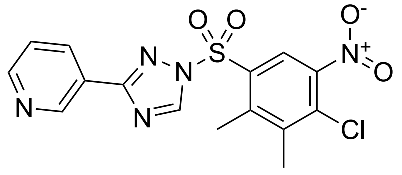

HSU050_DML367
Chemical Formula: $C_{15}H_{12}ClN_5O_4S$
Exact Mass: 393.0299

^{1}H NMR (400 MHz, Acetone-D6) δ 9.38 (s, 1H), 9.23 (s, 1H), 8.69 (s, 2H), 8.37 (dd, *J* = 8.1, 1.9 Hz, 1H), 7.52 (dd, *J* = 8.0, 4.6 Hz, 1H), 2.86 (s, 3H), 2.56 (s, 3H).

^{13}C NMR (100 MHz, Acetone-D6) δ 163.54, 152.49, 148.82, 148.35, 145.02, 143.31, 135.09, 134.86, 133.33, 126.03, 125.44, 124.71, 18.39, 17.85.

HRMS (ESI-TOF) *m/z*: $[M + H]^+$ calculated for $C_{15}H_{12}ClN_5O_4S$ 394.0371, Found, 394.0358.

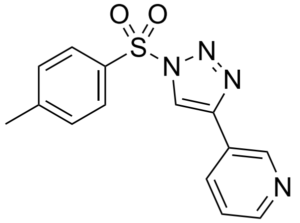

HSU053_AHL-001
Chemical Formula: $C_{14}H_{12}N_4O_2S$
Exact Mass: 300.0681

^{1}H NMR (400 MHz, Acetone-D6) δ 9.16 (s, 2H), 8.61 (d, *J* = 4.9 Hz, 1H), 8.31 (dd, *J* = 8.0, 1.8 Hz, 1H), 8.10 (d, *J* = 8.8 Hz, 2H), 7.58 (d, *J* = 8.1 Hz, 2H), 7.48 (dd, *J* = 8.0, 4.8 Hz, 1H), 2.48 (s, 3H).

^{13}C NMR (100 MHz, Acetone-D6) δ 150.85, 148.86, 148.14, 145.42, 133.99, 133.87, 131.63, 129.45, 126.24, 124.67, 122.07, 21.69.

HRMS (ESI-TOF) *m/z*: $[M + H]^+$ calculated for $C_{14}H_{13}BrN_4O_2S$ 301.0754, Found, 301.0755.

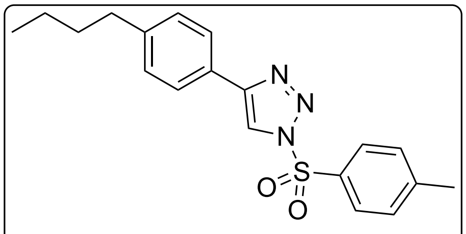

HSU054_AHL-010
Chemical Formula: $C_{19}H_{21}N_3O_2S$
Exact Mass: 355.1354

^{1}H NMR (400 MHz, DMSO-D6) δ 9.34 (s, 1H), 8.05 (d, *J* = 8.5 Hz, 2H), 7.83 (d, *J* = 8.3 Hz, 2H), 7.56 (d, *J* = 8.8 Hz, 2H), 7.29 (d, *J* = 8.2 Hz, 2H), 2.60 (t, *J* = 7.6 Hz, 2H), 2.42 (s, 3H), 1.56 (p, *J* = 7.3 Hz, 2H), 1.30 (h, *J* = 7.1 Hz, 2H), 0.89 (t, *J* = 7.3 Hz, 3H).

^{13}C NMR (151 MHz, DMSO-D6) δ 147.68, 147.07, 143.44, 132.40, 130.87, 128.92, 128.22, 126.09, 125.76, 120.87, 34.56, 32.92, 21.68, 21.26, 13.74.

HRMS (ESI-TOF) *m/z*: $[M + H]^+$ calculated for $C_{19}H_{22}N_3O_2S$ 356.1427, Found, 356.1426.

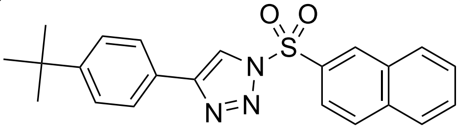

HSU056_AHL-020
Chemical Formula: $C_{22}H_{21}N_3O_2S$
Exact Mass: 391.1354

^{1}H NMR (400 MHz, DMSO-D6) δ 9.42 (s, 1H), 9.00 (d, *J* = 2.2 Hz, 1H), 8.33 (d, *J* = 8.3 Hz, 1H), 8.27 (d, *J* = 9.1 Hz, 1H), 8.12 (d, *J* = 7.1 Hz, 1H), 8.05 (dd, *J* = 8.8, 2.1 Hz, 1H), 7.91 – 7.80 (m, 3H), 7.77 (ddd, *J* = 8.2, 7.0, 1.4 Hz, 1H), 7.48 (d, *J* = 8.8 Hz, 2H), 1.28 (s, 9H).

^{13}C NMR (100 MHz, DMSO-D6) δ 151.74, 147.05, 135.62, 132.19, 131.55, 131.02, 130.92, 130.81, 130.04, 128.58, 128.17, 125.87, 125.84, 125.66, 121.75, 121.16, 34.50, 30.99.

HRMS (ESI-TOF) *m/z*: $[M + H]^+$ calculated for $C_{22}H_{22}N_3O_2S$ 392.1427, Found, 392.1428.

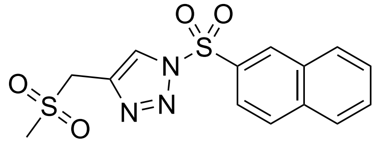

HSU058_AHL-032
Chemical Formula: $C_{14}H_{13}N_3O_4S_2$
Exact Mass: 351.0347

^{1}H NMR (400 MHz, DMSO-D6) δ 9.03 (d, *J* = 2.1 Hz, 1H), 8.96 (s, 1H), 8.33 (d, *J* = 8.0 Hz, 1H), 8.27 (d, *J* = 8.8 Hz, 1H), 8.13 (d, *J* = 7.9 Hz, 1H), 8.06 (dd, *J* = 8.8, 2.1 Hz, 1H), 7.84 (ddd, *J* = 8.3, 6.9, 1.4 Hz, 1H), 7.78 (ddd, *J* = 8.2, 6.9, 1.4 Hz, 1H), 4.68 (s, 2H), 3.03 (s, 3H).

^{13}C NMR (100 MHz, DMSO-D6) δ 137.10, 135.69, 131.76, 131.55, 131.36, 131.00, 130.82, 130.05, 128.59, 128.16, 125.81, 121.82, 50.38.

HRMS (ESI-TOF) *m/z*: $[M + H]^+$ calculated for $C_{14}H_{14}N_3O_4S_2$ 352.0420, Found, 352.0419.

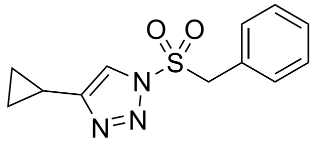

HSU059_AHL-033
Chemical Formula: $C_{12}H_{13}N_3O_2S$
Exact Mass: 263.0728

^{1}H NMR (400 MHz, DMSO-D6) δ 8.10 (s, 1H), 7.60 – 7.25 (m, 3H), 7.10 (d, *J* = 6.5 Hz, 2H), 5.29 (s, 2H), 1.97 (ddd, *J* = 13.4, 8.4, 5.0 Hz, 1H), 1.13 – 0.85 (m, 3H), 0.84 – 0.55 (m, 3H).

^{13}C NMR (151 MHz, DMSO-D6) δ 149.70, 130.81, 129.39, 128.77, 126.48, 121.68, 59.98, 7.89, 6.15.

HRMS (ESI-TOF) *m/z*: $[M + Na]^+$ calculated for $C_{12}H_{13}N_3O_2SNa$ 286.0621, Found, 286.0616.

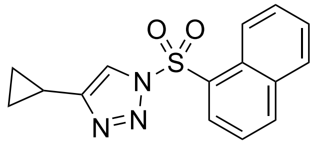

HSU060_AHL-035
Chemical Formula: $C_{15}H_{13}N_3O_2S$
Exact Mass: 299.0728

^{1}H NMR (400 MHz, DMSO-D6) δ 8.99 – 8.73 (m, 1H), 7.94 (d, *J* = 7.0 Hz, 1H), 7.91 – 7.83 (m, 2H), 7.60 (s, 1H), 7.53 – 7.47 (m, 2H), 7.43 (t, *J* = 7.7 Hz, 1H), 1.96 (ddd, *J* = 13.3, 8.1, 4.8 Hz, 1H), 0.99 – 0.89 (m, 2H), 0.75 – 0.67 (m, 2H).

^{13}C NMR (151 MHz, DMSO-D6) δ 143.93, 133.54, 129.42, 129.03, 127.75, 127.62, 125.58, 125.56, 124.46, 124.35, 7.95, 5.84.

HRMS (ESI-TOF) *m/z*: $[M + H]^+$ calculated for $C_{15}H_{14}N_3O_2S$ 300.0801, Found, 300.0788.

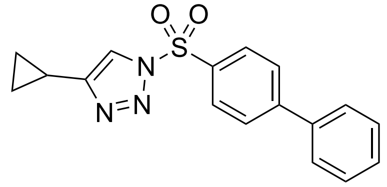

HSU061_AHL-038
Chemical Formula: $C_{17}H_{15}N_3O_2S$
Exact Mass: 325.0885

^{1}H NMR (400 MHz, DMSO-D6) δ 9.17 (s, 1H), 7.69 – 7.65 (m, 3H), 7.63 – 7.58 (m, 3H), 7.47 (t, *J* = 7.7 Hz, 2H), 7.37 (t, *J* = 7.6 Hz, 1H), 1.96 (tt, *J* = 8.5, 5.1 Hz, 1H), 0.97 – 0.89 (m, 2H), 0.75 – 0.65 (m, 2H).

^{13}C NMR (100 MHz, DMSO-D6) δ 147.17, 147.00, 140.29, 139.73, 128.99, 127.63, 127.41, 126.78, 126.19, 126.11, 8.00, 5.84.

HRMS (ESI-TOF) *m/z*: $[M + H]^+$ calculated for $C_{17}H_{16}N_3O_2S$ 326.0958, Found, 326.0946.

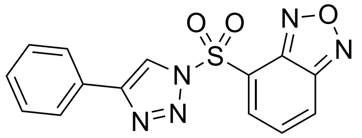

HSU062_AHL7004A
Chemical Formula: $C_{14}H_9N_5O_3S$
Exact Mass: 327.0426

^{1}H NMR (400 MHz, Acetone-D6) δ 9.37 (s, 1H), 8.69 (dd, *J* = 6.9, 0.8 Hz, 1H), 8.51 (dd, *J* = 9.1, 0.8 Hz, 1H), 8.09 – 7.85 (m, 3H), 7.63 – 7.35 (m, 3H).

^{13}C NMR (100 MHz, Acetone-D6) δ 165.86, 150.68, 149.09, 144.74, 139.51, 132.13, 131.58, 129.99, 129.66, 127.67, 125.77, 125.25.

HRMS (ESI-TOF) *m/z*: $[M + H]^+$ calculated for $C_{14}H_{10}N_5O_3S$ 328.0499, Found, 328.0488.

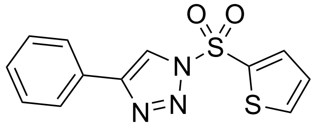

HSU063_AHL7004B
Chemical Formula: $C_{12}H_{10}N_3O_2S_2$
Exact Mass: 291.0136

^{1}H NMR (400 MHz, Acetone-D6) δ 9.16 (s, 1H), 8.25 (d, *J* = 5.0 Hz, 1H), 8.17 – 7.94 (m, 3H), 7.49 (s, 3H), 7.35 (t, *J* = 4.6 Hz, 1H).

^{13}C NMR (100 MHz, Acetone-D6) δ 165.51, 147.26, 139.25, 138.25, 136.02, 131.43, 130.22, 129.66, 129.65, 127.59.

HRMS (ESI-TOF) *m/z*: $[M + H]^+$ calculated for $C_{12}H_{11}N_3O_2S_2$ 292.0209, Found, 292.0196.

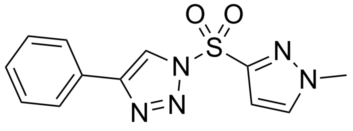

HSU064_AHL7004C
Chemical Formula: $C_{12}H_{11}N_5O_2S$
Exact Mass: 289.0633

^{1}H NMR (400 MHz, Acetone-D6) δ 9.14 (s, 1H), 8.10 (ddt, *J* = 5.5, 2.2, 0.7 Hz, 2H), 7.93 (d, *J* = 2.4 Hz, 1H), 7.49 (dd, *J* = 3.8, 2.8 Hz, 3H), 7.07 (d, *J* = 2.5 Hz, 1H). 4.01 (s, 3H).

^{13}C NMR (100 MHz, Acetone-D6) δ 165.41, 147.80, 146.46, 134.77, 131.38, 130.34, 129.67, 127.59, 110.22, 40.55.

HRMS (ESI-TOF) *m/z*: $[M + H]^+$ calculated for $C_{12}H_{12}N_5O_2S$ 290.0706, Found, 290.0695

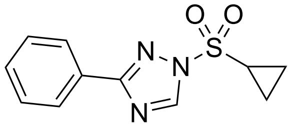

HSU071_JWB101
Chemical Formula: $C_{11}H_{11}N_3O_2S$
Exact Mass: 249.0572

^{1}H NMR (800 MHz, DMSO-D6) δ 9.27 (s, 1H), 8.32 – 7.96 (m, 2H), 7.72 – 7.31 (m, 3H), 3.37 (tt, *J* = 7.8, 4.5 Hz, 1H), 1.44 – 1.41 (m, 2H), 1.39 – 1.22 (m, 2H).

^{13}C NMR (201 MHz, DMSO-D6) δ 163.49, 147.48, 130.64, 129.07, 128.99, 126.53, 30.99, 7.25.

HRMS (ESI-TOF) *m/z*: $[M + H]^+$ calculated for $C_{11}H_{12}N_3O_2S$ 250.0645, Found, 250.0641.

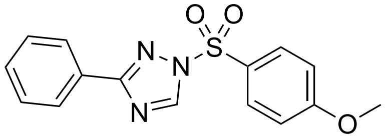

HSU072_JWB106
Chemical Formula: $C_{15}H_{13}N_3O_3S$
Exact Mass: 315.0678

^{1}H NMR (800 MHz, DMSO-D6) δ 9.42 (s, 1H), 8.10 – 8.07 (m, 2H), 8.00 – 7.96 (m, 2H), 7.49 (dd, *J* = 5.1, 2.0 Hz, 3H), 7.26 – 7.20 (m, 2H), 3.86 (s, 3H).

^{13}C NMR (201 MHz, DMSO-D6) δ 164.98, 163.79, 147.34, 130.95, 130.68, 129.02, 128.77, 126.47, 126.05, 115.54, 56.13.

HRMS (ESI-TOF) *m/z*: $[M + H]^+$ calculated for $C_{15}H_{14}N_3O_3S$ 316.0750, Found, 316.0748.

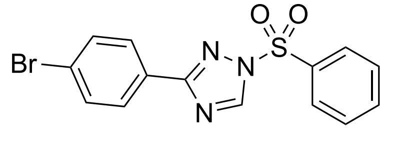

HSU073_JWB117
Chemical Formula: $C_{14}H_{10}BrN_3O_2S$
Exact Mass: 362.9677

^{1}H NMR (600 MHz, DMSO-D6) δ 9.51 (s, 1H), 8.15 (d, *J* = 7.5 Hz, 2H), 7.92 (d, *J* = 8.4 Hz, 2H), 7.88 (t, *J* = 7.5 Hz, 1H), 7.75 (t, *J* = 7.8 Hz, 2H), 7.70 (d, *J* = 8.4 Hz, 2H).

^{13}C NMR (201 MHz, DMSO-D6) δ 163.38, 152.71, 148.19, 132.23, 131.92, 128.52, 128.48, 128.00, 127.98, 127.67, 125.48.

HRMS (ESI-TOF) *m/z*: $[M + H]^+$ calculated for $C_{14}H_{11}BrN_3O_2S$ 363.9750, Found, 363.9744.

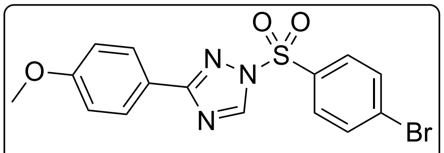

HSU074_JWB134
Chemical Formula: $C_{15}H_{12}BrN_3O_3S$
Exact Mass: 392.9783

^{1}H NMR (600 MHz, DMSO-D6) δ 9.42 (s, 1H), 8.06 (d, *J* = 8.6 Hz, 2H), 7.94 (dd, *J* = 17.3, 8.7 Hz, 4H), 7.04 (d, *J* = 8.7 Hz, 2H), 3.80 (s, 3H).

^{13}C NMR (201 MHz, DMSO-D6) δ 164.22, 161.33, 152.35, 147.60, 130.67, 128.24, 127.76, 121.66, 121.23, 114.62, 55.45.

HRMS (ESI-TOF) *m/z*: $[M + H]^+$ calculated for $C_{15}H_{13}BrN_3O_3S$ 393.9856, Found, 393.9849.

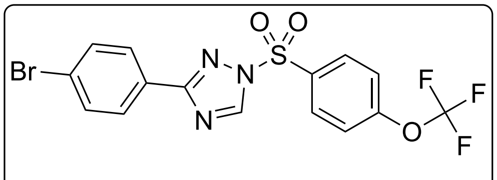

HSU076_JWB124
Chemical Formula: $C_{15}H_9BrF_3N_3O_3S$
Exact Mass: 446.9500

^{1}H NMR (800 MHz, DMSO-D6) δ 8.63 (s, 1H), 8.04 – 7.89 (m, 2H), 7.80 – 7.60 (m, 4H), 7.43 – 7.16 (m, 2H).

^{13}C NMR (201 MHz, DMSO-D6) δ 148.61, 148.09, 146.67, 132.37, 129.43, 128.41, 128.11, 123.24, 121.14, 120.73, 119.87.

^{19}F NMR (376 MHz, Acetone-D6) δ -58.50 (s, 3F).

HRMS (ESI-TOF) *m/z*: $[M + H]^+$ calculated for $C_{15}H_{10}BrF_3N_3O_3S$ 447.9573, Found, 447.9562.

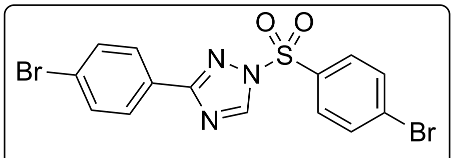

HSU077_JWB119
Chemical Formula: $C_{14}H_9Br_2N_3O_2S$
Exact Mass: 440.8782

^{1}H NMR (800 MHz, DMSO-D6) δ 8.62 (s, 1H), 8.06 – 7.84 (m, 2H), 7.76 – 7.65 (m, 2H), 7.61 – 7.44 (m, 4H).

^{13}C NMR (201 MHz, DMSO-D6) δ 157.69, 147.68, 146.17, 131.90, 130.62, 128.95, 127.95, 127.78, 122.77, 121.60.

HRMS (ESI-TOF) *m/z*: $[M + H]^+$ calculated for $C_{14}H_{10}Br_2N_3O_2S$ 441.8855, Found, 441.8853.

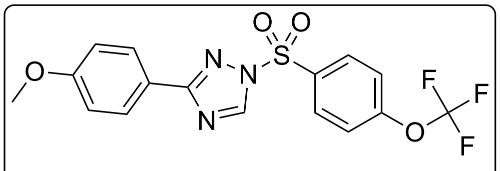

HSU078_JWB139
Chemical Formula: $C_{16}H_{12}F_3N_3O_4S$
Exact Mass: 399.0501

^{1}H NMR (800 MHz, DMSO-D6) δ 9.41 (s, 1H), 8.57 – 8.09 (m, 2H), 8.04 – 7.84 (m, 2H), 7.71 (dd, *J* = 9.1, 1.1 Hz, 2H), 7.25 – 6.76 (m, 2H), 3.81 (s, 3H).

^{13}C NMR (201 MHz, DMSO-D6) δ 164.08, 161.21, 153.06, 147.66, 133.93, 131.14, 128.17, 121.92, 120.92, 114.37, 55.26.

^{19}F NMR (376 MHz, Acetone-D6) δ -58.50 (s, 3F).

HRMS (ESI-TOF) *m/z*: $[M + H]^+$ calculated for $C_{16}H_{11}F_3N_3O_4S$ 400.0573, Found, 400.0568.

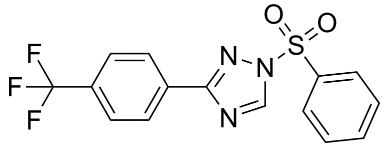

HSU079_JWB147
Chemical Formula: $C_{15}H_{10}F_3N_3O_2S$
Exact Mass: 353.0446

^{1}H NMR (600 MHz, DMSO-D6) δ 9.57 (s, 1H), 8.18 (t, *J* = 9.3 Hz, 4H), 7.87 (m, 3H), 7.75 (t, *J* = 7.9 Hz, 2H).

^{13}C NMR (201 MHz, DMSO-D6) δ 162.92, 158.08, 152.90, 148.18, 146.10, 128.49, 127.68, 127.35, 126.56, 126.20, 125.86 (q, *J* = 3.7 Hz), 125.49.

^{19}F NMR (376 MHz, acetone) δ -63.41 (s, 3F).

HRMS (ESI-TOF) *m/z*: $[M + H]^+$ calculated for $C_{15}H_{11}F_3N_3O_2S$ 354.0519, Found, 354.0519.

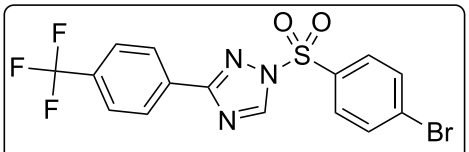

HSU080_JWB149
Chemical Formula: $C_{15}H_9BrF_3N_3O_2S$
Exact Mass: 430.9551

^{1}H NMR (800 MHz, DMSO-D6) δ 8.67 (s, 1H), 8.23 (d, *J* = 8.0 Hz, 2H), 7.86 (d, *J* = 7.9 Hz, 2H), 7.61 – 7.36 (m, 4H).

^{13}C NMR (201 MHz, DMSO-D6) δ 158.12, 147.65, 146.10, 134.08, 130.64, 129.30 (q, *J* = 31.6 Hz), 127.76, 126.55, 125.86 (q, *J* = 3.9 Hz), 124.18 (d, *J* = 272.4 Hz), 121.63.

^{19}F NMR (376 MHz, Acetone-D6) δ -63.41 (s, 3F).

HRMS (ESI-TOF) *m/z*: $[M + H]^+$ calculated for $C_{15}H_{10}BrF_3N_3O_2S$ 431.9624, Found, 431.9614.

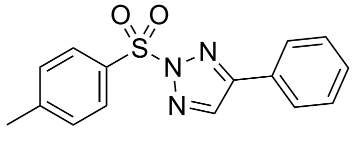

HSU086_AHL-1101
Chemical Formula: $C_{15}H_{13}N_3O_2S$
Exact Mass: 299.0728

^{1}H NMR (400 MHz, Aceton-D6) δ 8.53 (d, *J* = 1.0 Hz, 1H), 7.99 (d, *J* = 8.5 Hz, 2H), 7.94 (dd, *J* = 6.9, 1.4 Hz, 2H), 7.55 – 7.39 (m, 5H), 2.42 (s, 3H).

^{13}C NMR (100 MHz, Acetone-D6) δ 152.51, 148.04, 137.40, 133.97, 131.30, 130.89, 129.98, 129.32, 127.48, 21.62.

HRMS (ESI-TOF) *m/z*: $[M + H]^+$ calculated for $C_{15}H_{14}N_3O_2S$ 300.0801, Found, 300.0793.

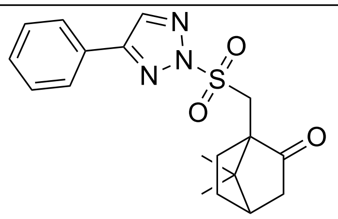

HSU087_AHL1118
Chemical Formula: $C_{18}H_{21}N_3O_3S$
Exact Mass: 359.1304

^{1}H NMR (400 MHz, Acetone-D6) δ 8.59 (s, 1H), 8.00 (d, *J* = 6.5 Hz, 2H), 7.60 – 7.45 (m, 3H), 4.03 (d, *J* = 15.2 Hz, 1H), 3.72 (d, *J* = 15.2 Hz, 1H), 2.43 – 2.25 (m, 2H), 2.16 (t, *J* = 4.5 Hz, 1H), 2.13 – 2.07 (m, 1H), 1.94 (d, *J* = 18.4 Hz, 1H), 1.76 (ddd, *J* = 13.8, 9.4, 4.6 Hz, 1H), 1.52 (ddd, *J* = 12.9, 9.3, 3.8 Hz, 1H), 1.12 (s, 3H), 0.91 (s, 3H).

^{13}C NMR (100 MHz, Aceton-D6) δ 213.17, 151.90, 136.63, 130.80, 130.01, 129.60, 127.51, 59.19, 52.69, 49.06, 43.48, 42.69, 27.37, 25.76, 19.72, 19.70.

HRMS (ESI-TOF) *m/z*: $[M + H]^+$ calculated for $C_{18}H_{22}N_3O_3S$ 360.1376, Found, 360.1363.

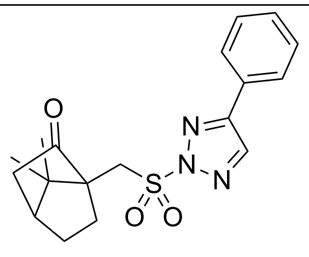

HSU088_AHL1119
Chemical Formula: $C_{18}H_{21}N_3O_3S$
Exact Mass: 359.1304

^{1}H NMR (400 MHz, $CDCl_3$) δ 8.18 (s, 1H), 7.90 (dd, *J* = 8.0, 1.7 Hz, 2H), 7.53 – 7.42 (m, 3H), 3.99 (d, *J* = 15.0 Hz, 1H), 3.43 (d, *J* = 14.9 Hz, 1H), 2.49 (ddd, *J* = 14.9, 11.7, 4.0 Hz, 1H), 2.39 (dt, *J* = 18.6, 4.0 Hz, 1H), 2.19 – 2.03 (m, 2H), 1.94 (d, *J* = 18.6 Hz, 1H), 1.80 (ddd, *J* = 13.9, 9.3, 4.5 Hz, 1H), 1.47 (ddd, *J* = 13.1, 9.2, 3.8 Hz, 1H), 1.18 (s, 3H), 0.91 (s, 3H).

^{13}C NMR (151 MHz, DMSO-D6) δ 213.02, 150.63, 136.47, 130.10, 129.25, 128.11, 126.60, 58.14, 51.78, 48.35, 42.06, 41.79, 26.24, 24.72, 19.09, 18.95.

HRMS (ESI-TOF) *m/z*: $[M + H]^+$ calculated for $C_{18}H_{22}N_3O_3S$ 360.1376, Found, 360.1363.

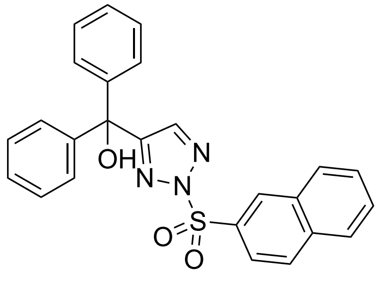

HSU089_AHL1122

Chemical Formula: $C_{25}H_{19}N_3O_3S$

Exact Mass: 441.1147

^{1}H NMR (400 MHz, DMSO-D6) δ 8.80 (d, *J* = 2.5 Hz, 1H), 8.31 (d, *J* = 7.4 Hz, 1H), 8.21 (d, *J* = 9.0 Hz, 1H), 8.17 (s, 1H), 8.11 (d, *J* = 8.1 Hz, 1H), 7.88 – 7.79 (m, 2H), 7.76 (ddd, *J* = 8.2, 6.9, 1.4 Hz, 1H), 7.23 – 7.11 (m, 10H), 6.97 (s, 1H).

^{13}C NMR (100 MHz, DMSO-D6) δ 160.07, 145.11, 139.77, 135.39, 131.81, 131.53, 130.67, 130.57, 130.29, 129.95, 128.39, 128.08, 127.80, 127.28, 126.74, 121.78, 75.93.

HRMS (ESI-TOF) *m/z*: $[M + H]^+$ calculated for $C_{25}H_{20}N_3O_3S$ 442.1220, Found, 442.1204.

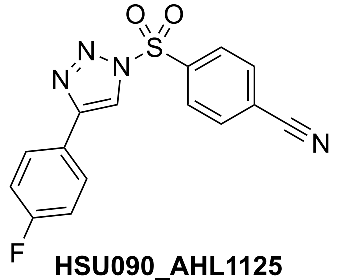

HSU090_AHL1125

Chemical Formula: $C_{15}H_9FN_4O_2S$

Exact Mass: 328.0430

^{1}H NMR (400 MHz, Acetone-D6) δ 8.63 (s, 1H), 8.30 (d, *J* = 8.9 Hz, 2H), 8.14 (d, *J* = 8.8 Hz, 2H), 8.02 (dd, *J* = 8.8, 5.3 Hz, 2H), 7.29 (t, *J* = 8.8 Hz, 2H).

^{13}C NMR (100 MHz, Aceton-D6) δ 164.74 (d, *J* = 248.7 Hz), 163.50, 152.52, 140.57, 138.43, 134.77, 130.01, 130.00 (d, *J* = 8.7 Hz), 125.50 (d, *J* = 3.2 Hz), 125.48, 119.60, 117.60, 117.03 (d, *J* = 22.2 Hz).

^{19}F NMR (376 MHz, Aceton-D6) δ -111.64 – -111.76 (m, 1F).

HRMS (ESI-TOF) *m/z*: $[M + H]^+$ calculated for $C_{15}H_{10}FN_4O_2S$ 329.0503, Found, 329.0499.

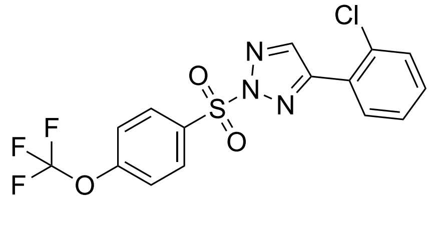

HSU091_AHL1126
Chemical Formula: $C_{15}H_9ClF_3N_3O_3S$
Exact Mass: 403.0005

^{1}H NMR (400 MHz, $CDCl_3$) δ 8.38 (s, 1H), 8.20 (d, *J* = 9.1 Hz, 2H), 7.98 – 7.81 (m, 1H), 7.57 – 7.43 (m, 1H), 7.43 – 7.32 (m, 4H).

^{13}C NMR (151 MHz, DMSO-D6) δ 153.24, 149.41, 139.95, 133.25, 131.79, 131.73, 131.34, 131.24, 131.21, 130.45, 127.84, 126.65, 122.02.

^{19}F NMR (376 MHz, Acetone-D6) δ -58.51 (s, 3F).

HRMS (ESI-TOF) *m/z*: $[M + H]^+$ calculated for $C_{15}H_{10}ClF_3N_3O_3S$ 404.0078, Found, 404.0076.

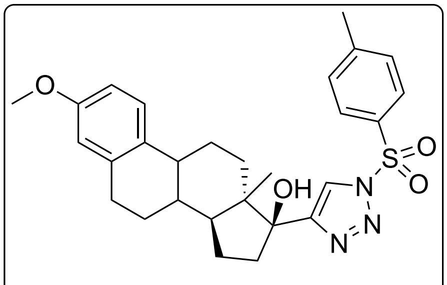

HSU092_AHL2065
Chemical Formula: $C_{28}H_{33}N_3O_4S$
Exact Mass: 507.2192

^{1}H NMR (400 MHz, DMSO-D6) δ 7.92 (d, *J* = 8.6 Hz, 2H), 7.72 (s, 1H), 7.50 (d, *J* = 9.4 Hz, 2H), 7.10 (d, *J* = 8.2 Hz, 1H), 6.65 (dd, *J* = 8.6, 2.9 Hz, 1H), 6.58 (d, *J* = 2.9 Hz, 1H), 5.51 (s, 1H), 3.67 (s, 3H), 3.38 (d, *J* = 7.0 Hz, 1H), 2.76 (d, *J* = 5.4 Hz, 2H), 2.43 (s, 3H), 2.29 – 2.05 (m, 4H), 1.96 – 1.77 (m, 3H), 1.52 – 1.18 (m, 5H), 0.98 (s, 3H), 0.59 – 0.49 (m, 1H).

^{13}C NMR (100 MHz, DMSO-D6) δ 157.05, 146.39, 146.33, 137.33, 134.38, 133.80, 131.93, 130.07, 129.07, 126.16, 113.37, 111.48, 82.06, 54.86, 48.77, 47.89, 42.81, 40.73, 33.62, 29.25, 26.90, 26.14, 22.47, 21.25, 13.86.

HRMS (ESI-TOF) *m/z*: $[M + H]^+$ calculated for $C_{28}H_{34}N_3O_4S$ 508.2265, Found, 508.2258.

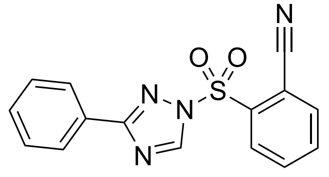

HSU093_JWB230

Chemical Formula: $C_{15}H_{10}N_4O_2S$

Exact Mass: 310.0524

^{1}H NMR (800 MHz, DMSO-D6) δ 8.79 (s, 1H), 8.07 – 7.98 (m, 2H), 7.87 (dd, *J* = 7.8, 1.3 Hz, 2H), 7.76 (dd, *J* = 7.6, 1.3 Hz, 2H), 7.64 (td, *J* = 7.7, 1.3 Hz, 2H), 7.56 – 7.52 (m, 2H), 7.52 – 7.47 (m, 2H).

^{13}C NMR (201 MHz, DMSO-D6) δ 156.64, 150.35, 145.86, 133.95, 132.27, 130.10, 129.04, 128.93, 127.05, 126.55, 126.17, 117.67, 108.95.

HRMS (ESI-TOF) *m/z*: $[M + H]^+$ calculated for $C_{15}H_{11}N_4O_2S$ 311.0597, Found, 311.0596.

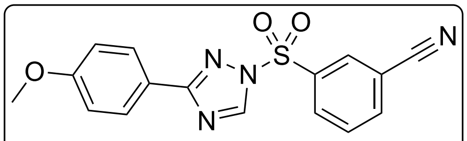

HSU094_JWB234

Chemical Formula: $C_{16}H_{12}N_4O_3S$

Exact Mass: 340.0630

^{1}H NMR (800 MHz, $CDCl_3$) δ 8.72 (s, 1H), 8.42 (t, *J* = 1.7 Hz, 1H), 8.34 (ddd, *J* = 8.1, 1.9, 1.1 Hz, 1H), 8.03 (d, *J* = 8.9 Hz, 2H), 7.98 (dt, *J* = 7.7, 1.3 Hz, 1H), 7.76 (t, *J* = 7.9 Hz, 2H), 6.95 (d, *J* = 8.8 Hz, 2H), 3.85 (s, 3H).

^{13}C NMR (201 MHz, $CDCl_3$) δ 165.85, 161.89, 145.65, 138.30, 138.08, 132.48, 132.26, 130.93, 128.92, 121.38, 116.49, 114.69, 114.27, 55.55.

HRMS (ESI-TOF) *m/z*: $[M + H]^+$ calculated for $C_{16}H_{12}N_4O_3S$ 341.0703, Found, 341.0694.

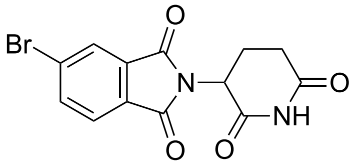

2

Chemical Formula: $C_{13}H_9BrN_2O_4$
Exact Mass: 335.9746

^{1}H NMR (600 MHz, DMSO-D6) δ 11.14 (s, 1H), 8.15 (d, *J* = 1.8 Hz, 1H), 8.10 (dd, *J* = 7.9, 1.7 Hz, 1H), 7.87 (d, *J* = 7.9 Hz, 1H), 5.16 (dd, *J* = 13.0, 5.4 Hz, 1H), 2.89 (ddd, *J* = 17.1, 13.9, 5.4 Hz, 1H), 2.61 (dd, *J* = 17.4, 4.0 Hz, 1H), 2.56 – 2.48 (m, 1H), 2.06 (dtd, *J* = 13.0, 5.4, 2.4 Hz, 1H).

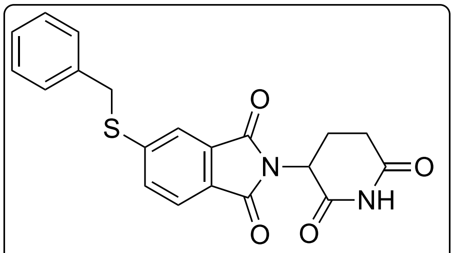

3

Chemical Formula: $C_{20}H_{16}N_2O_4S$
Exact Mass: 380.0831

^{1}H NMR (600 MHz, DMSO-D6) δ 11.09 (s, 1H), 7.93 – 7.57 (m, 3H), 7.53 – 7.36 (m, 2H), 7.31 (t, *J* = 7.6 Hz, 2H), 7.27 – 7.17 (m, 1H), 5.10 (dd, *J* = 12.9, 5.4 Hz, 1H), 4.47 (s, 2H), 2.85 (ddd, *J* = 17.1, 13.9, 5.4 Hz, 1H), 2.57 (d, *J* = 17.3 Hz, 1H), 2.49 (m, 2H), 2.12 – 1.97 (m, 1H).

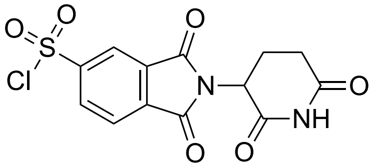

4

Chemical Formula: $C_{13}H_9ClN_2O_6S$
Exact Mass: 355.9870

^{1}H NMR (600 MHz, DMSO-D6) δ 11.12 (s, 1H), 8.07 (dd, *J* = 7.6, 1.3 Hz, 1H), 7.96 (s, 1H), 7.90 (d, *J* = 7.7 Hz, 1H), 5.15 (dd, *J* = 12.9, 5.5 Hz, 1H), 2.89 (ddd, *J* = 17.9, 13.8, 5.5 Hz, 1H), 2.70 – 2.52 (m, 2H), 2.16 – 2.03 (m, 1H).

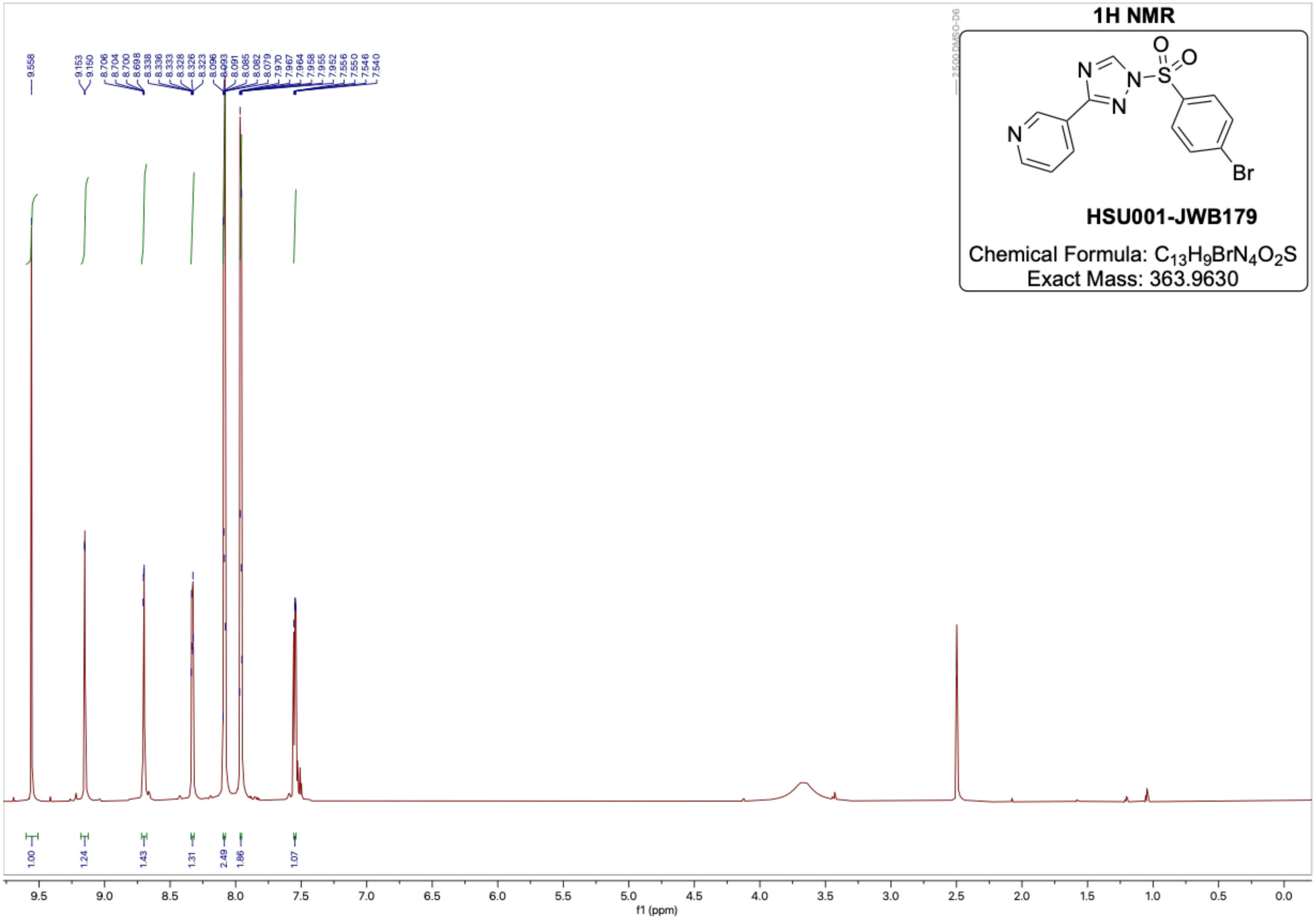
1H NMR
HSU001-JWB179
Chemical Formula: $C_{13}H_9BrN_4O_2S$
Exact Mass: 363.9630
2.500 DMSO-D6
9.558
9.153
9.150
8.706
8.704
8.700
8.698
8.338
8.336
8.333
8.328
8.326
8.323
8.096
8.093
8.091
8.085
8.082
8.079
7.970
7.967
7.964
7.958
7.955
7.952
7.556
7.550
7.546
7.540
1.00
1.24
1.43
1.31
2.49
1.86
1.07
f1 (ppm)

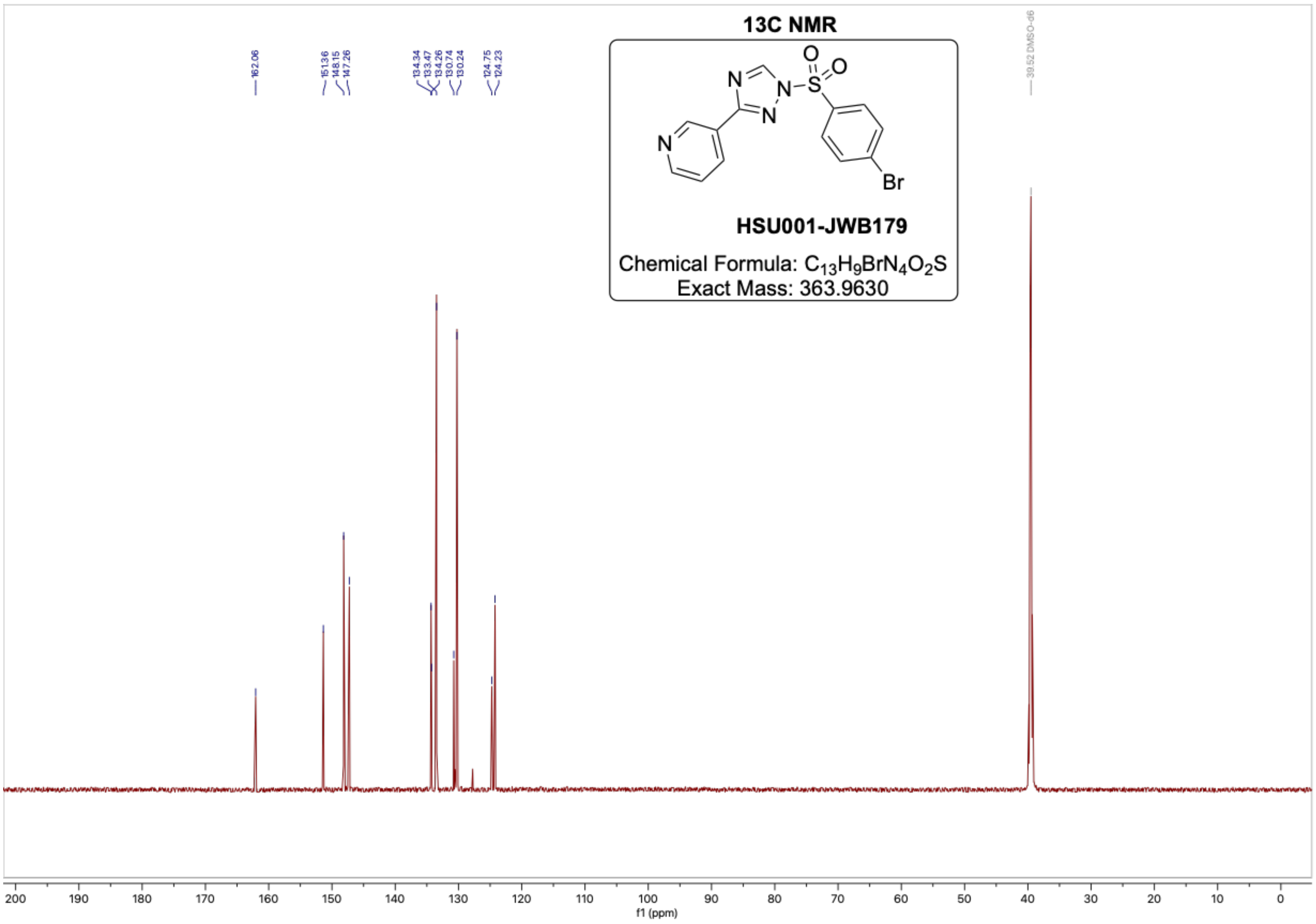

13C NMR
HSU001-JWB179
Chemical Formula: $C_{13}H_9BrN_4O_2S$
Exact Mass: 363.9630
162.06
151.36
148.15
147.26
134.34
133.47
134.26
130.74
130.24
124.75
124.23
39.52 DMSO-d6
f1 (ppm)

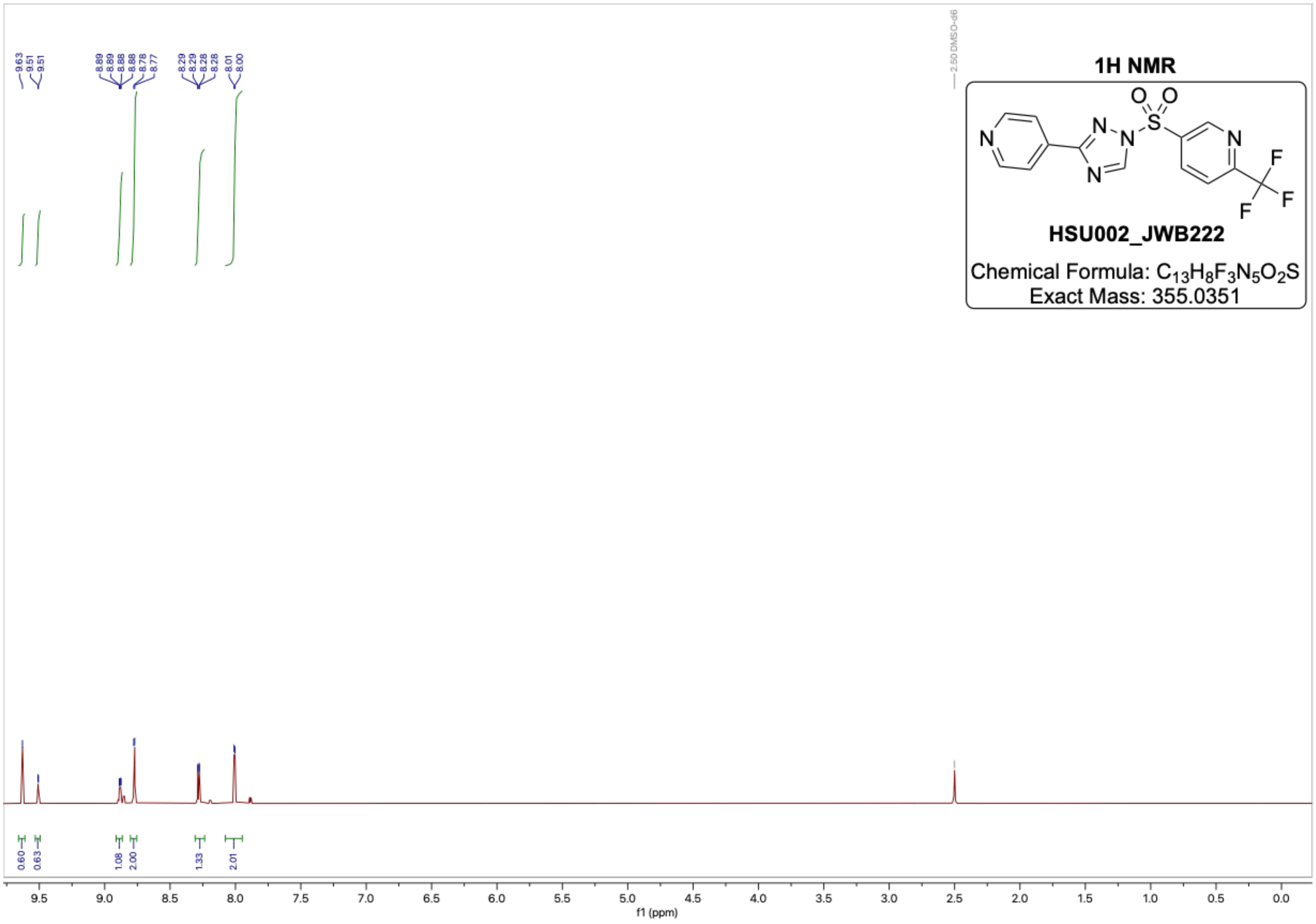

1H NMR
HSU002_JWB222
Chemical Formula: C13H8F3N5O2S
Exact Mass: 355.0351
9.63
9.51
9.51
8.89
8.89
8.88
8.88
8.78
8.77
8.29
8.29
8.28
8.28
8.01
8.00
2.50 DMSO-d6
0.60
0.63
1.08
2.00
1.33
2.01
f1 (ppm)

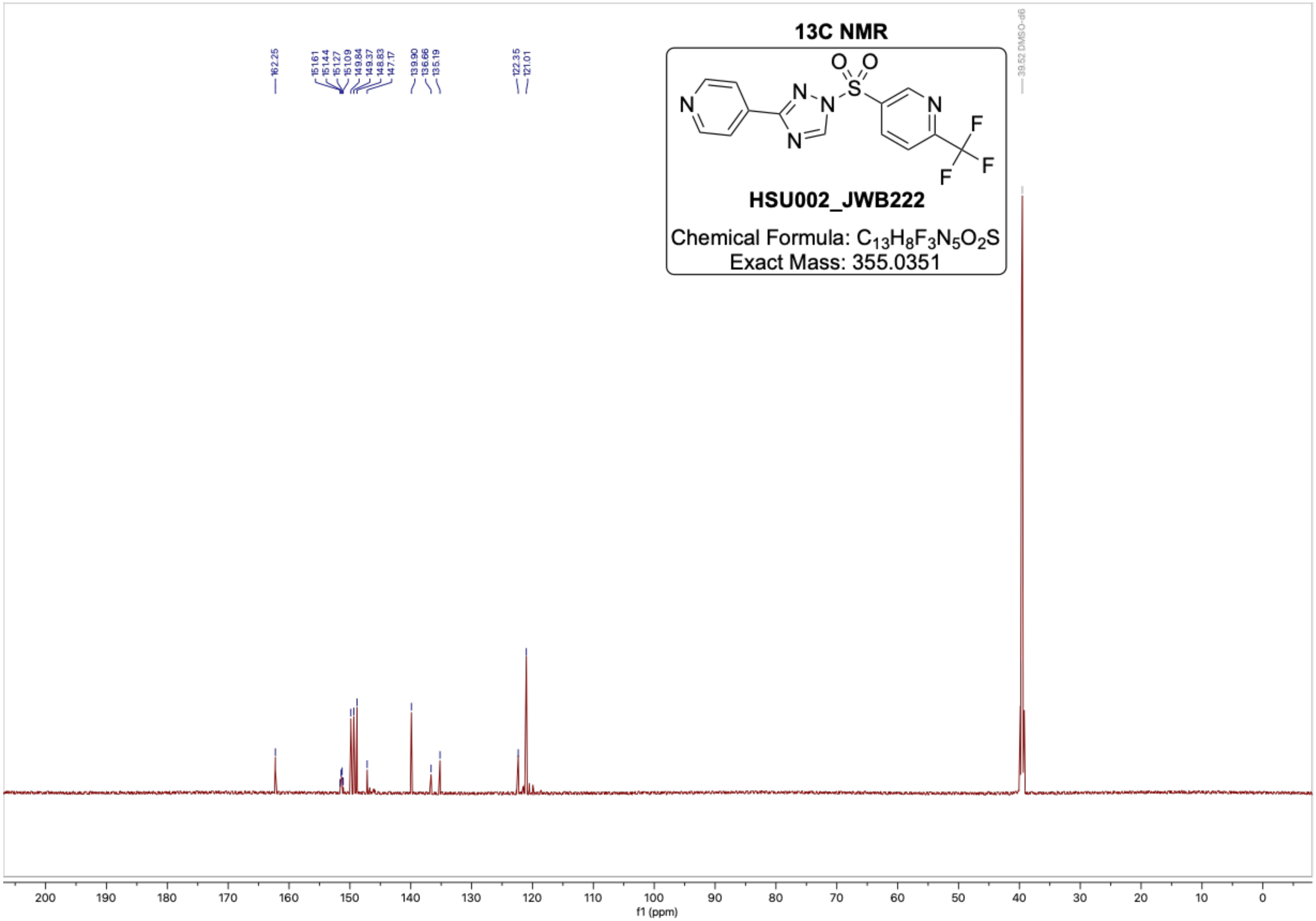

13C NMR
HSU002_JWB222
Chemical Formula: C13H8F3N5O2S
Exact Mass: 355.0351
162.25
151.61
151.44
151.27
151.09
149.84
149.37
148.83
147.17
139.90
136.66
135.19
122.35
121.01
39.52 DMSO-d6
f1 (ppm)

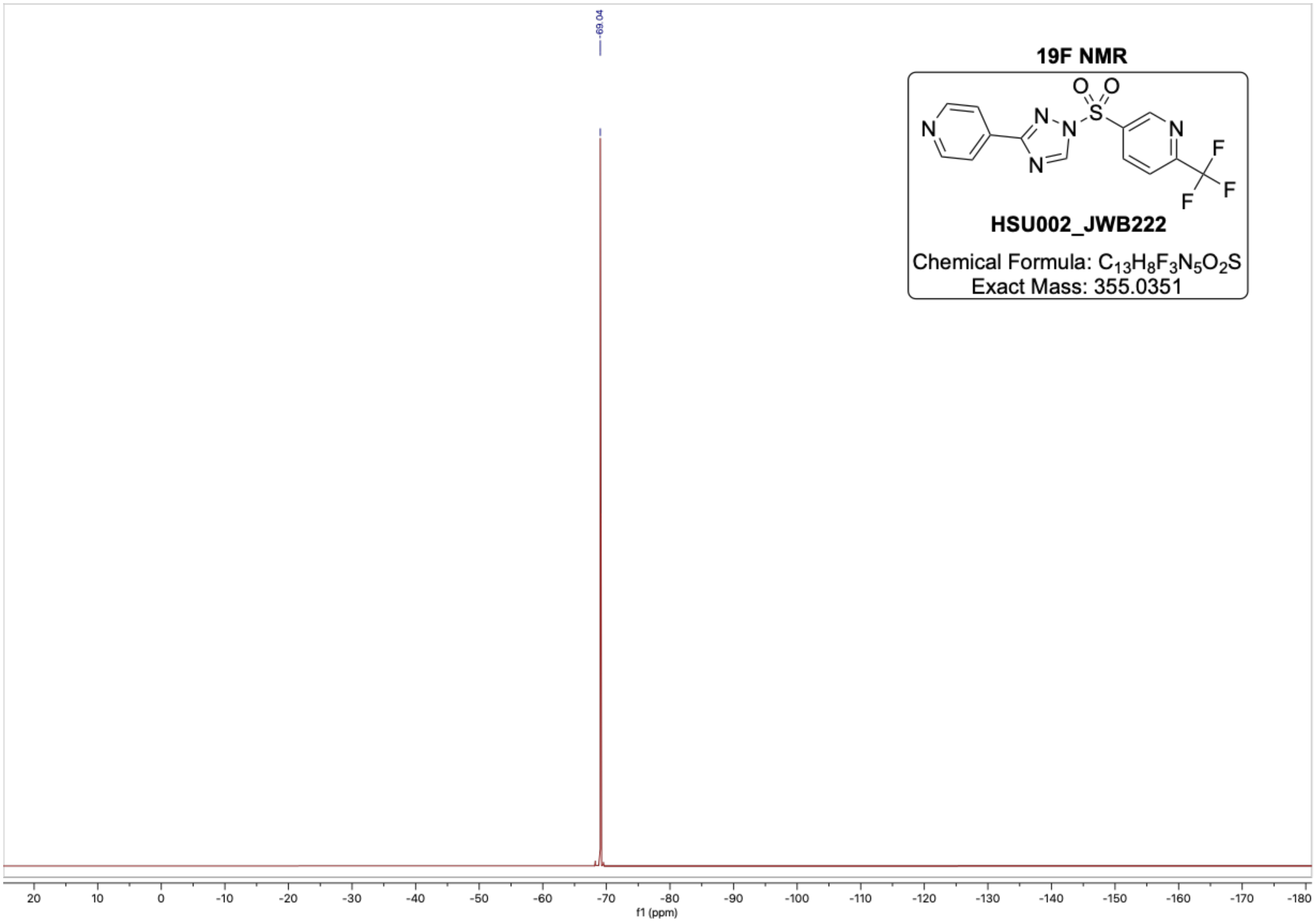

-69.04
19F NMR
O
O
S
N
N
N
N
N
F
F
F
HSU002_JWB222
Chemical Formula: C13H8F3N5O2S
Exact Mass: 355.0351
20
10
0
-10
-20
-30
-40
-50
-60
-70
-80
-90
-100
-110
-120
-130
-140
-150
-160
-170
-180
f1 (ppm)

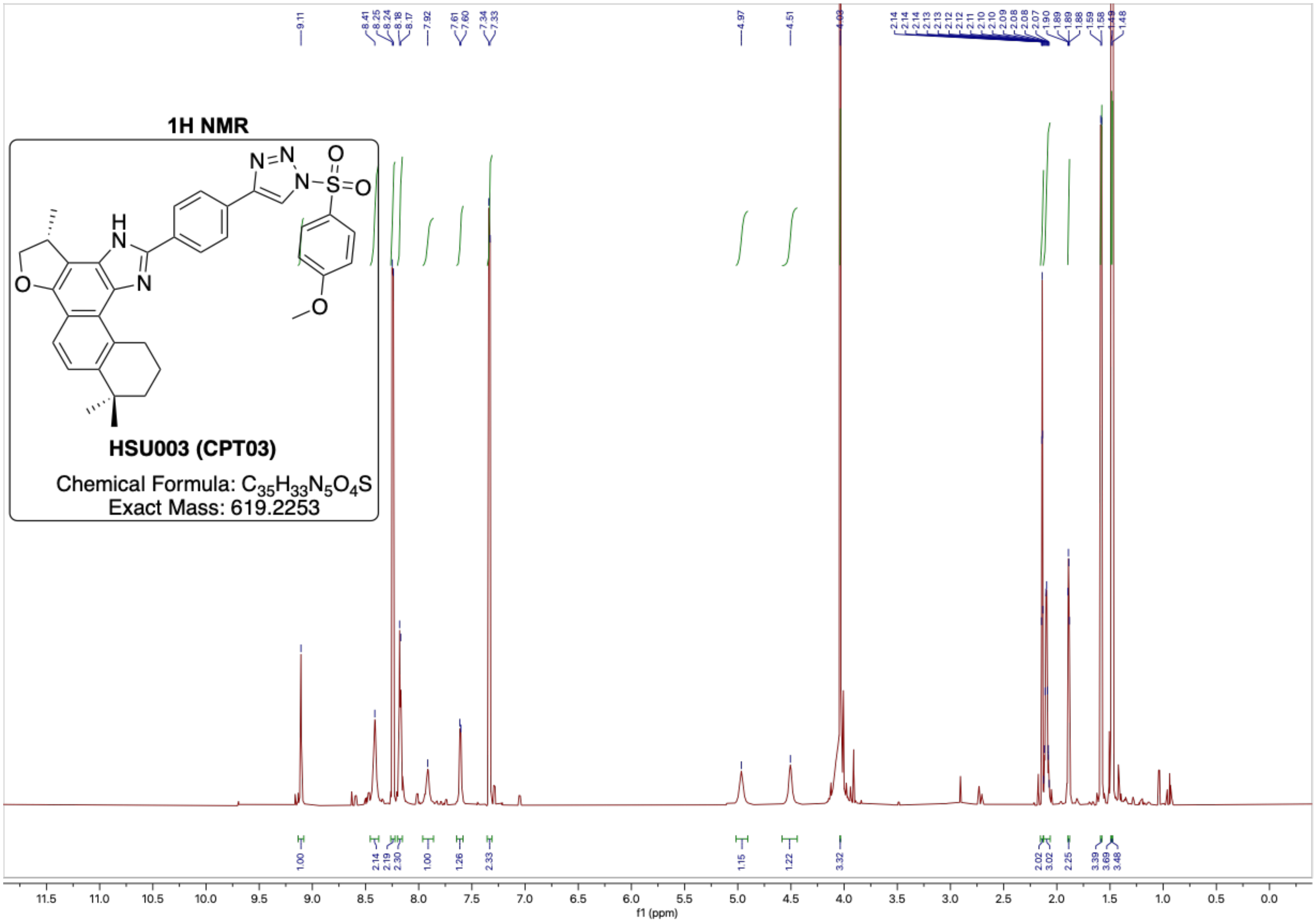
1H NMR
HSU003 (CPT03)
Chemical Formula: $C_{35}H_{33}N_5O_4S$
Exact Mass: 619.2253
f1 (ppm)

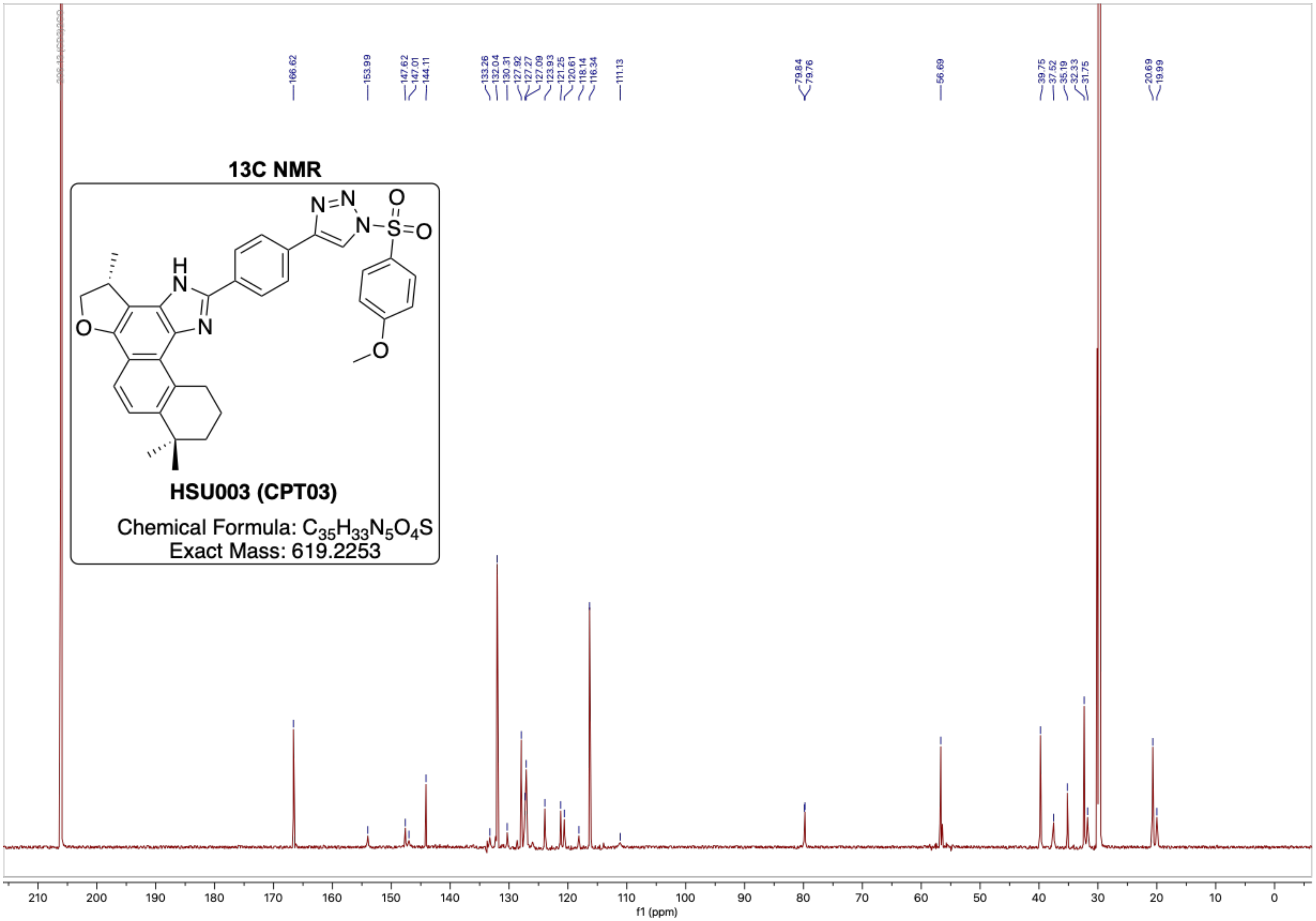
13C NMR
HSU003 (CPT03)
Chemical Formula: $C_{35}H_{33}N_5O_4S$
Exact Mass: 619.2253
166.62
153.99
147.62
147.01
144.11
133.26
132.04
130.31
127.92
127.27
127.09
123.93
121.25
120.61
118.14
116.34
111.13
79.84
79.76
56.69
39.75
37.52
35.19
32.33
31.75
20.69
19.99
f1 (ppm)

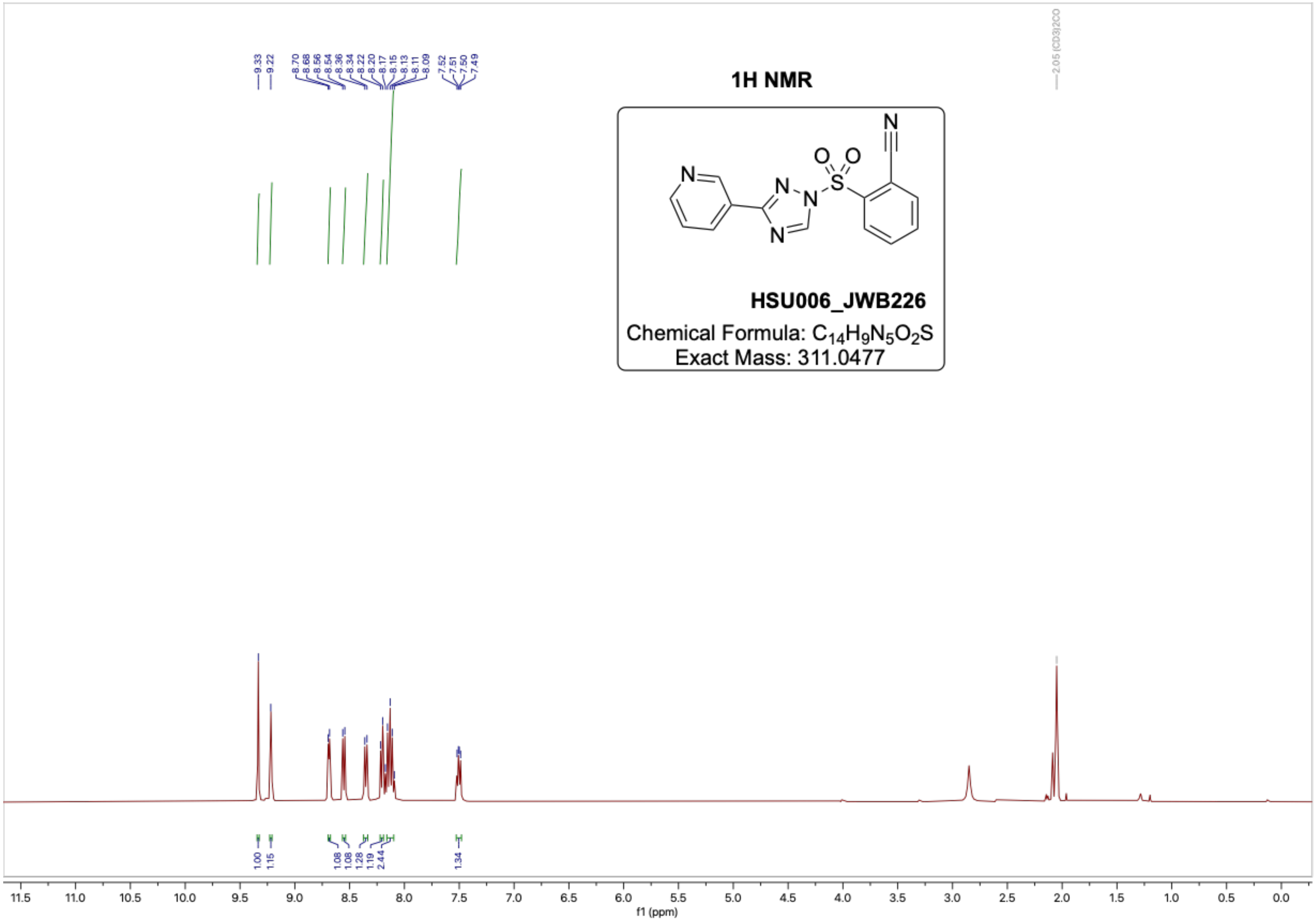
1H NMR
HSU006_JWB226
Chemical Formula: C14H9N5O2S
Exact Mass: 311.0477
9.33
9.22
8.70
8.68
8.56
8.54
8.36
8.34
8.22
8.20
8.17
8.15
8.13
8.11
8.09
7.52
7.51
7.50
7.49
2.05 (CD3)2CO
1.00
1.15
1.08
1.08
1.28
1.19
2.44
1.34
11.5
11.0
10.5
10.0
9.5
9.0
8.5
8.0
7.5
7.0
6.5
6.0
5.5
5.0
4.5
4.0
3.5
3.0
2.5
2.0
1.5
1.0
0.5
0.0
f1 (ppm)

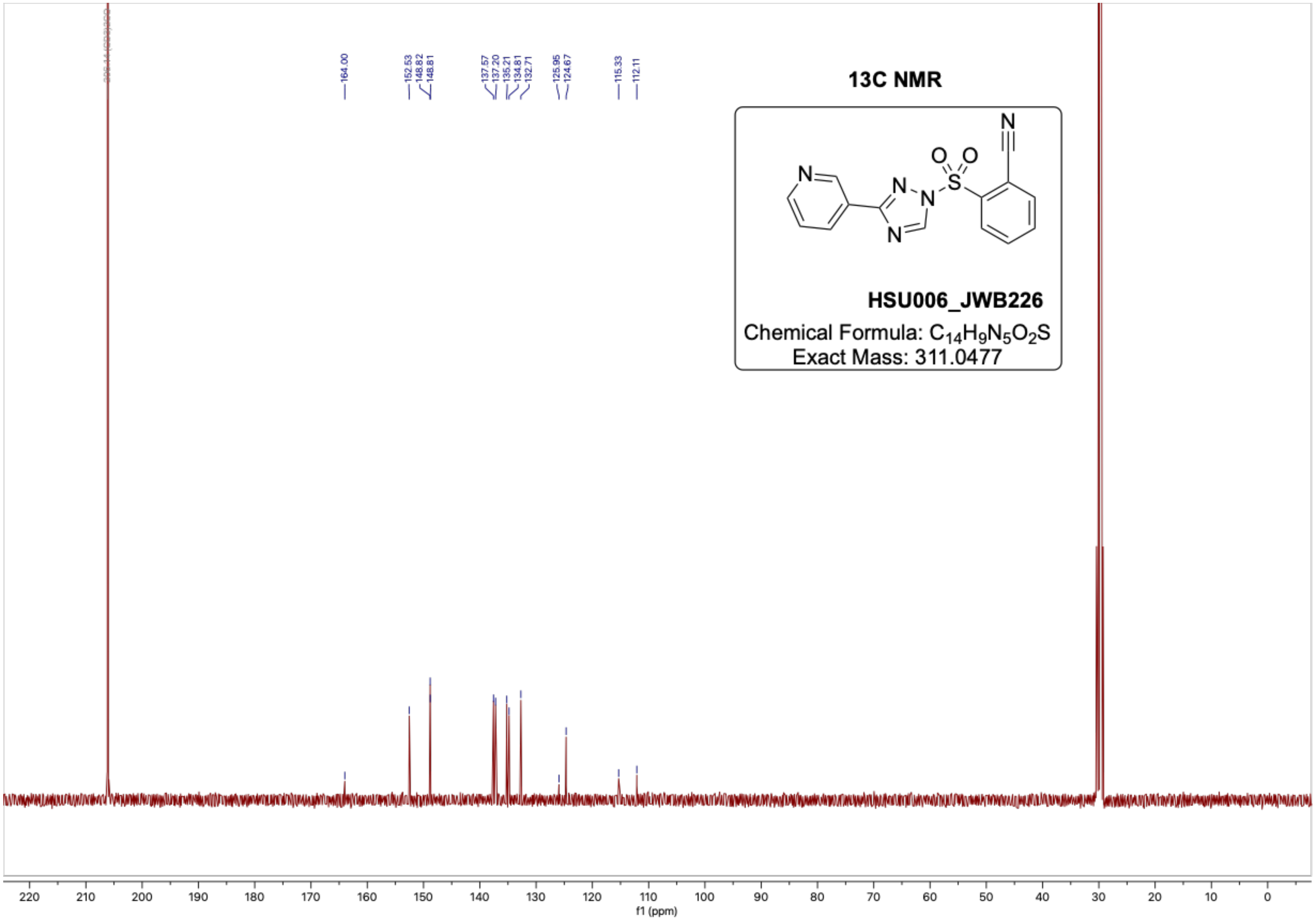
13C NMR
HSU006_JWB226
Chemical Formula: C14H9N5O2S
Exact Mass: 311.0477
164.00
152.53
148.82
148.81
137.57
137.20
135.21
134.81
132.71
125.95
124.67
115.33
112.11
f1 (ppm)

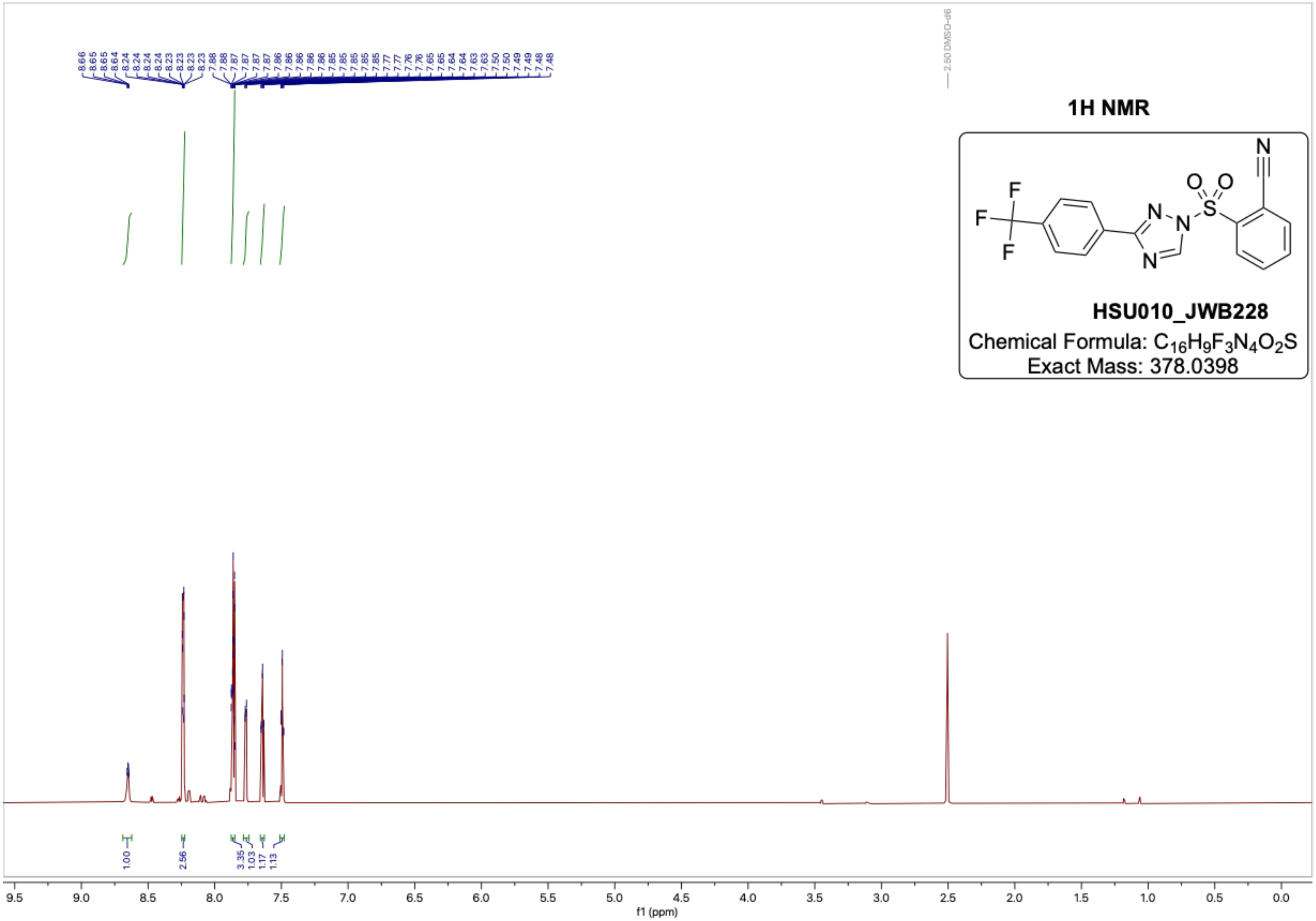
1H NMR
HSU010_JWB228
Chemical Formula: C16H9F3N4O2S
Exact Mass: 378.0398
2.50 DMSO-d6
1.00
2.56
3.35
1.03
1.17
1.13
f1 (ppm)

13C NMR

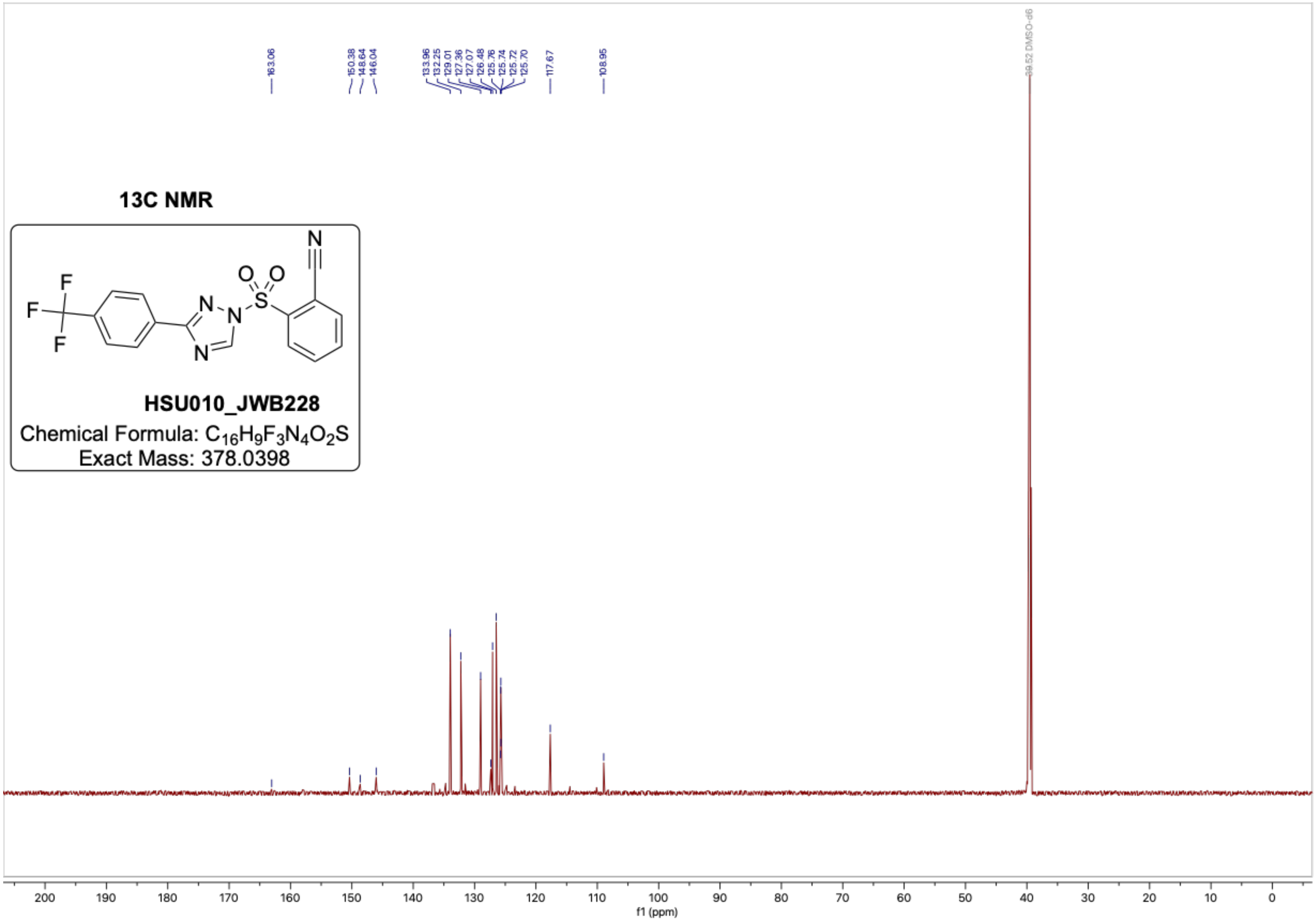
HSU010_JWB228
Chemical Formula: $C_{16}H_9F_3N_4O_2S$
Exact Mass: 378.0398
163.06
150.38
148.64
146.04
133.96
132.25
129.01
127.36
127.07
126.48
125.76
125.74
125.72
125.70
117.67
108.95
39.52 DMSO-d6
200
190
180
170
160
150
140
130
120
110
100
90
80
70
60
50
40
30
20
10
0
f1 (ppm)

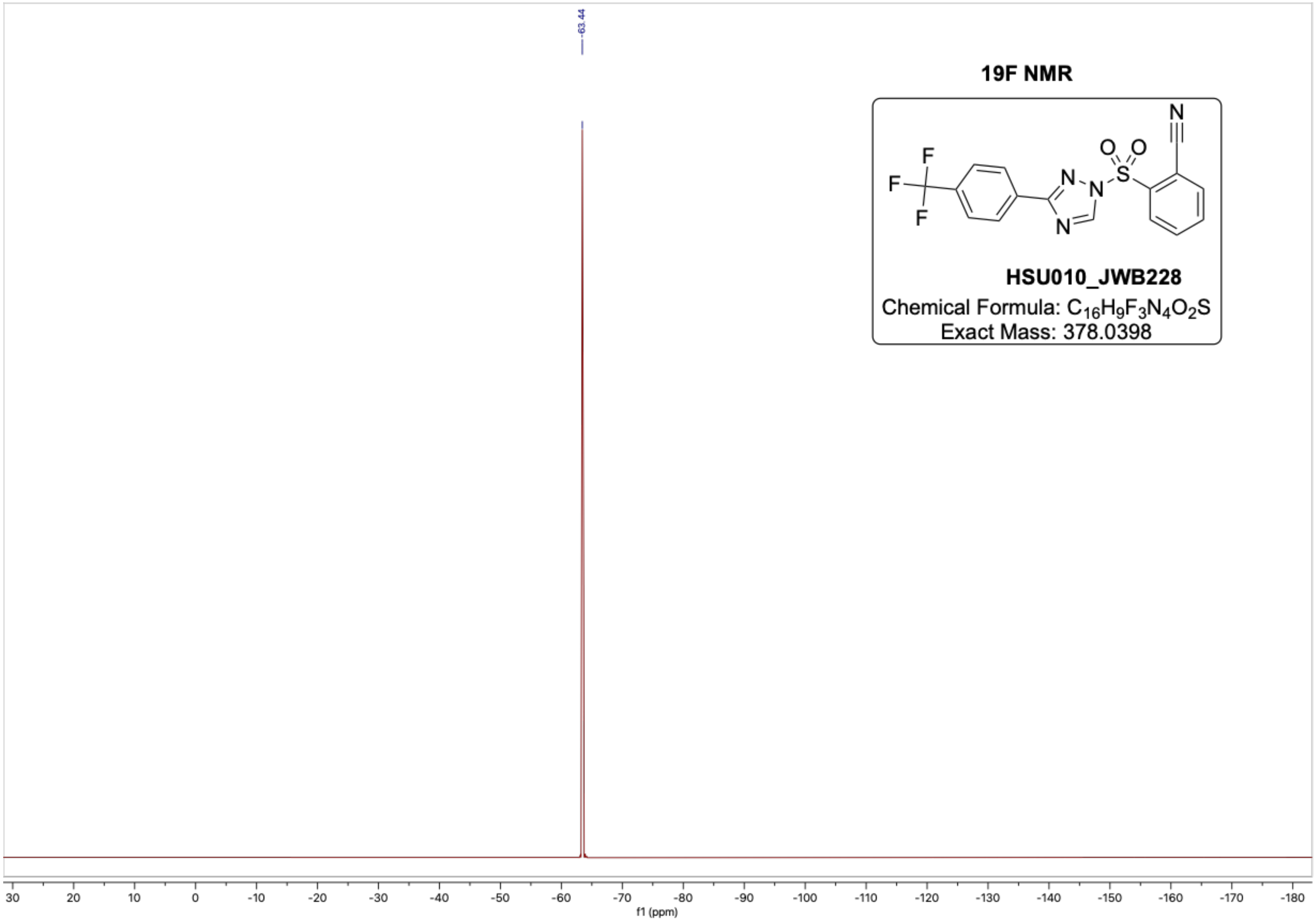
-63.44
19F NMR
HSU010_JWB228
Chemical Formula: $C_{16}H_9F_3N_4O_2S$
Exact Mass: 378.0398
30 20 10 0 -10 -20 -30 -40 -50 -60 -70 -80 -90 -100 -110 -120 -130 -140 -150 -160 -170 -180
f1 (ppm)

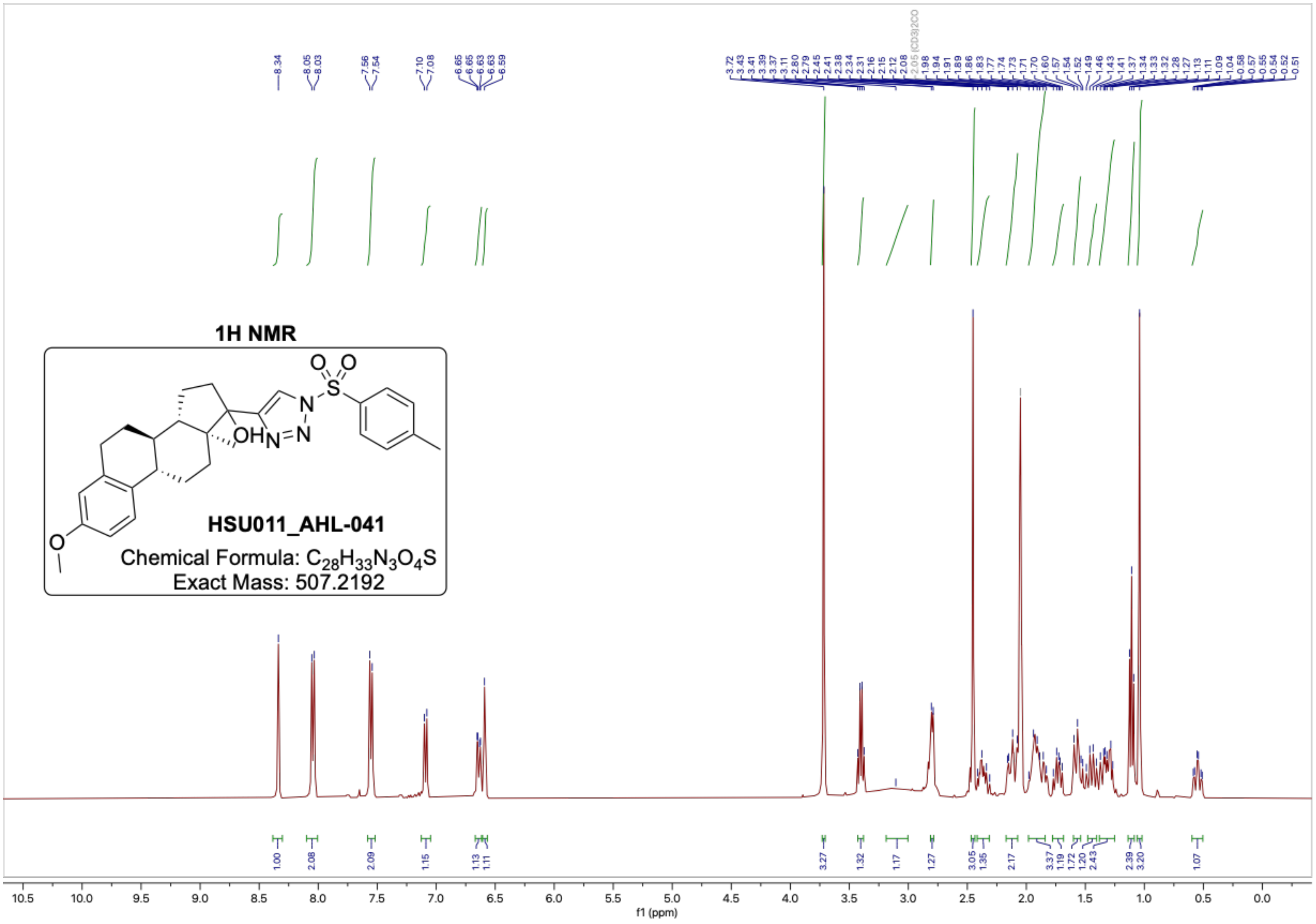
1H NMR
HSU011_AHL-041
Chemical Formula: C28H33N3O4S
Exact Mass: 507.2192
f1 (ppm)

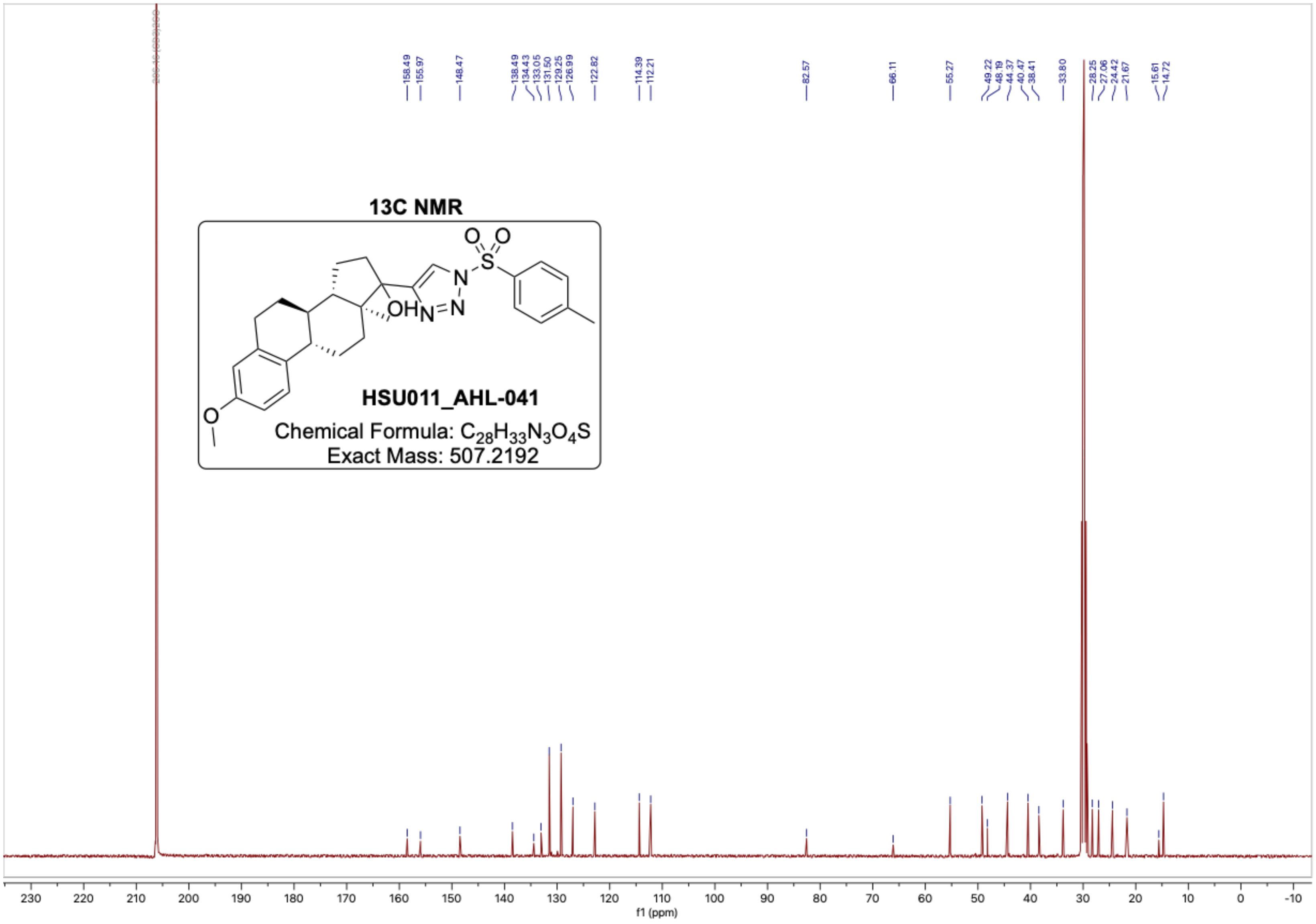

13C NMR
HSU011_AHL-041
Chemical Formula: C28H33N3O4S
Exact Mass: 507.2192
158.49
155.97
148.47
138.49
134.43
133.05
131.50
129.25
126.99
122.82
114.39
112.21
82.57
66.11
55.27
49.22
48.19
44.37
40.47
38.41
33.80
28.25
27.06
24.42
21.67
15.61
14.72
f1 (ppm)

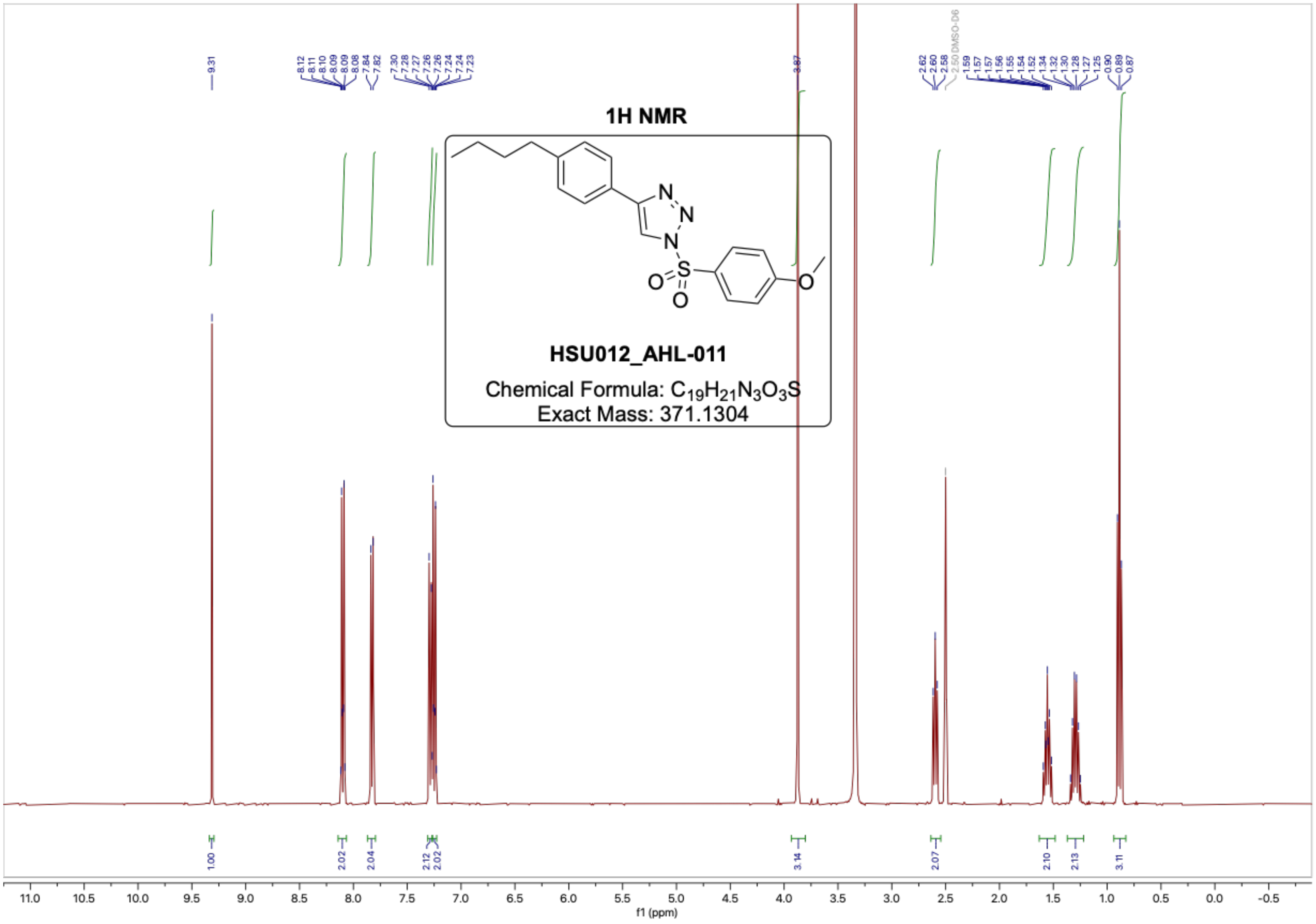

1H NMR
HSU012_AHL-011
Chemical Formula: C19H21N3O3S
Exact Mass: 371.1304
f1 (ppm)

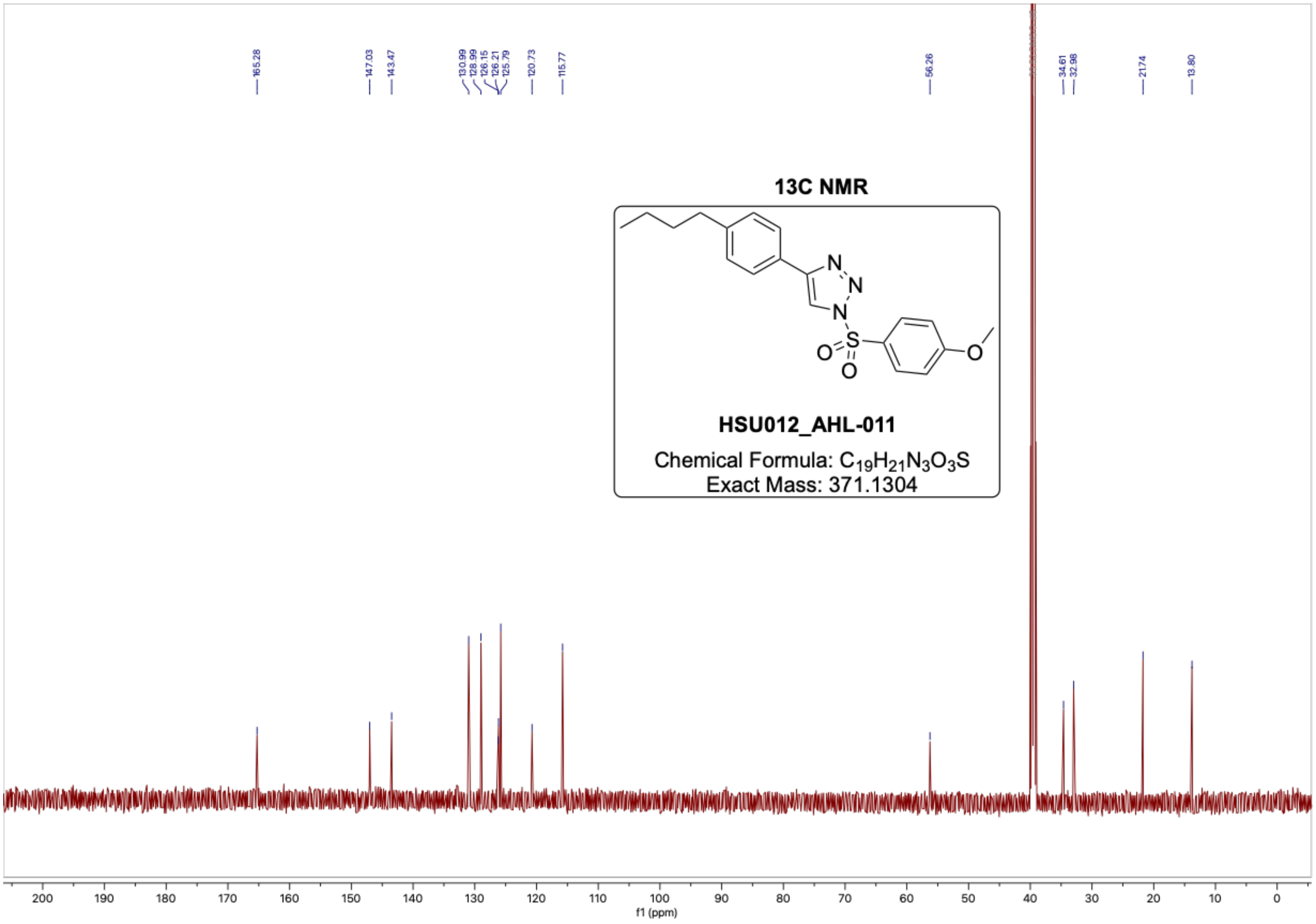

165.28
147.03
143.47
130.99
128.99
126.15
126.21
125.79
120.73
115.77
56.26
34.61
32.98
21.74
13.80
13C NMR
HSU012_AHL-011
Chemical Formula: C19H21N3O3S
Exact Mass: 371.1304
200
190
180
170
160
150
140
130
120
110
100
90
80
70
60
50
40
30
20
10
0
f1 (ppm)

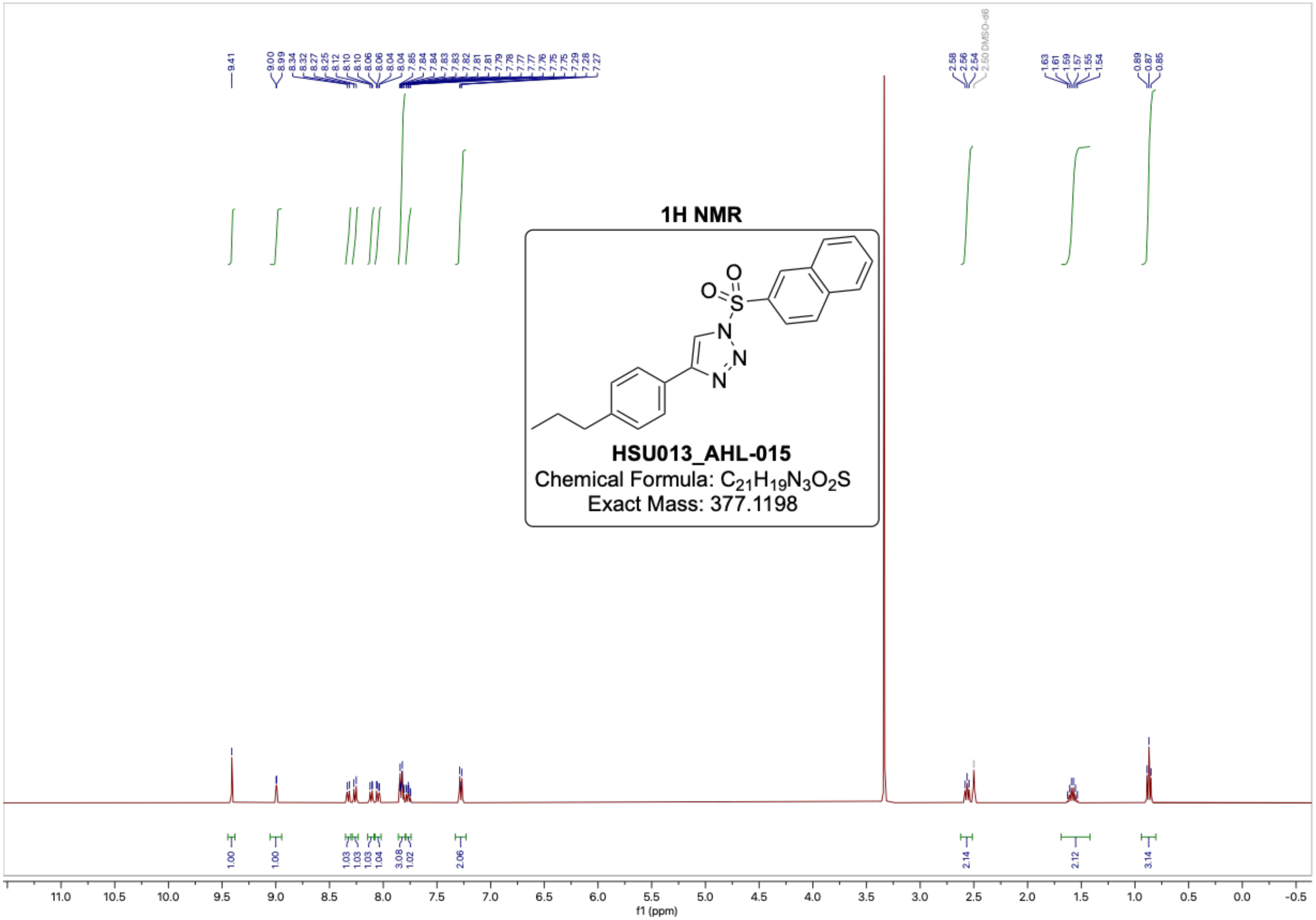
1H NMR
HSU013_AHL-015
Chemical Formula: $C_{21}H_{19}N_3O_2S$
Exact Mass: 377.1198
O
O
S
N
N
N
f1 (ppm)
9.41
9.00
8.99
2.58
2.56
2.54
2.50 DMSO-d6
0.89
0.87
0.85
1.00
1.00
1.03
1.03
1.03
1.04
3.08
1.02
2.06
2.14
2.12
3.14

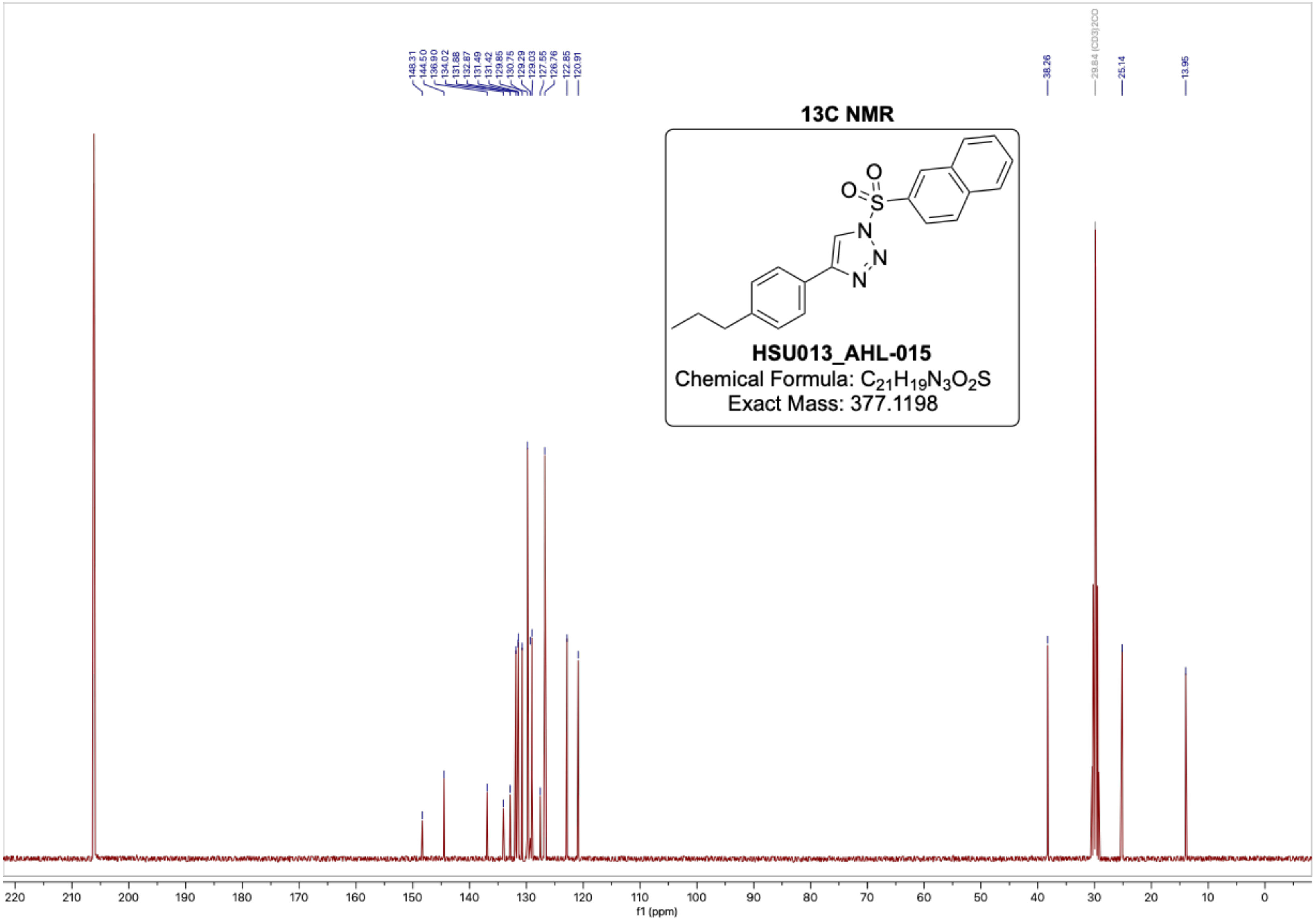

13C NMR
HSU013_AHL-015
Chemical Formula: C21H19N3O2S
Exact Mass: 377.1198
148.31
144.50
136.90
134.02
131.88
132.87
131.49
131.42
129.85
130.75
129.29
129.03
127.55
126.76
122.85
120.91
38.26
29.84 (CD3)2CO
25.14
13.95
f1 (ppm)

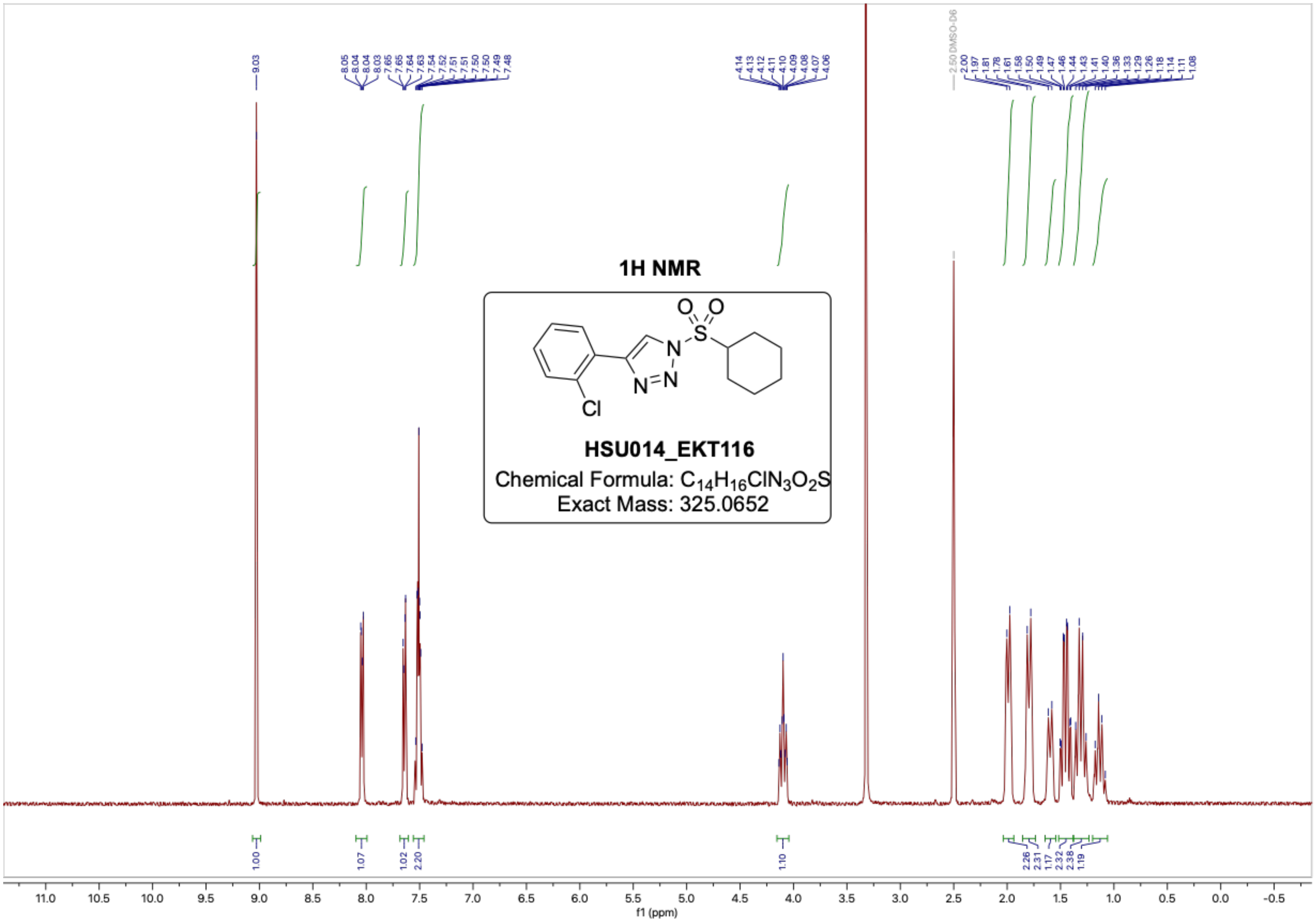

1H NMR
HSU014_EKT116
Chemical Formula: C14H16ClN3O2S
Exact Mass: 325.0652
f1 (ppm)
2.50 DMSO-D6

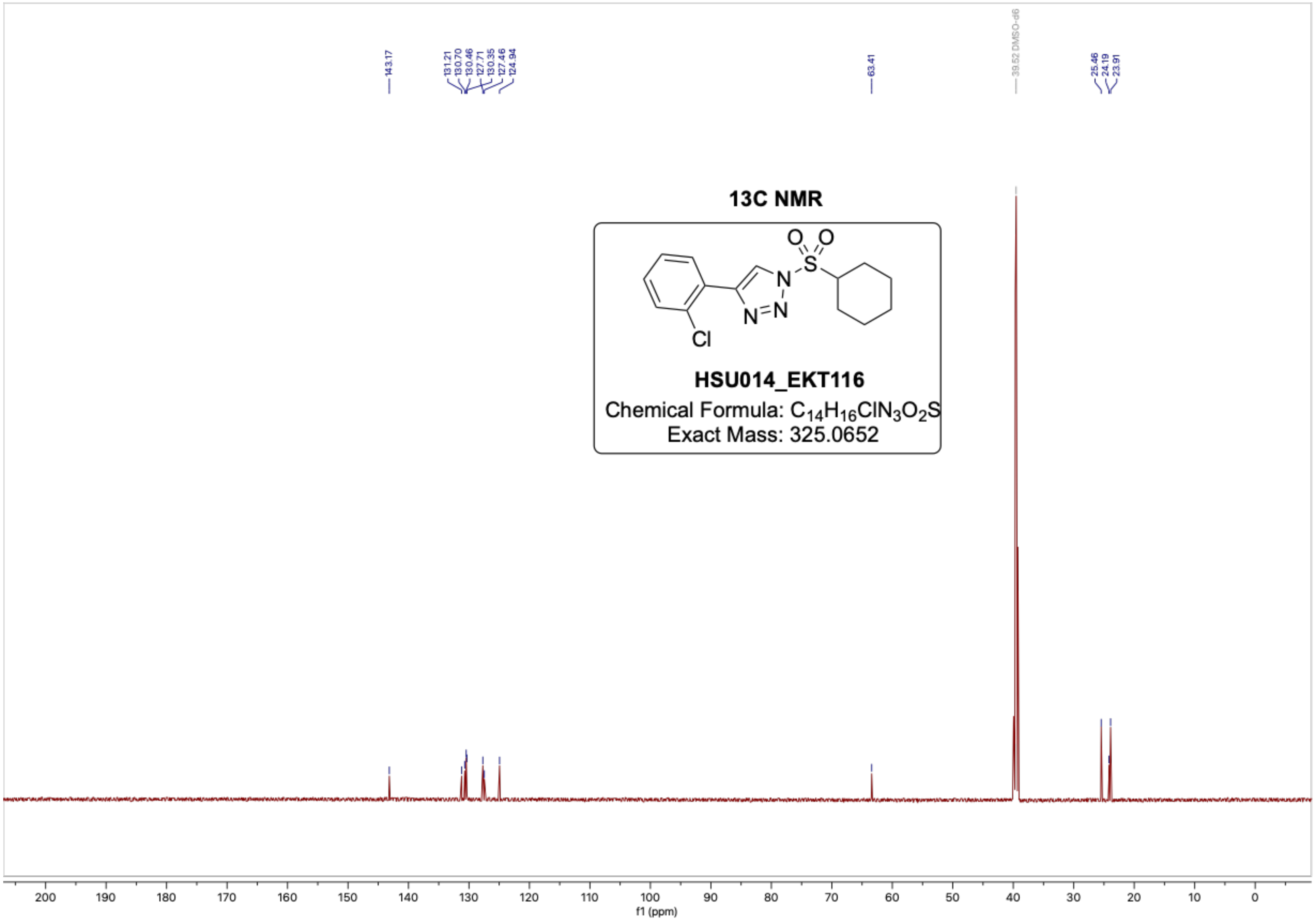

143.17
131.21
130.70
130.46
127.71
130.35
127.46
124.94
63.41
39.52 DMSO-d6
25.46
24.19
23.91
13C NMR
HSU014_EKT116
Chemical Formula: $C_{14}H_{16}ClN_3O_2S$
Exact Mass: 325.0652
f1 (ppm)

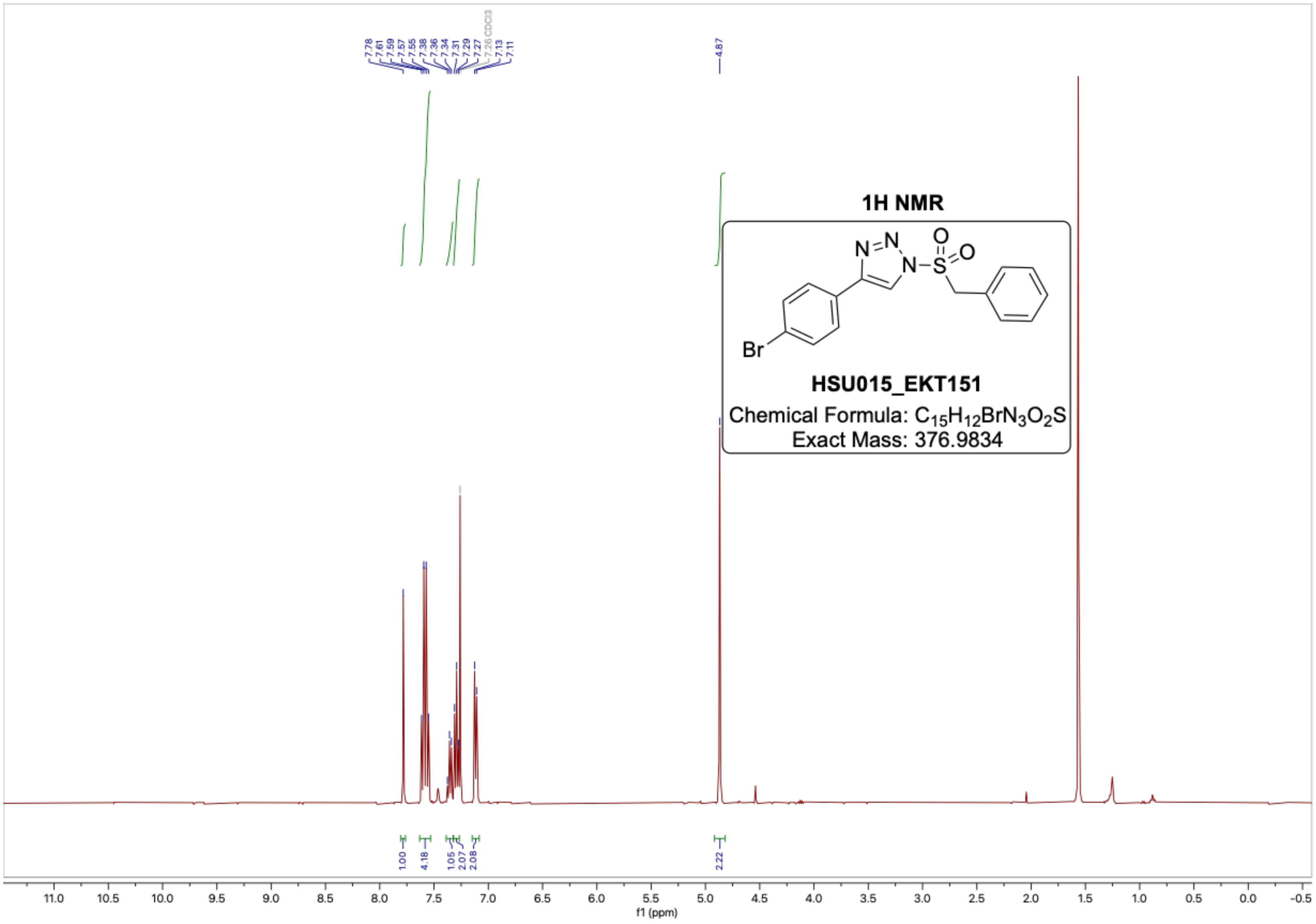
7.78
7.61
7.59
7.57
7.55
7.38
7.36
7.34
7.31
7.29
7.27
7.26 CDCl3
7.13
7.11
4.87
1H NMR
HSU015_EKT151
Chemical Formula: C15H12BrN3O2S
Exact Mass: 376.9834
1.00
4.18
1.05
2.07
2.08
2.22
f1 (ppm)

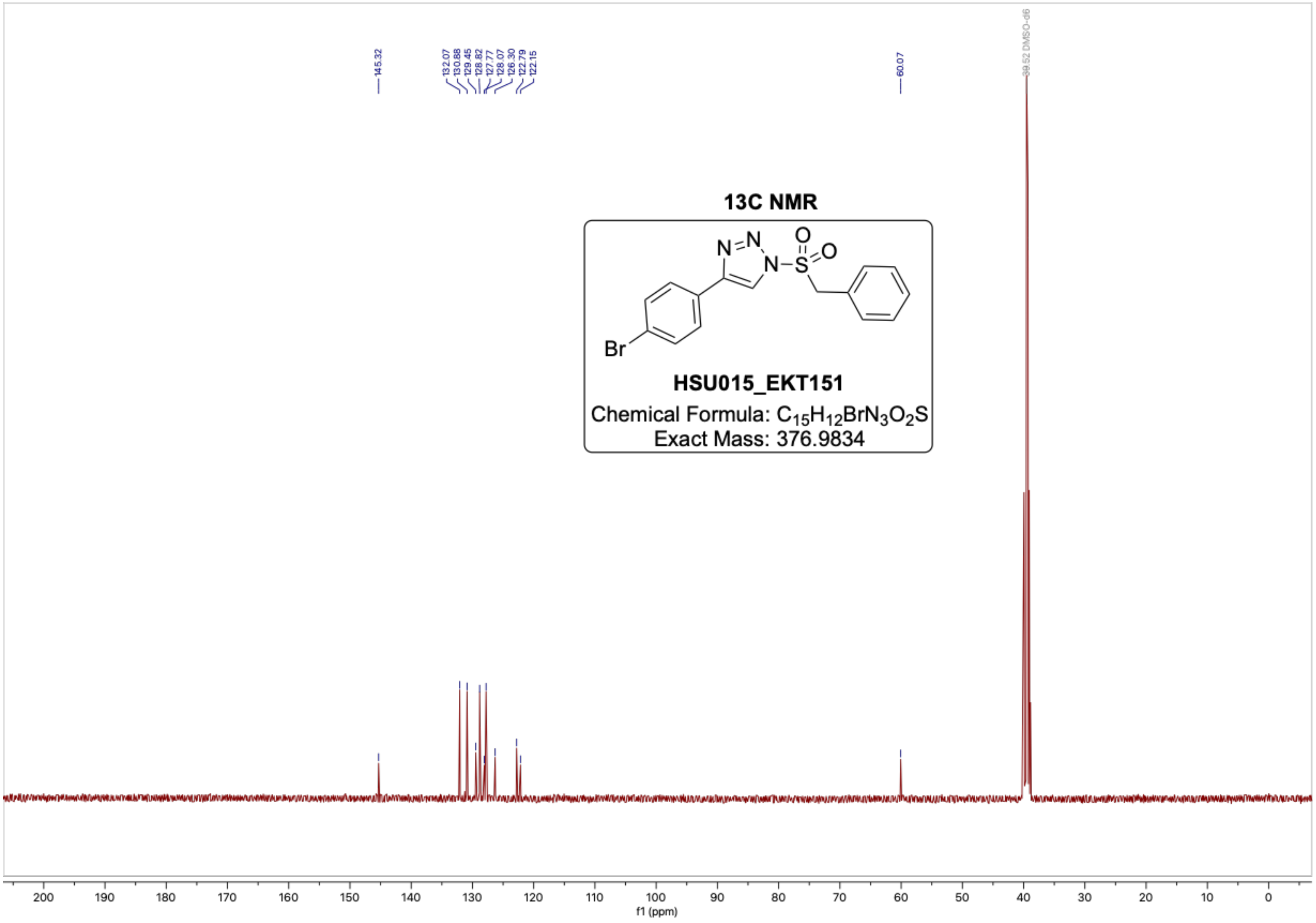

145.32
132.07
130.88
129.45
128.82
127.77
128.07
126.30
122.79
122.15
60.07
39.52 DMSO-d6
13C NMR
HSU015_EKT151
Chemical Formula: C15H12BrN3O2S
Exact Mass: 376.9834
f1 (ppm)

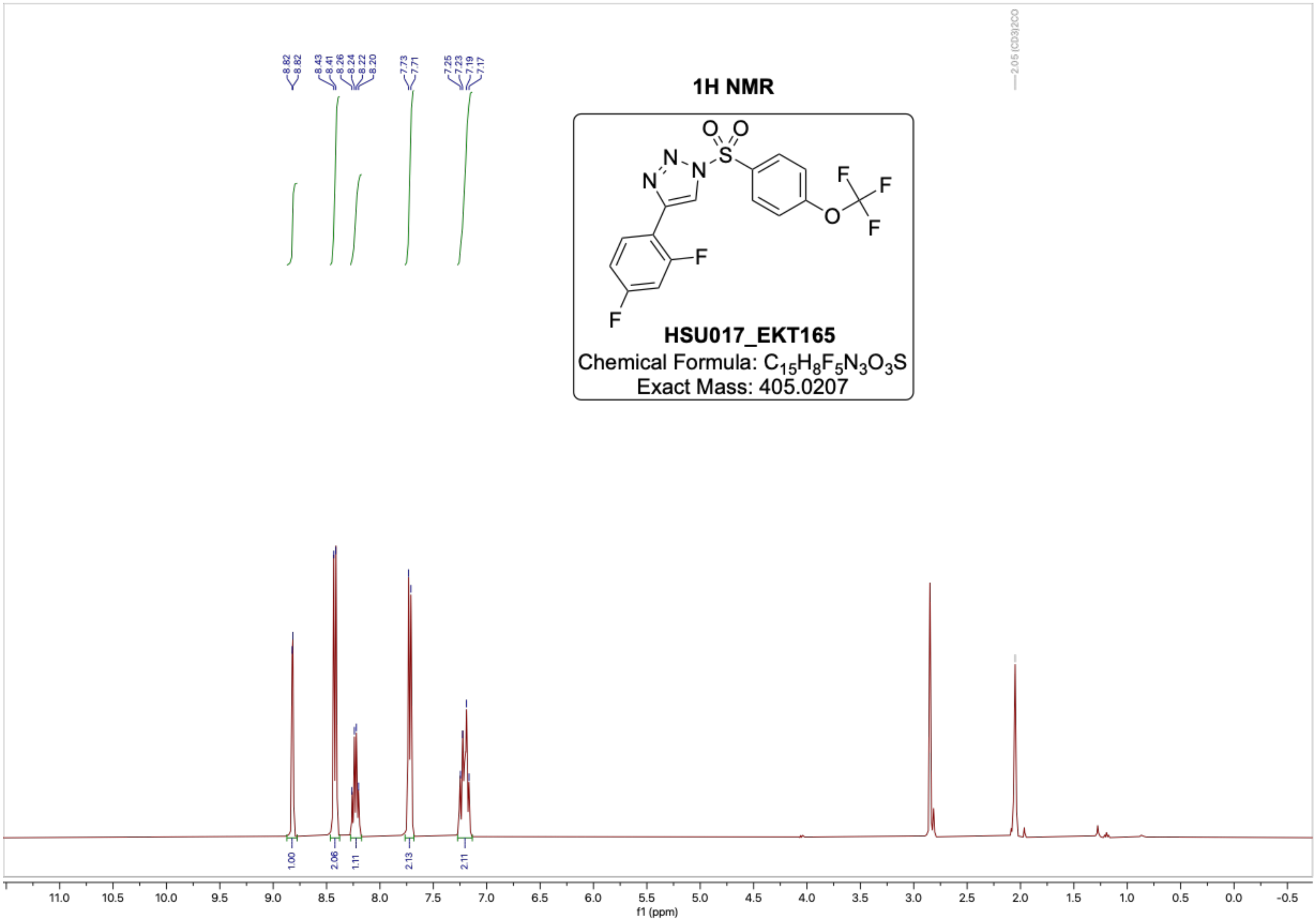

1H NMR
HSU017_EKT165
Chemical Formula: C15H8F5N3O3S
Exact Mass: 405.0207
f1 (ppm)

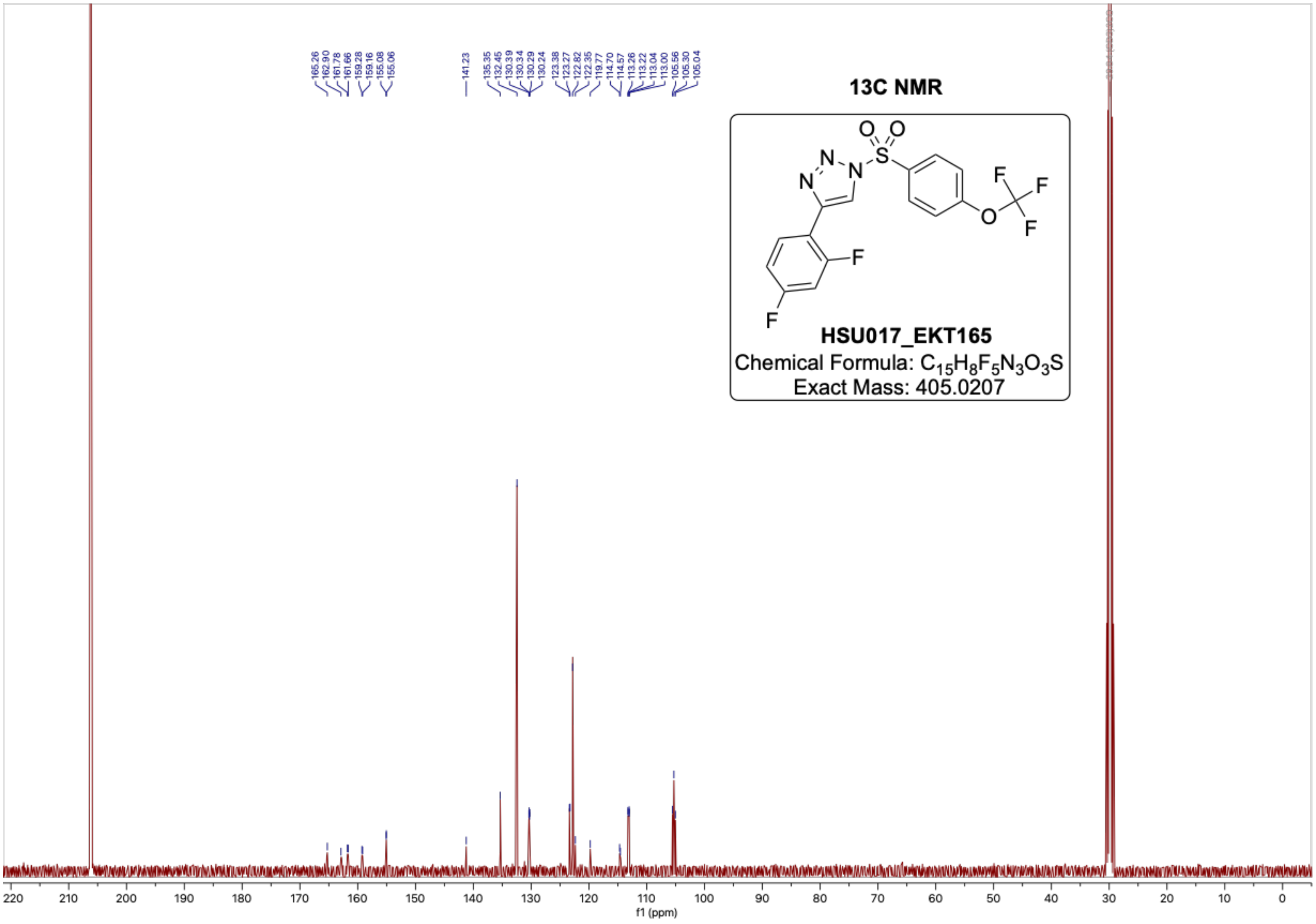

165.26
162.90
161.78
161.66
159.28
159.16
155.08
155.06
141.23
135.35
132.45
130.39
130.34
130.29
130.24
123.38
123.27
122.82
122.35
119.77
114.70
114.57
113.26
113.22
113.04
113.00
105.56
105.30
105.04
13C NMR
HSU017_EKT165
Chemical Formula: $C_{15}H_8F_5N_3O_3S$
Exact Mass: 405.0207
f1 (ppm)

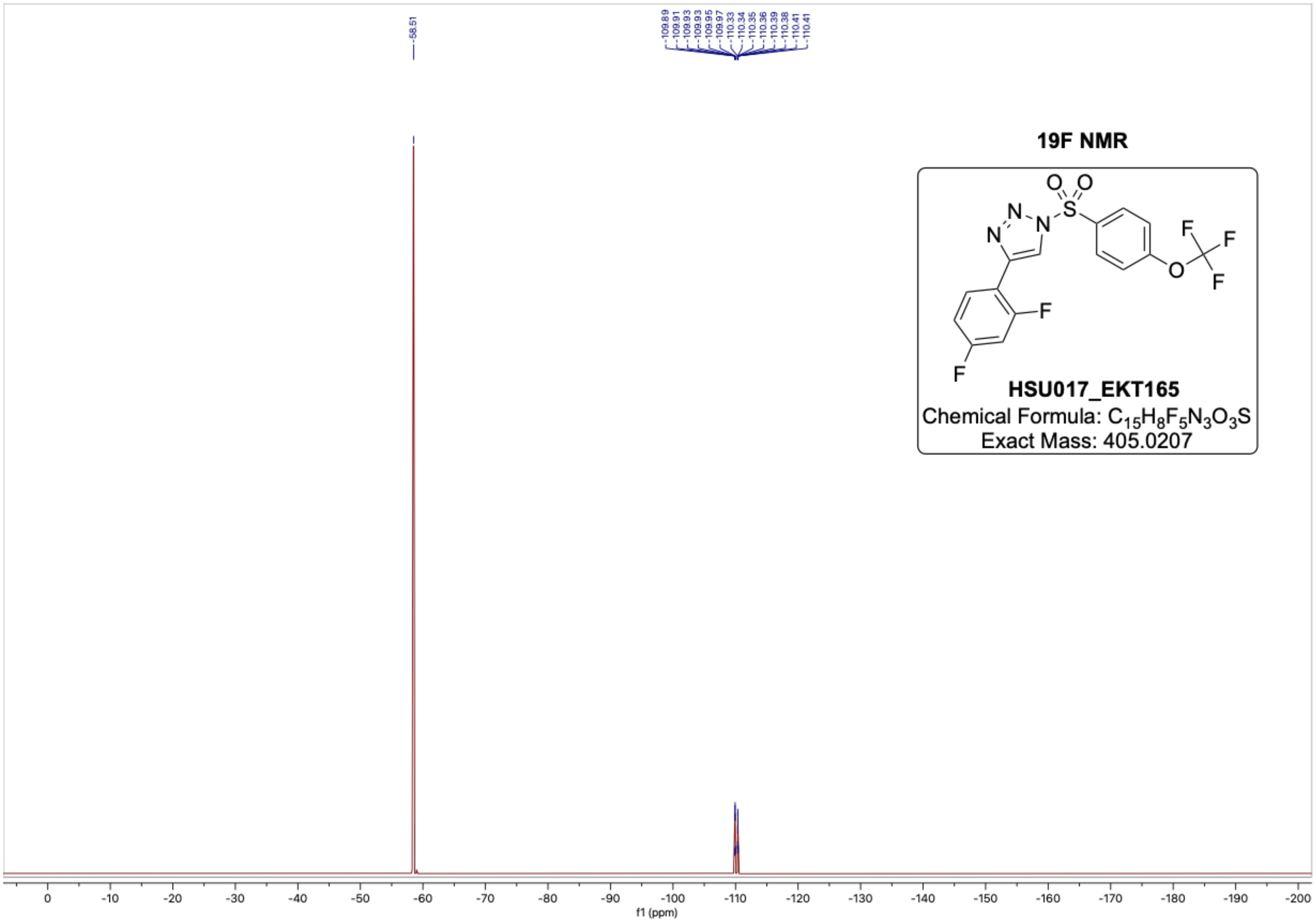
-58.51
-109.89
-109.91
-109.93
-109.93
-109.95
-109.97
-110.33
-110.34
-110.35
-110.36
-110.38
-110.39
-110.41
-110.41
19F NMR
HSU017_EKT165
Chemical Formula: C15H8F5N3O3S
Exact Mass: 405.0207
f1 (ppm)

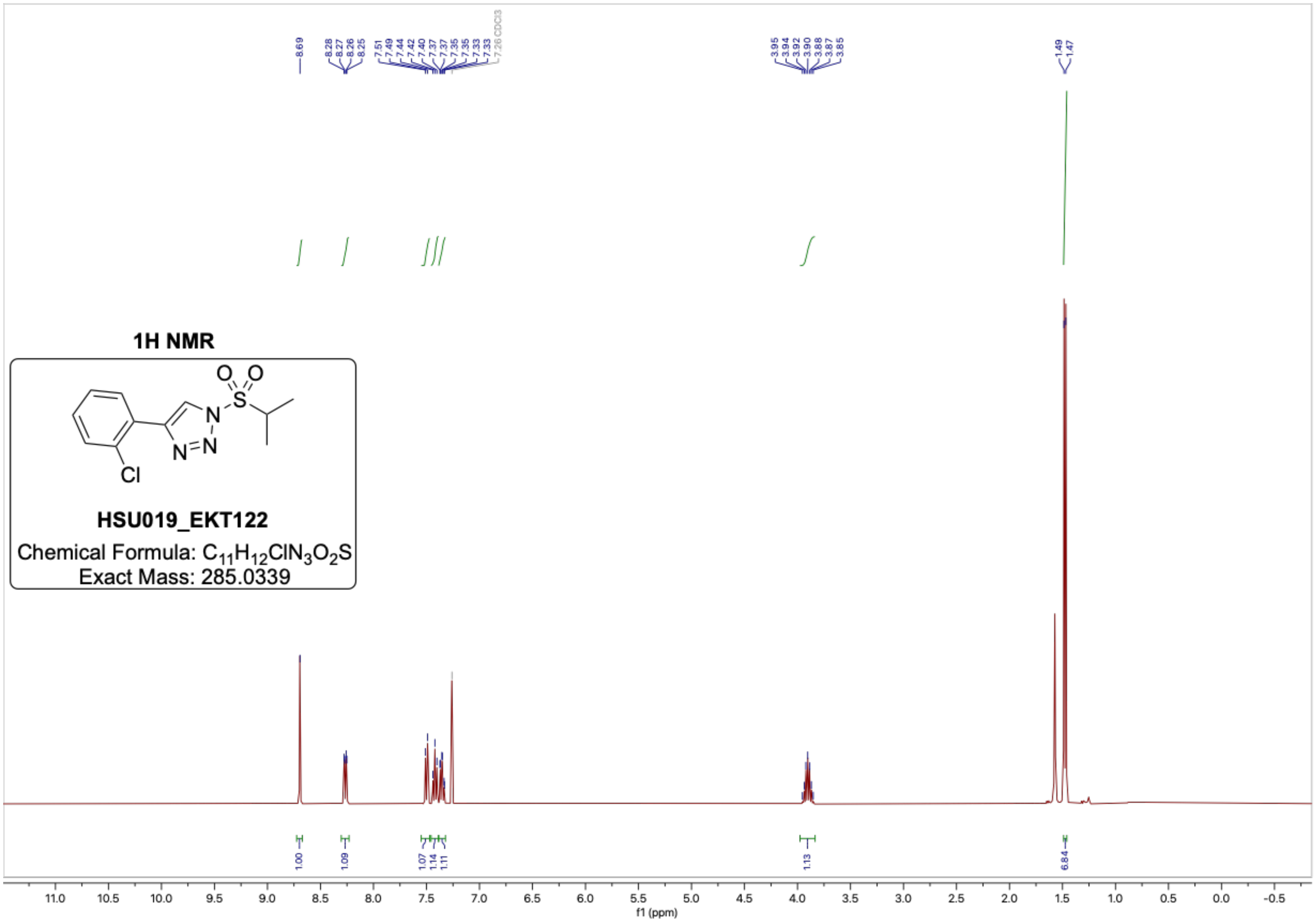

1H NMR
HSU019_EKT122
Chemical Formula: C11H12ClN3O2S
Exact Mass: 285.0339
f1 (ppm)

13C NMR

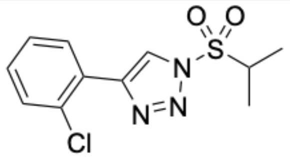

HSU019_EKT122

Chemical Formula: $C_{11}H_{12}ClN_3O_2S$

Exact Mass: 285.0339

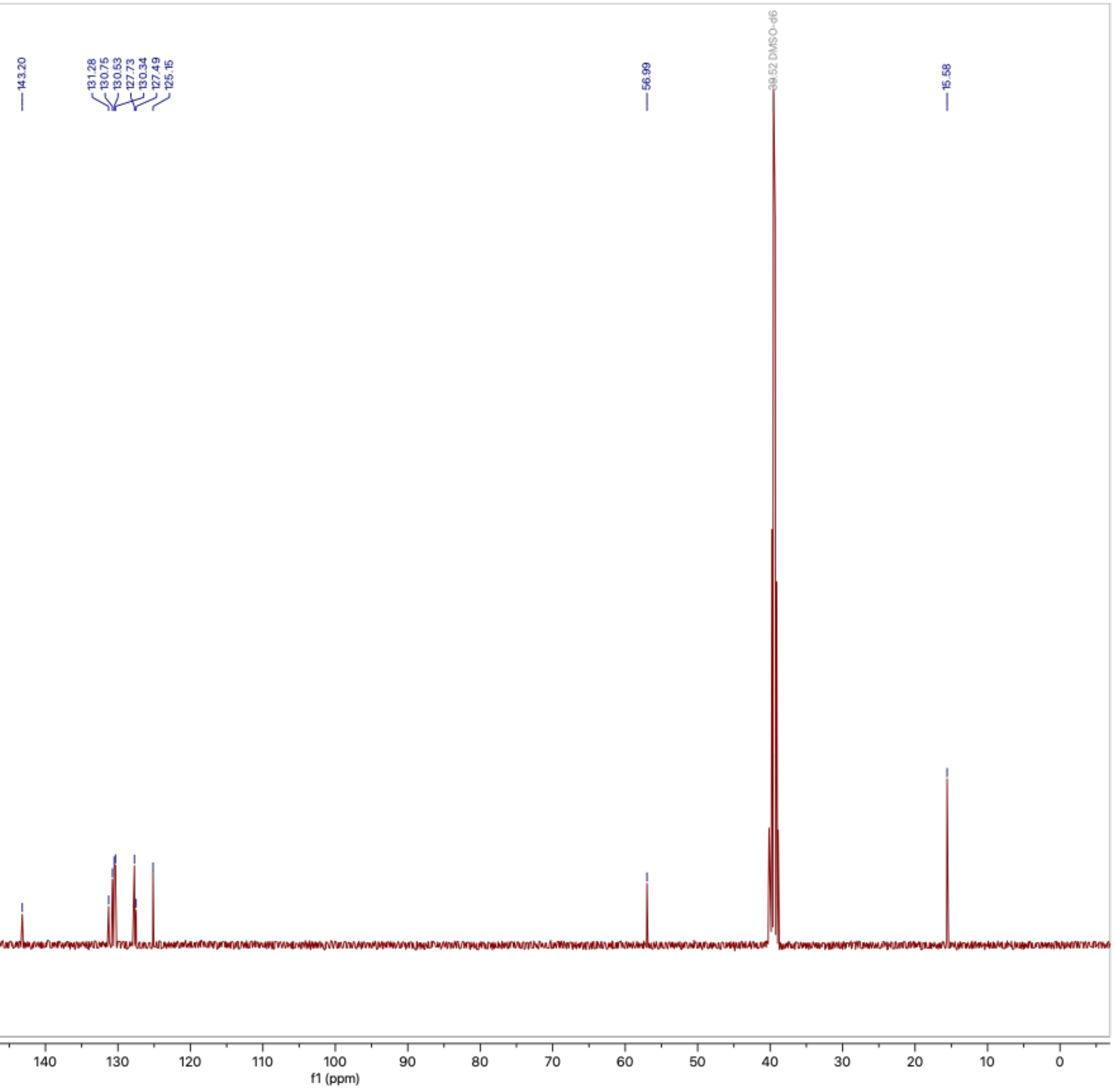

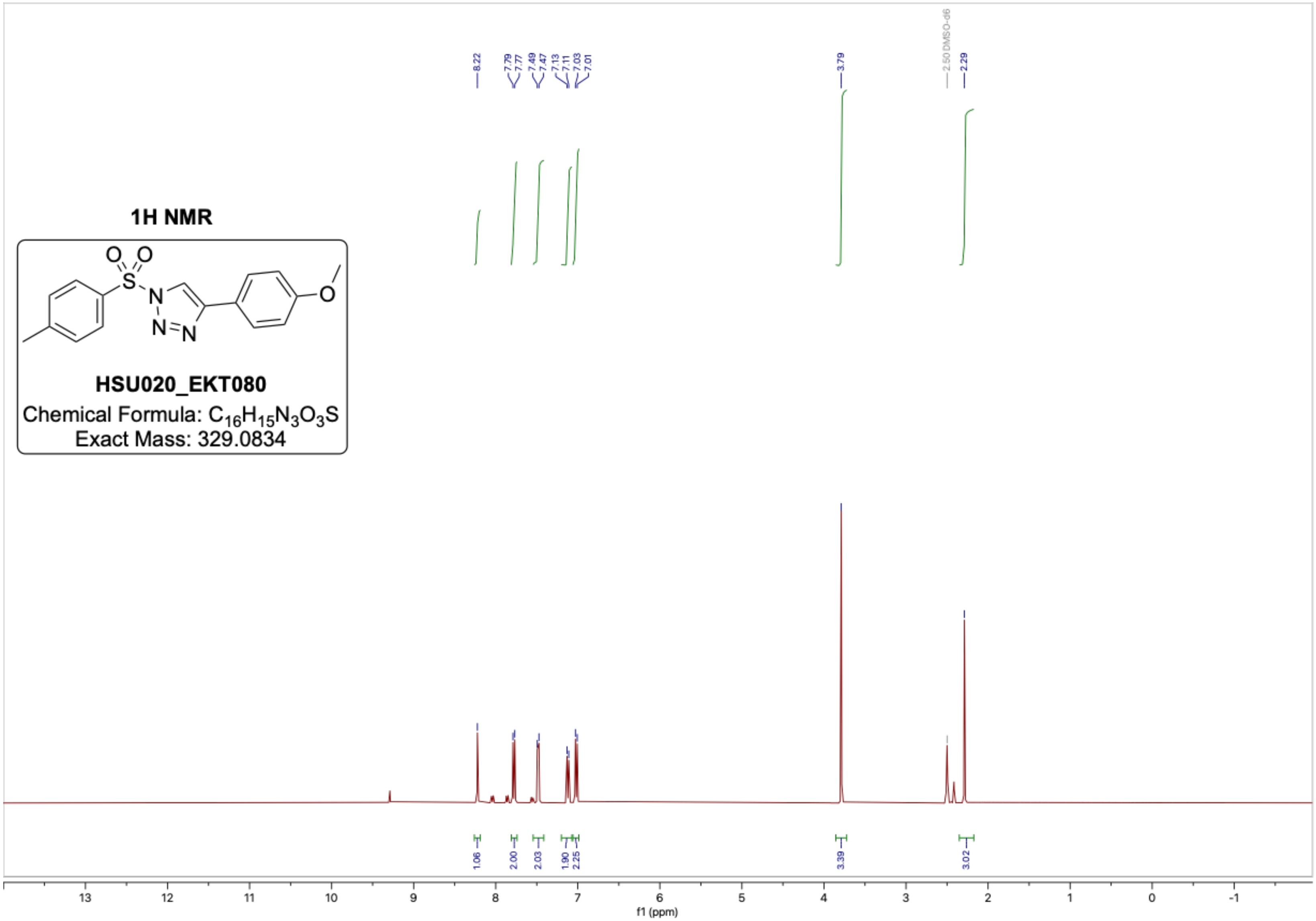
1H NMR
HSU020_EKT080
Chemical Formula: C16H15N3O3S
Exact Mass: 329.0834
8.22
7.79
7.77
7.49
7.47
7.13
7.11
7.03
7.01
3.79
2.50 DMSO-d6
2.29
1.06
2.00
2.03
1.90
2.25
3.39
3.02
f1 (ppm)

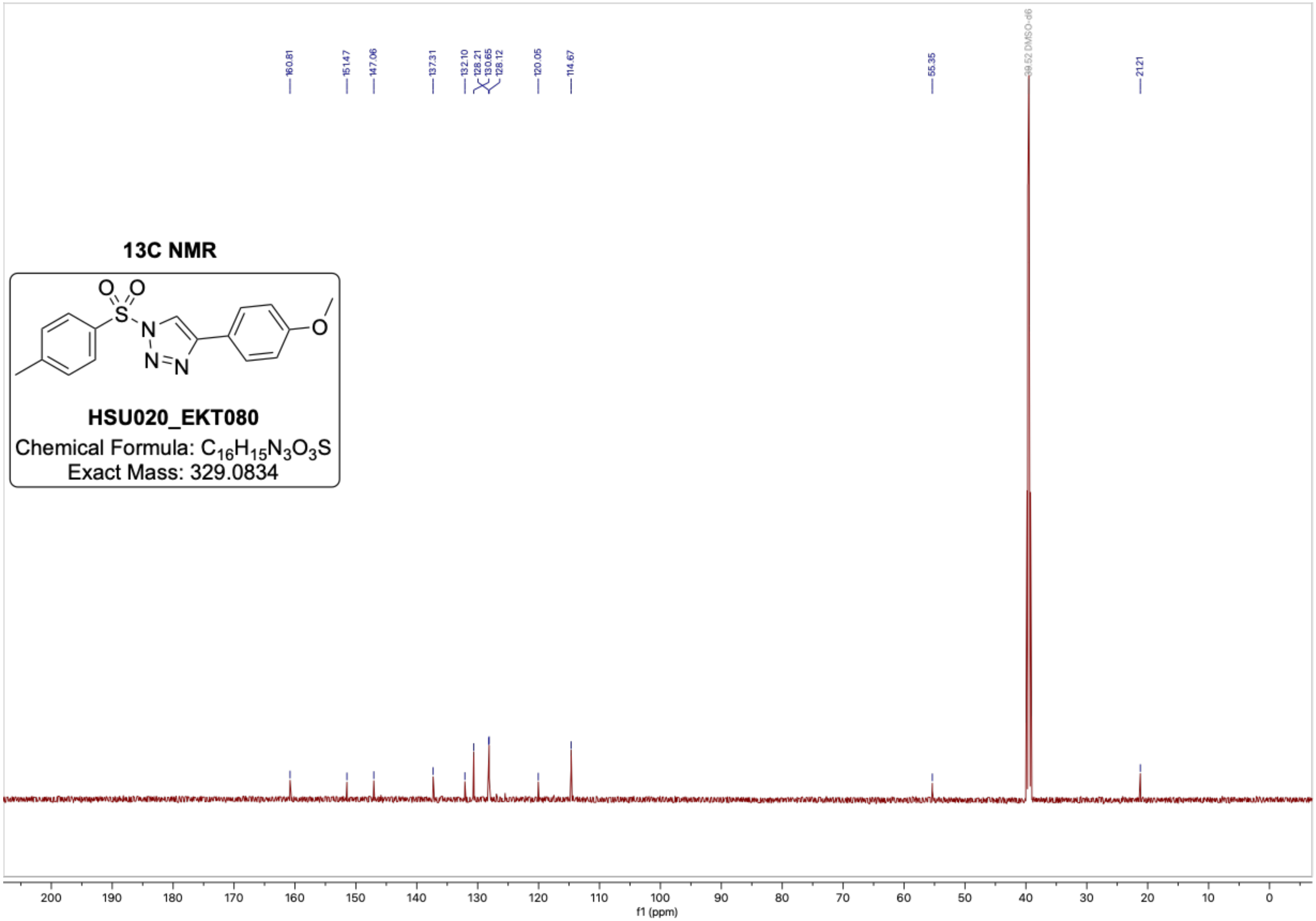

13C NMR
HSU020_EKT080
Chemical Formula: $C_{16}H_{15}N_3O_3S$
Exact Mass: 329.0834
160.81
151.47
147.06
137.31
132.10
128.21
130.65
128.12
120.05
114.67
55.35
39.52 DMSO-d6
21.21
200 190 180 170 160 150 140 130 120 110 100 90 80 70 60 50 40 30 20 10 0
f1 (ppm)

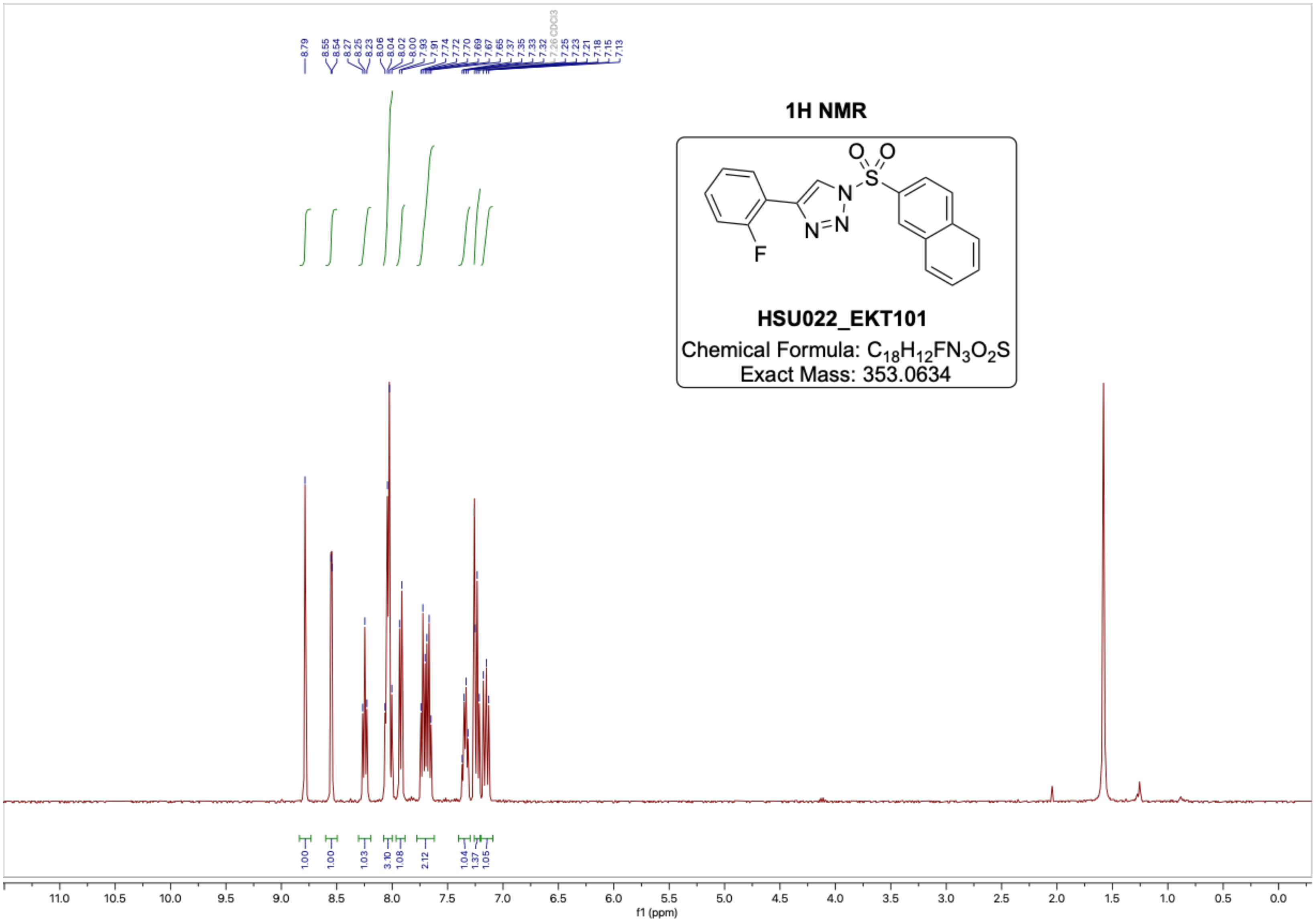

1H NMR
HSU022_EKT101
Chemical Formula: C18H12FN3O2S
Exact Mass: 353.0634
f1 (ppm)

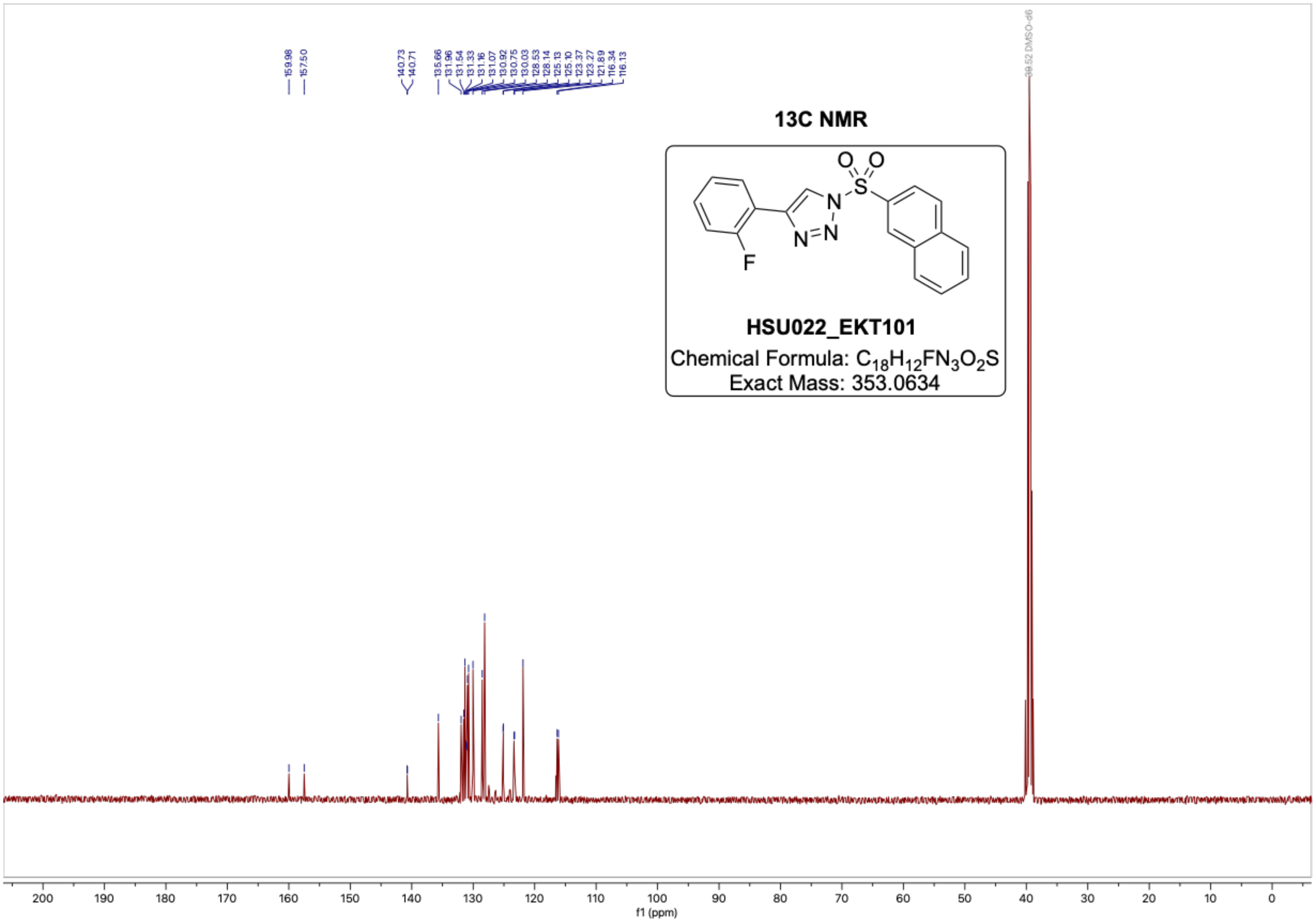

13C NMR
HSU022_EKT101
Chemical Formula: C18H12FN3O2S
Exact Mass: 353.0634
159.98
157.50
140.73
140.71
135.66
131.96
131.54
131.33
131.16
131.07
130.92
130.75
130.03
128.53
128.14
125.13
125.10
123.37
123.27
121.89
116.34
116.13
39.52 DMSO-d6
f1 (ppm)

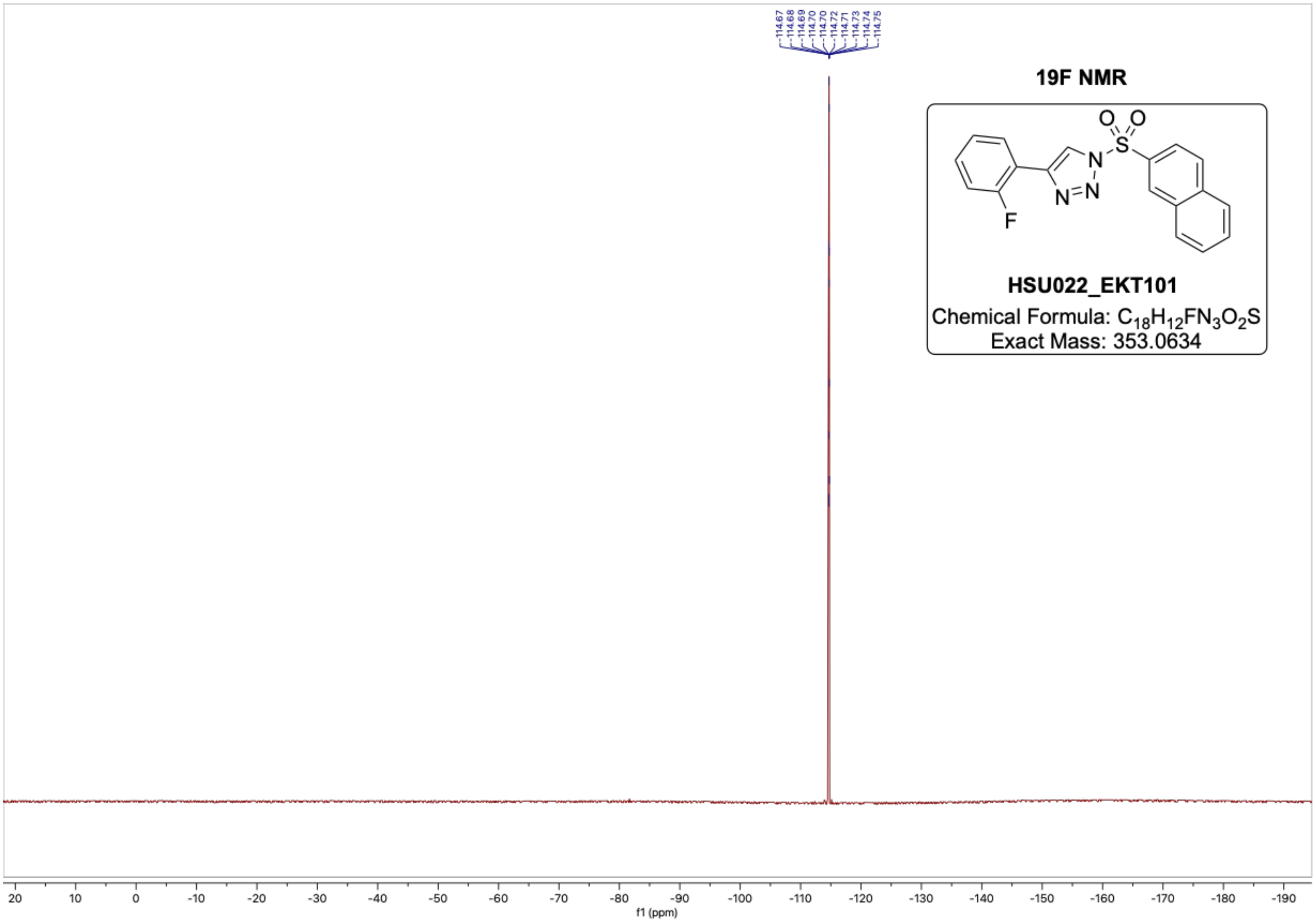
-114.67
-114.68
-114.69
-114.70
-114.70
-114.72
-114.71
-114.73
-114.74
-114.75
19F NMR
F
N
N=N
S
O O
HSU022_EKT101
Chemical Formula: $C_{18}H_{12}FN_3O_2S$
Exact Mass: 353.0634
20 10 0 -10 -20 -30 -40 -50 -60 -70 -80 -90 -100 -110 -120 -130 -140 -150 -160 -170 -180 -190
f1 (ppm)

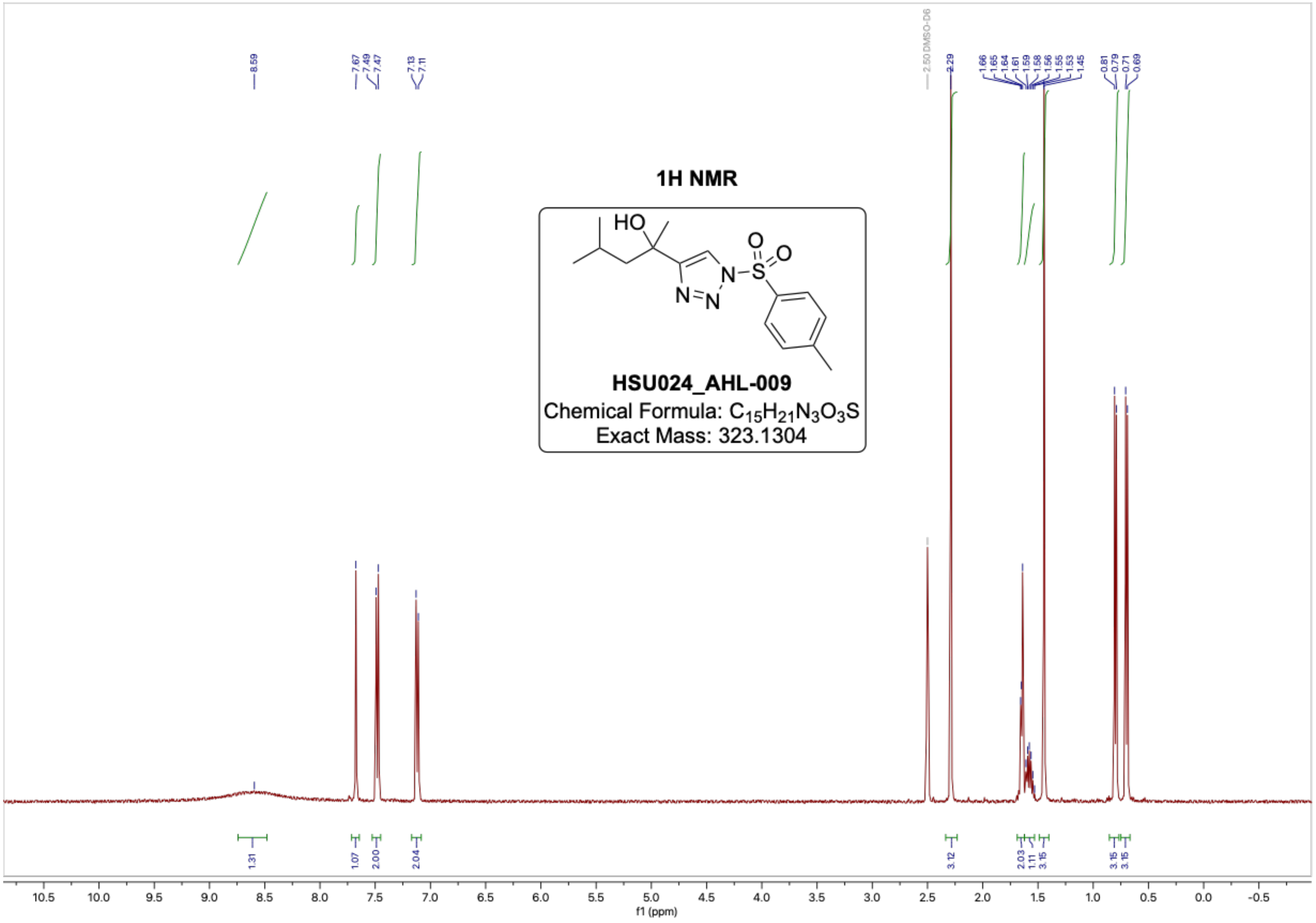
1H NMR
HO
O
S
O
N
N
N
HSU024_AHL-009
Chemical Formula: C15H21N3O3S
Exact Mass: 323.1304
8.59
7.67
7.49
7.47
7.13
7.11
2.50 DMSO-D6
2.29
1.66
1.65
1.64
1.61
1.59
1.58
1.56
1.55
1.53
1.45
0.81
0.79
0.71
0.69
1.31
1.07
2.00
2.04
3.12
2.03
1.11
3.15
3.15
3.15
f1 (ppm)

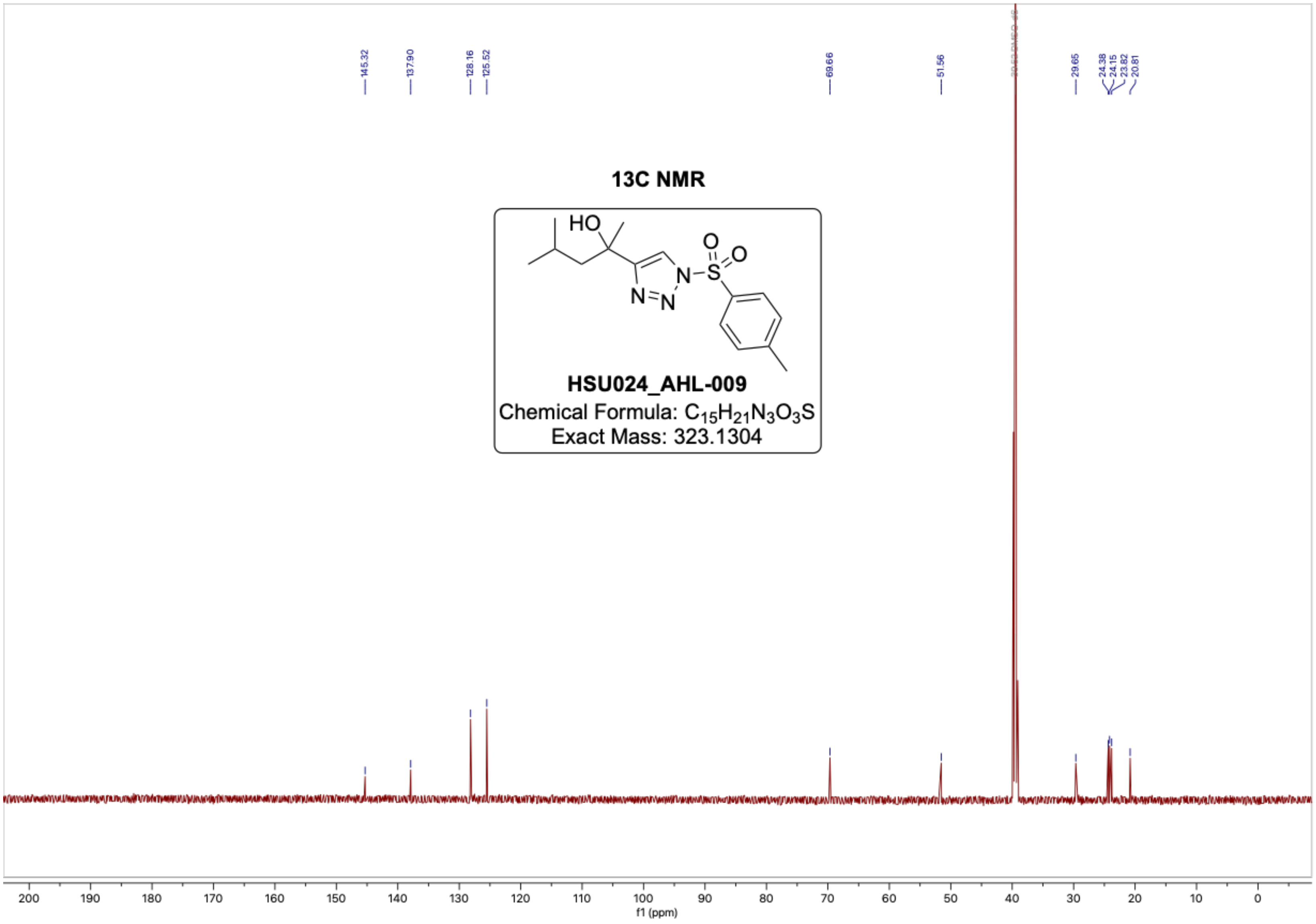

145.32
137.90
128.16
125.52
69.66
51.56
29.65
24.38
24.15
23.82
20.81
13C NMR
HO
O
S
O
N
N
N
HSU024_AHL-009
Chemical Formula: C15H21N3O3S
Exact Mass: 323.1304
200
190
180
170
160
150
140
130
120
110
100
90
80
70
60
50
40
30
20
10
0
f1 (ppm)

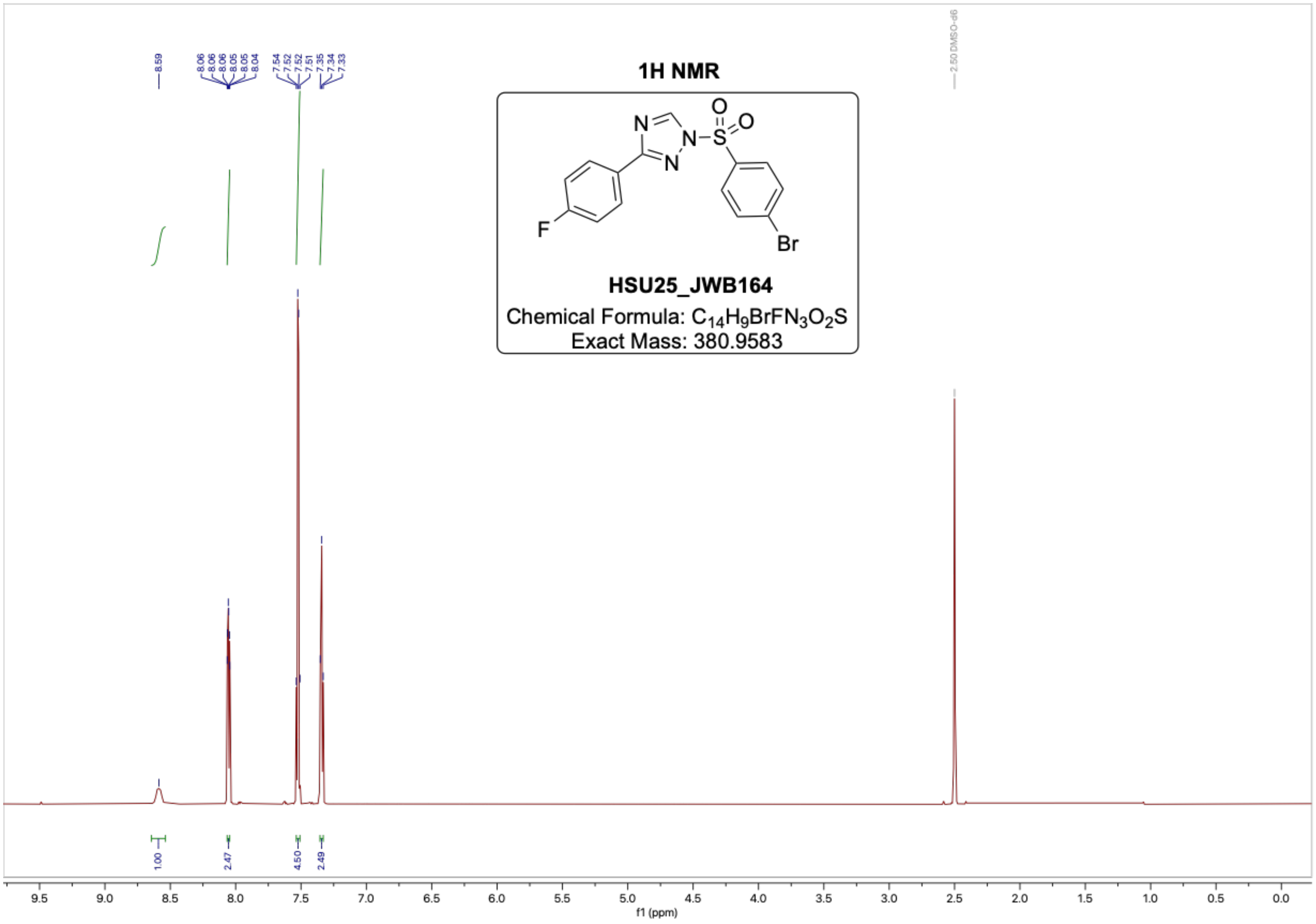
1H NMR
HSU25_JWB164
Chemical Formula: C14H9BrFN3O2S
Exact Mass: 380.9583
8.59
8.06
8.06
8.06
8.05
8.05
8.04
7.54
7.52
7.52
7.51
7.35
7.34
7.33
2.50 DMSO-d6
1.00
2.47
4.50
2.49
f1 (ppm)

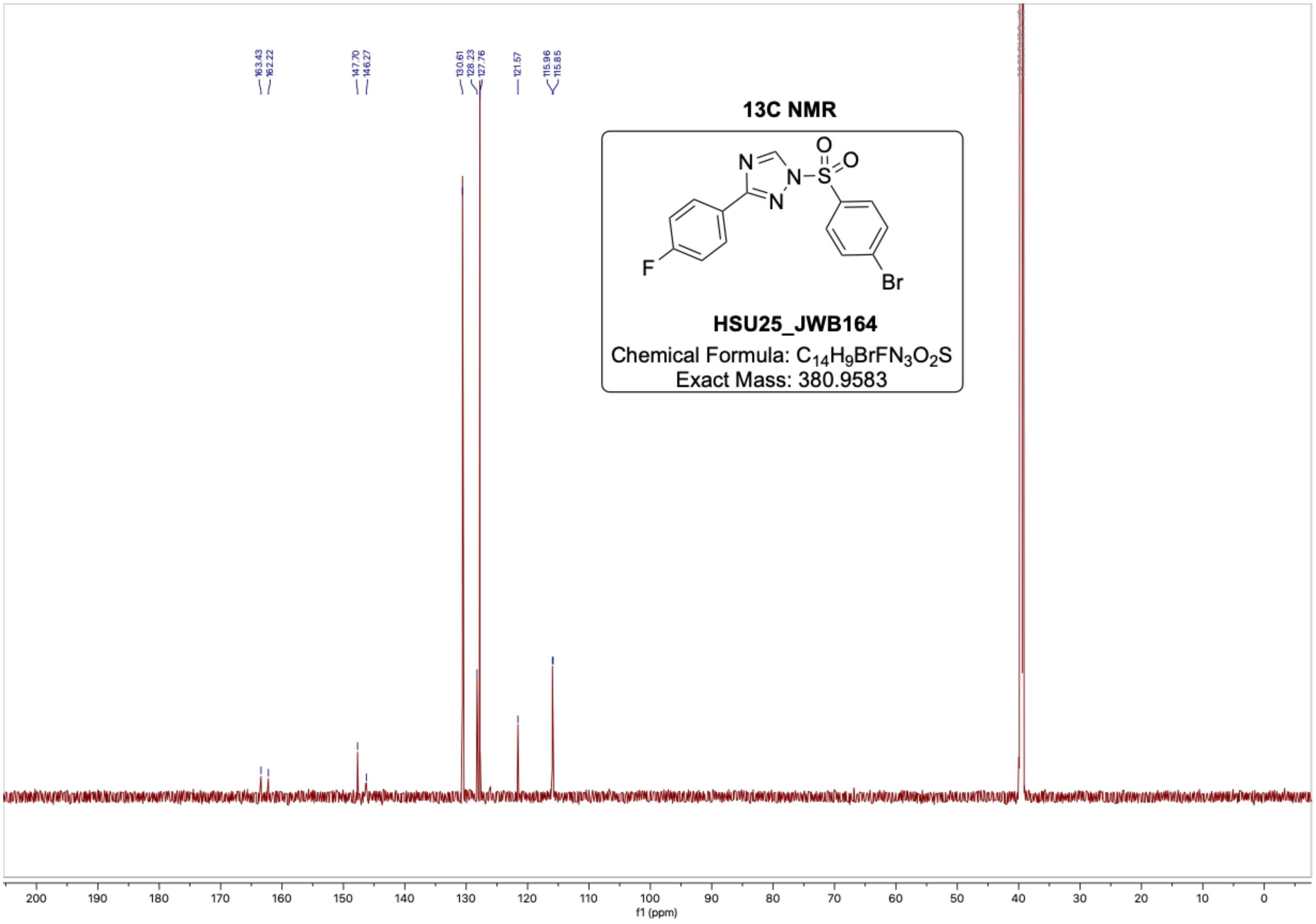
163.43
162.22
147.70
146.27
130.61
128.23
127.76
121.57
115.96
115.85
13C NMR
N
N
N
O
S
O
F
Br
HSU25_JWB164
Chemical Formula: $C_{14}H_9BrFN_3O_2S$
Exact Mass: 380.9583
200
190
180
170
160
150
140
130
120
110
100
90
80
70
60
50
40
30
20
10
0
f1 (ppm)

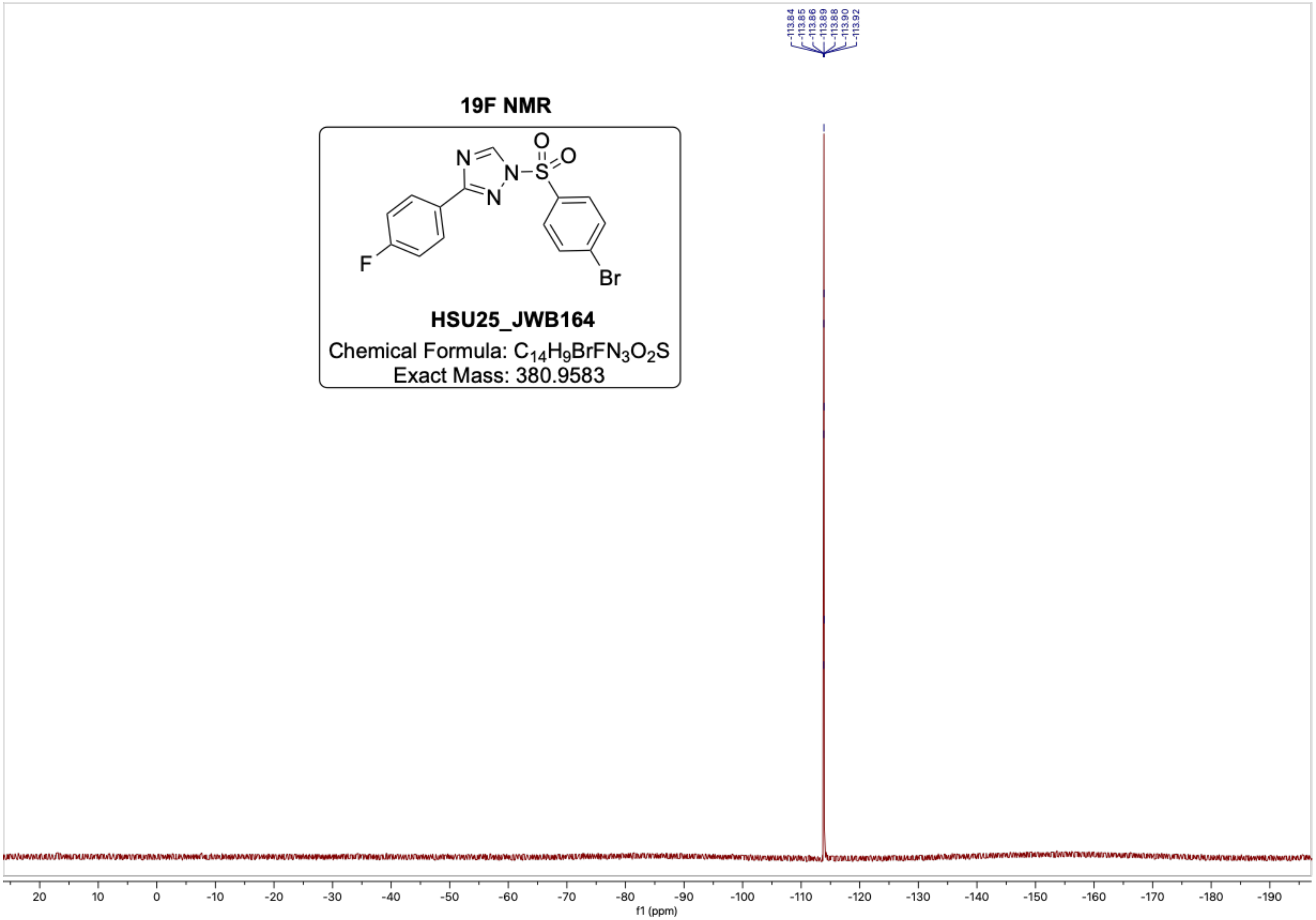
-113.84
-113.85
-113.86
-113.89
-113.88
-113.90
-113.92
19F NMR
N
N
N
S
O
O
F
Br
HSU25_JWB164
Chemical Formula: $C_{14}H_9BrFN_3O_2S$
Exact Mass: 380.9583
20
10
0
-10
-20
-30
-40
-50
-60
-70
-80
-90
-100
-110
-120
-130
-140
-150
-160
-170
-180
-190
f1 (ppm)

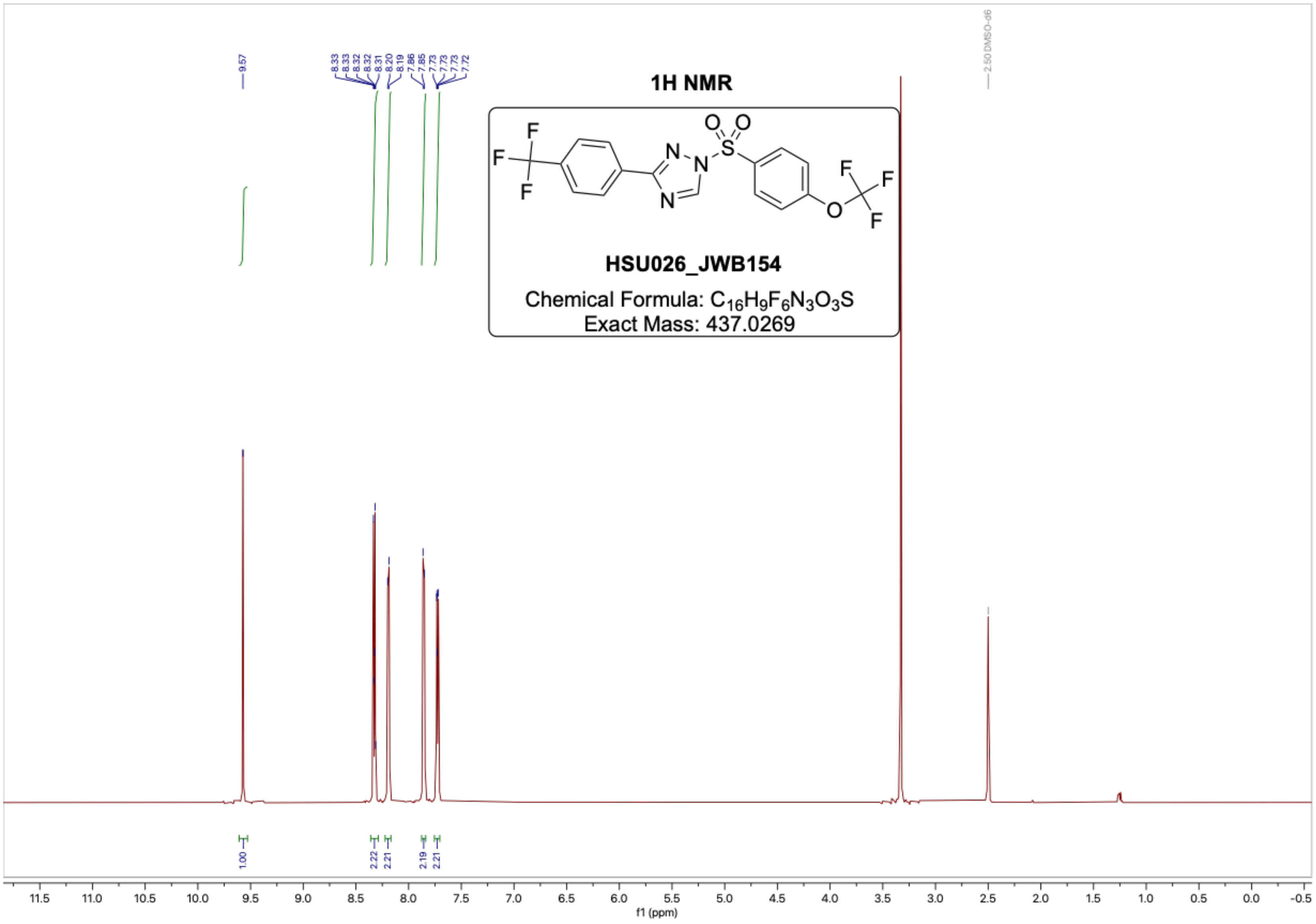

1H NMR
HSU026_JWB154
Chemical Formula: C16H9F6N3O3S
Exact Mass: 437.0269
9.57
8.33
8.33
8.32
8.32
8.31
8.20
8.19
7.86
7.85
7.73
7.73
7.73
7.72
2.50 DMSO-d6
1.00
2.22
2.21
2.19
2.21
f1 (ppm)

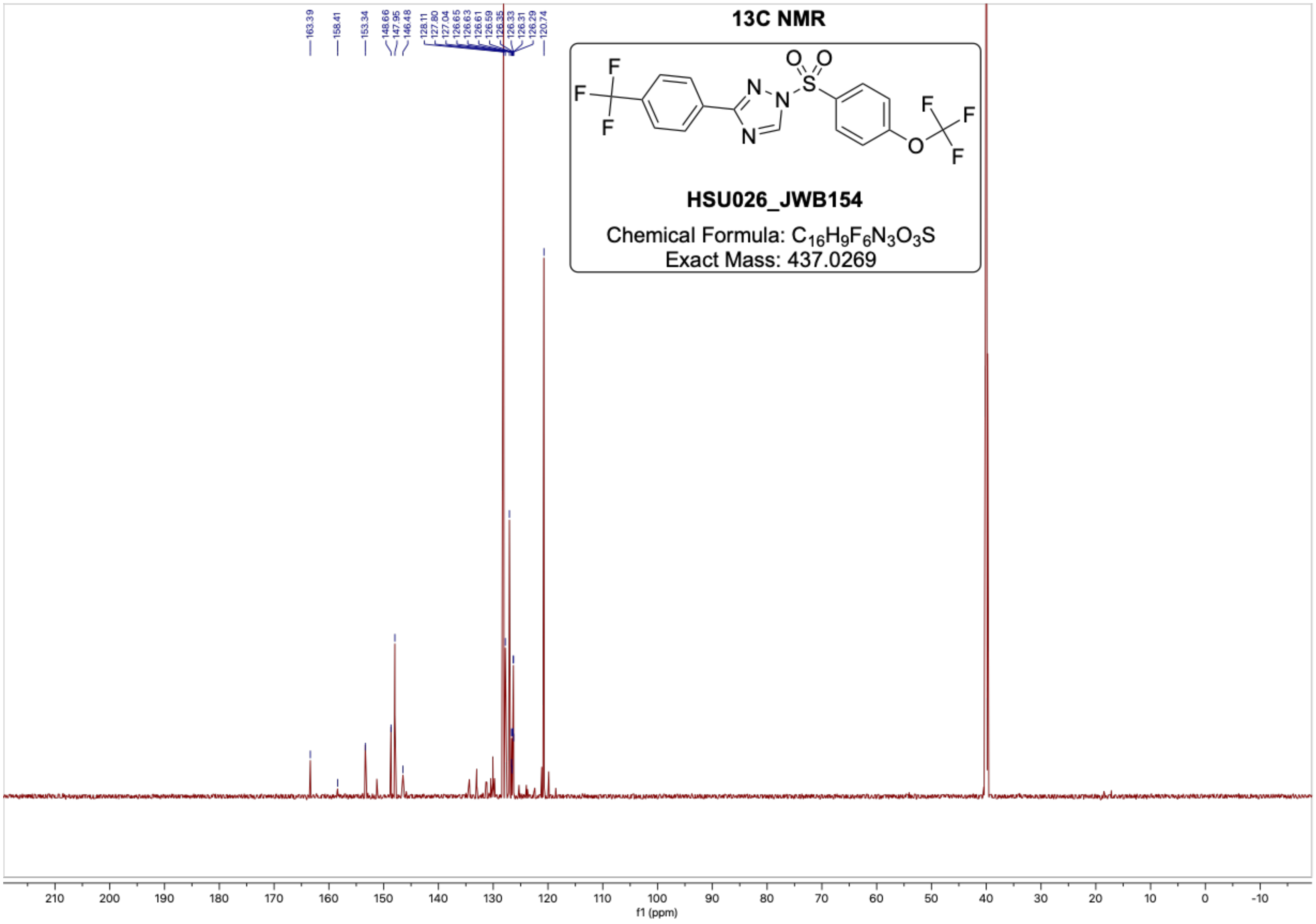

13C NMR
163.39
158.41
153.34
148.66
147.95
146.48
128.11
127.80
127.04
126.65
126.63
126.61
126.59
126.35
126.33
126.31
126.29
120.74
HSU026_JWB154
Chemical Formula: $C_{16}H_9F_6N_3O_3S$
Exact Mass: 437.0269
210 200 190 180 170 160 150 140 130 120 110 100 90 80 70 60 50 40 30 20 10 0 -10
f1 (ppm)

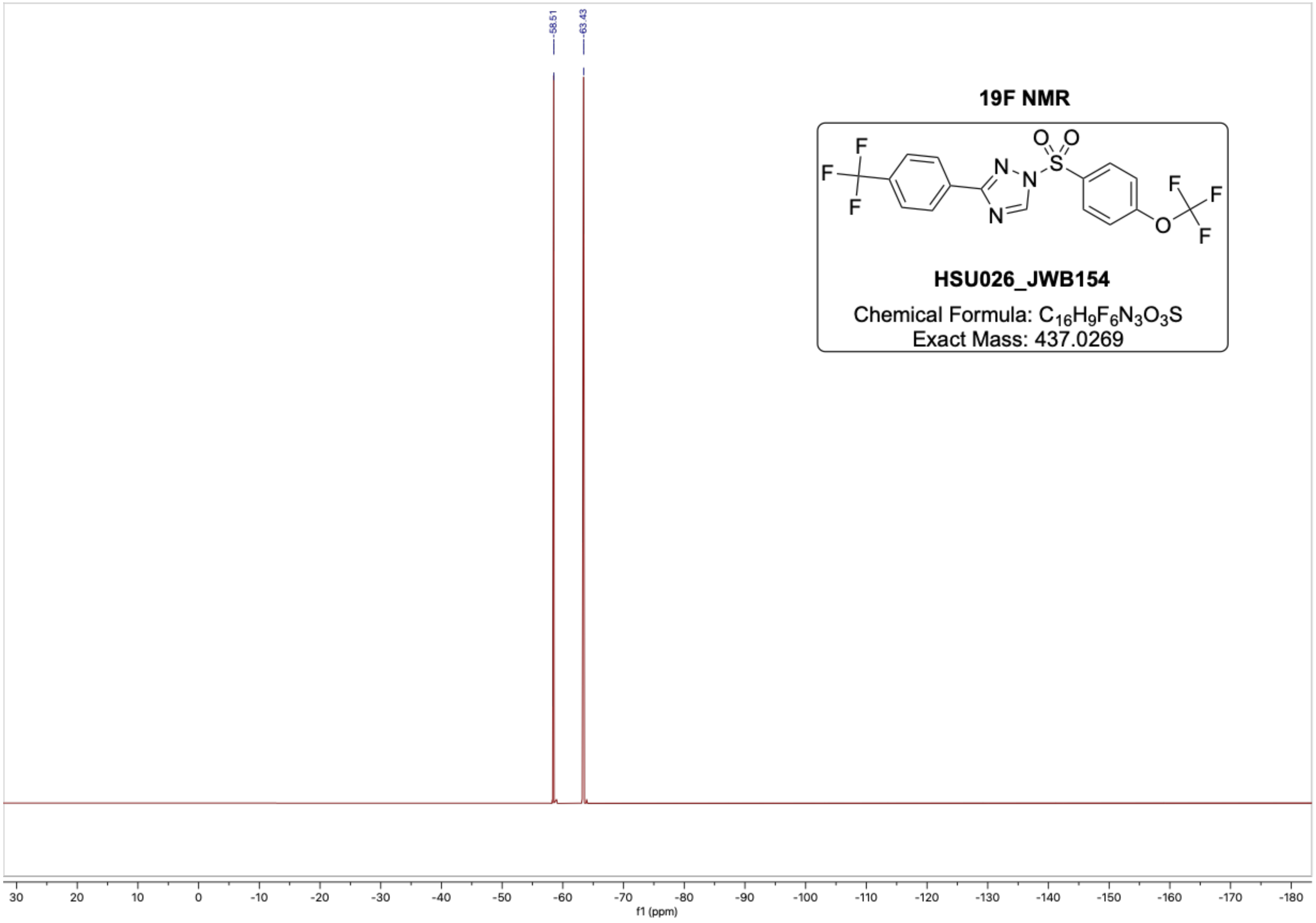

-58.51
-63.43
19F NMR
HSU026_JWB154
Chemical Formula: C16H9F6N3O3S
Exact Mass: 437.0269
30 20 10 0 -10 -20 -30 -40 -50 -60 -70 -80 -90 -100 -110 -120 -130 -140 -150 -160 -170 -180
f1 (ppm)

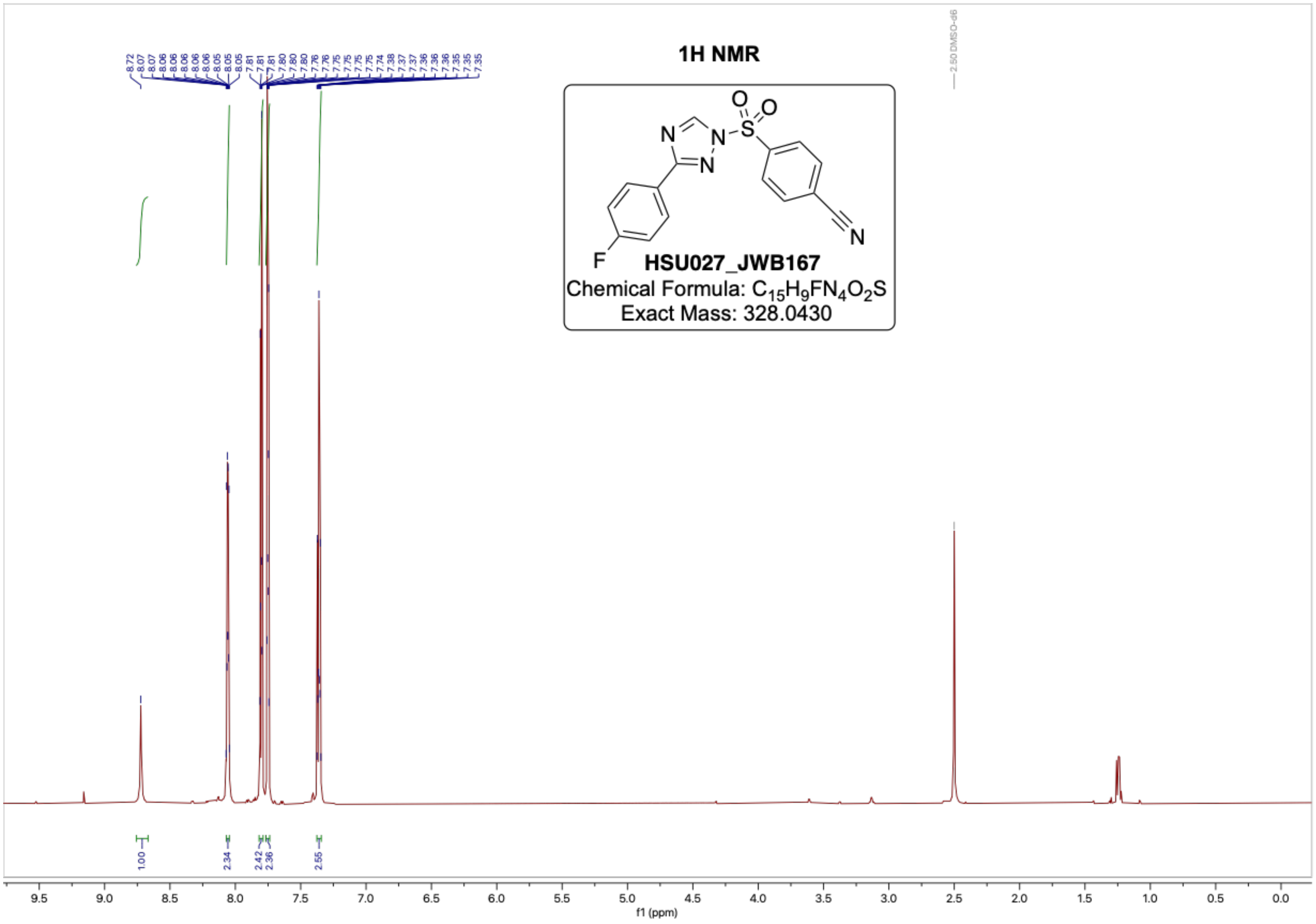
1H NMR
HSU027_JWB167
Chemical Formula: C15H9FN4O2S
Exact Mass: 328.0430
f1 (ppm)
2.50 DMSO-d6

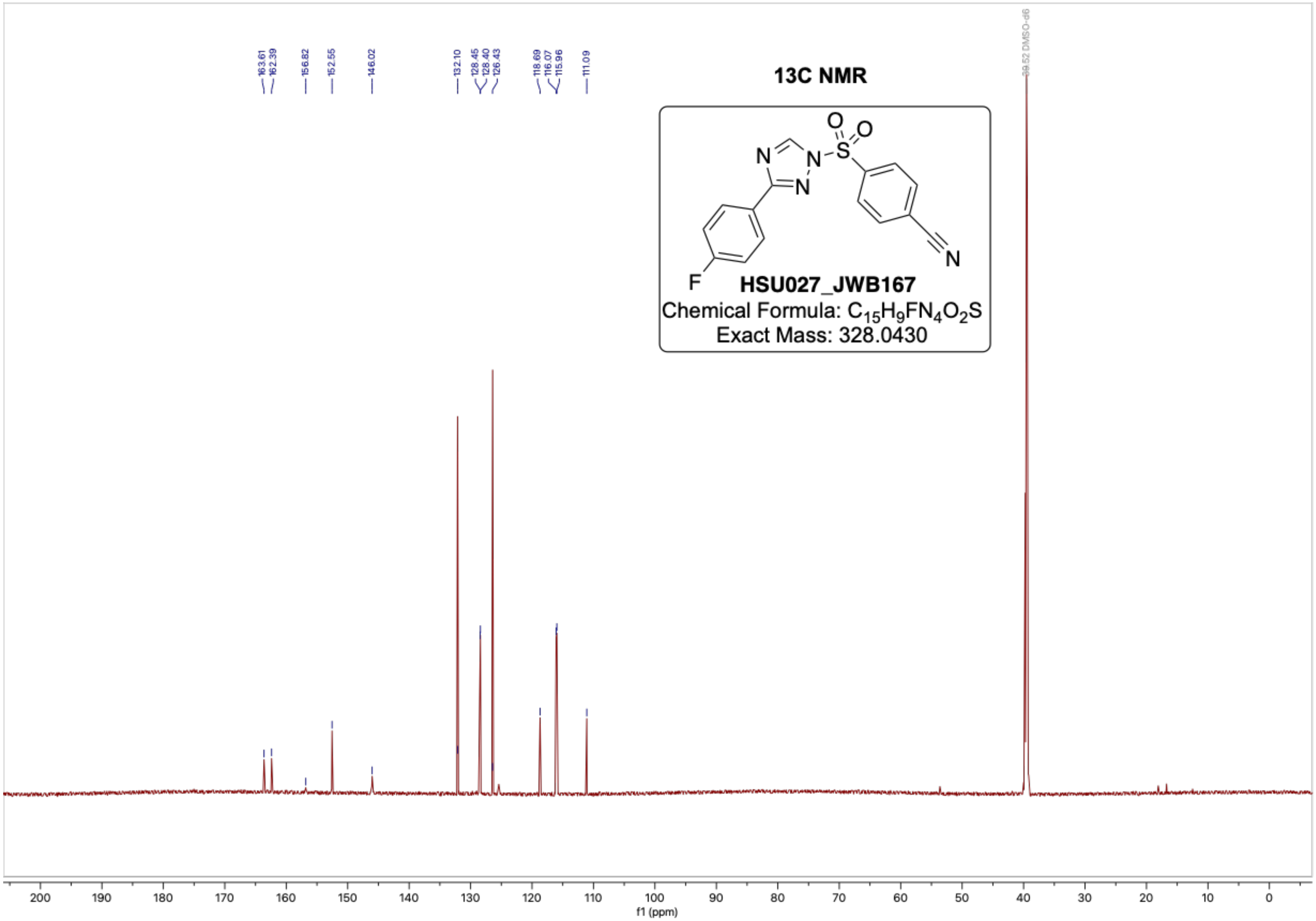
13C NMR
HSU027_JWB167
Chemical Formula: C15H9FN4O2S
Exact Mass: 328.0430
163.61
162.39
156.82
152.55
146.02
132.10
128.45
128.40
126.43
118.69
116.07
115.96
111.09
39.52 DMSO-d6
f1 (ppm)

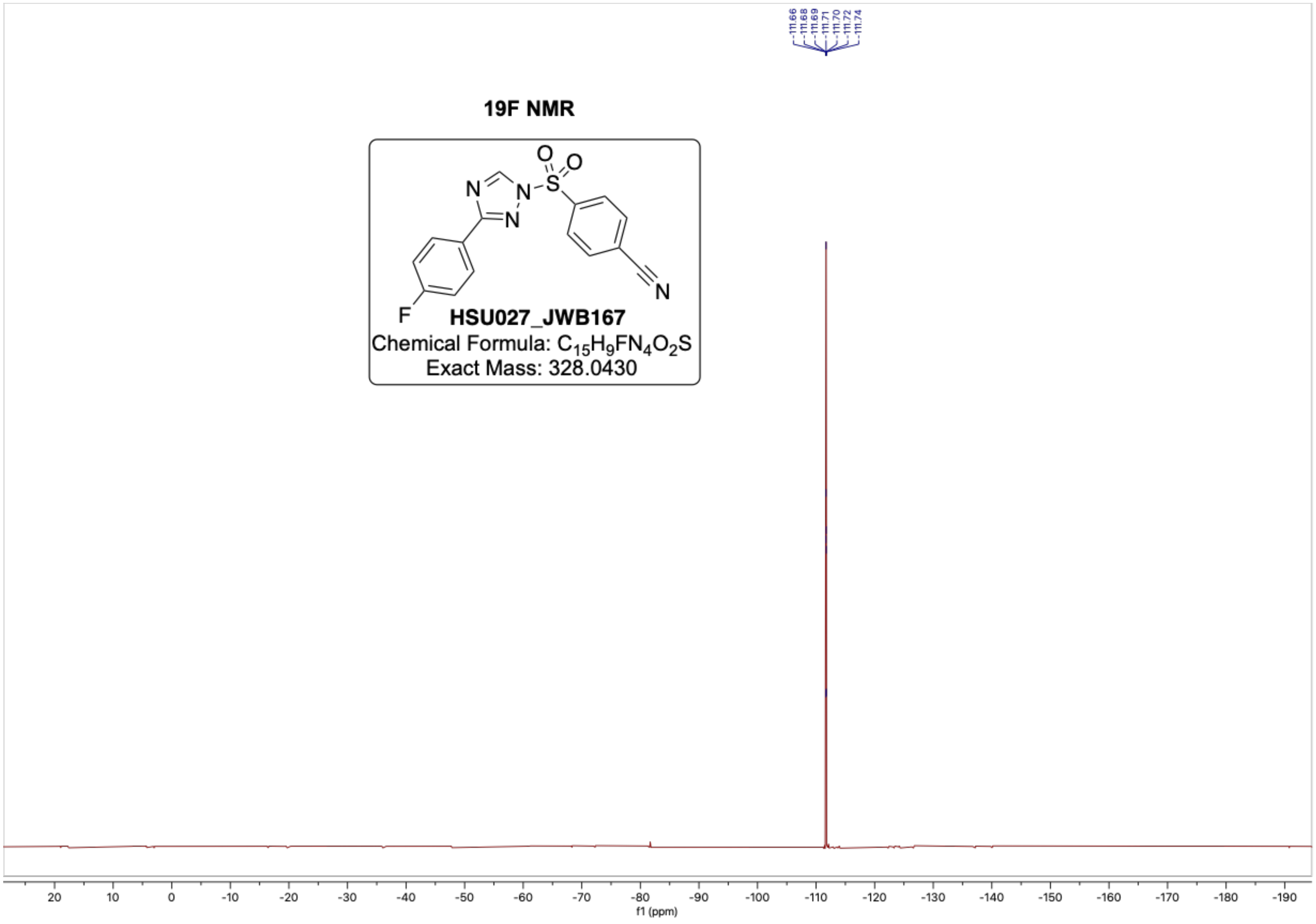
-111.66
-111.68
-111.69
-111.71
-111.70
-111.72
-111.74
19F NMR
HSU027_JWB167
Chemical Formula: $C_{15}H_9FN_4O_2S$
Exact Mass: 328.0430
20 10 0 -10 -20 -30 -40 -50 -60 -70 -80 -90 -100 -110 -120 -130 -140 -150 -160 -170 -180 -190
f1 (ppm)

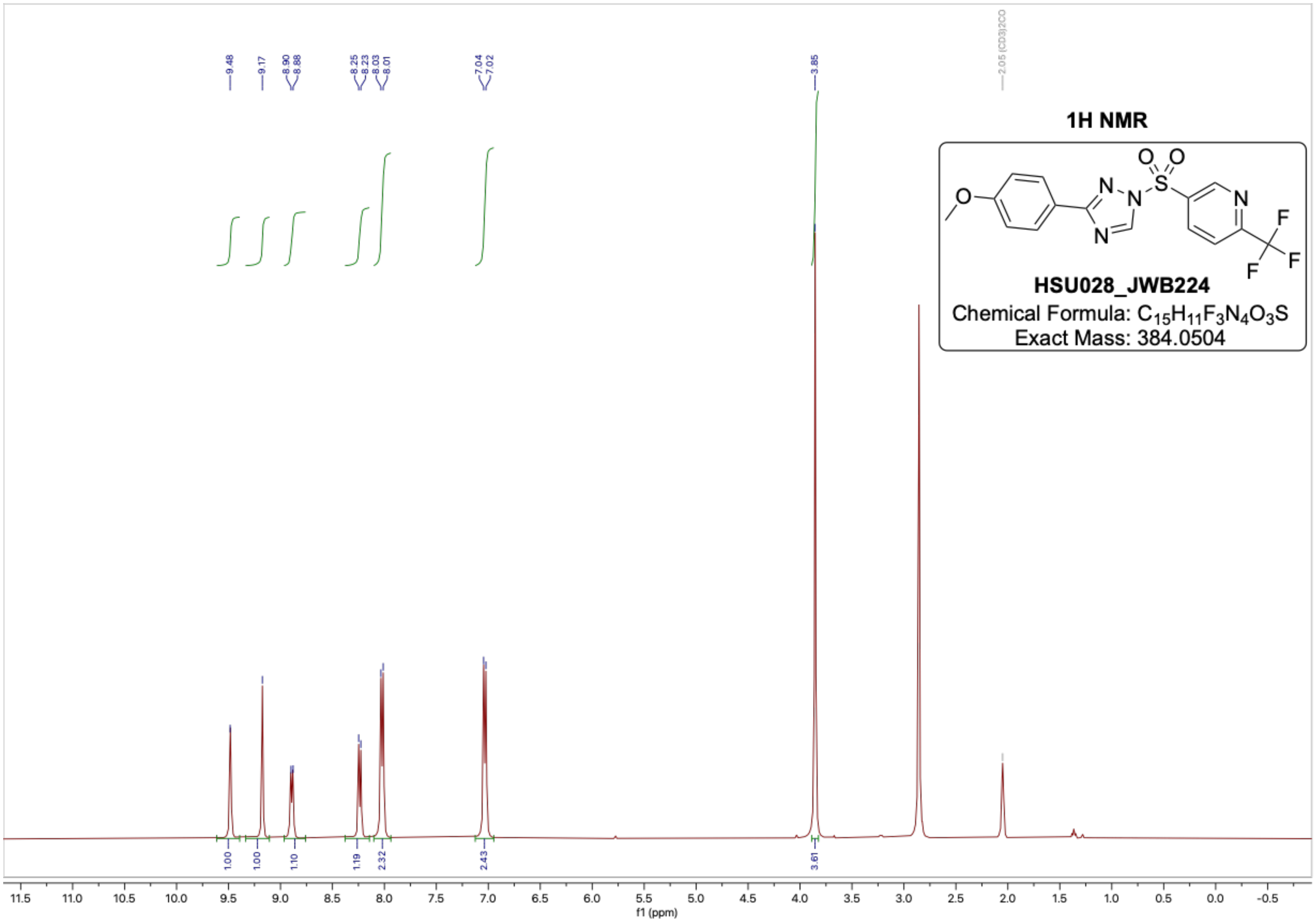
1H NMR
HSU028_JWB224
Chemical Formula: C15H11F3N4O3S
Exact Mass: 384.0504
9.48
9.17
8.90
8.88
8.25
8.23
8.03
8.01
7.04
7.02
3.85
2.05 (CD3)2CO
1.00
1.00
1.10
1.19
2.32
2.43
3.61
f1 (ppm)

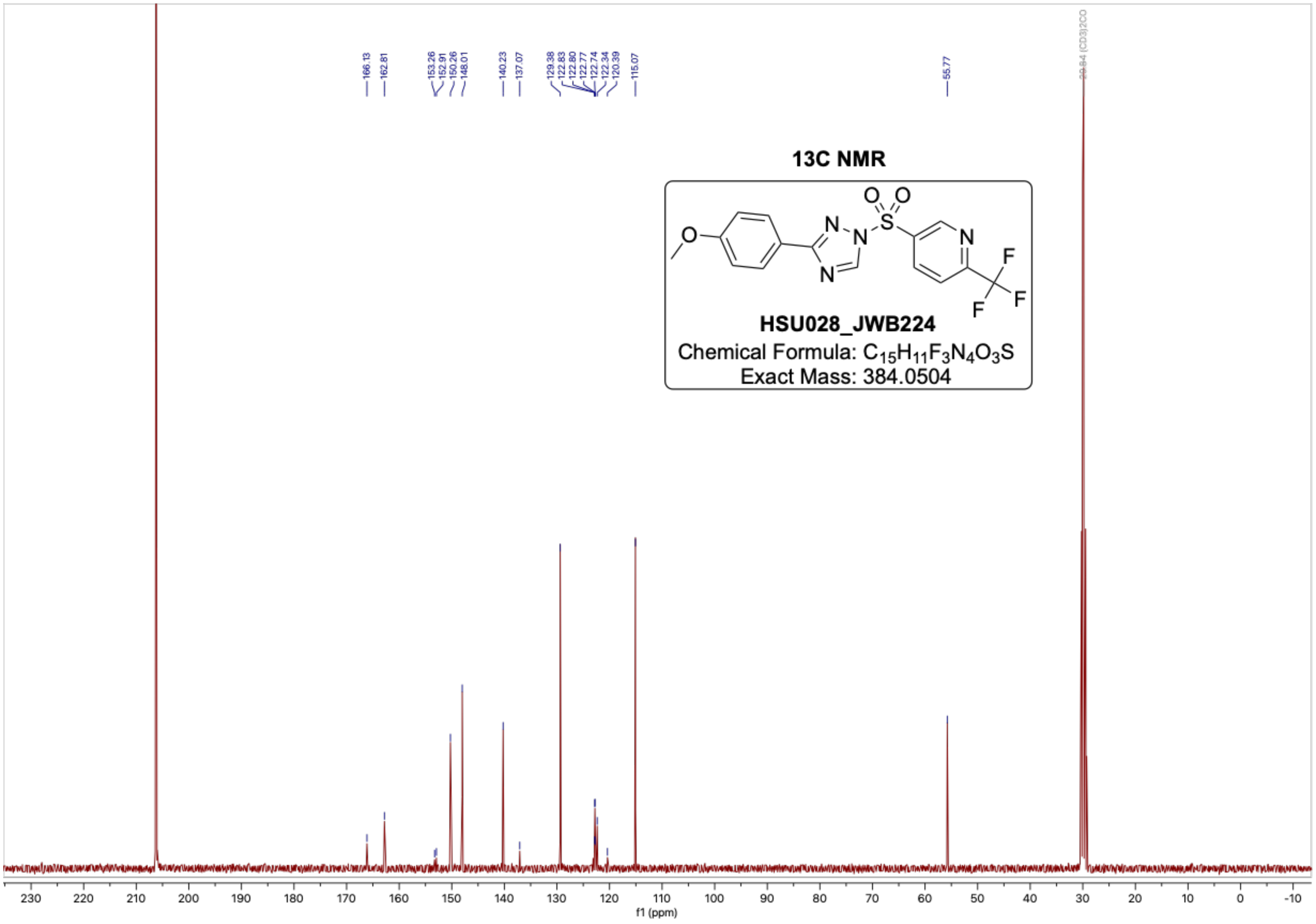

166.13
162.81
153.26
152.91
150.26
148.01
140.23
137.07
129.38
122.83
122.80
122.77
122.74
122.34
120.39
115.07
55.77
29.84 (CD3)2CO
13C NMR
O
N
S
F
HSU028_JWB224
Chemical Formula: $C_{15}H_{11}F_3N_4O_3S$
Exact Mass: 384.0504
230
220
210
200
190
180
170
160
150
140
130
120
110
100
90
80
70
60
50
40
30
20
10
0
-10
f1 (ppm)

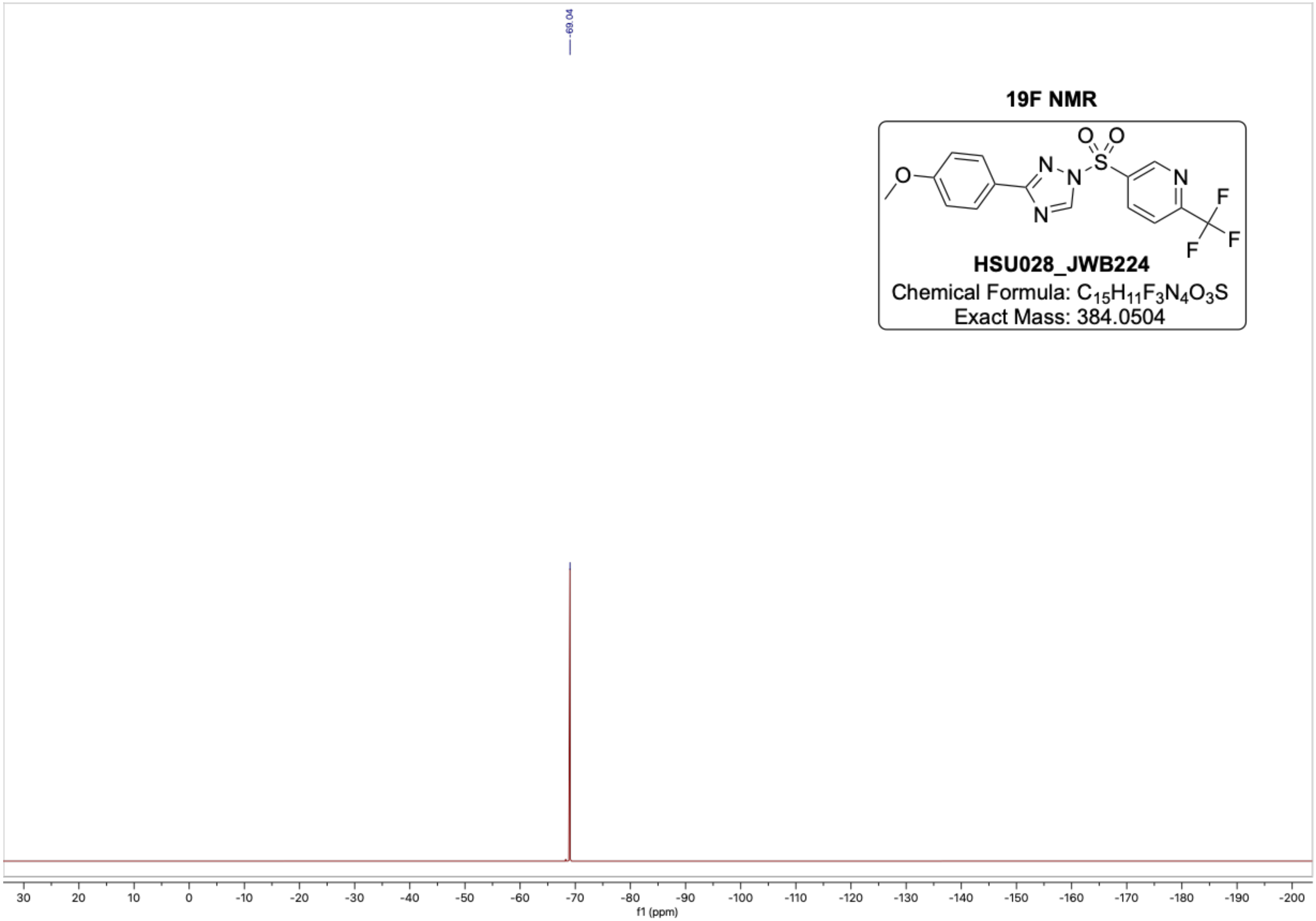

-69.04
19F NMR
HSU028_JWB224
Chemical Formula: $C_{15}H_{11}F_3N_4O_3S$
Exact Mass: 384.0504
30
20
10
0
-10
-20
-30
-40
-50
-60
-70
-80
-90
-100
-110
-120
-130
-140
-150
-160
-170
-180
-190
-200
f1 (ppm)

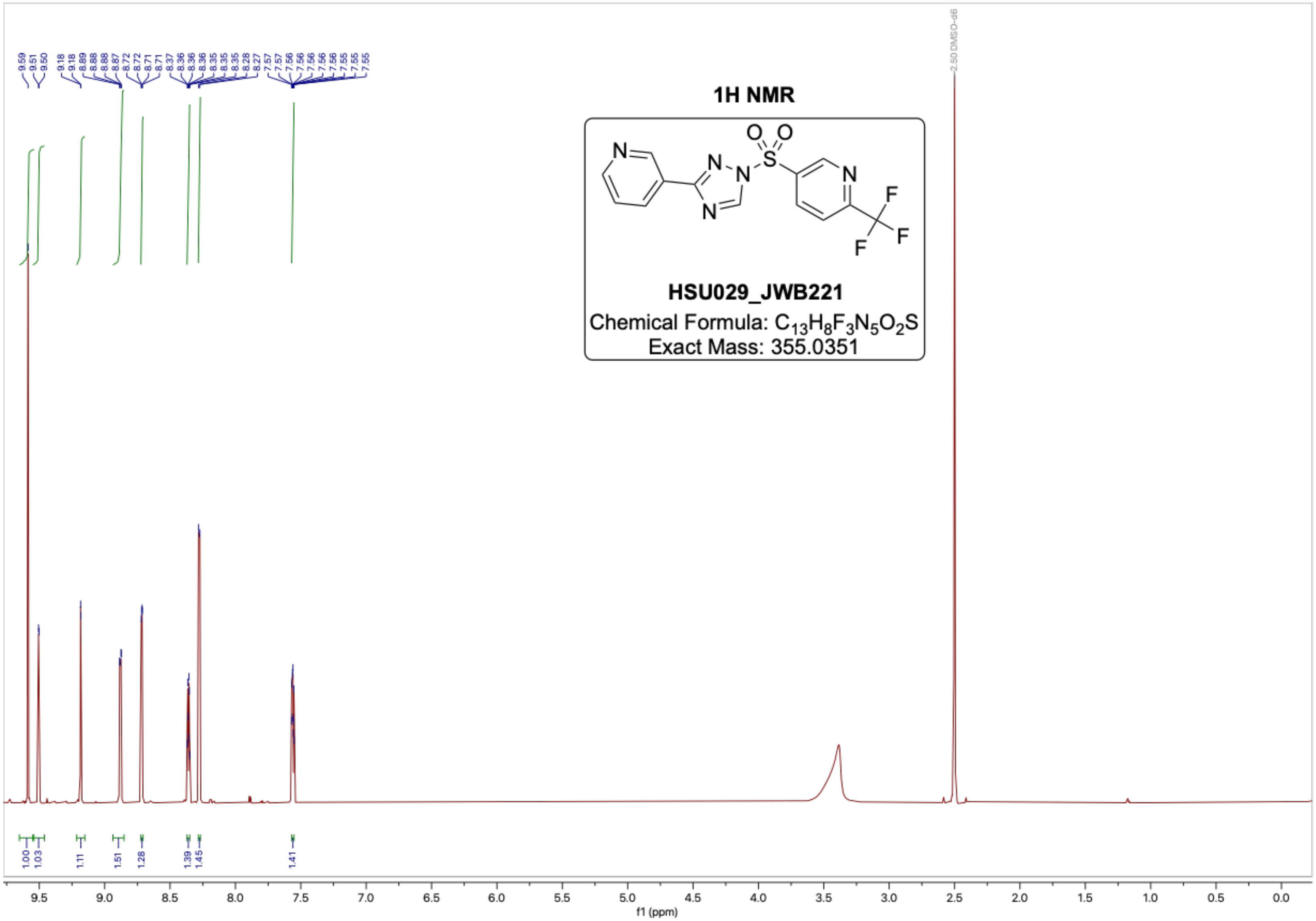
1H NMR
HSU029_JWB221
Chemical Formula: $C_{13}H_8F_3N_5O_2S$
Exact Mass: 355.0351
f1 (ppm)

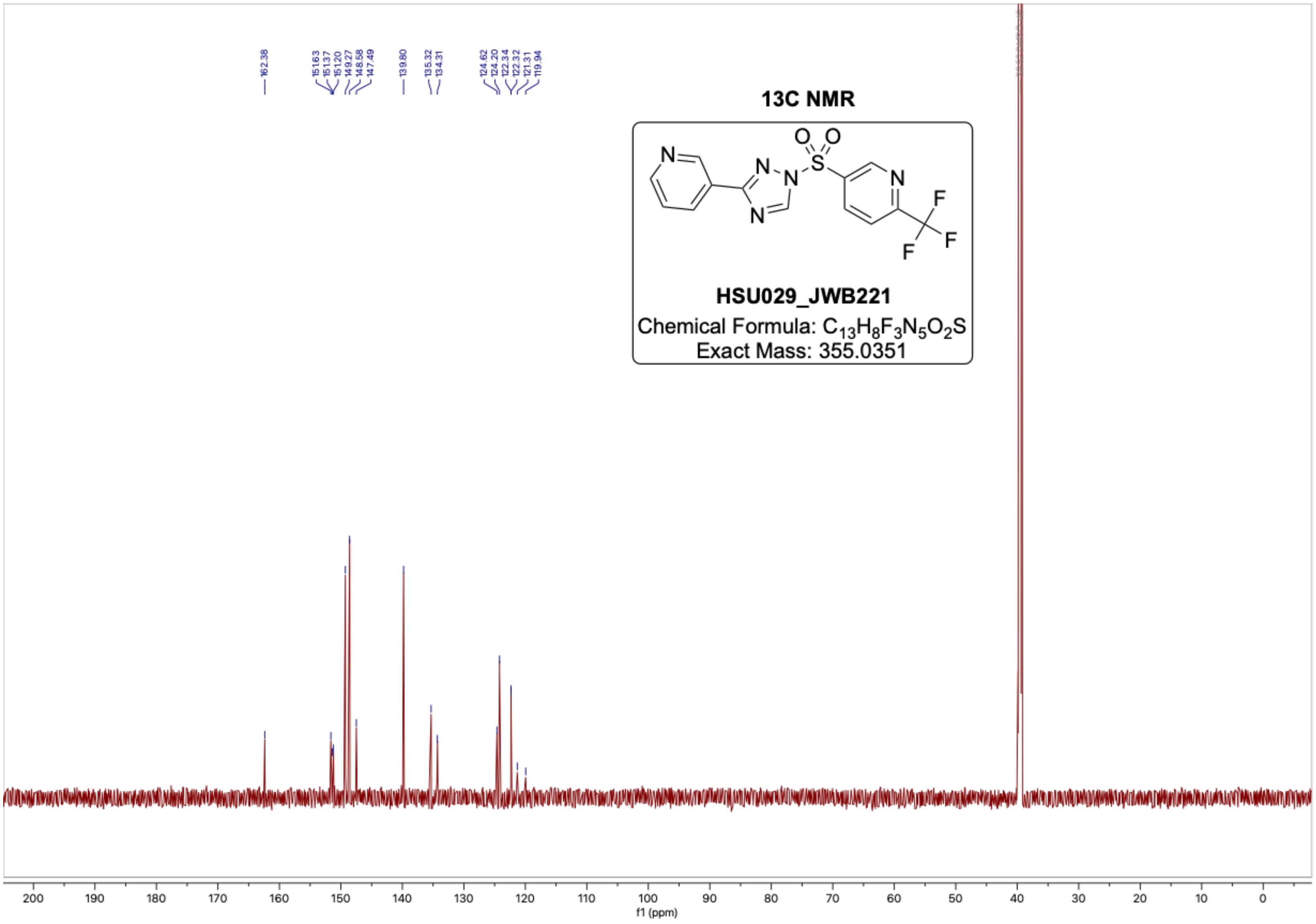
13C NMR
HSU029_JWB221
Chemical Formula: $C_{13}H_8F_3N_5O_2S$
Exact Mass: 355.0351
162.38
151.63
151.37
151.20
149.27
148.58
147.49
139.80
135.32
134.31
124.62
124.20
122.34
122.32
121.31
119.94
200 190 180 170 160 150 140 130 120 110 100 90 80 70 60 50 40 30 20 10 0
f1 (ppm)

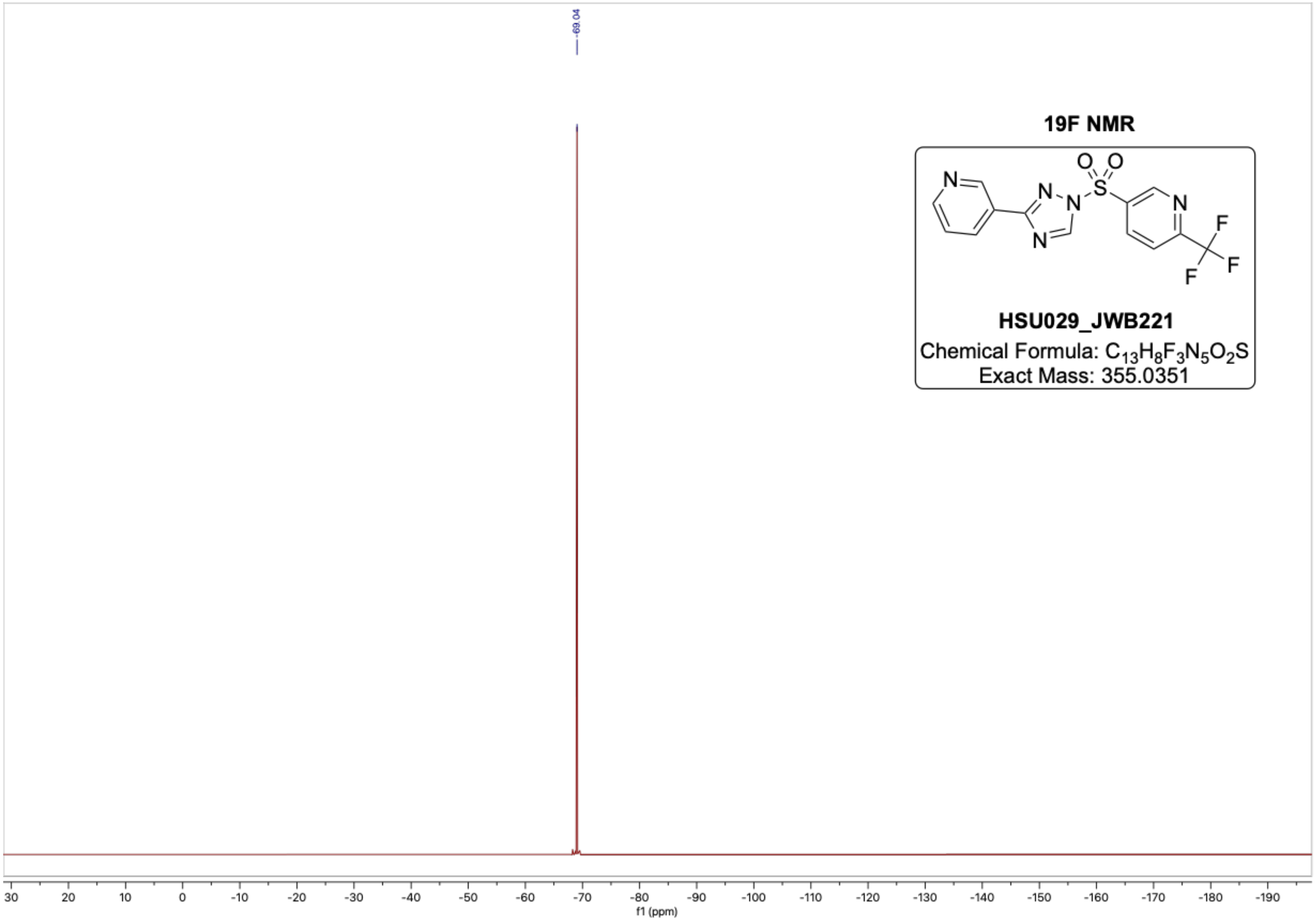
-69.04
19F NMR
HSU029_JWB221
Chemical Formula: $C_{13}H_8F_3N_5O_2S$
Exact Mass: 355.0351
30 20 10 0 -10 -20 -30 -40 -50 -60 -70 -80 -90 -100 -110 -120 -130 -140 -150 -160 -170 -180 -190
f1 (ppm)

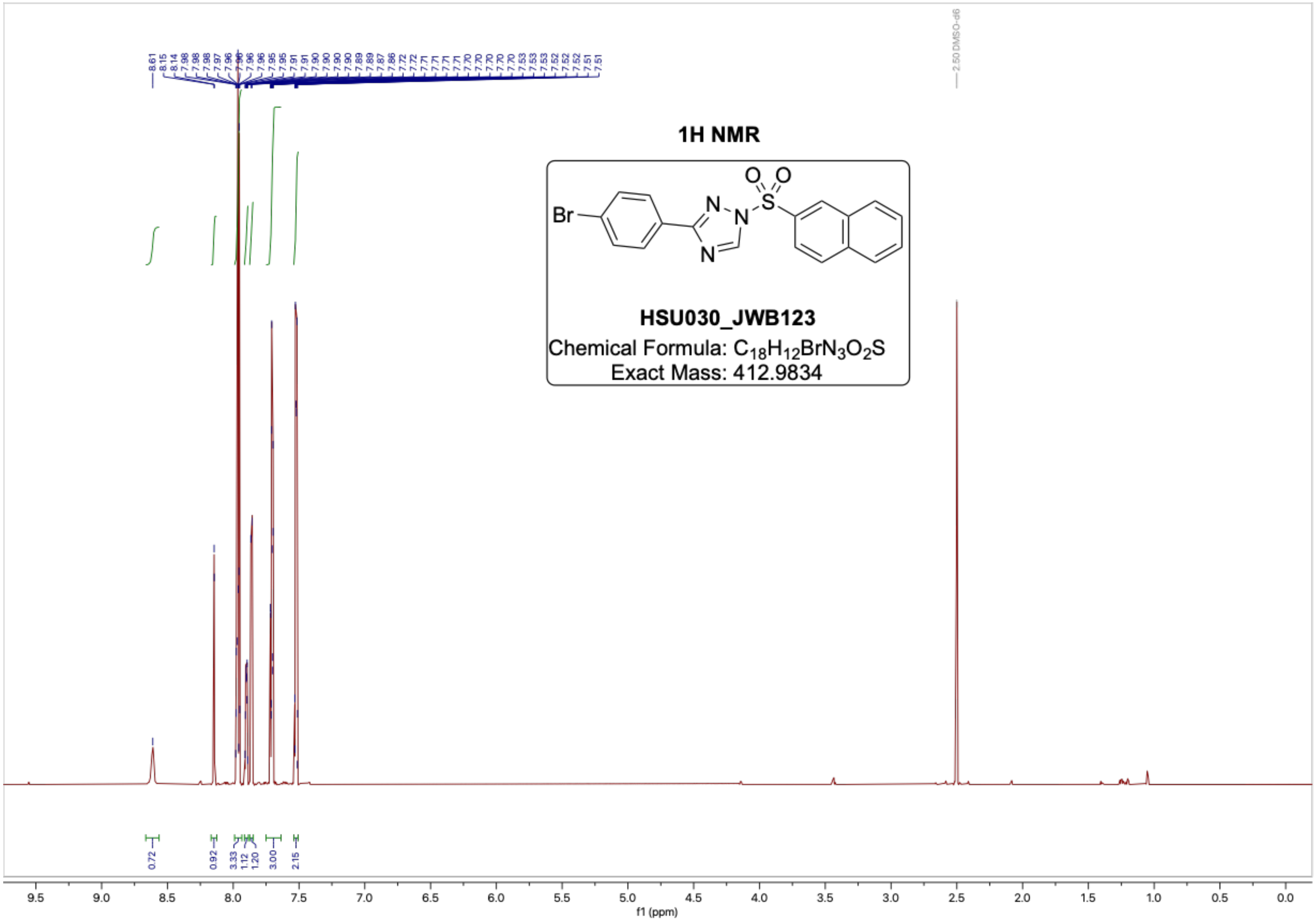

1H NMR
HSU030_JWB123
Chemical Formula: C18H12BrN3O2S
Exact Mass: 412.9834
2.50 DMSO-d6
f1 (ppm)

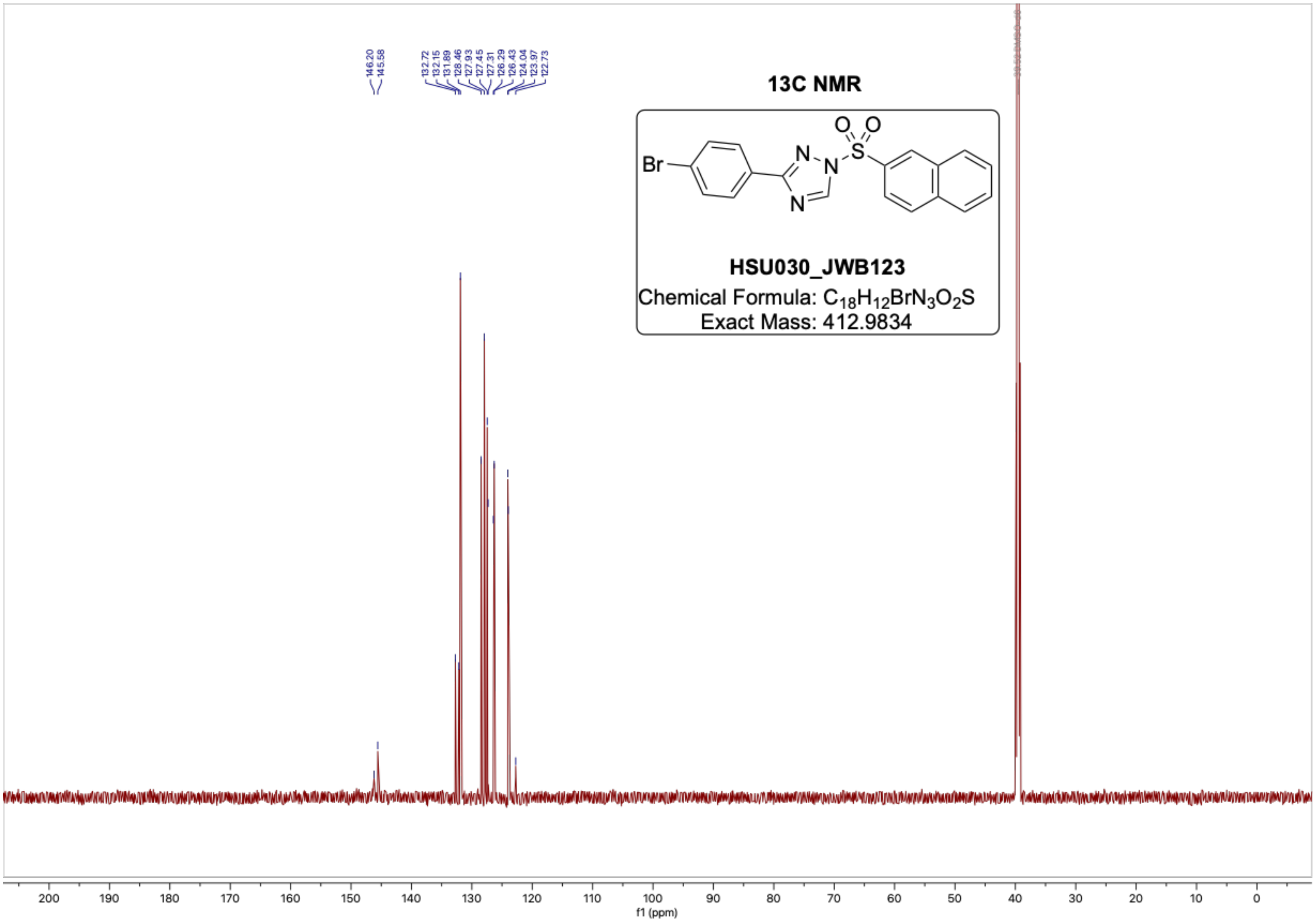
146.20
145.58
132.72
132.15
131.89
128.46
127.93
127.45
127.31
126.29
126.43
124.04
123.97
122.73
13C NMR
Br
N
N
N
S
O
O
HSU030_JWB123
Chemical Formula: $C_{18}H_{12}BrN_3O_2S$
Exact Mass: 412.9834
200
190
180
170
160
150
140
130
120
110
100
90
80
70
60
50
40
30
20
10
0
f1 (ppm)

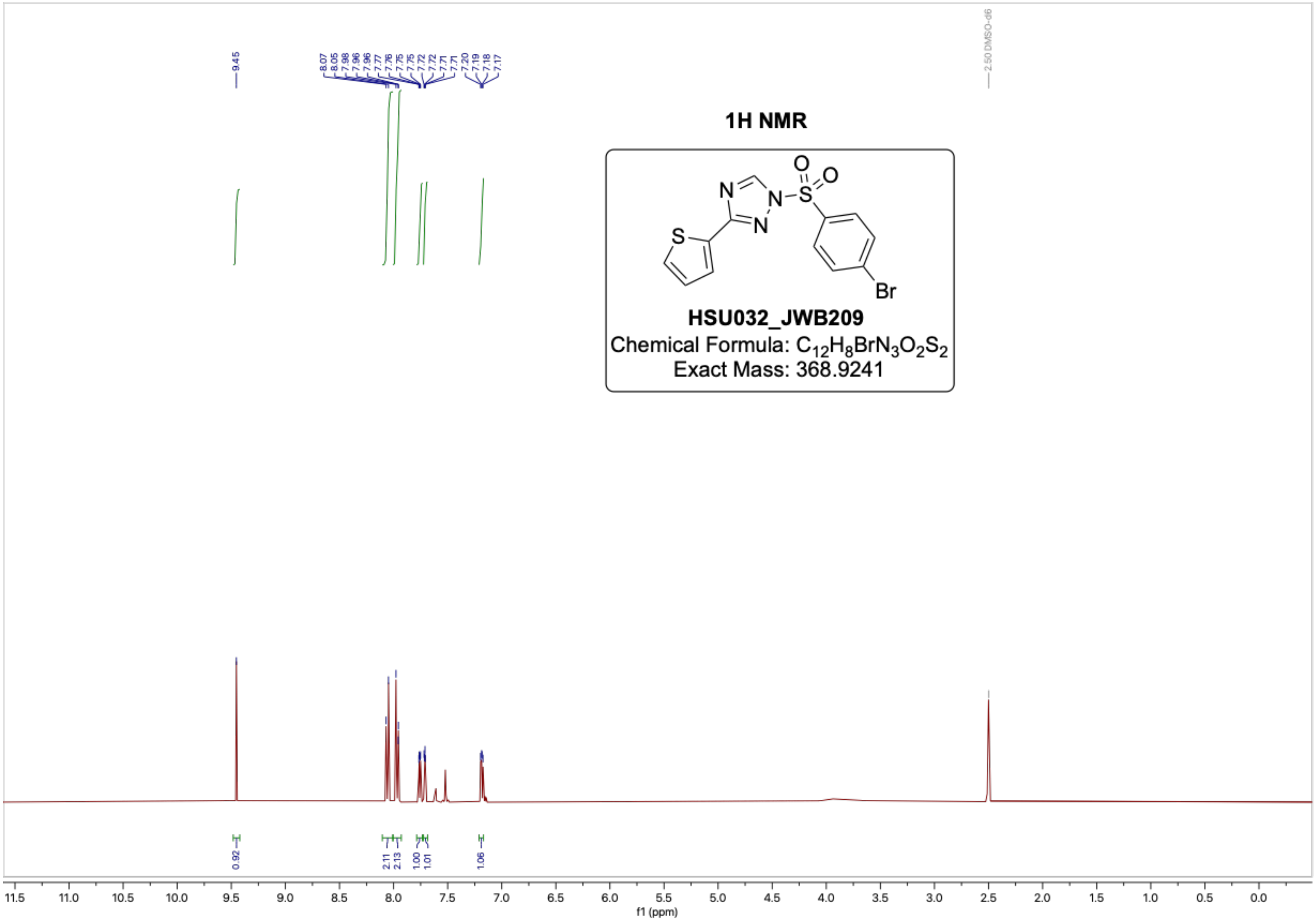
1H NMR
HSU032_JWB209
Chemical Formula: $C_{12}H_8BrN_3O_2S_2$
Exact Mass: 368.9241
9.45
8.07 8.05 7.98 7.96 7.77 7.76 7.75 7.75 7.72 7.72 7.71 7.71 7.20 7.19 7.18 7.17
2.50 DMSO-d6
0.92 2.11 2.13 1.00 1.01 1.06
f1 (ppm)

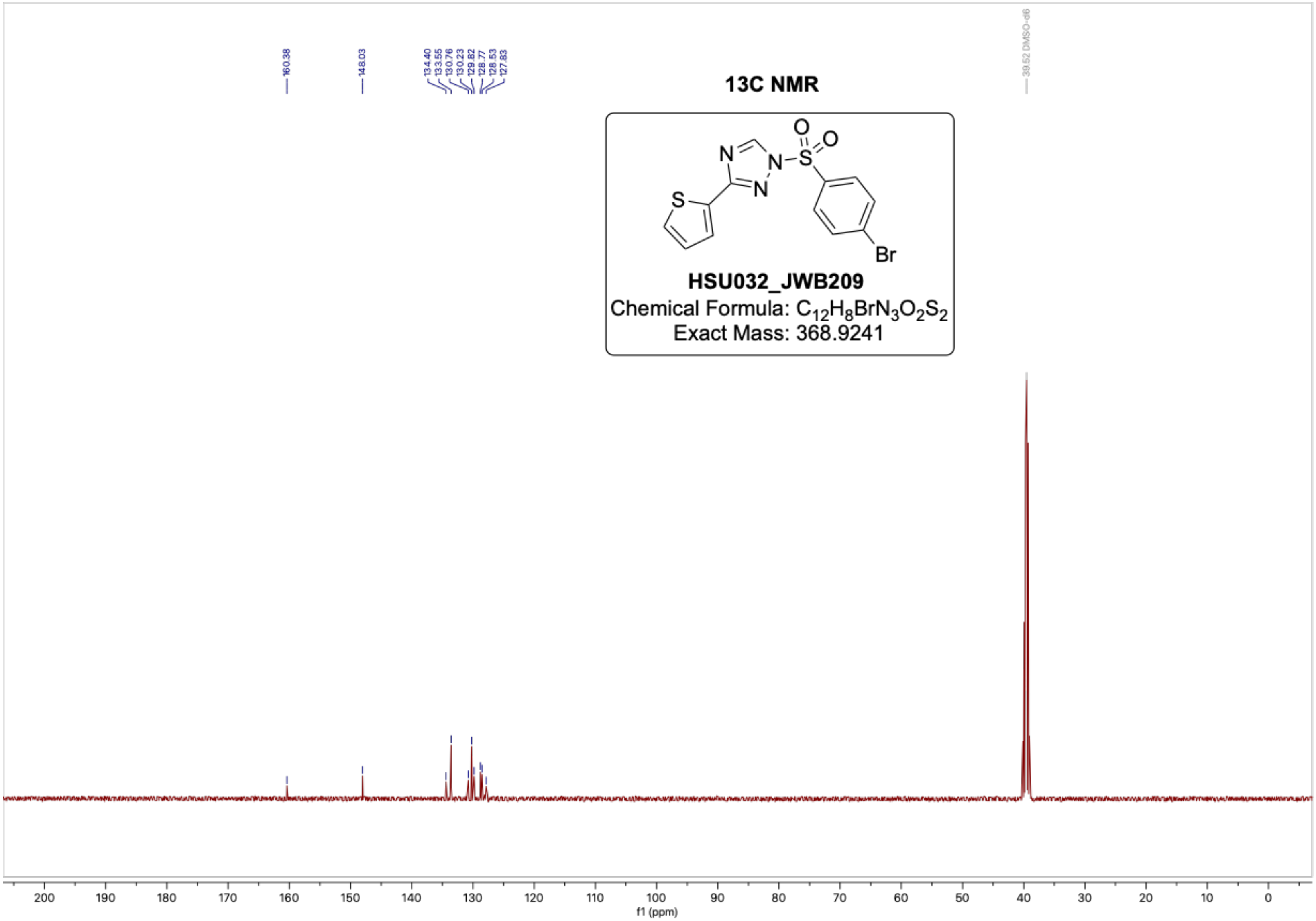

13C NMR
HSU032_JWB209
Chemical Formula: C12H8BrN3O2S2
Exact Mass: 368.9241
160.38
148.03
134.40
133.55
130.76
130.23
129.82
128.77
128.53
127.83
39.52 DMSO-d6
f1 (ppm)

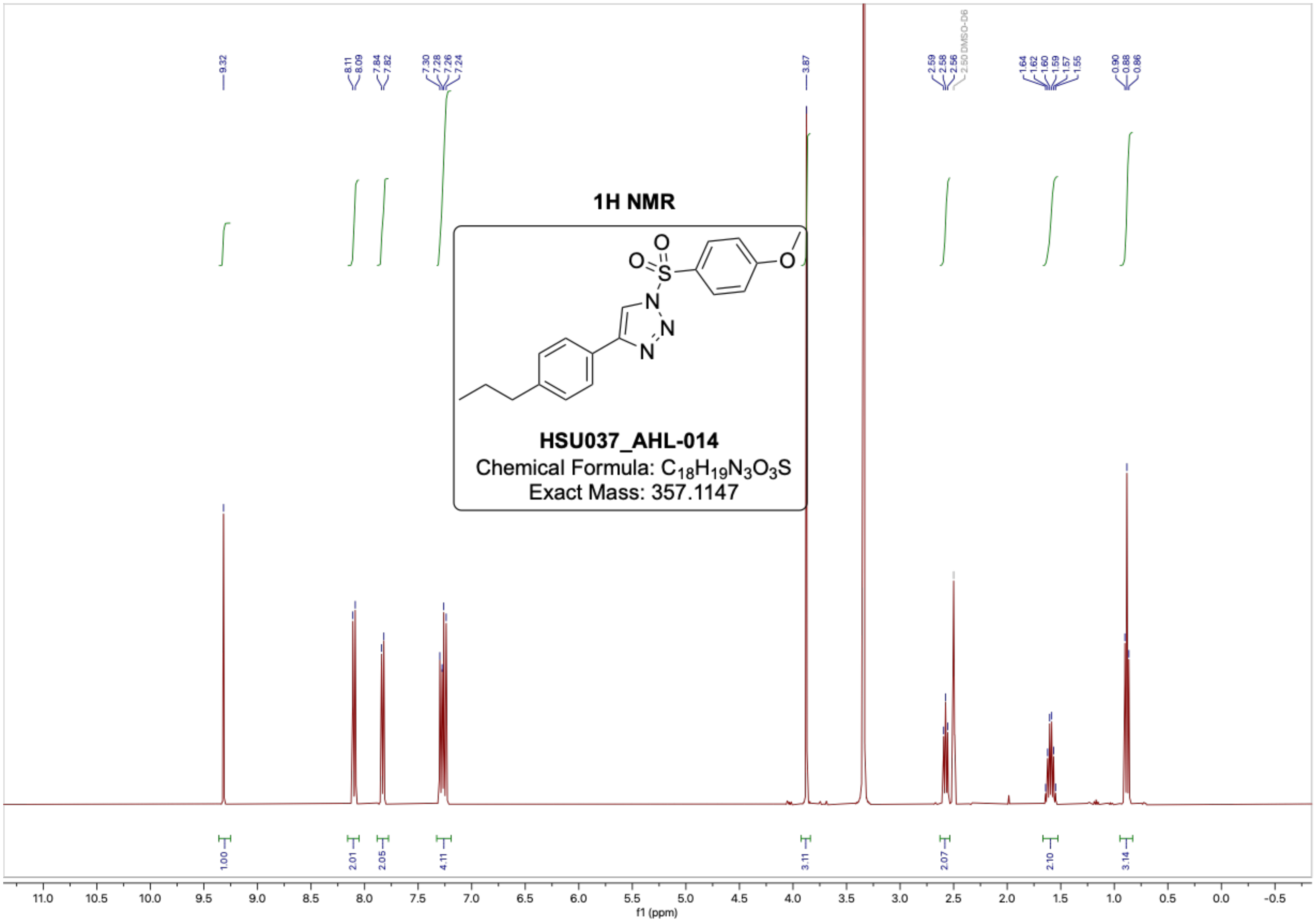
1H NMR
HSU037_AHL-014
Chemical Formula: C18H19N3O3S
Exact Mass: 357.1147
9.32
8.11
8.09
7.84
7.82
7.30
7.28
7.26
7.24
3.87
2.59
2.58
2.56
2.50 DMSO-D6
1.64
1.62
1.60
1.59
1.57
1.55
0.90
0.88
0.86
1.00
2.01
2.05
4.11
3.11
2.07
2.10
3.14
f1 (ppm)

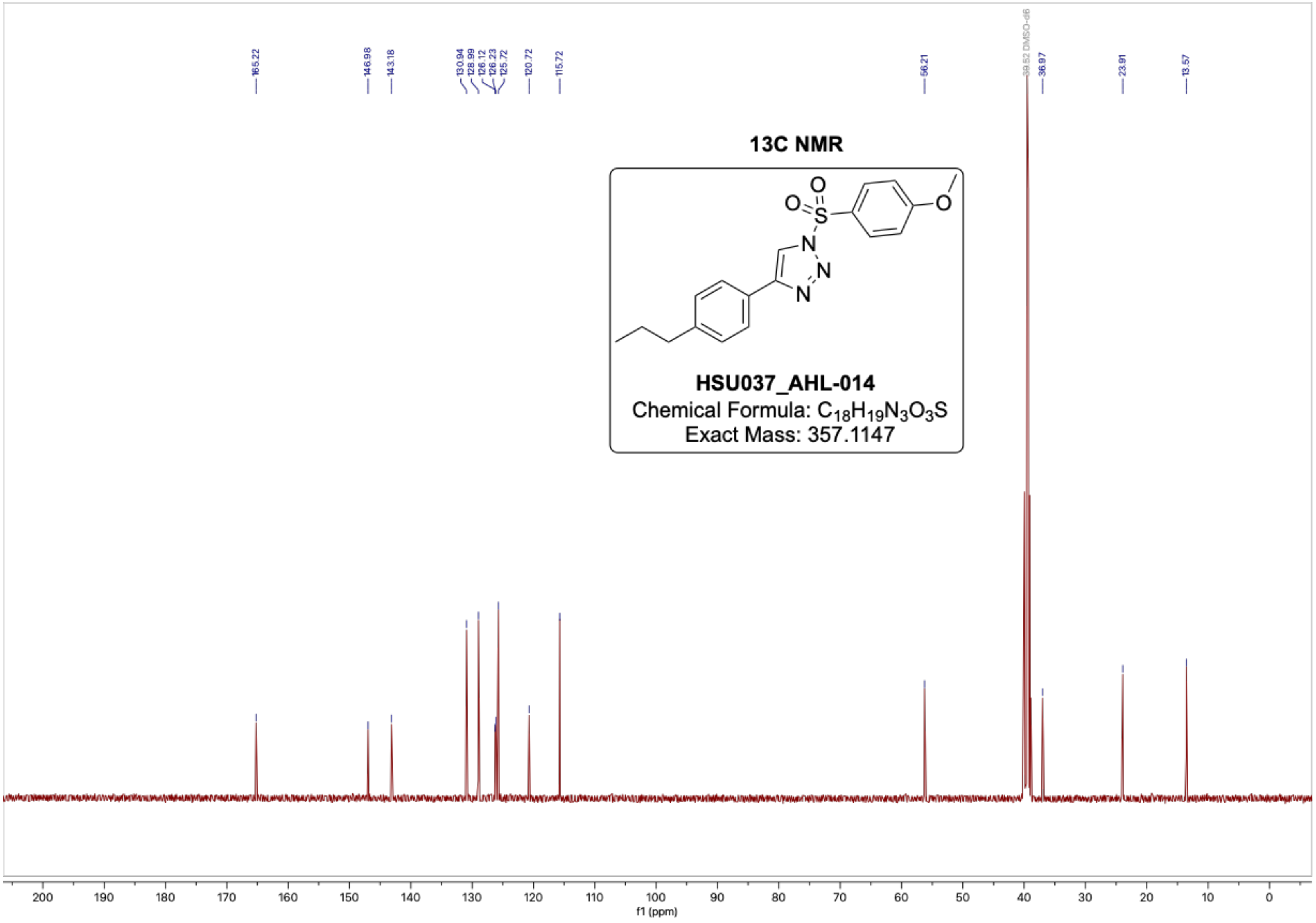
165.22
146.98
143.18
130.94
128.99
126.12
126.23
125.72
120.72
115.72
56.21
39.52 DMSO-d6
36.97
23.91
13.57
13C NMR
HSU037_AHL-014
Chemical Formula: $C_{18}H_{19}N_3O_3S$
Exact Mass: 357.1147
f1 (ppm)
200
190
180
170
160
150
140
130
120
110
100
90
80
70
60
50
40
30
20
10
0

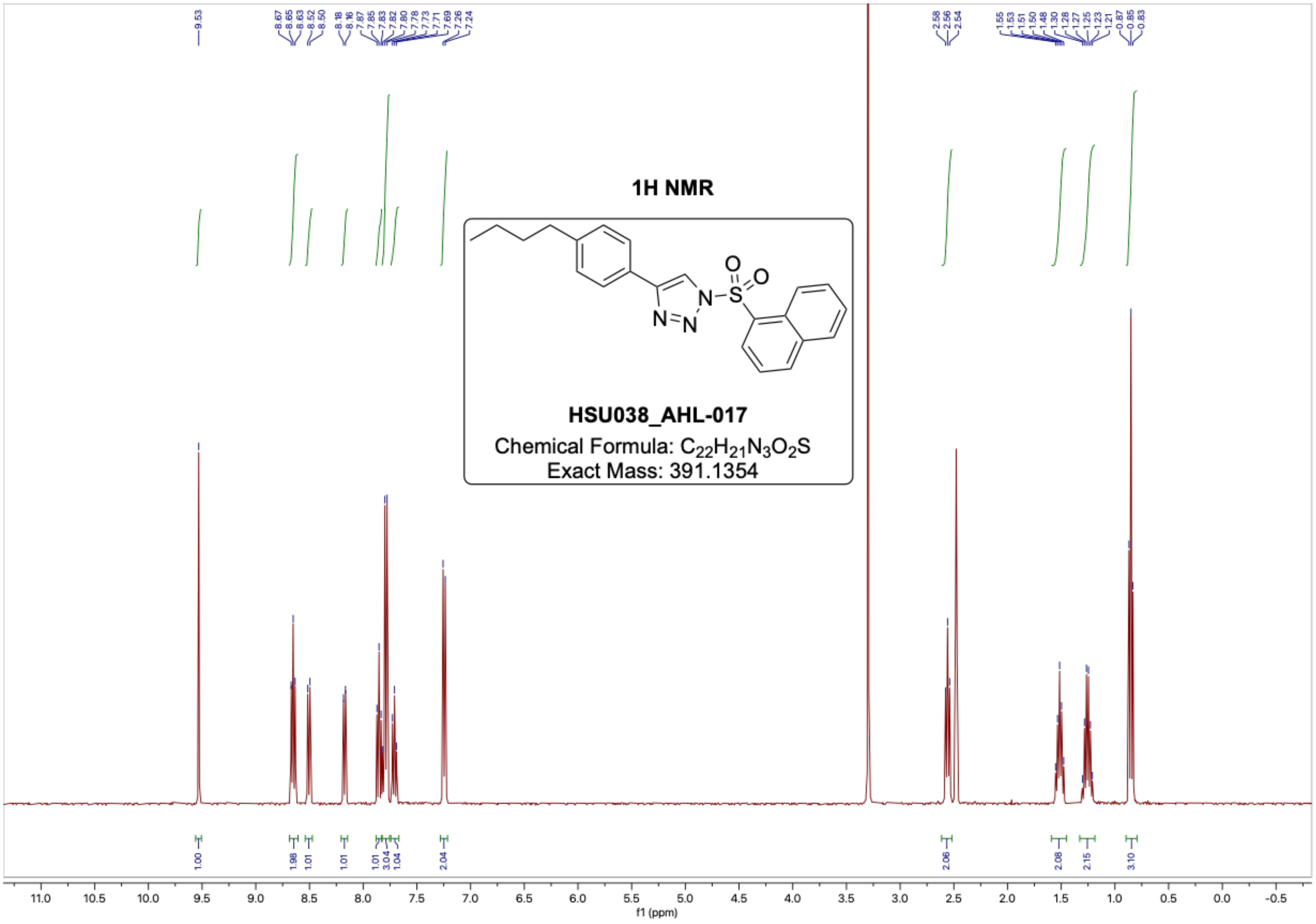
1H NMR
HSU038_AHL-017
Chemical Formula: C22H21N3O2S
Exact Mass: 391.1354
f1 (ppm)

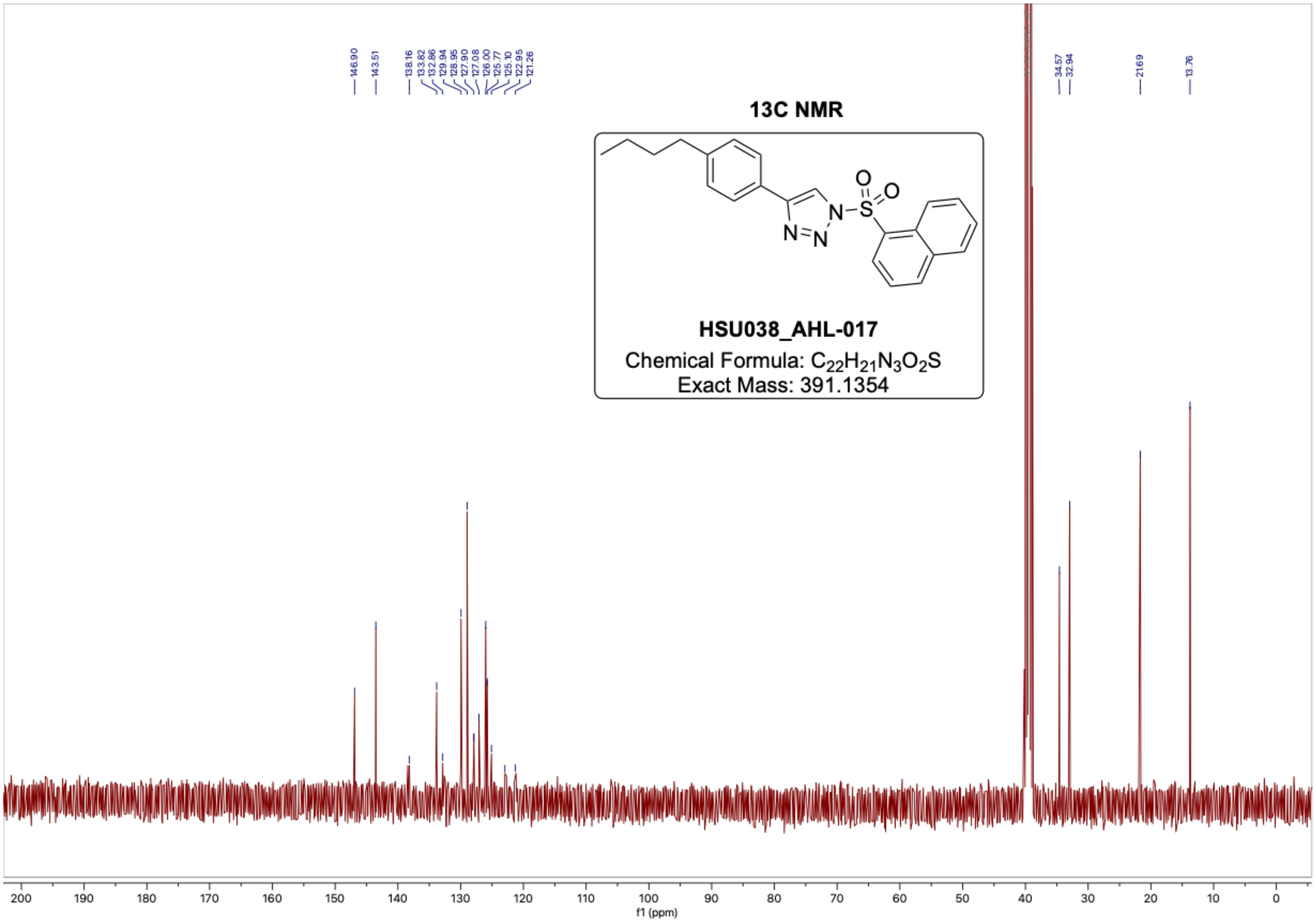

146.90
143.51
138.16
133.82
132.86
129.94
128.95
127.90
127.08
126.00
125.77
125.10
122.95
121.26
34.57
32.94
21.69
13.76
13C NMR
HSU038_AHL-017
Chemical Formula: C22H21N3O2S
Exact Mass: 391.1354
f1 (ppm)

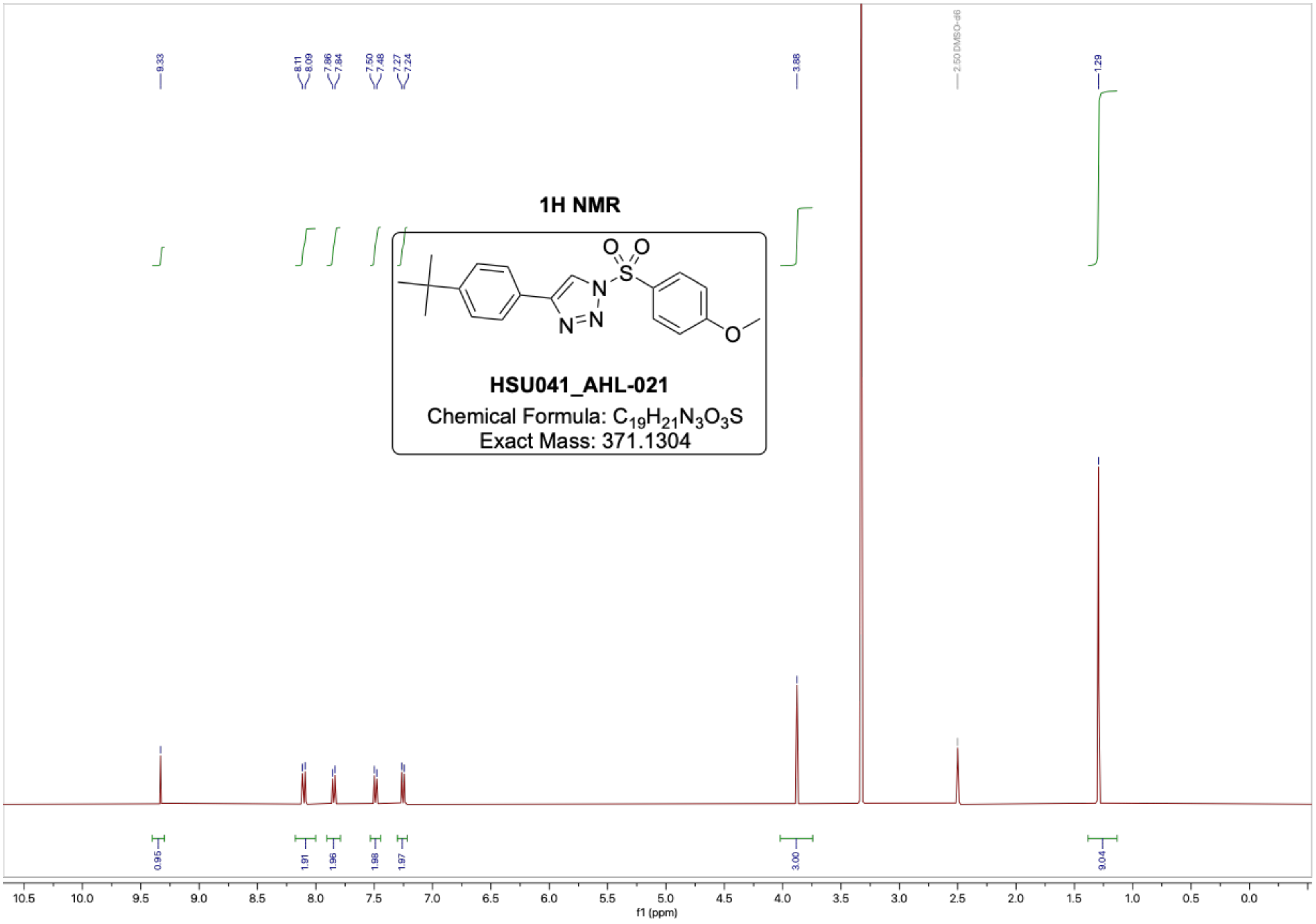
9.33
8.11
8.09
7.86
7.84
7.50
7.48
7.27
7.24
3.88
2.50 DMSO-d6
1.29
1H NMR
HSU041_AHL-021
Chemical Formula: C19H21N3O3S
Exact Mass: 371.1304
0.95
1.91
1.96
1.98
1.97
3.00
9.04
10.5
10.0
9.5
9.0
8.5
8.0
7.5
7.0
6.5
6.0
5.5
5.0
4.5
4.0
3.5
3.0
2.5
2.0
1.5
1.0
0.5
0.0
f1 (ppm)

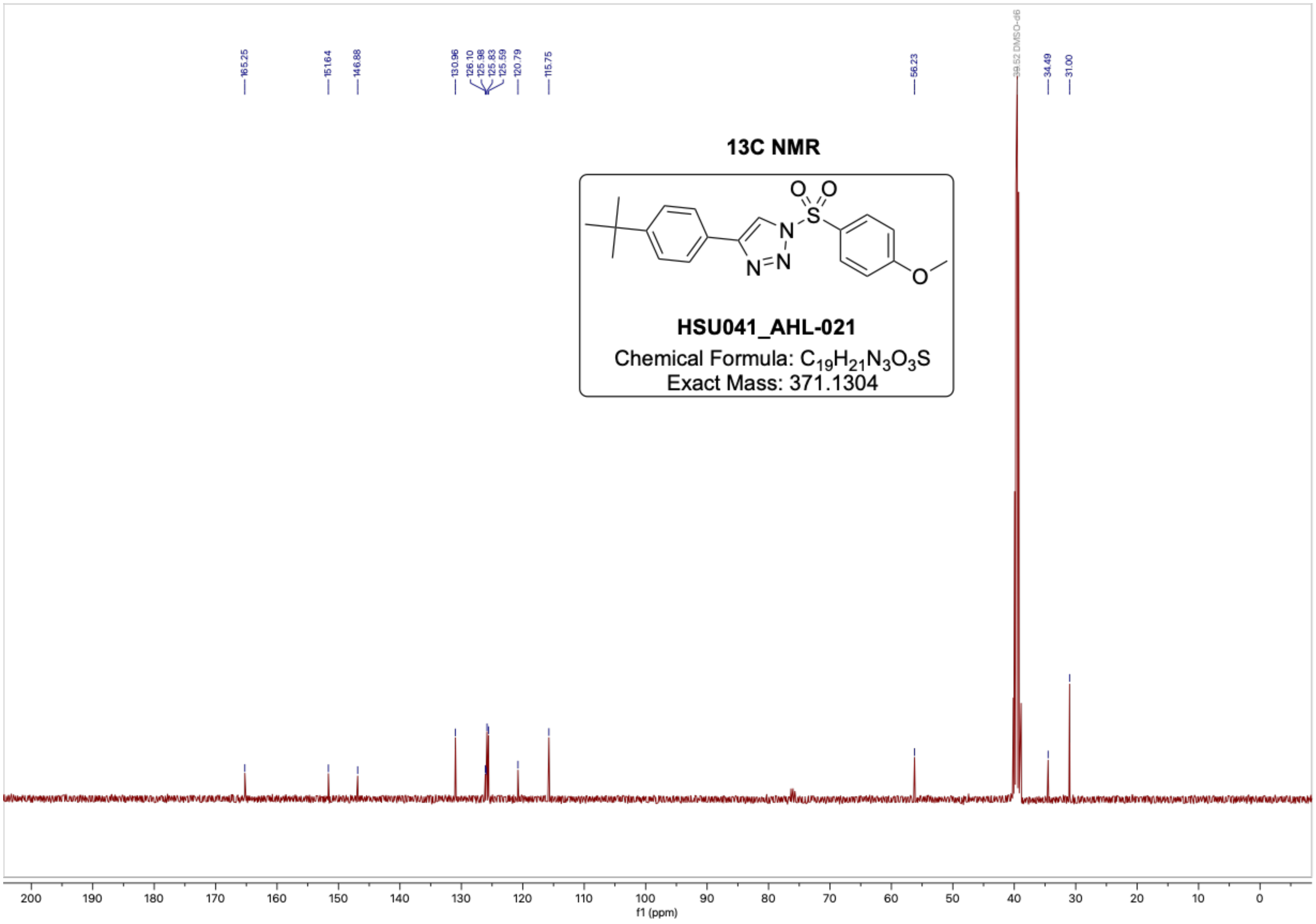
13C NMR
HSU041_AHL-021
Chemical Formula: C19H21N3O3S
Exact Mass: 371.1304
165.25
151.64
146.88
130.96
126.10
125.98
125.83
125.59
120.79
115.75
56.23
39.52 DMSO-d6
34.49
31.00
f1 (ppm)

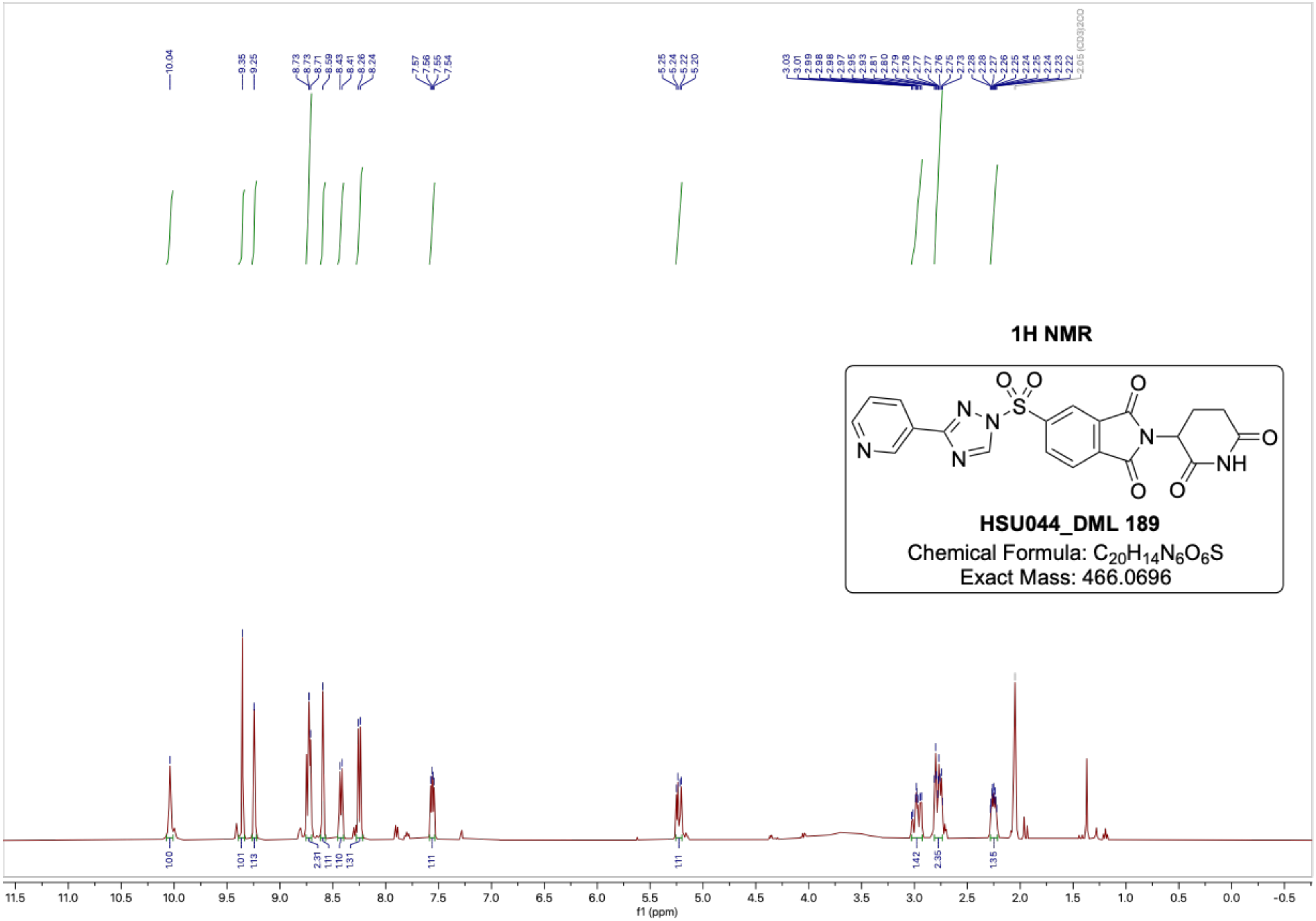
1H NMR
HSU044_DML 189
Chemical Formula: $C_{20}H_{14}N_6O_6S$
Exact Mass: 466.0696
f1 (ppm)

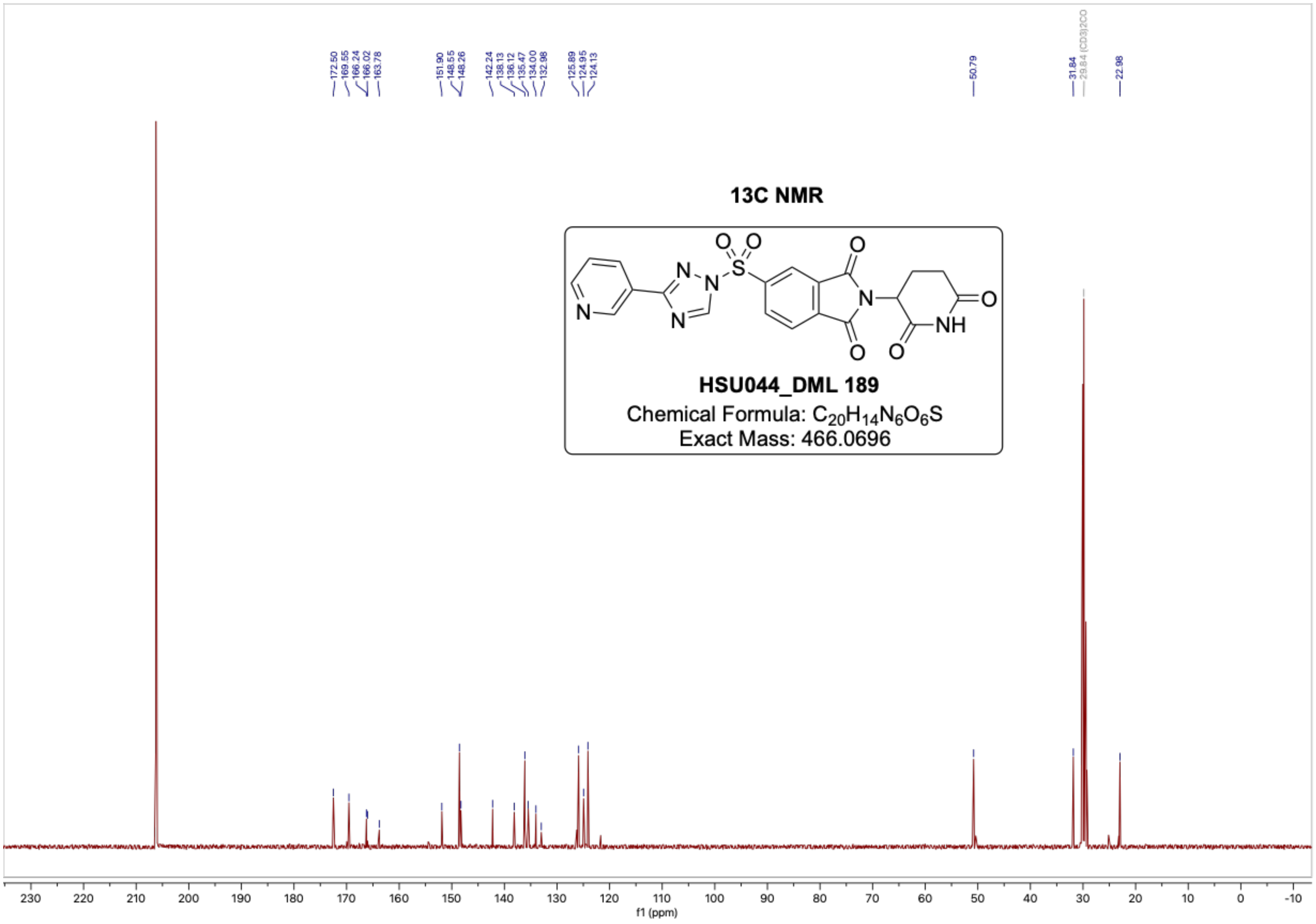

172.50
169.55
166.24
166.02
163.78
151.90
148.55
148.26
142.24
138.13
136.12
135.47
134.00
132.98
125.89
124.95
124.13
50.79
31.84
29.84 (CD3)2CO
22.98
13C NMR
HSU044_DML 189
Chemical Formula: C20H14N6O6S
Exact Mass: 466.0696
f1 (ppm)

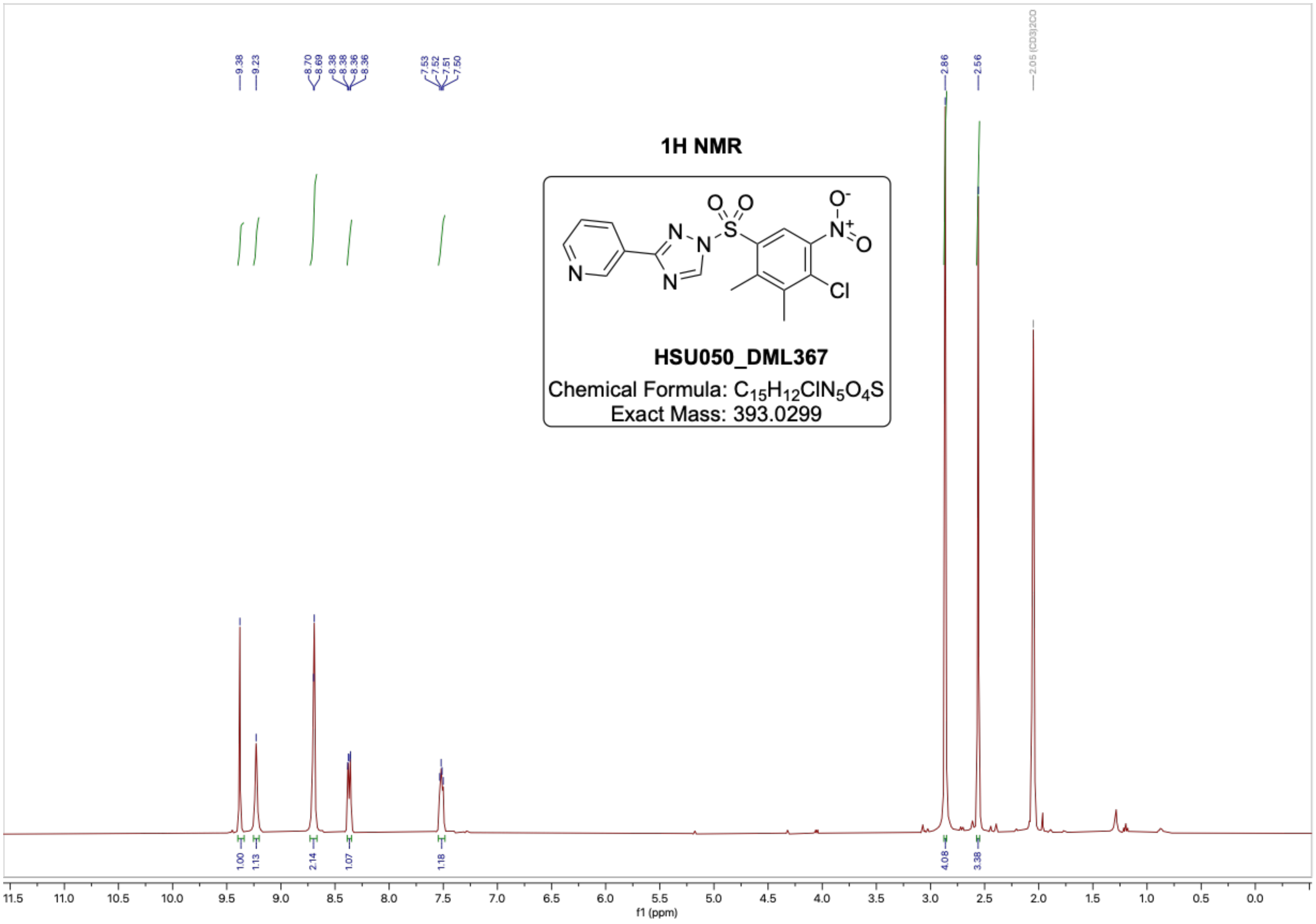

9.38
9.23
8.70
8.69
8.38
8.38
8.36
8.36
7.53
7.52
7.51
7.50
2.86
2.56
2.05 (CD3)2CO
1H NMR
HSU050_DML367
Chemical Formula: C15H12ClN5O4S
Exact Mass: 393.0299
1.00
1.13
2.14
1.07
1.18
4.08
3.38
f1 (ppm)

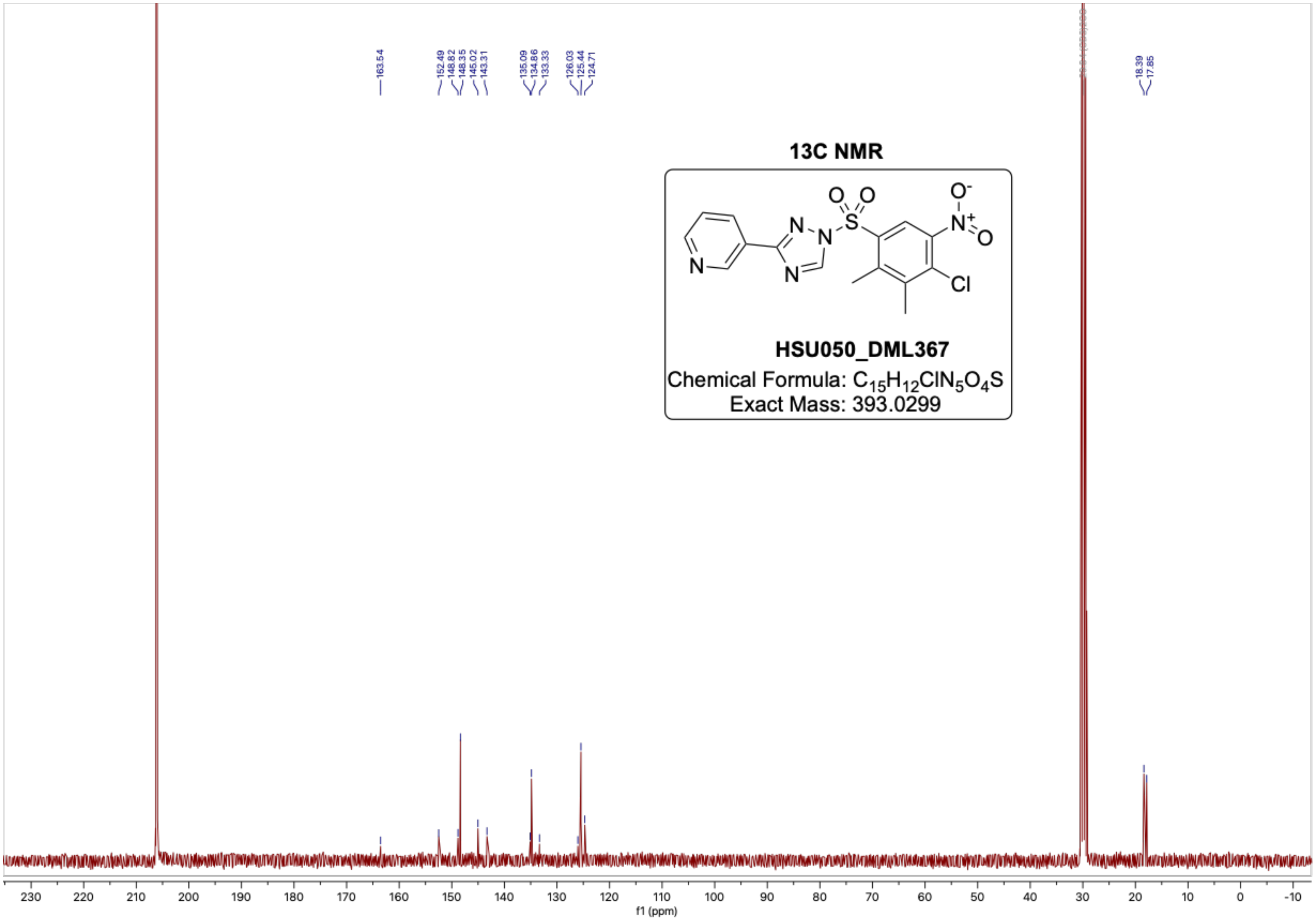

163.54
152.49
148.82
148.35
145.02
143.31
135.09
134.86
133.33
126.03
125.44
124.71
18.39
17.85
13C NMR
HSU050_DML367
Chemical Formula: C15H12ClN5O4S
Exact Mass: 393.0299
f1 (ppm)

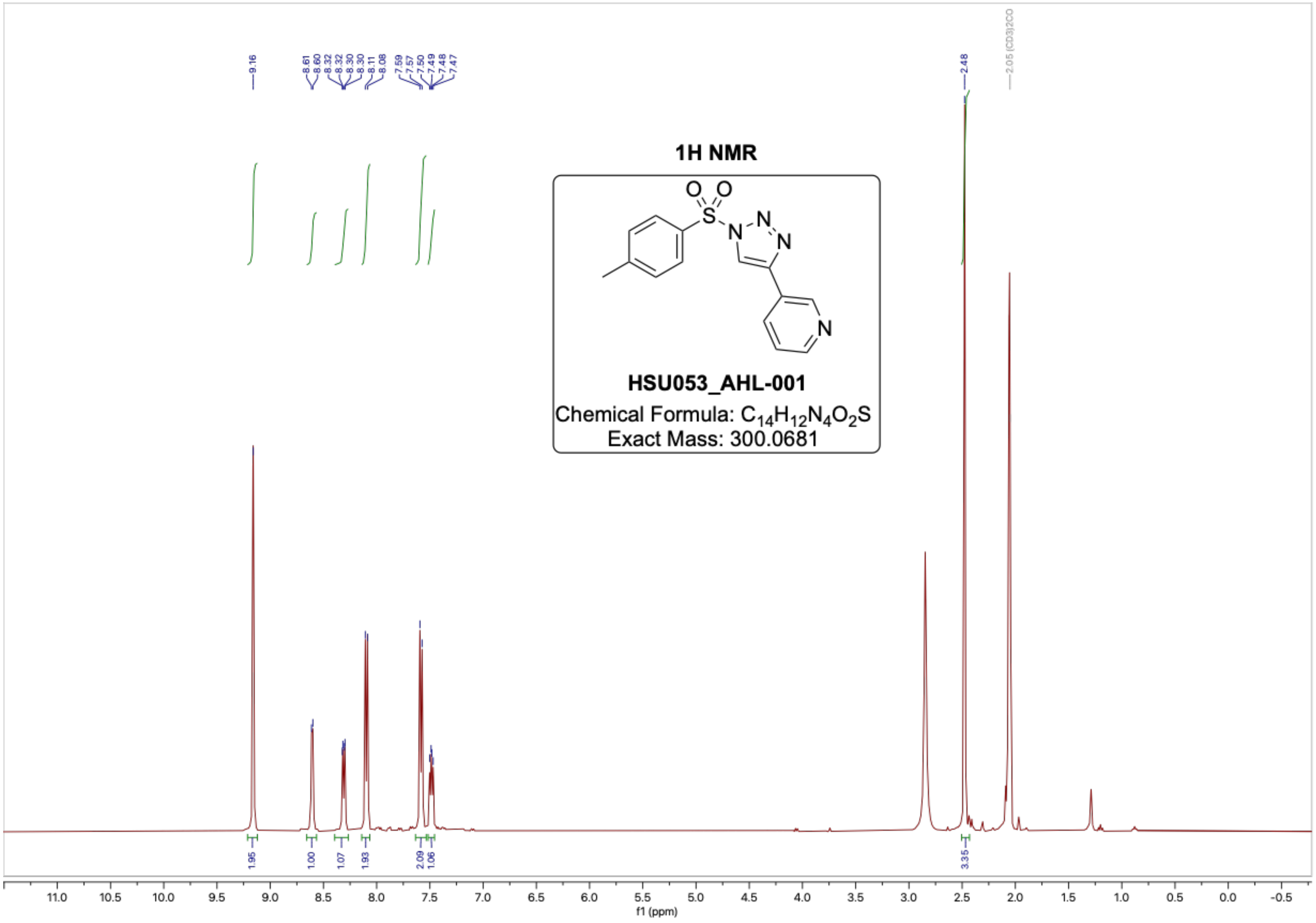

9.16
8.61
8.60
8.32
8.32
8.30
8.30
8.11
8.08
7.59
7.57
7.50
7.49
7.48
7.47
2.48
2.05 (CD3)2CO
1H NMR
HSU053_AHL-001
Chemical Formula: C14H12N4O2S
Exact Mass: 300.0681
1.95
1.00
1.07
1.93
2.09
1.06
3.35
f1 (ppm)

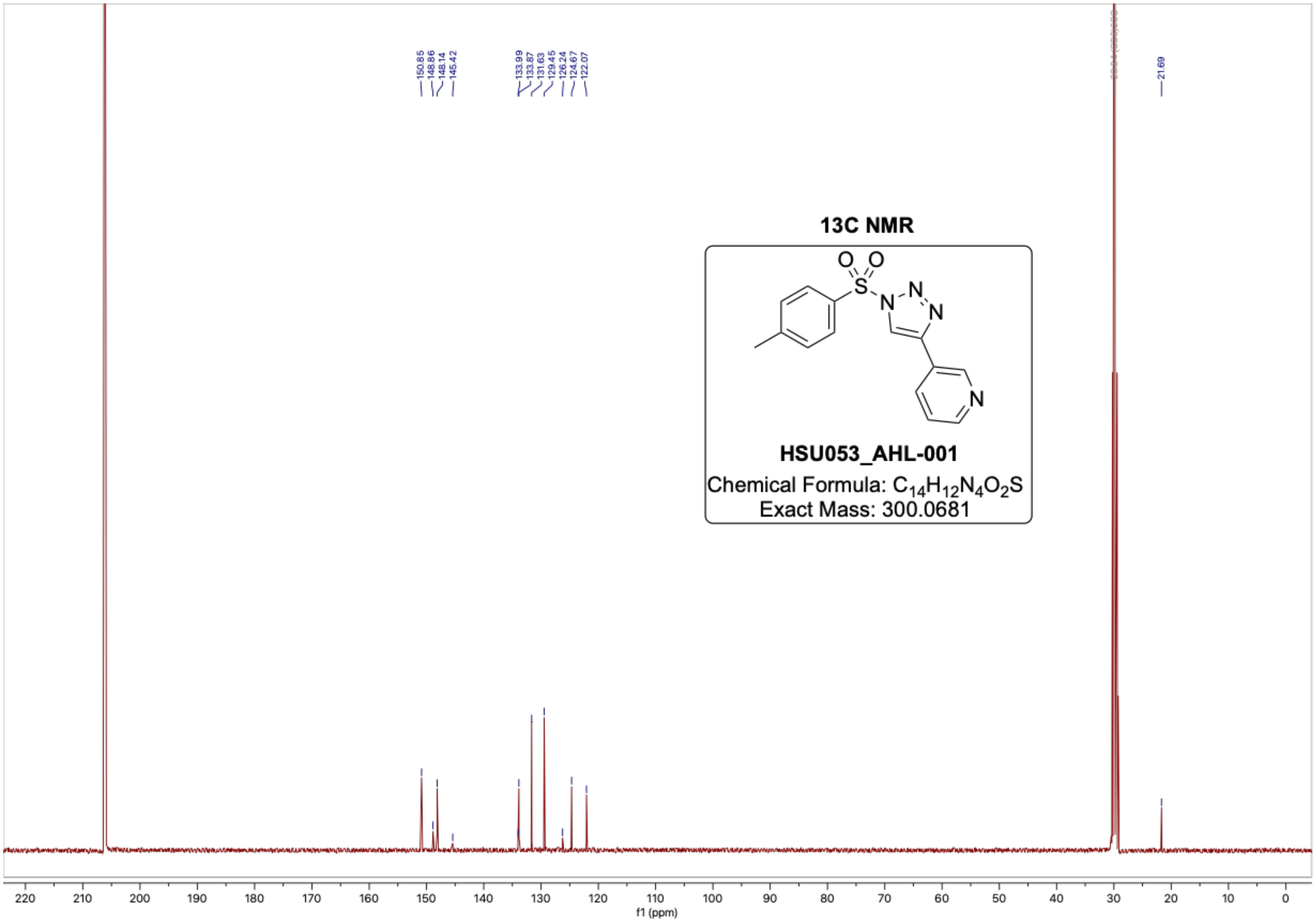

150.85
148.86
148.14
145.42
133.99
133.87
131.63
129.45
126.24
124.67
122.07
21.69
13C NMR
HSU053_AHL-001
Chemical Formula: C14H12N4O2S
Exact Mass: 300.0681
f1 (ppm)
220
210
200
190
180
170
160
150
140
130
120
110
100
90
80
70
60
50
40
30
20
10
0

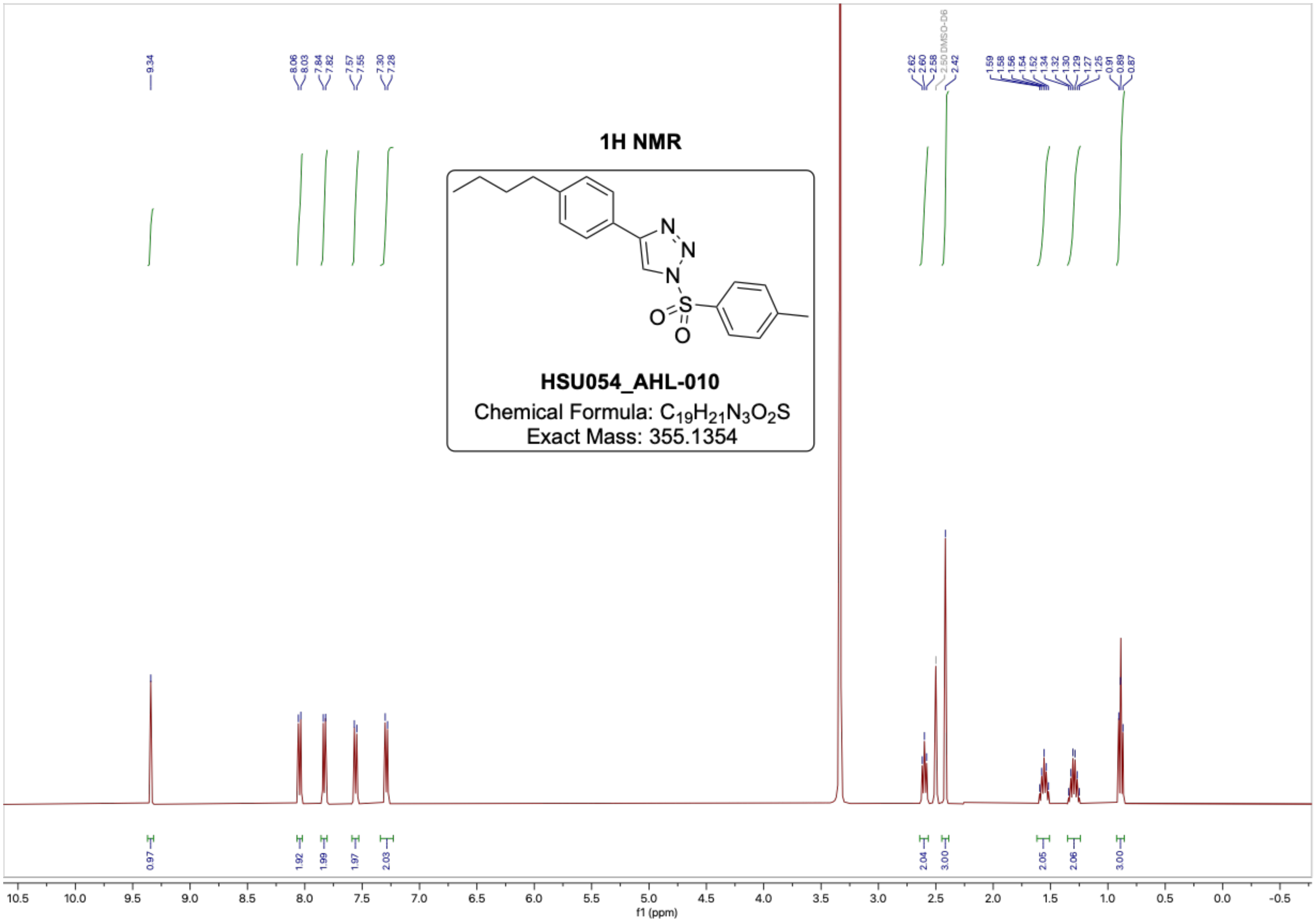

1H NMR
HSU054_AHL-010
Chemical Formula: $C_{19}H_{21}N_3O_2S$
Exact Mass: 355.1354
9.34
8.06
8.03
7.84
7.82
7.57
7.55
7.30
7.28
2.62
2.60
2.58
2.50 DMSO-D6
2.42
1.59
1.58
1.56
1.54
1.52
1.34
1.32
1.30
1.29
1.27
1.25
0.91
0.89
0.87
0.97
1.92
1.99
1.97
2.03
2.04
3.00
2.05
2.06
3.00
f1 (ppm)

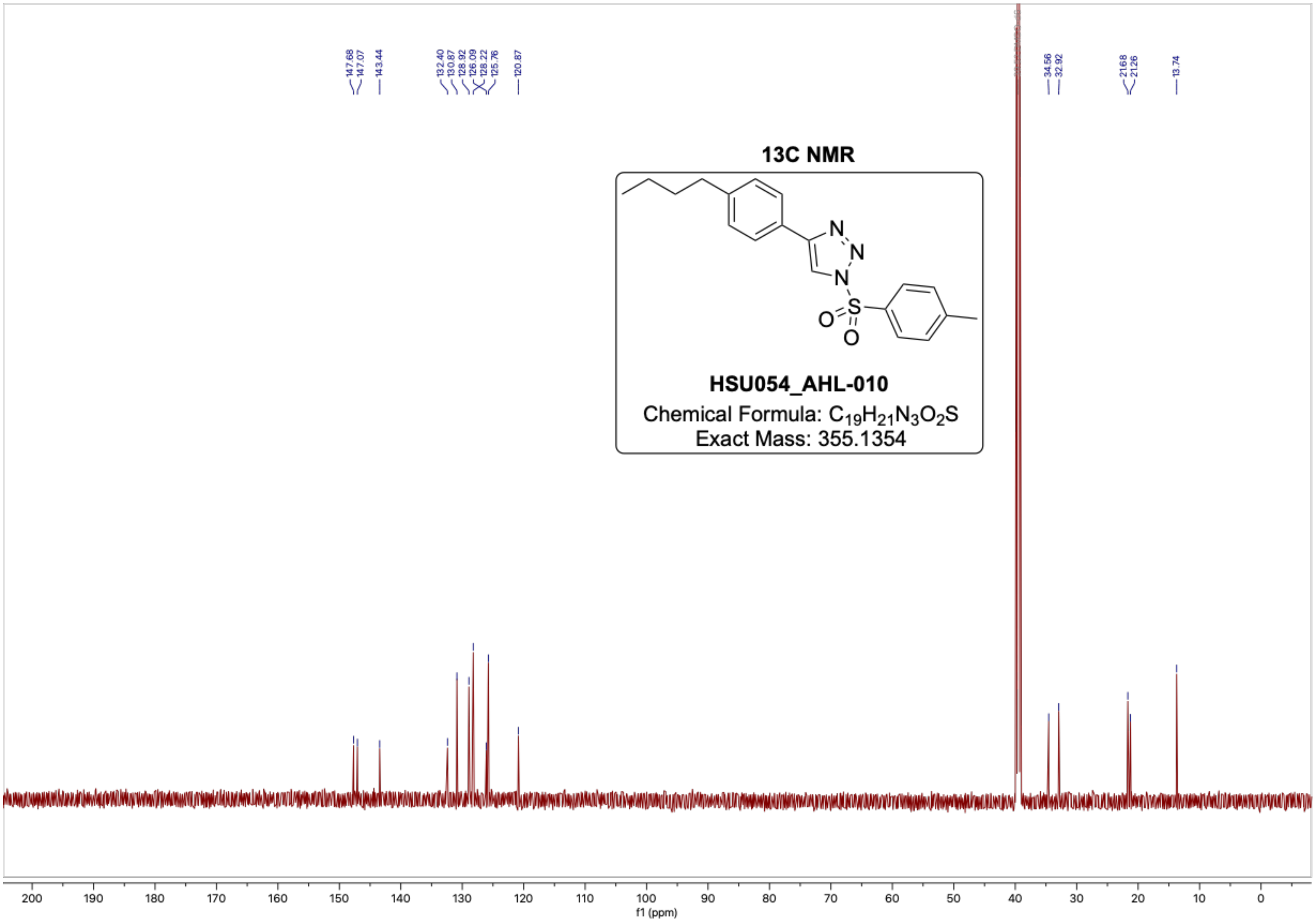
13C NMR
HSU054_AHL-010
Chemical Formula: C19H21N3O2S
Exact Mass: 355.1354
147.68
147.07
143.44
132.40
130.87
128.92
128.22
126.09
125.76
120.87
34.56
32.92
21.68
21.26
13.74
f1 (ppm)

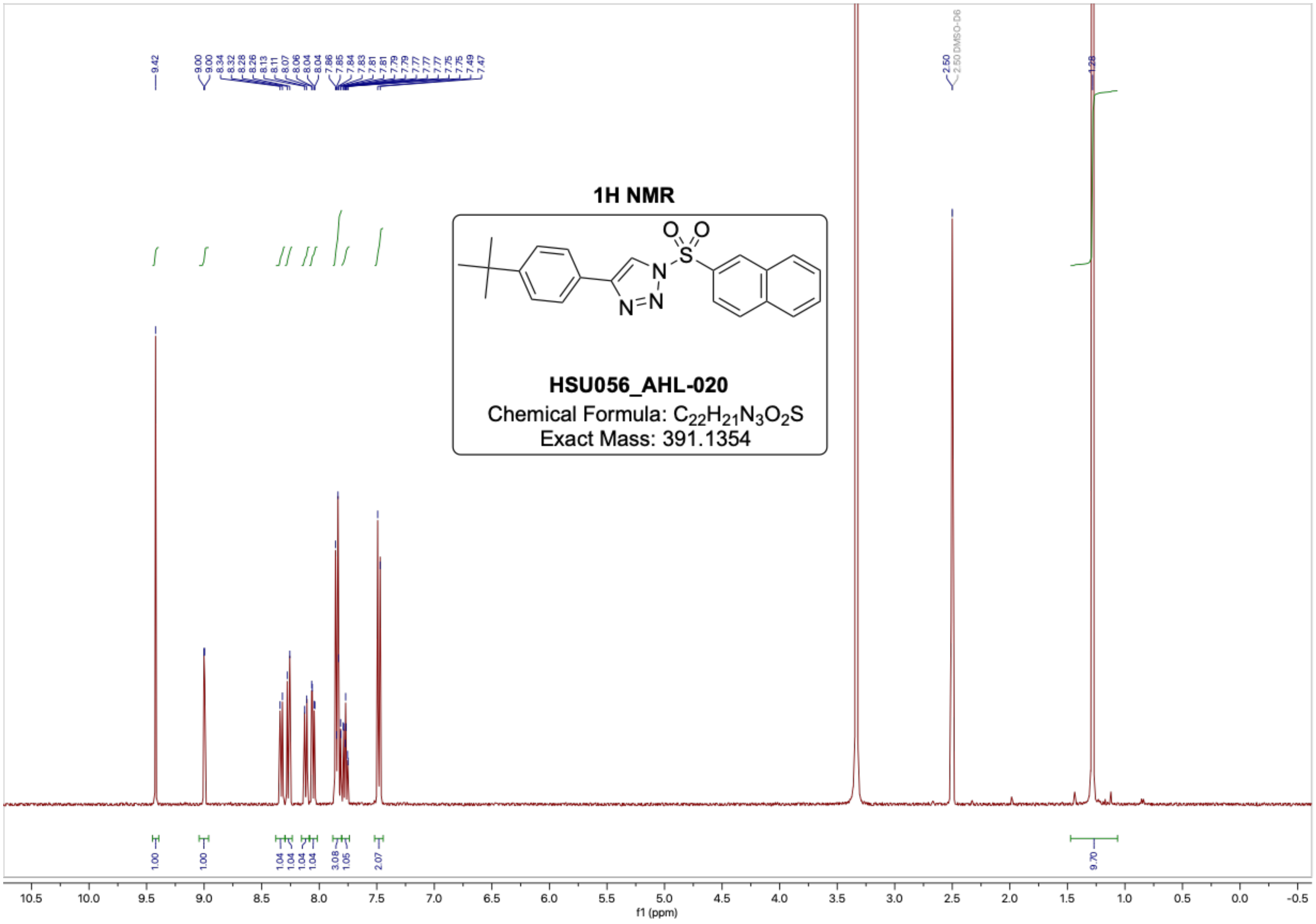

1H NMR
HSU056_AHL-020
Chemical Formula: C22H21N3O2S
Exact Mass: 391.1354
f1 (ppm)

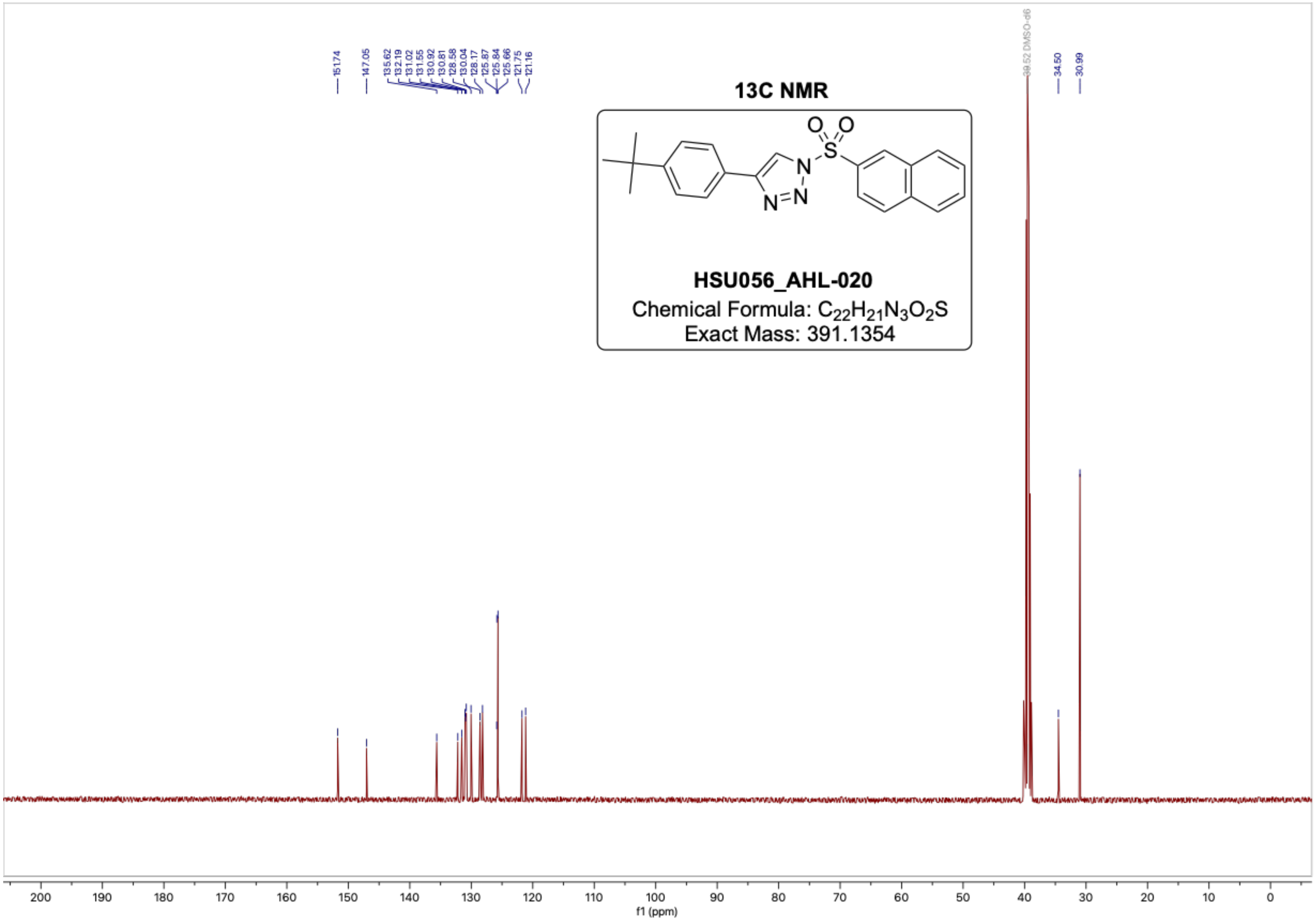
151.74
147.05
135.62
132.19
131.02
131.55
130.92
130.81
128.58
130.04
128.17
125.87
125.84
125.66
121.75
121.16
39.52 DMSO-d6
34.50
30.99
13C NMR
O
O
S
N
N
N
HSU056_AHL-020
Chemical Formula: C22H21N3O2S
Exact Mass: 391.1354
200
190
180
170
160
150
140
130
120
110
100
90
80
70
60
50
40
30
20
10
0
f1 (ppm)

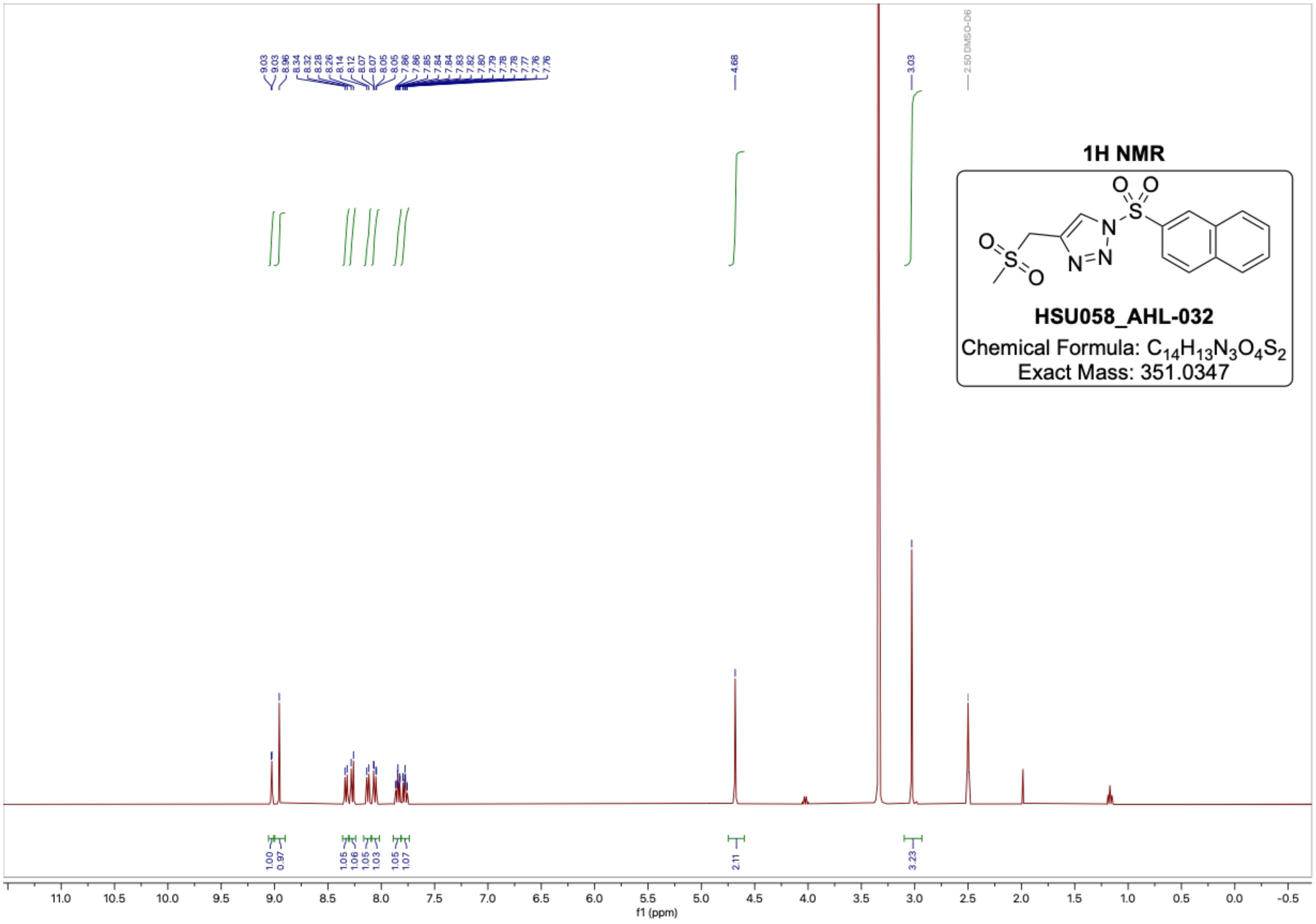
1H NMR
HSU058_AHL-032
Chemical Formula: C14H13N3O4S2
Exact Mass: 351.0347
f1 (ppm)

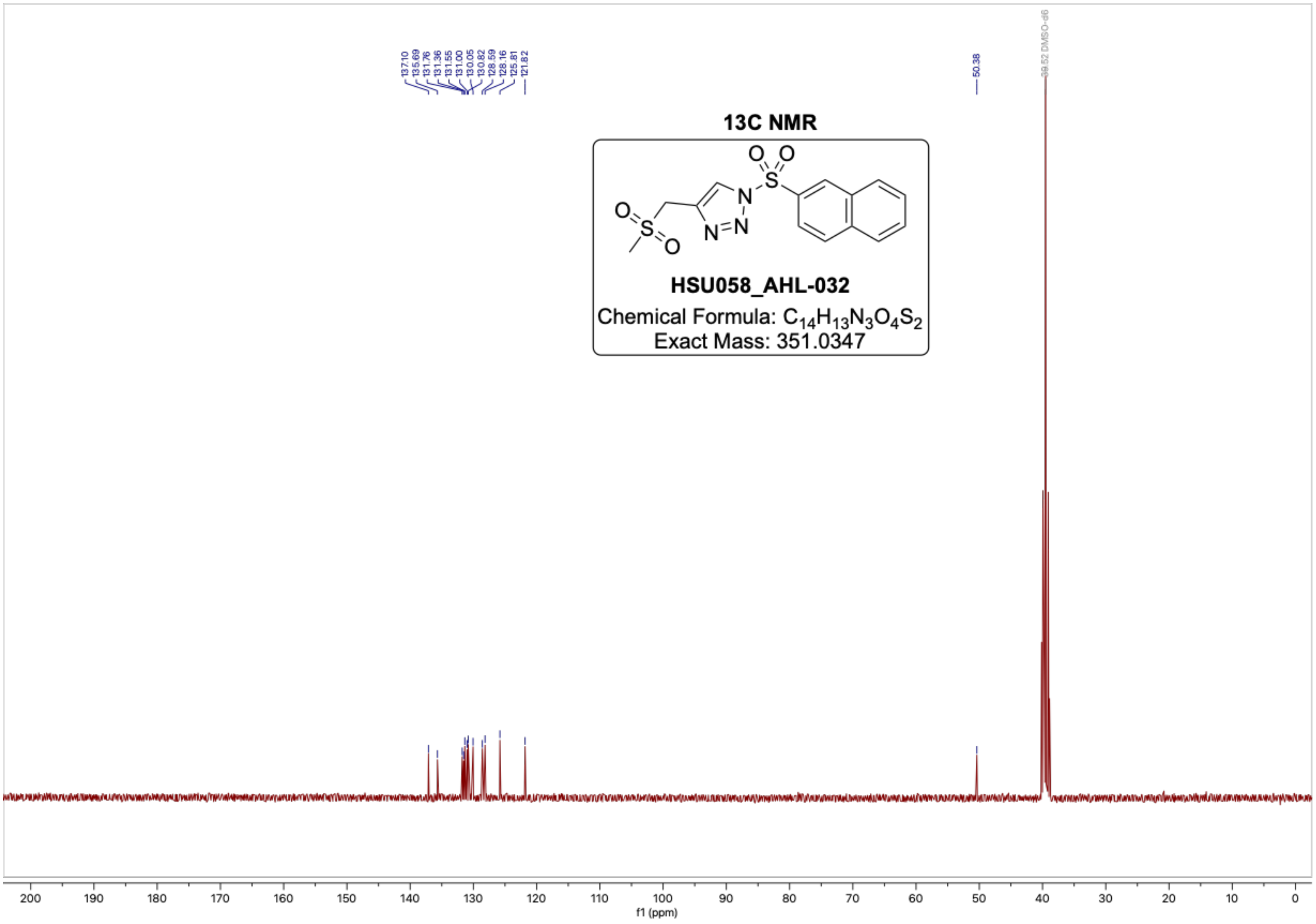
137.10
135.69
131.76
131.36
131.55
131.00
130.05
130.82
128.59
128.16
125.81
121.82
50.38
39.52 DMSO-d6
13C NMR
HSU058_AHL-032
Chemical Formula: C14H13N3O4S2
Exact Mass: 351.0347
200
190
180
170
160
150
140
130
120
110
100
90
80
70
60
50
40
30
20
10
0
f1 (ppm)

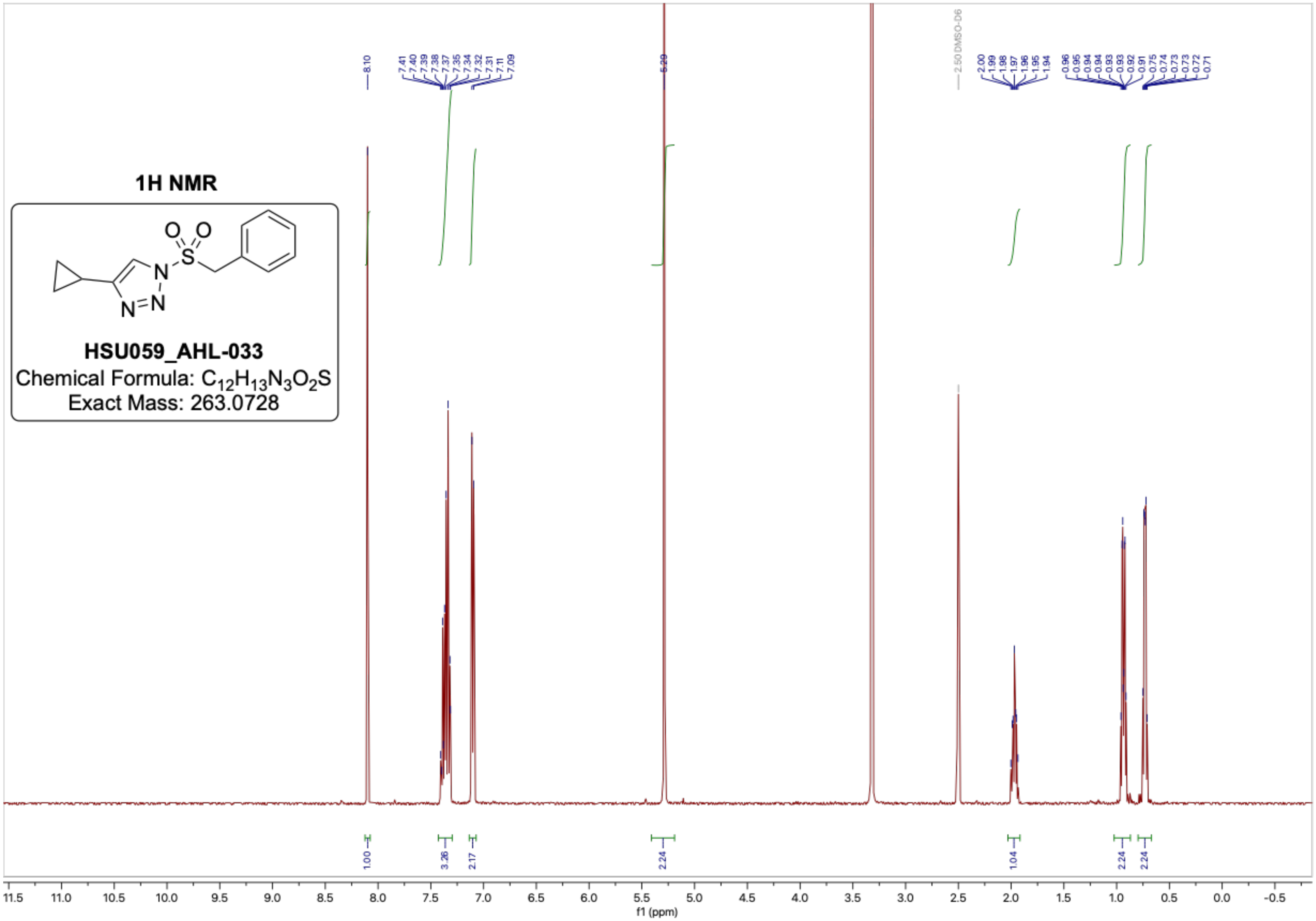
1H NMR
HSU059_AHL-033
Chemical Formula: C12H13N3O2S
Exact Mass: 263.0728
8.10
7.41
7.40
7.39
7.38
7.37
7.35
7.34
7.32
7.31
7.11
7.09
5.29
2.50 DMSO-D6
2.00
1.99
1.98
1.97
1.96
1.95
1.94
0.96
0.95
0.94
0.94
0.93
0.93
0.92
0.91
0.75
0.74
0.73
0.73
0.72
0.71
1.00
3.26
2.17
2.24
1.04
2.24
2.24
f1 (ppm)

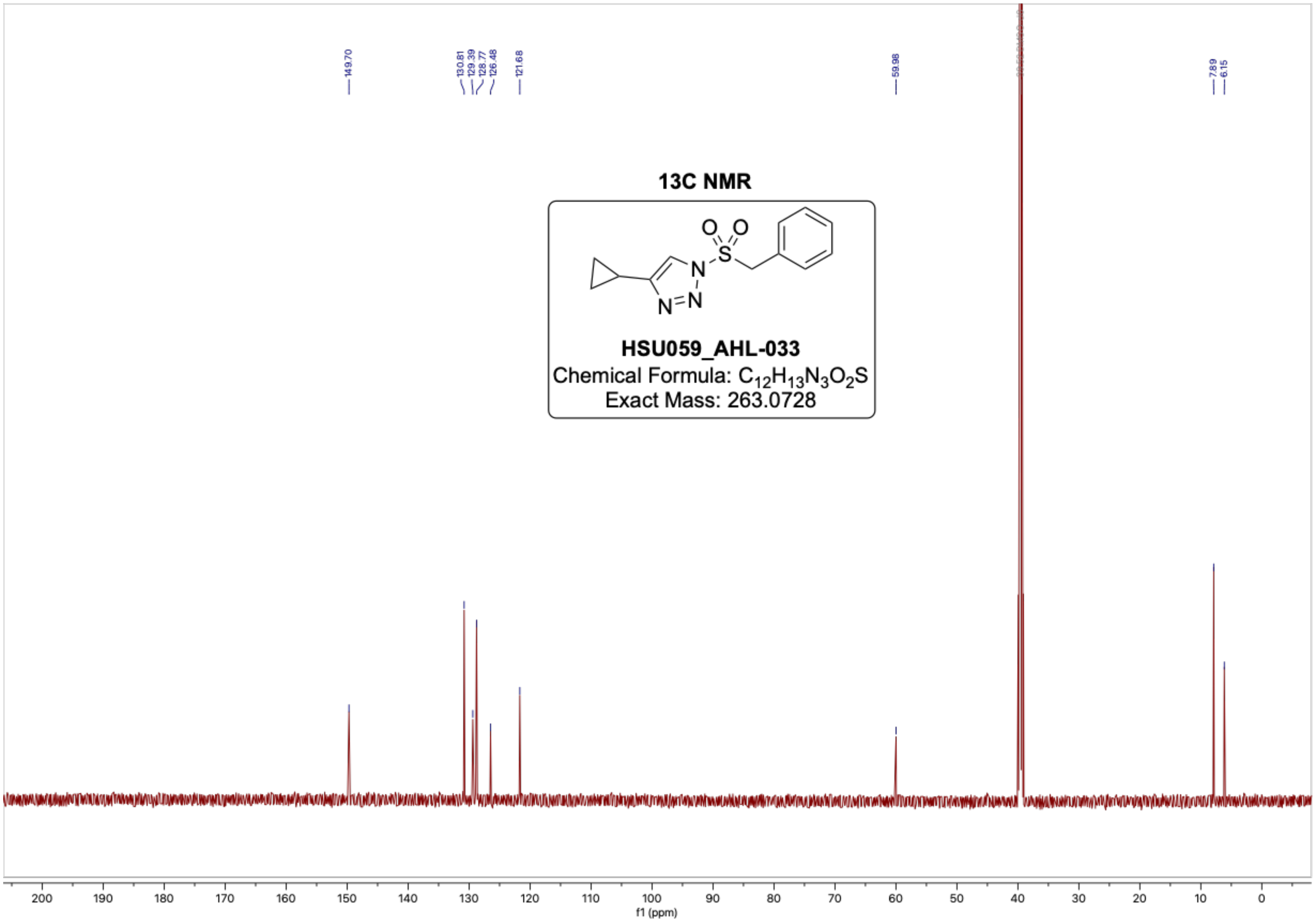

149.70
130.81
129.39
128.77
126.48
121.68
59.98
7.89
6.15
13C NMR
O
O
S
N
N
N
HSU059_AHL-033
Chemical Formula: C12H13N3O2S
Exact Mass: 263.0728
200
190
180
170
160
150
140
130
120
110
100
90
80
70
60
50
40
30
20
10
0
f1 (ppm)

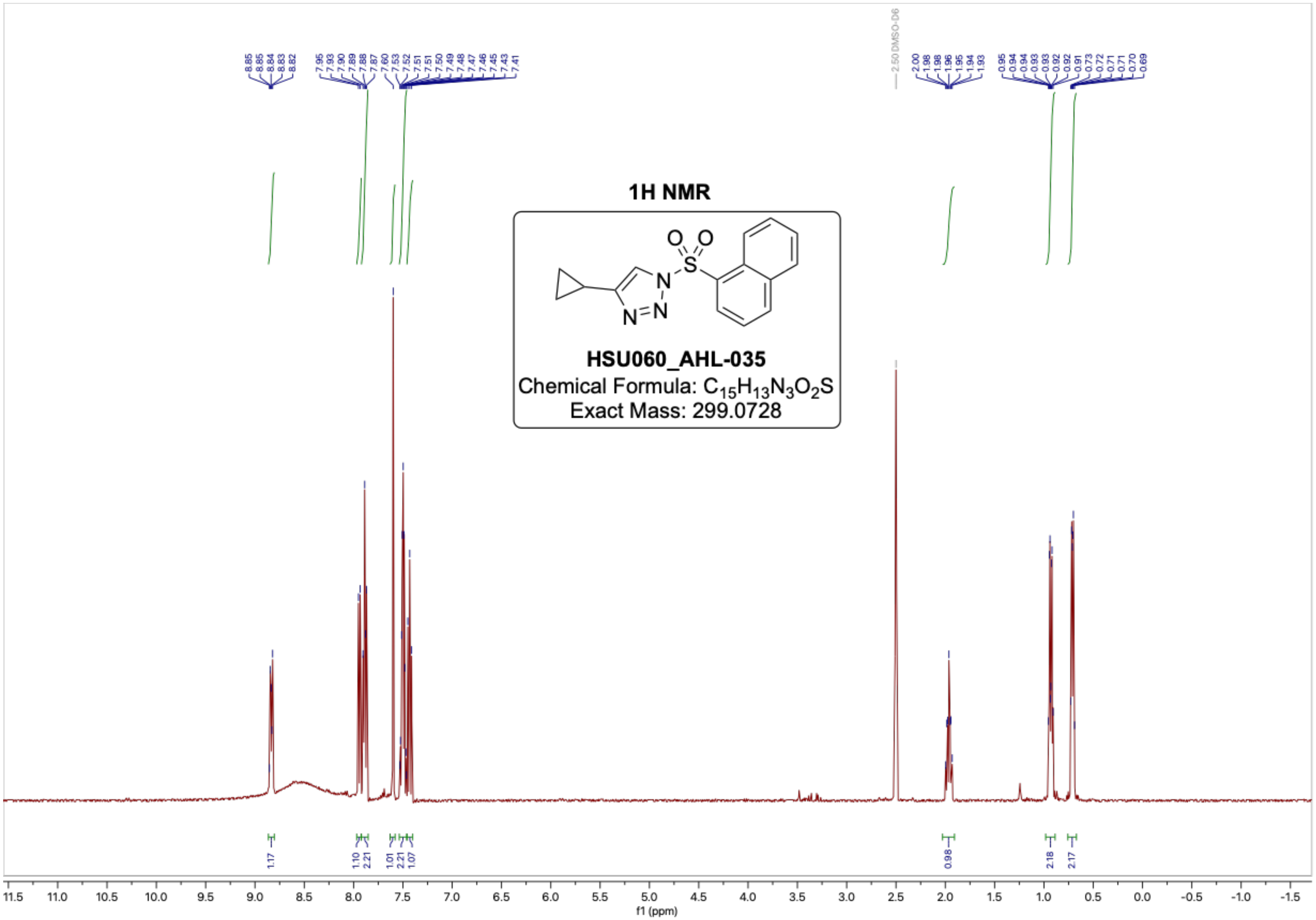

1H NMR
HSU060_AHL-035
Chemical Formula: C15H13N3O2S
Exact Mass: 299.0728
f1 (ppm)
2.50 DMSO-D6

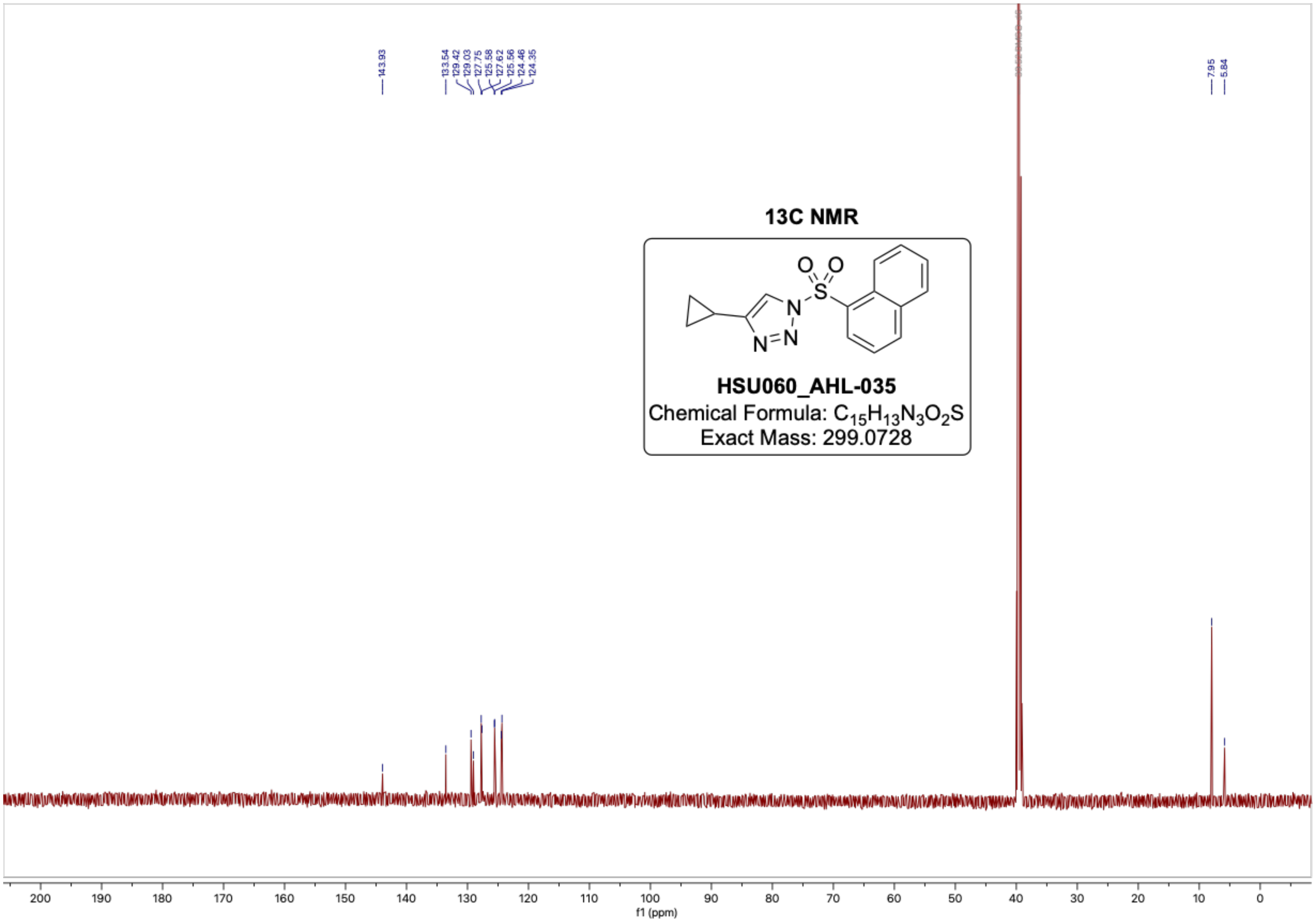
143.93
133.54
129.42
129.03
127.75
125.58
127.62
125.56
124.46
124.35
7.95
5.84
13C NMR
HSU060_AHL-035
Chemical Formula: $C_{15}H_{13}N_3O_2S$
Exact Mass: 299.0728
f1 (ppm)

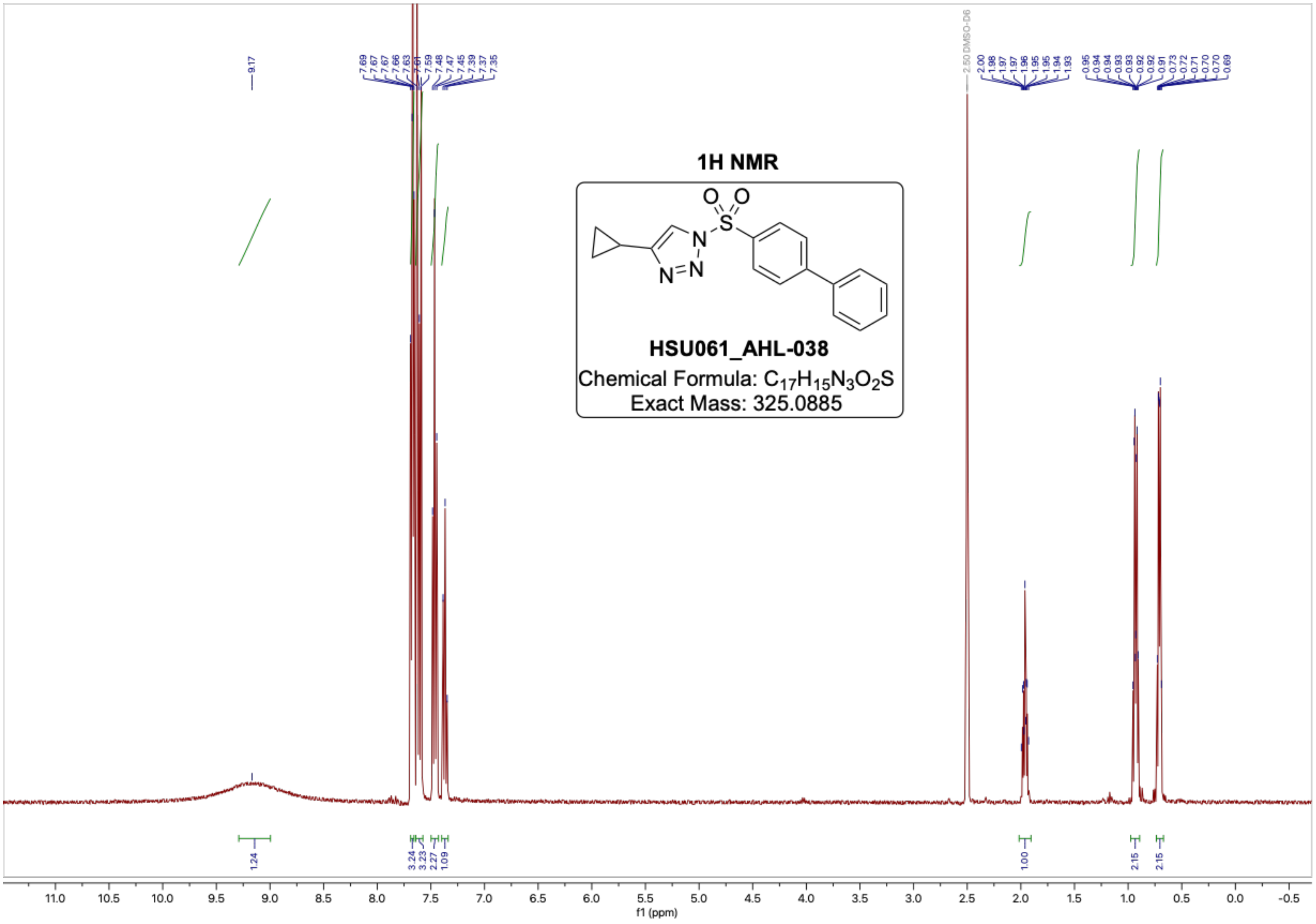
1H NMR
HSU061_AHL-038
Chemical Formula: C17H15N3O2S
Exact Mass: 325.0885
f1 (ppm)

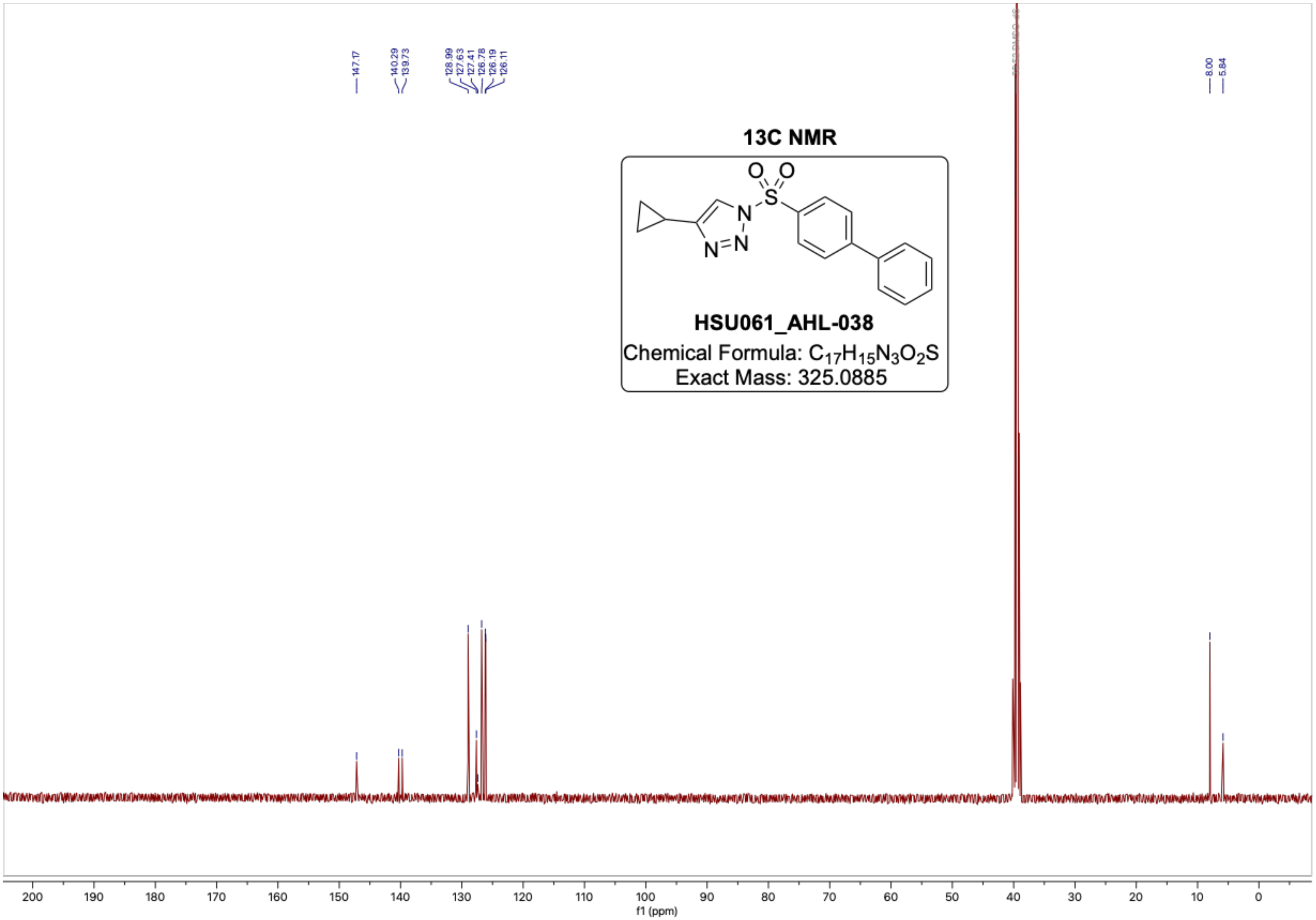
13C NMR
HSU061_AHL-038
Chemical Formula: $C_{17}H_{15}N_3O_2S$
Exact Mass: 325.0885
147.17
140.29
139.73
128.99
127.63
127.41
126.78
126.19
126.11
8.00
5.84
200 190 180 170 160 150 140 130 120 110 100 90 80 70 60 50 40 30 20 10 0
f1 (ppm)

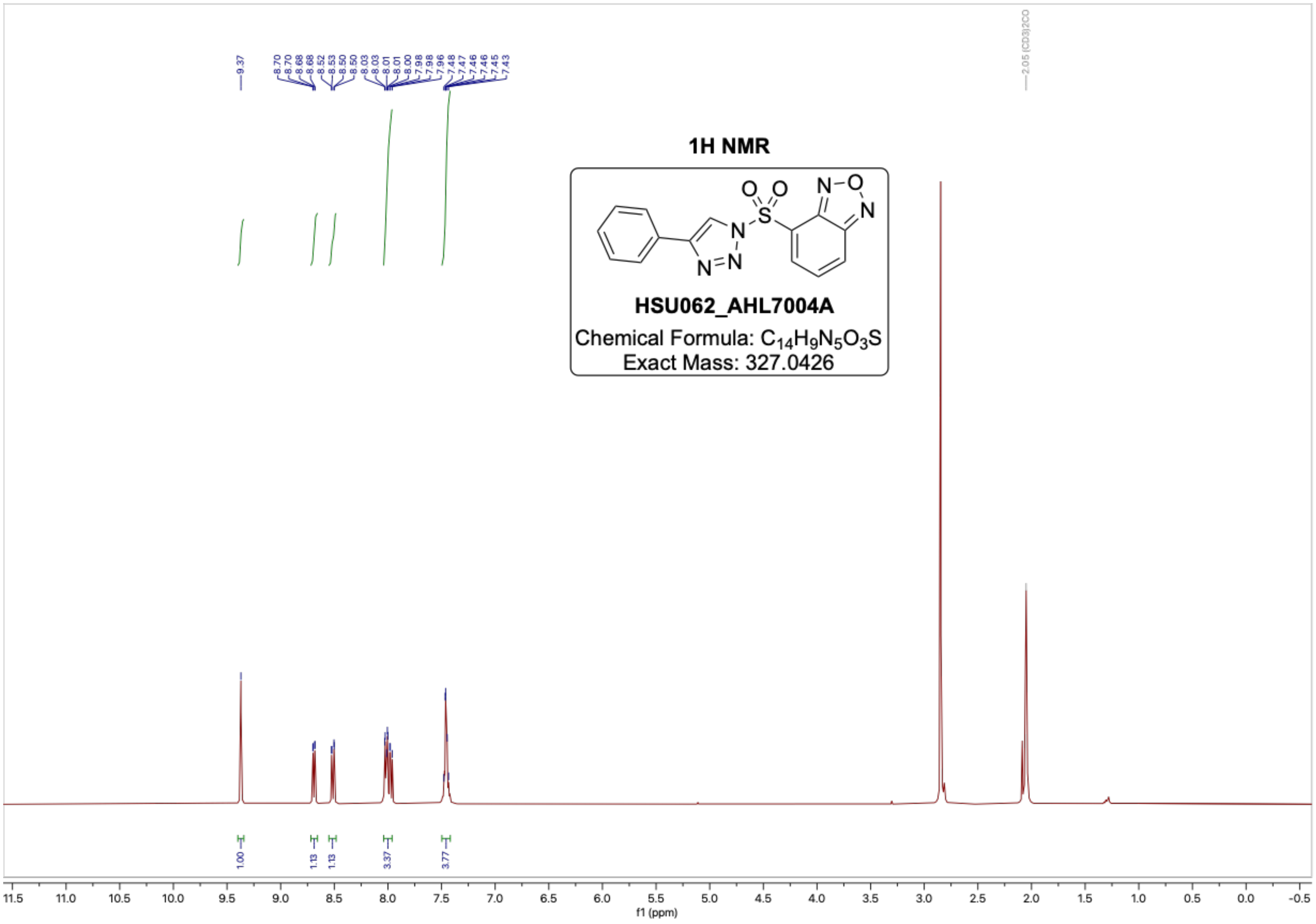

1H NMR
HSU062_AHL7004A
Chemical Formula: C14H9N5O3S
Exact Mass: 327.0426
9.37
8.70
8.70
8.68
8.68
8.52
8.53
8.50
8.50
8.03
8.03
8.01
8.01
8.00
7.98
7.98
7.96
7.48
7.47
7.46
7.46
7.45
7.43
2.05 (CD3)2CO
1.00
1.13
1.13
3.37
3.77
f1 (ppm)

13C NMR

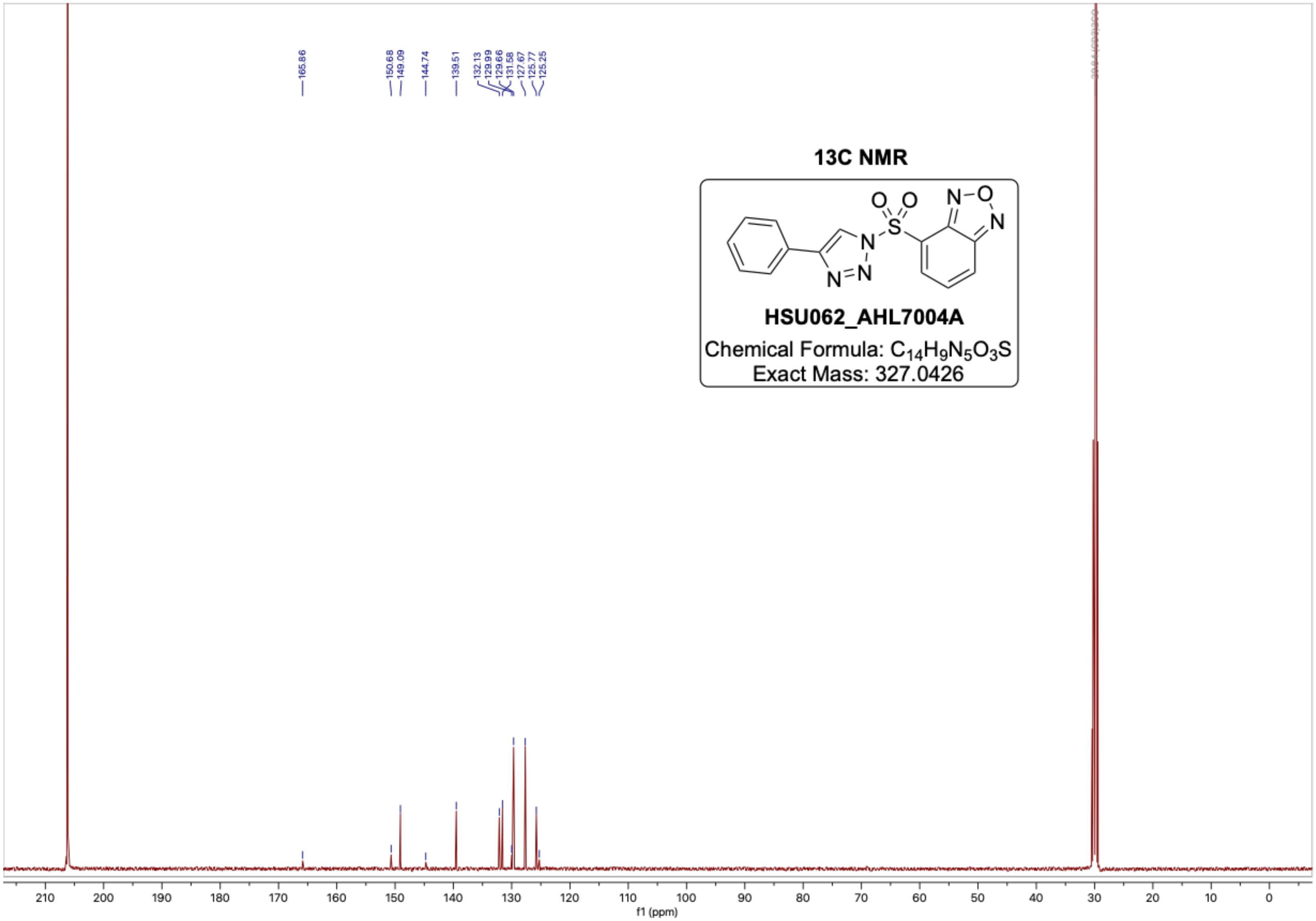

HSU062_AHL7004A

Chemical Formula: $C_{14}H_9N_5O_3S$

Exact Mass: 327.0426

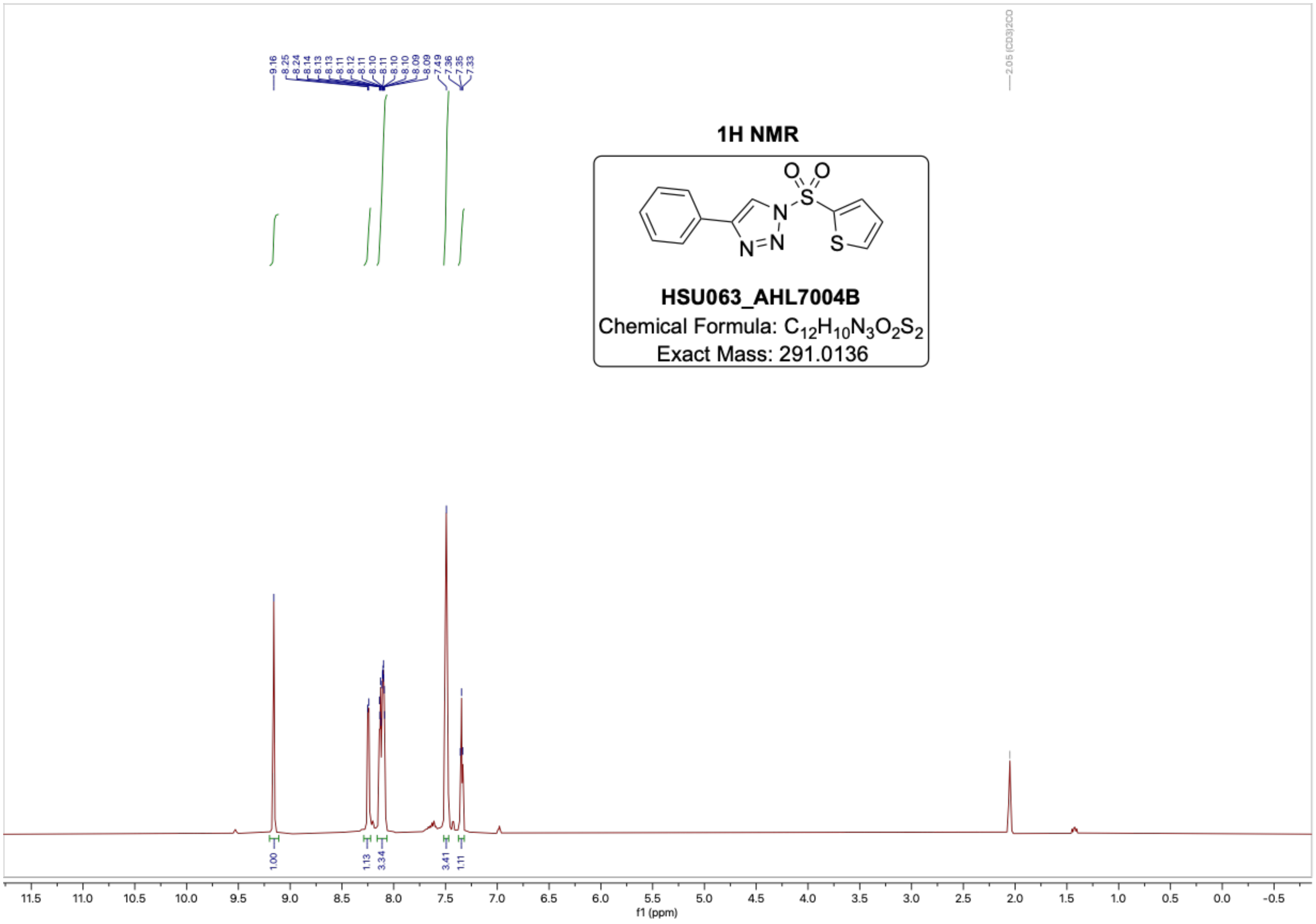

1H NMR
HSU063_AHL7004B
Chemical Formula: C12H10N3O2S2
Exact Mass: 291.0136
9.16
8.25
8.24
8.14
8.13
8.13
8.11
8.11
8.12
8.11
8.10
8.11
8.10
8.10
8.09
8.09
7.49
7.36
7.35
7.33
2.05 (CD3)2CO
1.00
1.13
3.34
3.41
1.11
f1 (ppm)

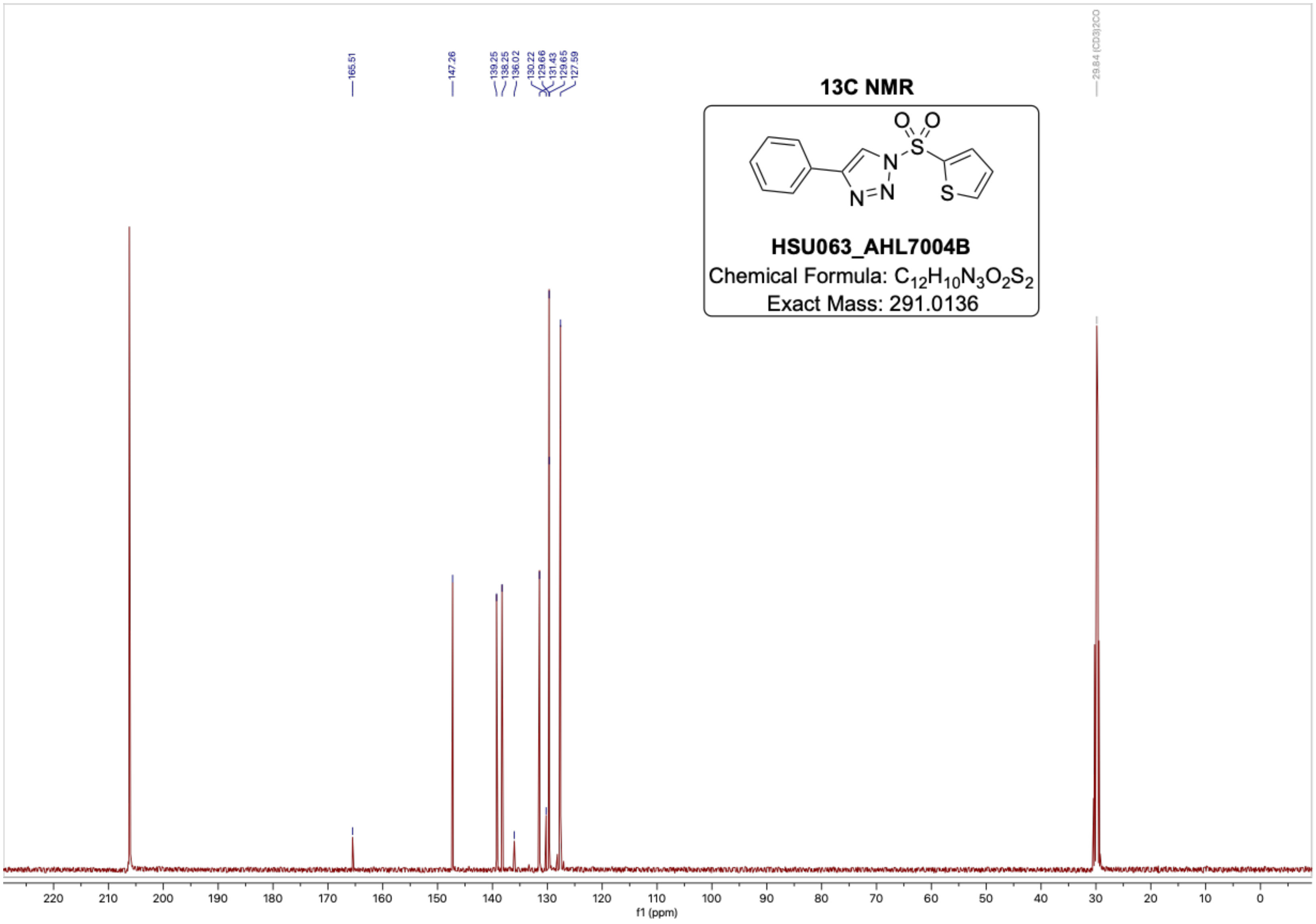
13C NMR
HSU063_AHL7004B
Chemical Formula: C12H10N3O2S2
Exact Mass: 291.0136
165.51
147.26
139.25
138.25
136.02
130.22
129.66
131.43
129.65
127.59
29.84 (CD3)2CO
220
210
200
190
180
170
160
150
140
130
120
110
100
90
80
70
60
50
40
30
20
10
0
f1 (ppm)

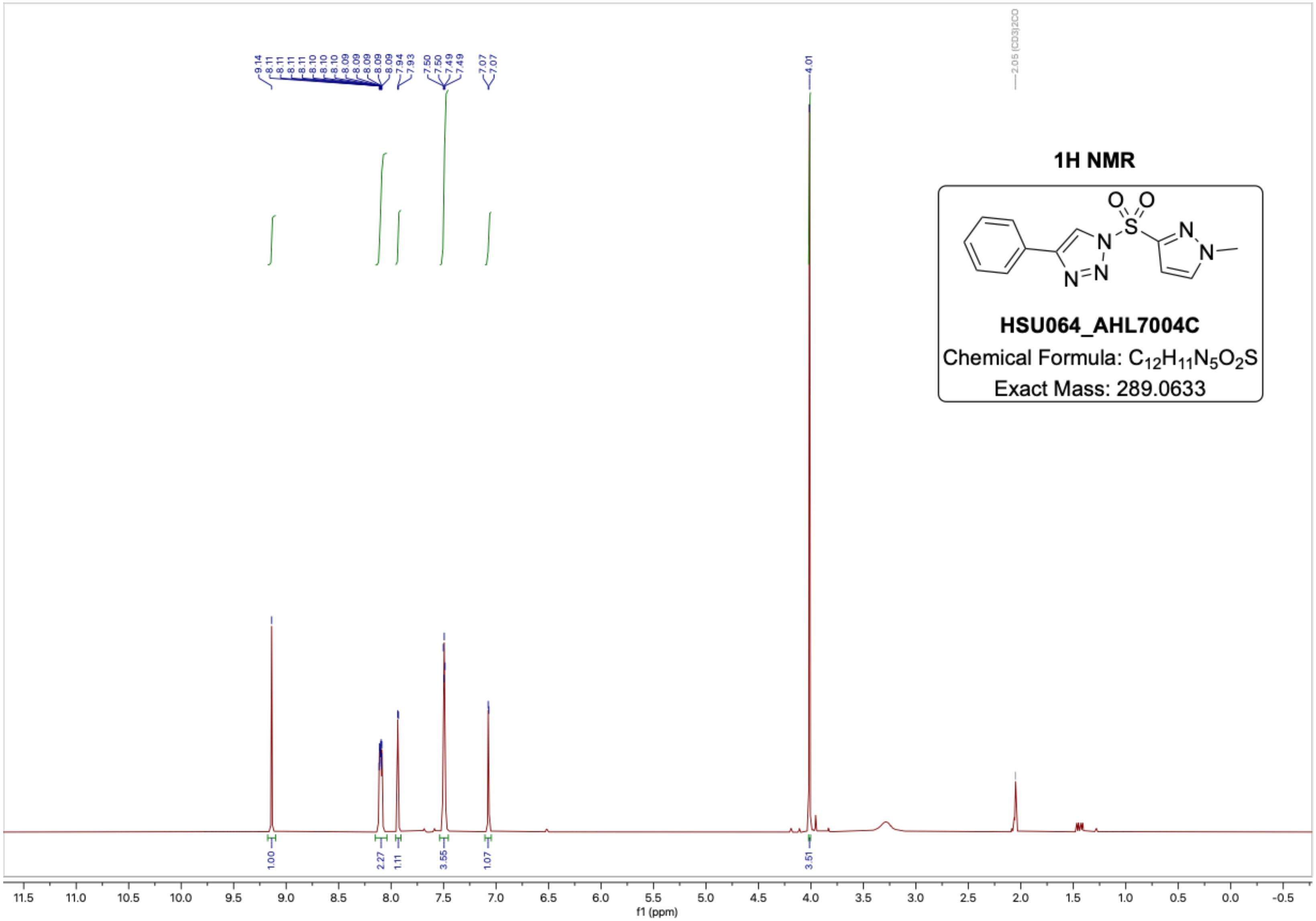
1H NMR
HSU064_AHL7004C
Chemical Formula: C12H11N5O2S
Exact Mass: 289.0633
9.14
8.11
8.11
8.11
8.11
8.10
8.10
8.10
8.09
8.09
8.09
8.09
8.09
7.94
7.93
7.50
7.50
7.49
7.49
7.07
7.07
4.01
2.05 (CD3)2CO
1.00
2.27
1.11
3.55
1.07
3.51
11.5
11.0
10.5
10.0
9.5
9.0
8.5
8.0
7.5
7.0
6.5
6.0
5.5
5.0
4.5
4.0
3.5
3.0
2.5
2.0
1.5
1.0
0.5
0.0
-0.5
f1 (ppm)

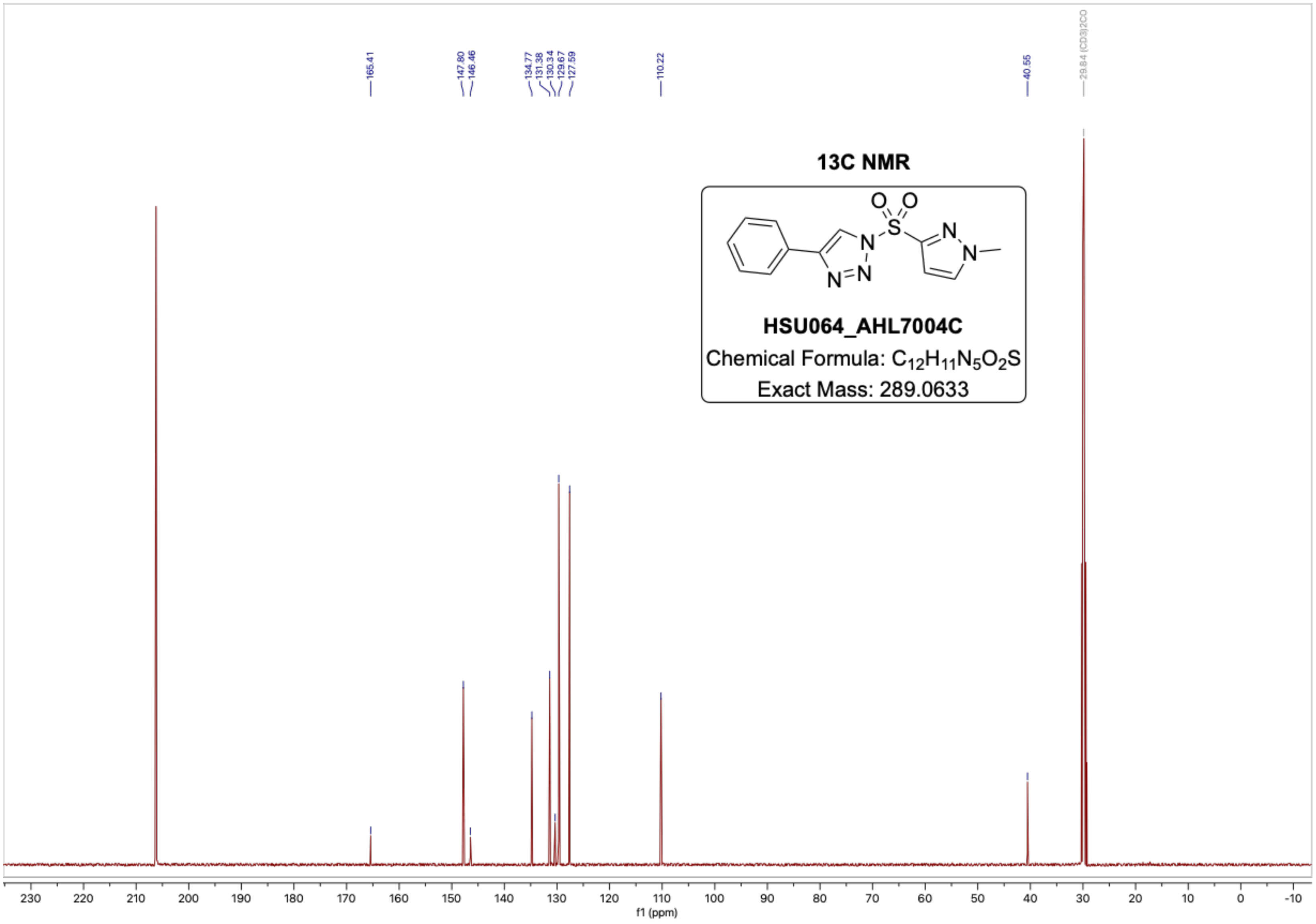

165.41
147.80
146.46
134.77
131.38
130.34
129.67
127.59
110.22
40.55
29.84 (CD3)2CO
13C NMR
O
O
S
N
N
N
N
N
HSU064_AHL7004C
Chemical Formula: $C_{12}H_{11}N_5O_2S$
Exact Mass: 289.0633
230
220
210
200
190
180
170
160
150
140
130
120
110
100
90
80
70
60
50
40
30
20
10
0
-10
f1 (ppm)

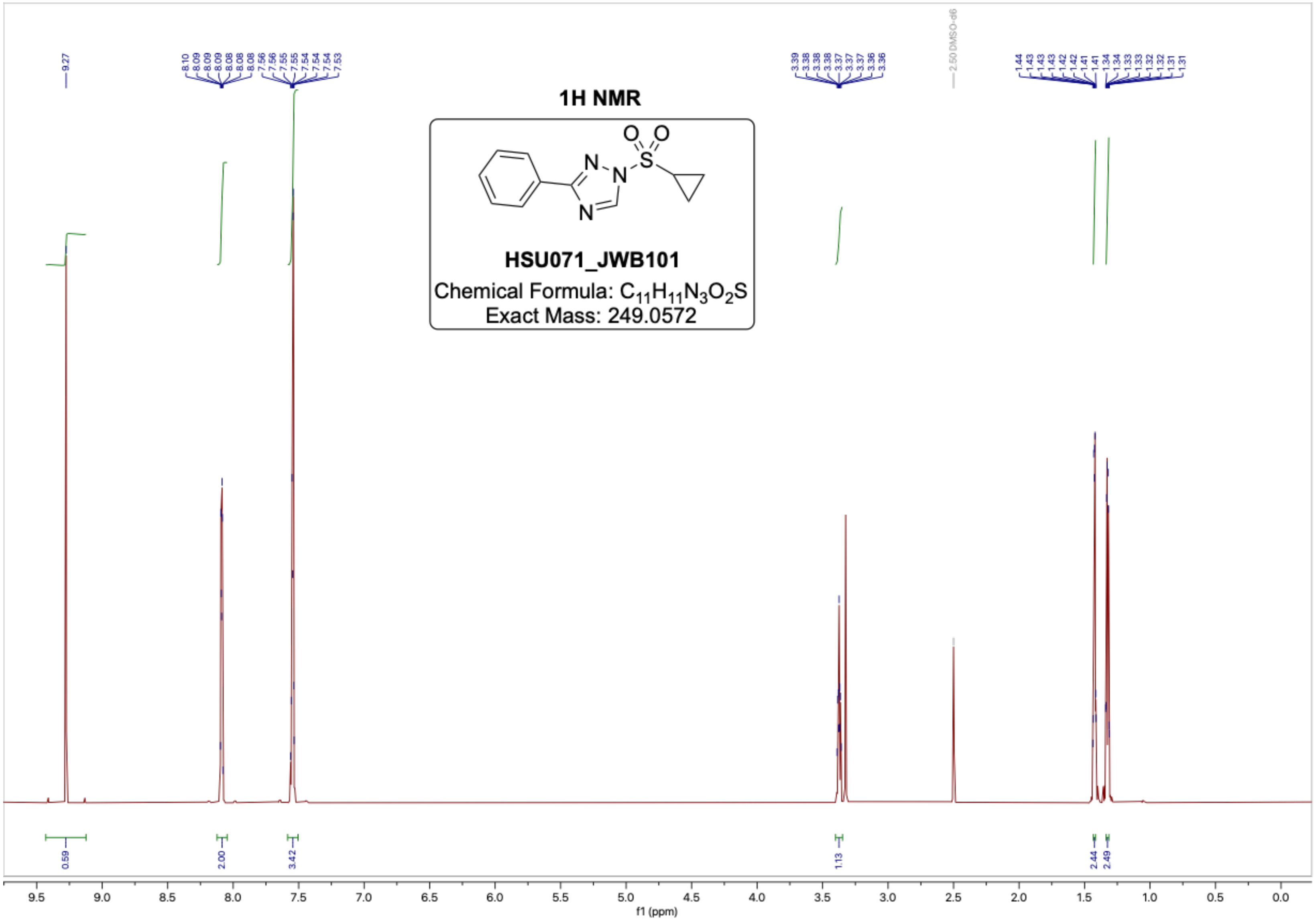
1H NMR
HSU071_JWB101
Chemical Formula: C11H11N3O2S
Exact Mass: 249.0572
9.27
8.10 8.09 8.09 8.09 8.08 8.08 8.08 7.56 7.56 7.55 7.55 7.54 7.54 7.54 7.53
3.39 3.38 3.38 3.38 3.37 3.37 3.37 3.36 3.36
2.50 DMSO-d6
1.44 1.43 1.43 1.43 1.42 1.42 1.41 1.34 1.34 1.33 1.33 1.33 1.32 1.32 1.31 1.31
0.59
2.00
3.42
1.13
2.44
2.49
9.5 9.0 8.5 8.0 7.5 7.0 6.5 6.0 5.5 5.0 4.5 4.0 3.5 3.0 2.5 2.0 1.5 1.0 0.5 0.0
f1 (ppm)

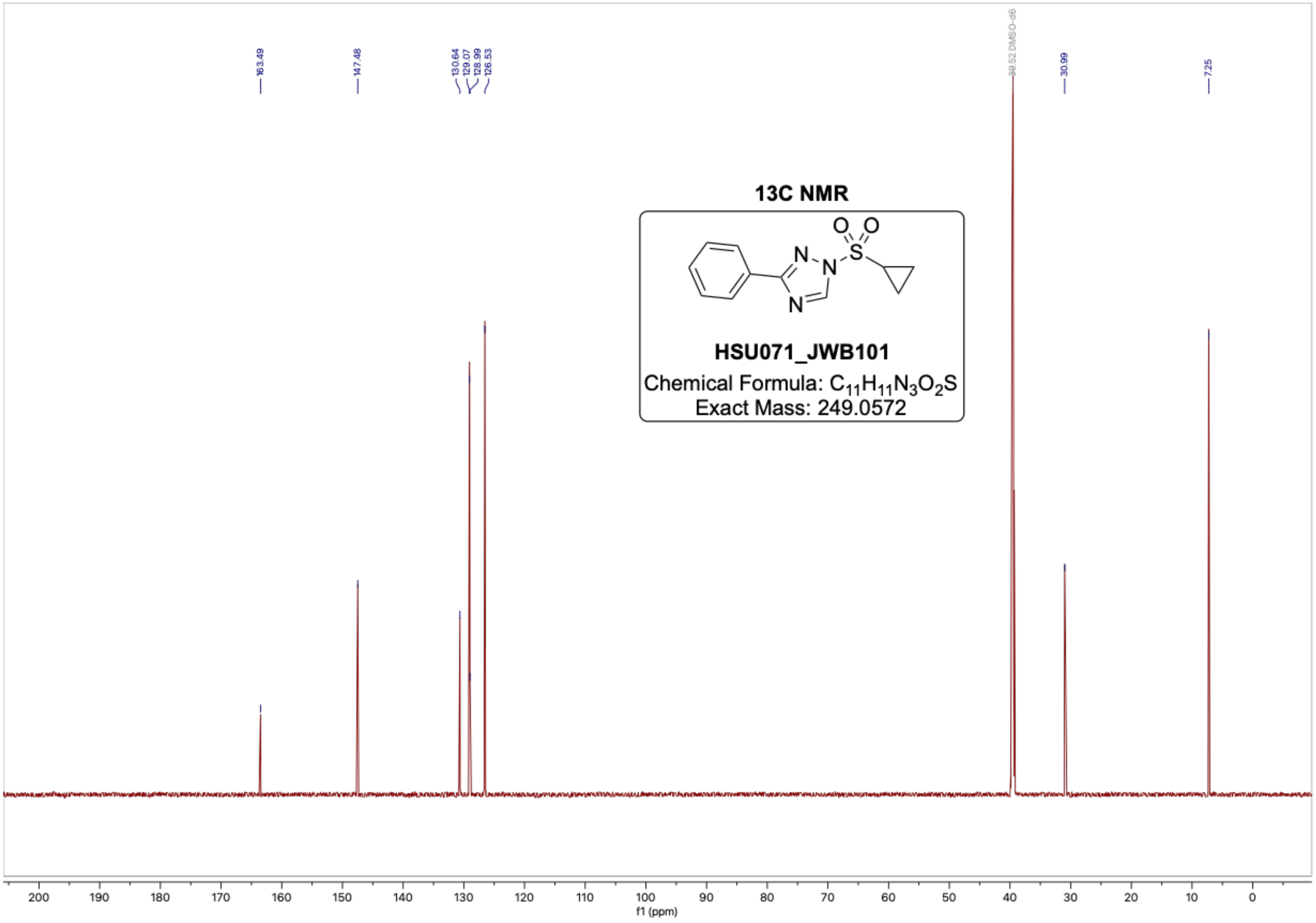
163.49
147.48
130.64
129.07
128.99
126.53
39.52 DMSO-d6
30.99
7.25
13C NMR
HSU071_JWB101
Chemical Formula: $C_{11}H_{11}N_3O_2S$
Exact Mass: 249.0572
200
190
180
170
160
150
140
130
120
110
100
90
80
70
60
50
40
30
20
10
0
f1 (ppm)

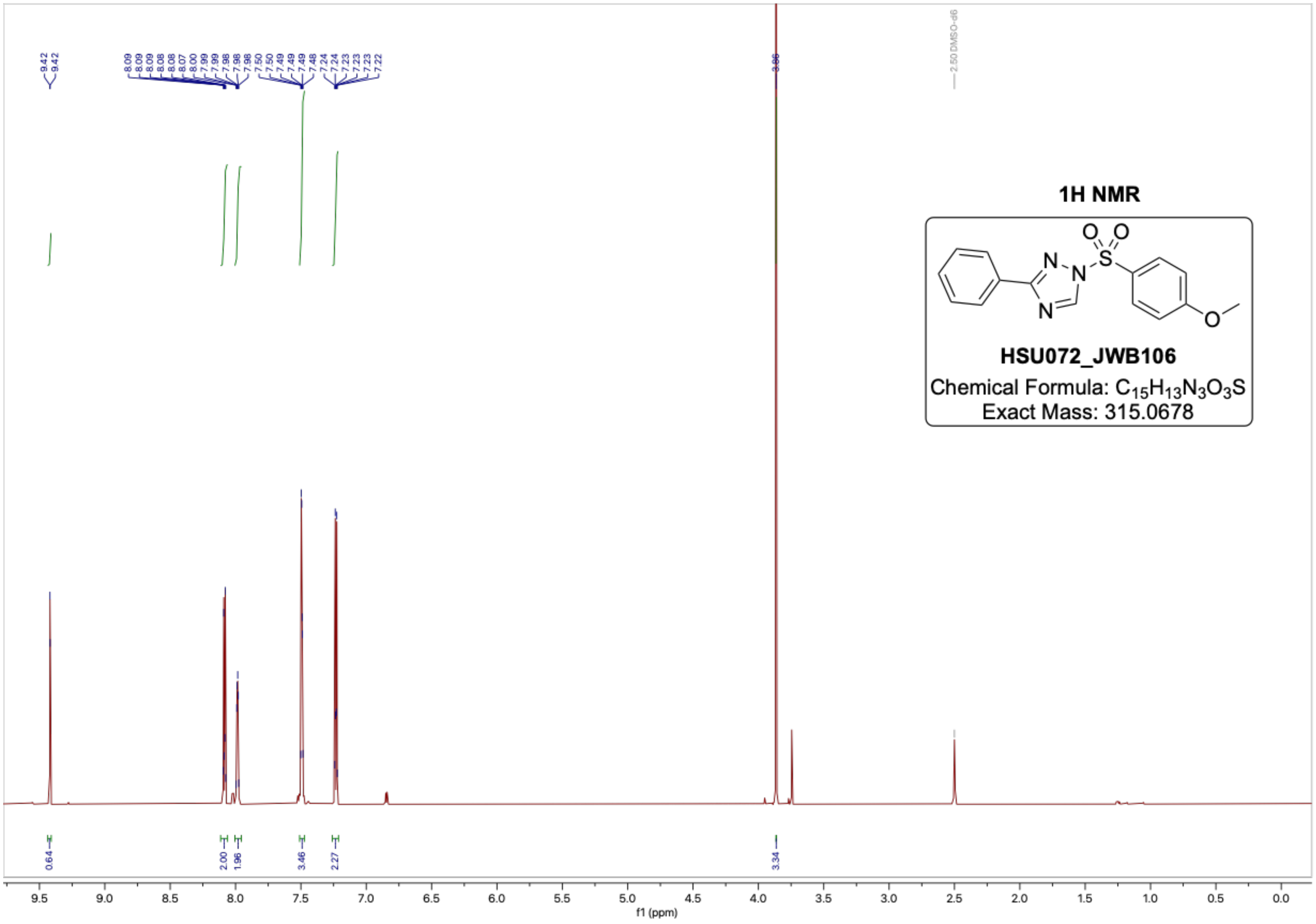

1H NMR
HSU072_JWB106
Chemical Formula: C15H13N3O3S
Exact Mass: 315.0678
f1 (ppm)
2.50 DMSO-d6

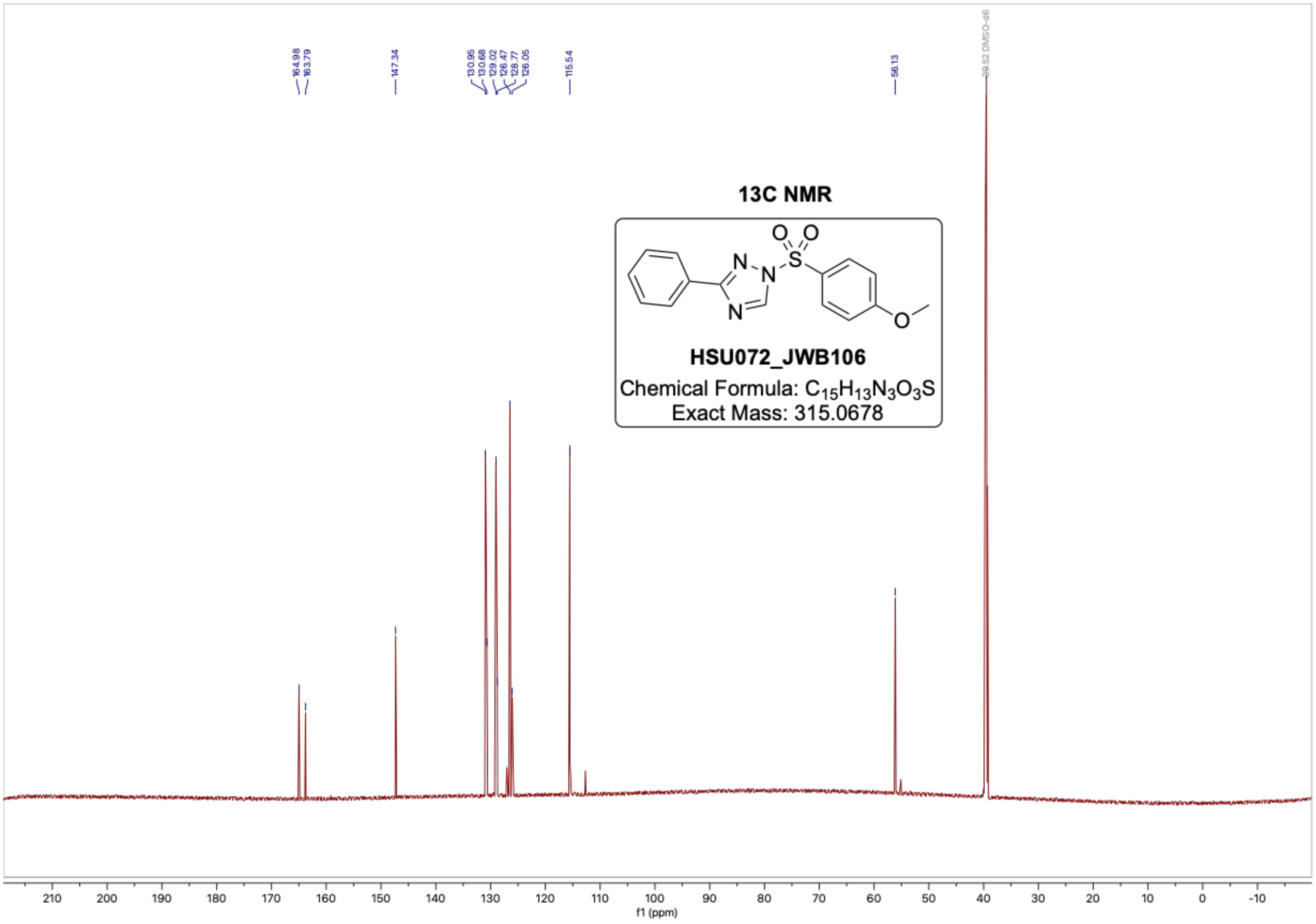
13C NMR
HSU072_JWB106
Chemical Formula: C15H13N3O3S
Exact Mass: 315.0678
164.98
163.79
147.34
130.95
130.68
129.02
126.47
128.77
126.05
115.54
56.13
39.52 DMSO-d6
f1 (ppm)

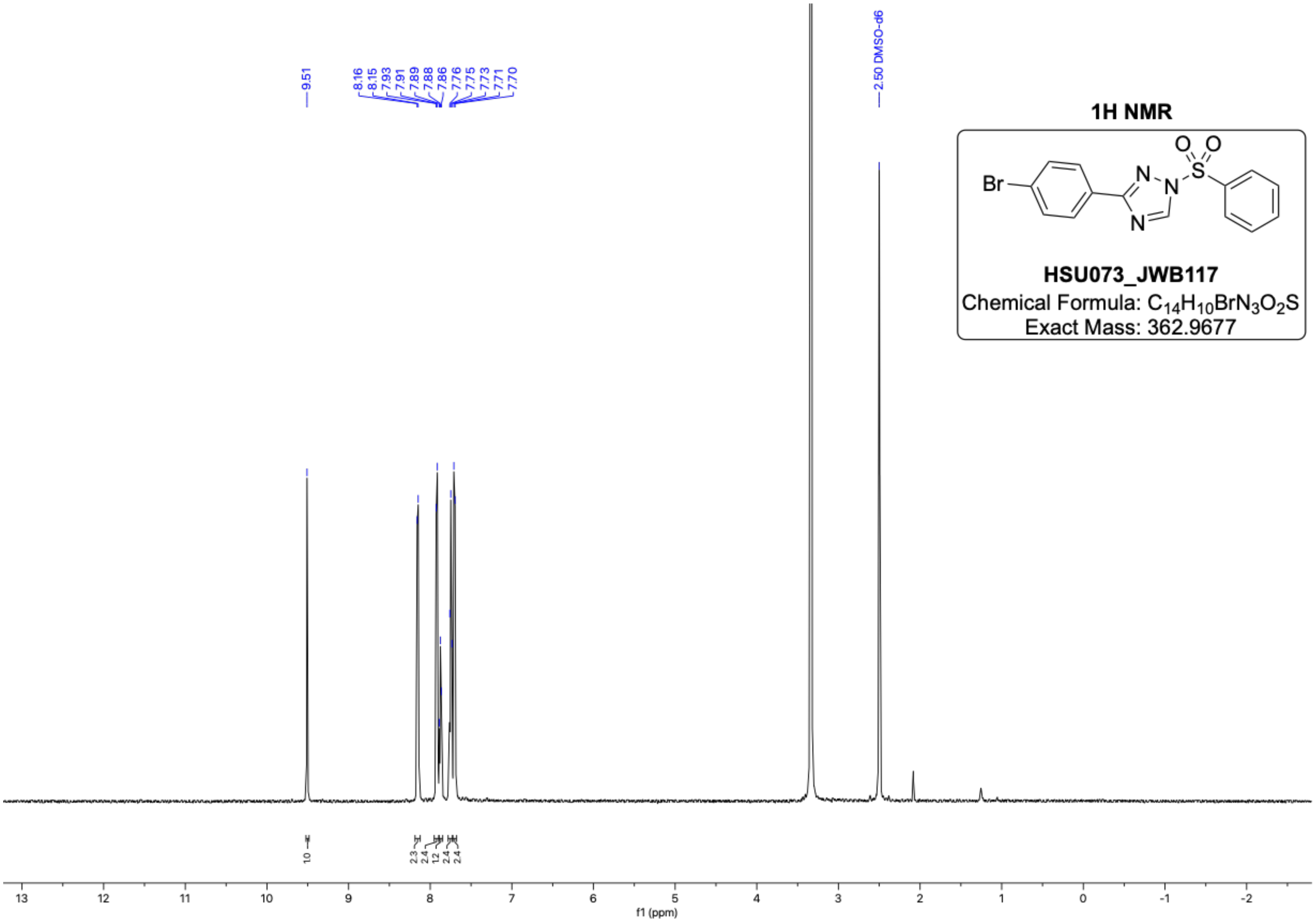

9.51
8.16
8.15
7.93
7.91
7.89
7.88
7.86
7.76
7.75
7.73
7.71
7.70
2.50 DMSO-d6
1H NMR
Br
N
N
N
S
O
O
HSU073_JWB117
Chemical Formula: C14H10BrN3O2S
Exact Mass: 362.9677
1.0
2.3
2.4
1.2
2.4
2.4
13
12
11
10
9
8
7
6
5
4
3
2
1
0
-1
-2
f1 (ppm)

13C NMR

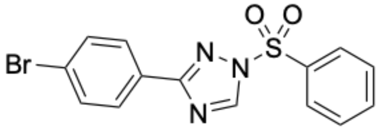

HSU073_JWB117

Chemical Formula: $C_{14}H_{10}BrN_3O_2S$

Exact Mass: 362.9677

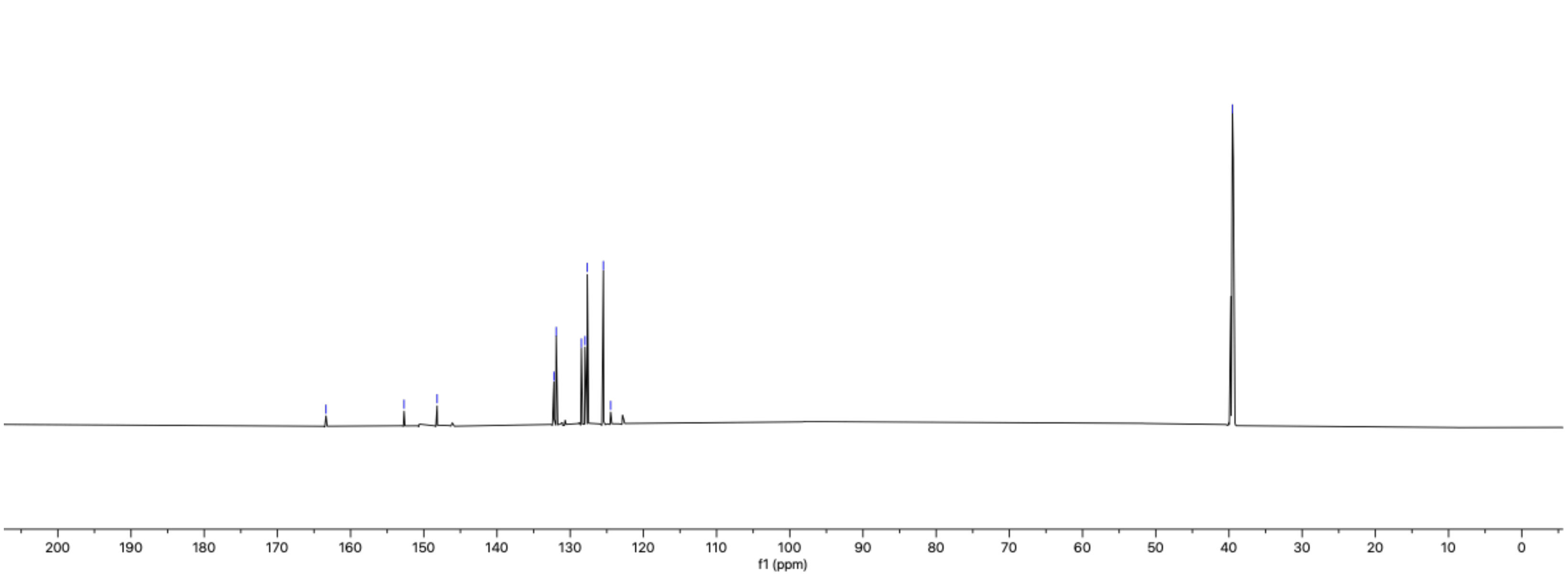

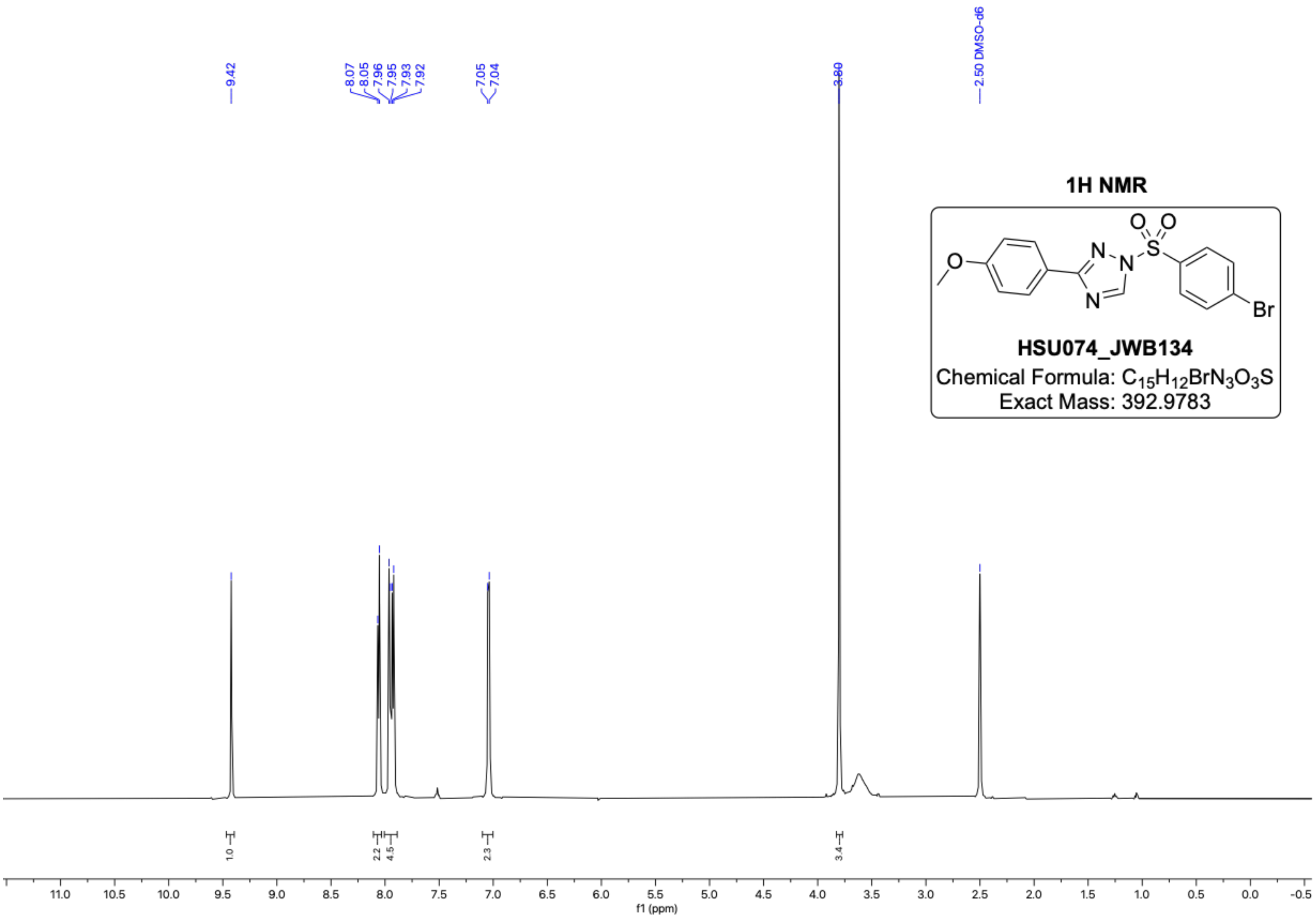
1H NMR
HSU074_JWB134
Chemical Formula: C15H12BrN3O3S
Exact Mass: 392.9783
9.42
8.07
8.05
7.96
7.95
7.93
7.92
7.05
7.04
3.80
2.50 DMSO-d6
1.0
2.2
4.5
2.3
3.4
f1 (ppm)

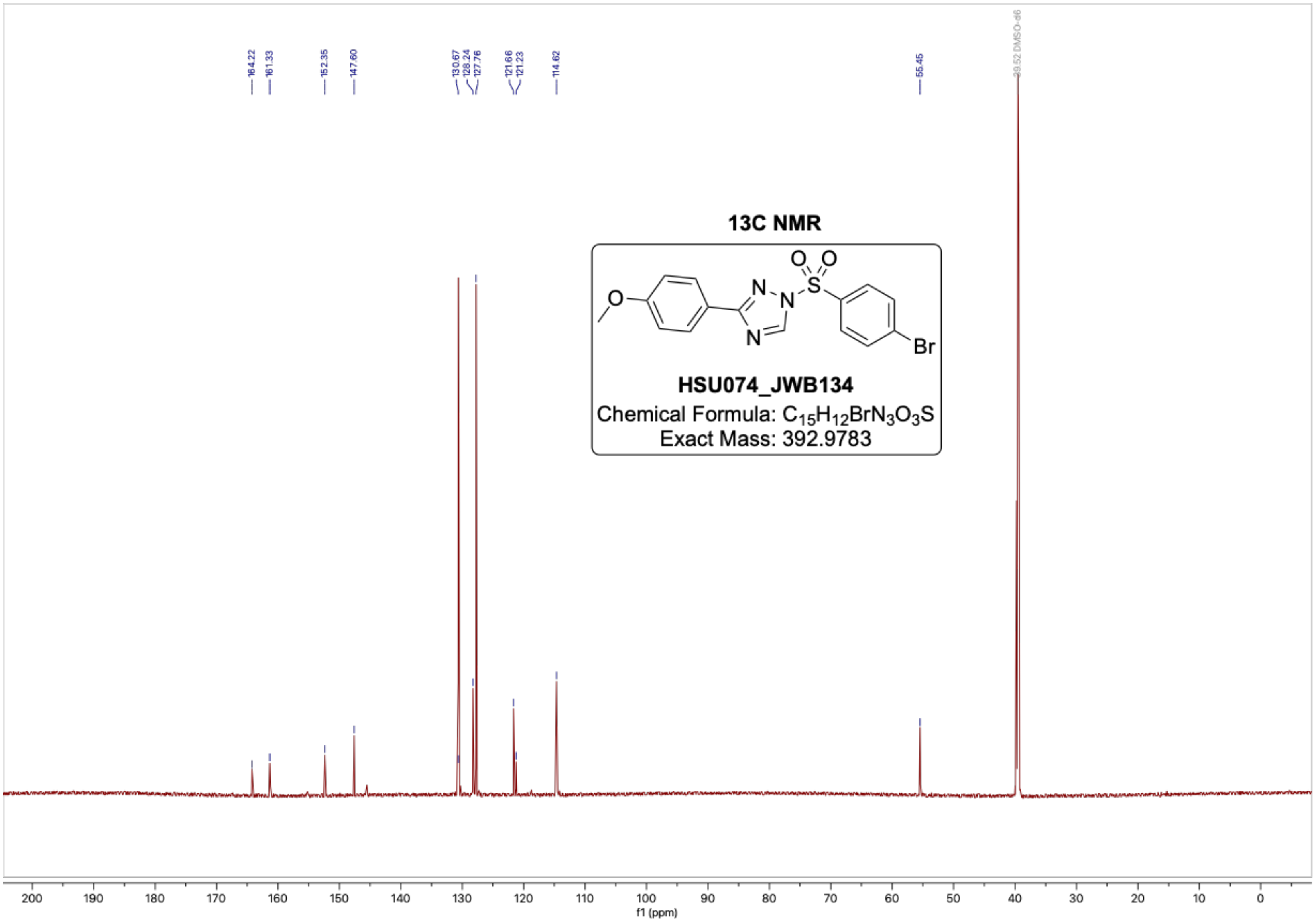

164.22
161.33
152.35
147.60
130.67
128.24
127.76
121.66
121.23
114.62
55.45
39.52 DMSO-d6
13C NMR
HSU074_JWB134
Chemical Formula: C15H12BrN3O3S
Exact Mass: 392.9783
f1 (ppm)

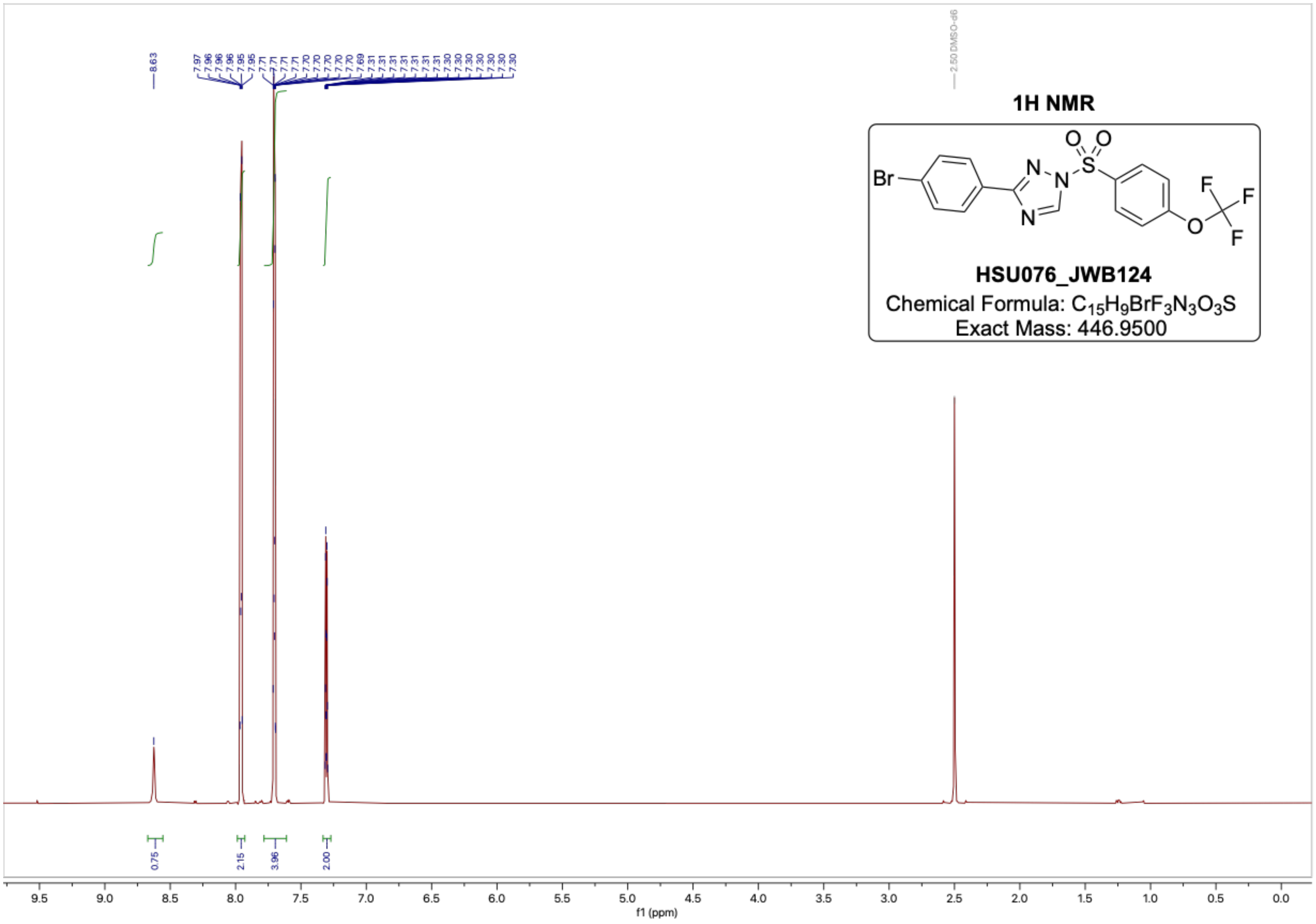

1H NMR
HSU076_JWB124
Chemical Formula: C15H9BrF3N3O3S
Exact Mass: 446.9500
8.63
7.97
7.96
7.96
7.96
7.95
7.95
7.71
7.71
7.71
7.71
7.70
7.70
7.70
7.70
7.69
7.31
7.31
7.31
7.31
7.31
7.31
7.30
7.30
7.30
7.30
7.30
7.30
2.50 DMSO-d6
0.75
2.15
3.96
2.00
f1 (ppm)

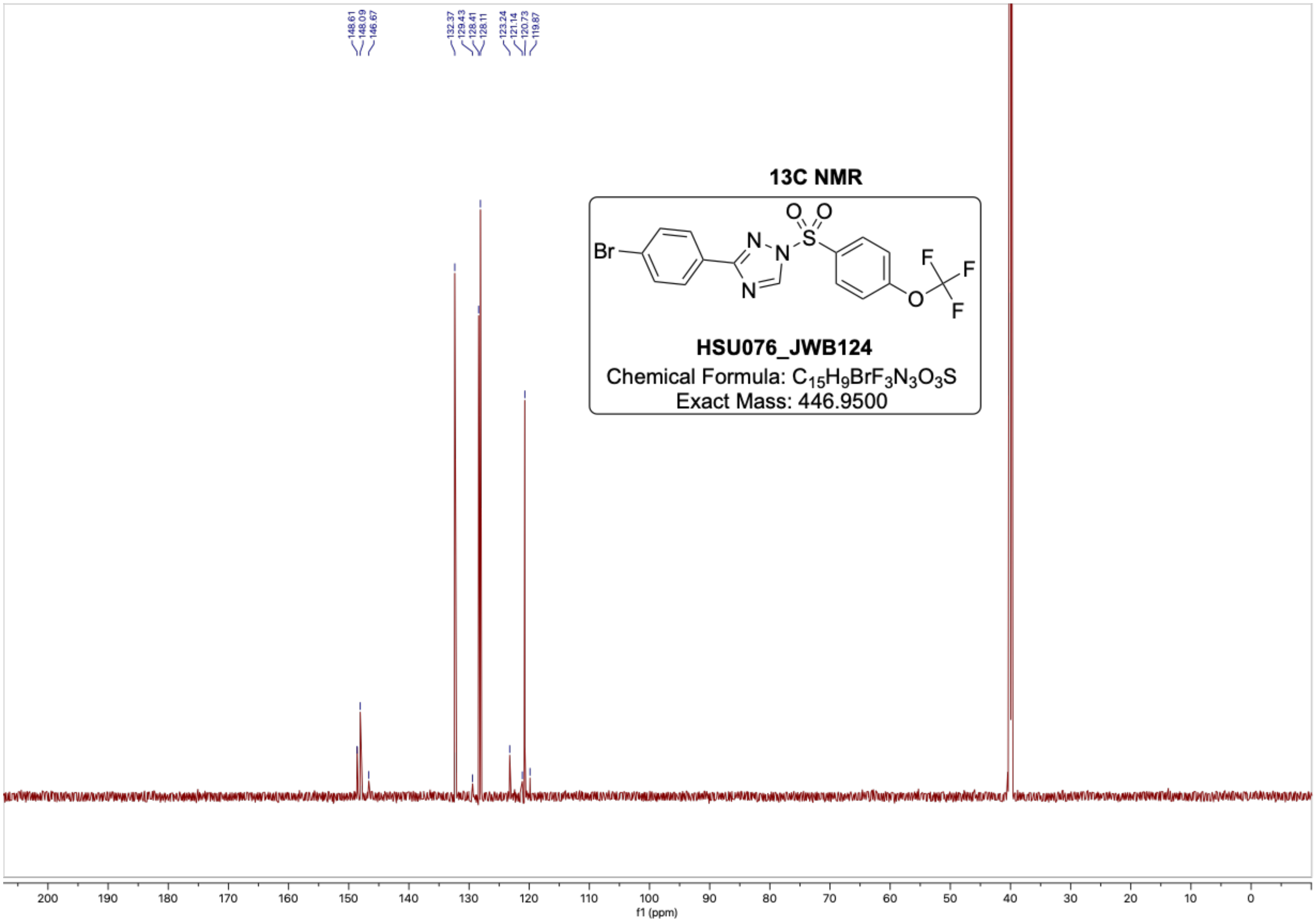

148.61
148.09
146.67
132.37
129.43
128.41
128.11
123.24
121.14
120.73
119.87
13C NMR
HSU076_JWB124
Chemical Formula: C15H9BrF3N3O3S
Exact Mass: 446.9500
f1 (ppm)

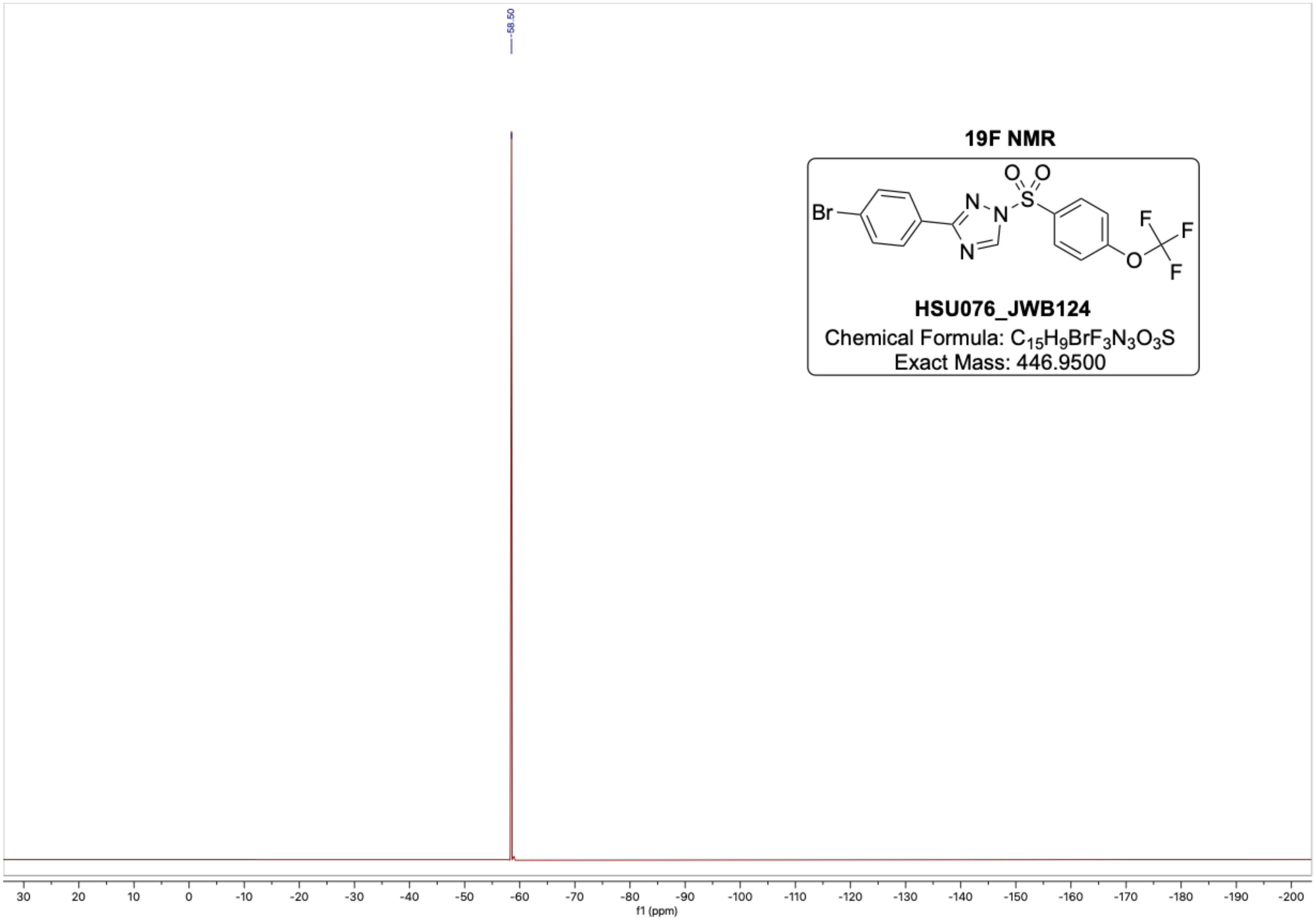
-58.50
19F NMR
Br
N
N
N
S
O O
O
F
F
F
HSU076_JWB124
Chemical Formula: $C_{15}H_9BrF_3N_3O_3S$
Exact Mass: 446.9500
30 20 10 0 -10 -20 -30 -40 -50 -60 -70 -80 -90 -100 -110 -120 -130 -140 -150 -160 -170 -180 -190 -200
f1 (ppm)

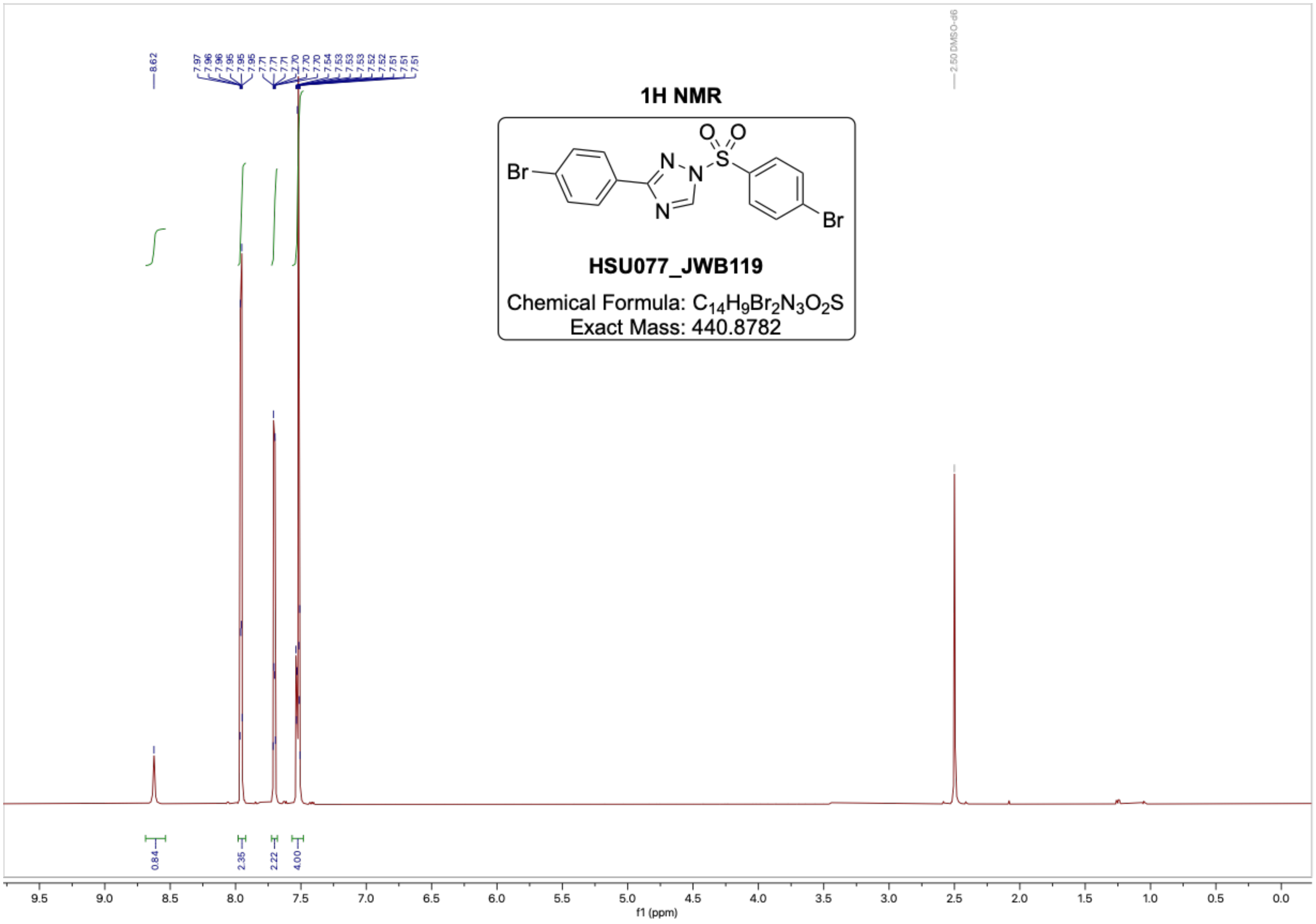
1H NMR
Br
N
N
N
O
O
S
Br
HSU077_JWB119
Chemical Formula: C14H9Br2N3O2S
Exact Mass: 440.8782
8.62
7.97
7.96
7.96
7.95
7.95
7.95
7.71
7.71
7.71
7.70
7.70
7.70
7.54
7.53
7.53
7.53
7.52
7.52
7.51
7.51
7.51
2.50 DMSO-d6
0.84
2.35
2.22
4.00
9.5
9.0
8.5
8.0
7.5
7.0
6.5
6.0
5.5
5.0
4.5
4.0
3.5
3.0
2.5
2.0
1.5
1.0
0.5
0.0
f1 (ppm)

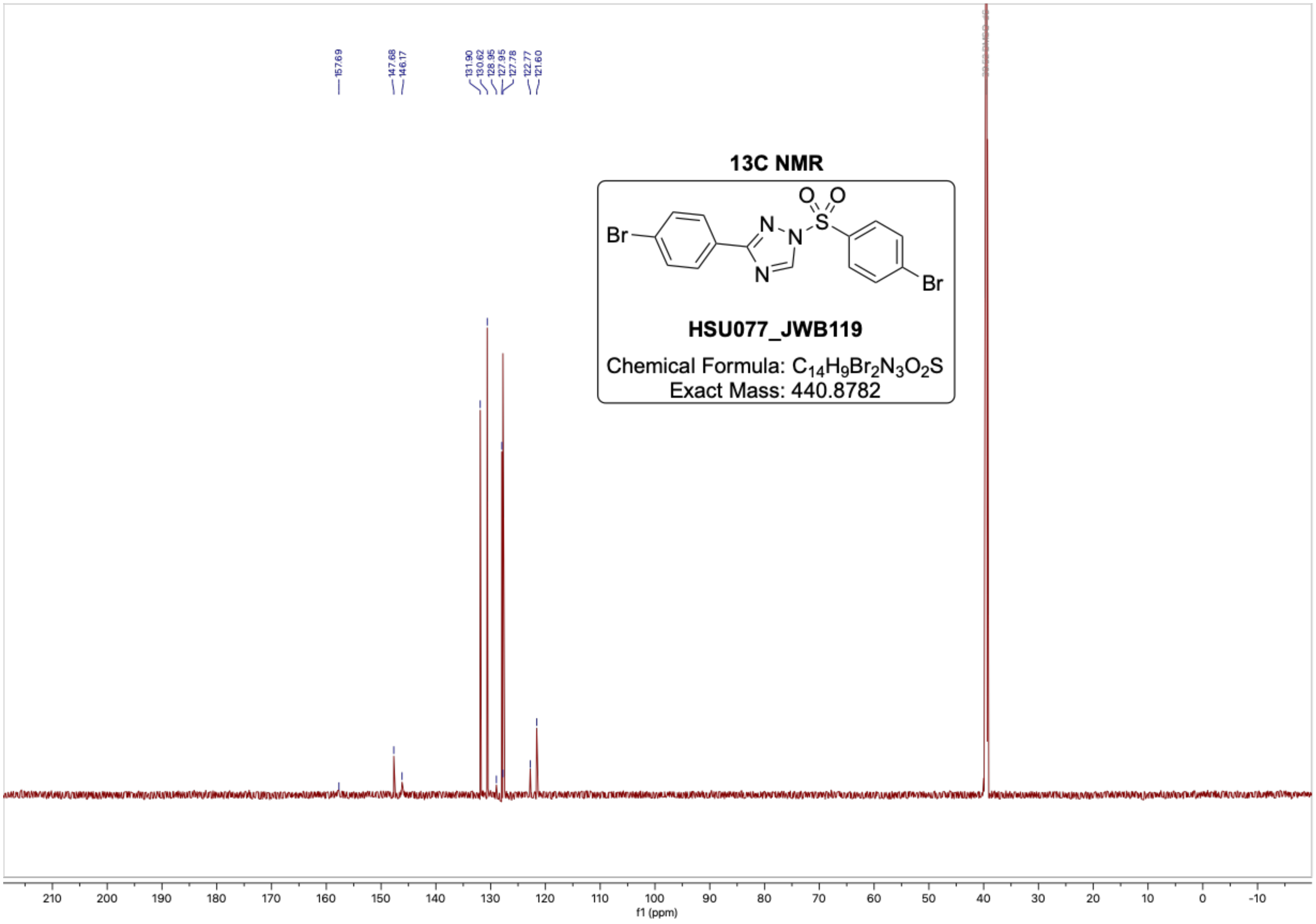

157.69
147.68
146.17
131.90
130.62
128.95
127.95
127.78
122.77
121.60
13C NMR
HSU077_JWB119
Chemical Formula: $C_{14}H_9Br_2N_3O_2S$
Exact Mass: 440.8782
210 200 190 180 170 160 150 140 130 120 110 100 90 80 70 60 50 40 30 20 10 0 -10
f1 (ppm)

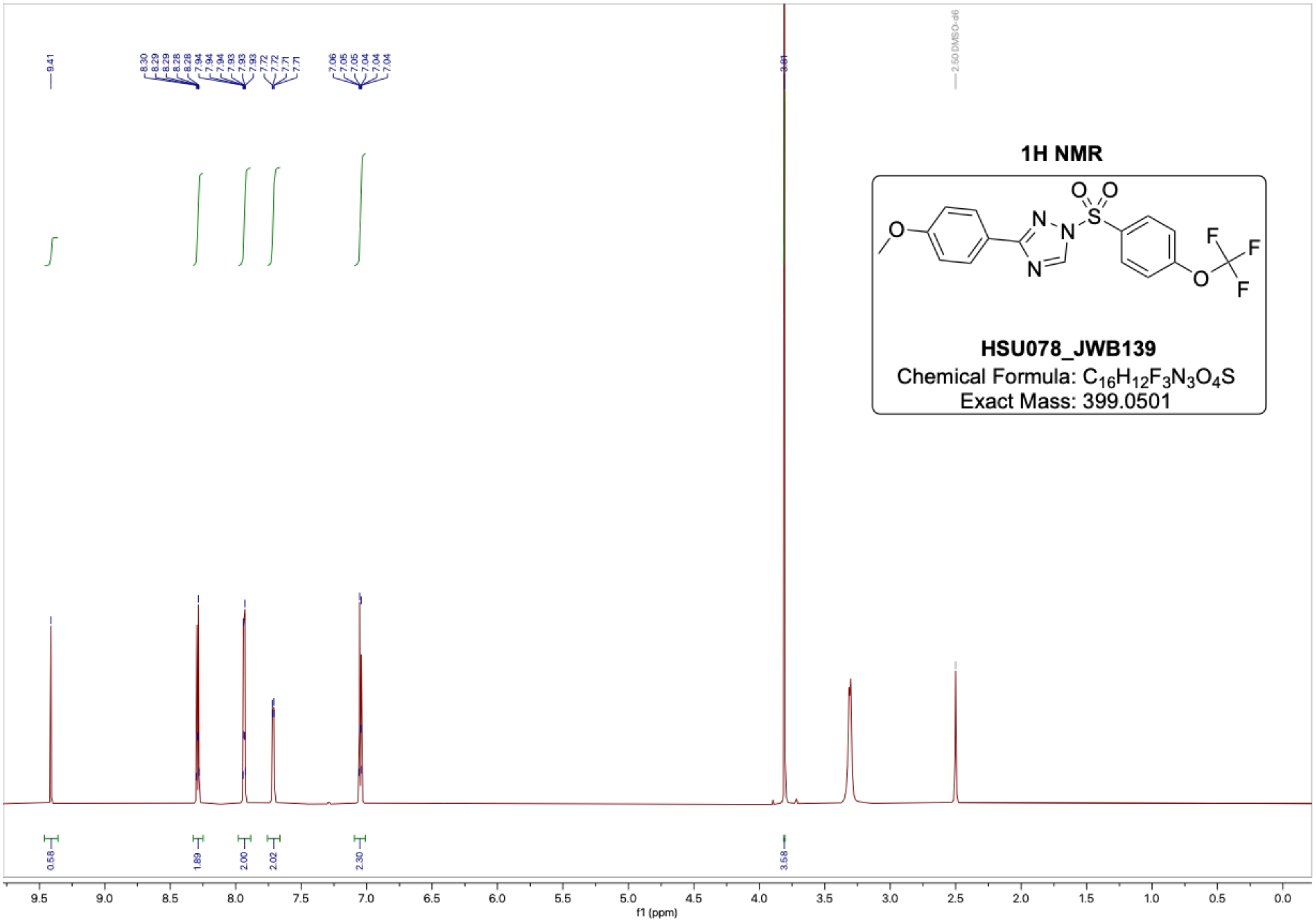
1H NMR
HSU078_JWB139
Chemical Formula: C16H12F3N3O4S
Exact Mass: 399.0501
f1 (ppm)
2.50 DMSO-d6

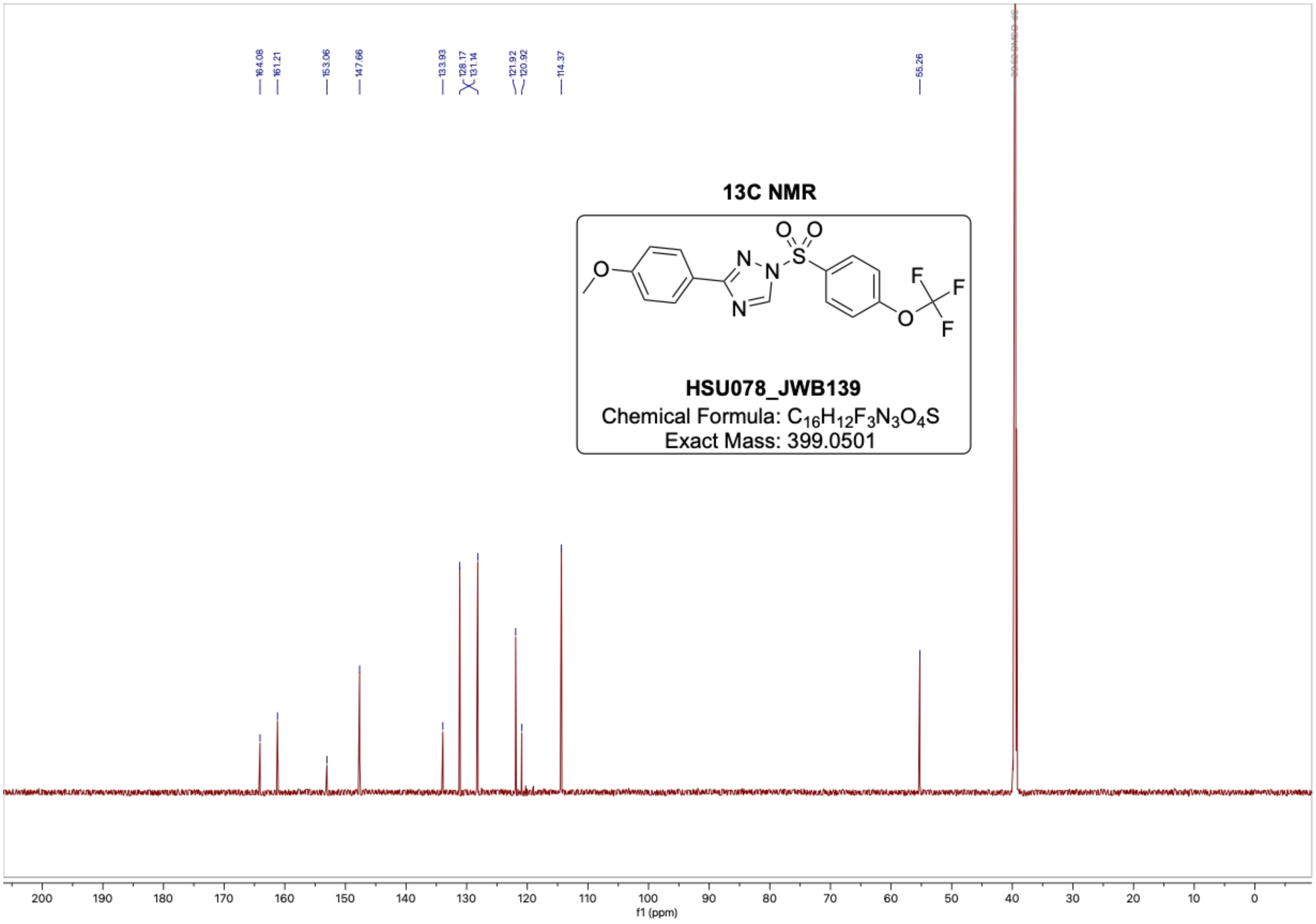

164.08
161.21
153.06
147.66
133.93
131.14
128.17
121.92
120.92
114.37
55.26
13C NMR
HSU078_JWB139
Chemical Formula: C16H12F3N3O4S
Exact Mass: 399.0501
f1 (ppm)

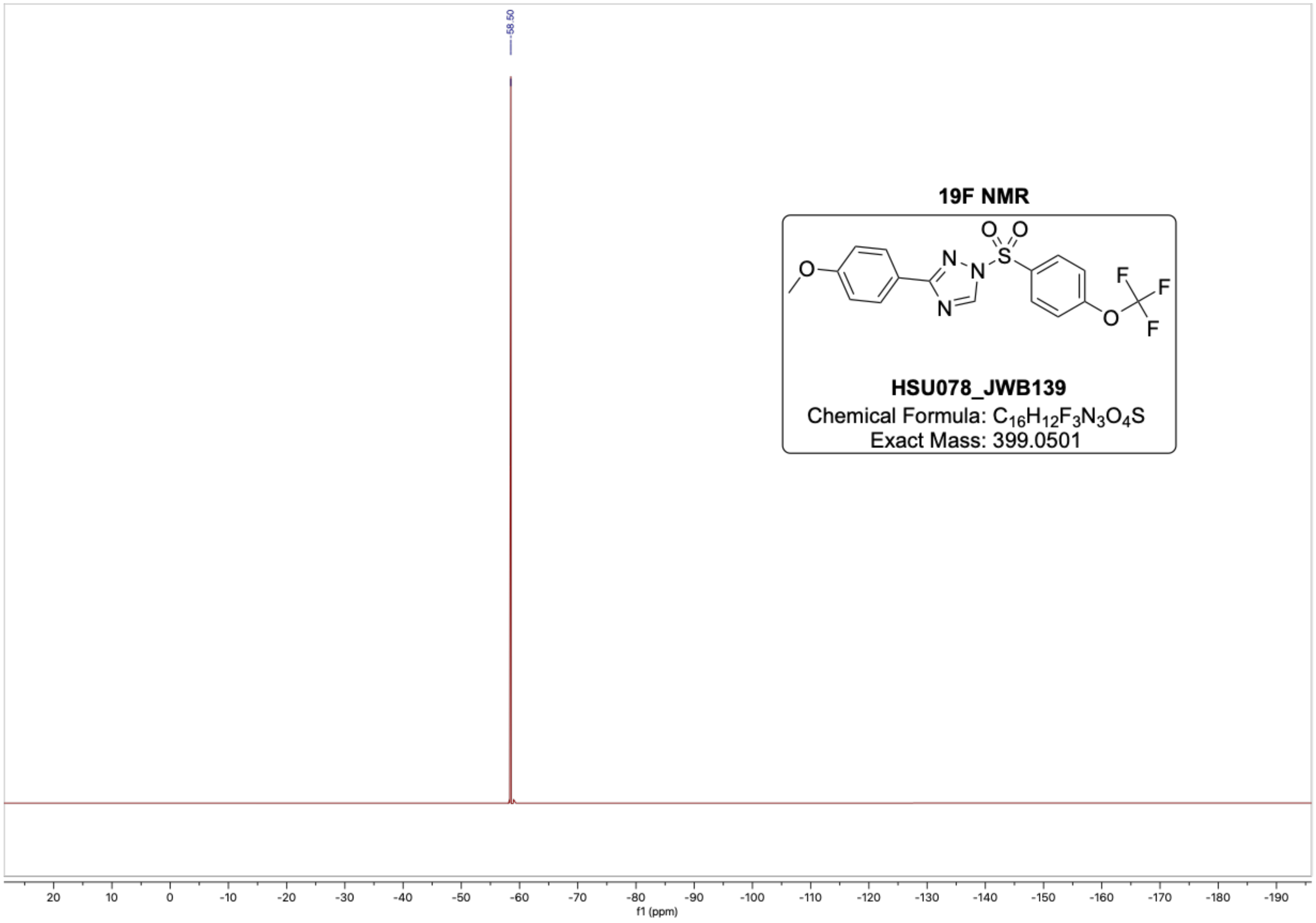

-58.50
19F NMR
HSU078_JWB139
Chemical Formula: C16H12F3N3O4S
Exact Mass: 399.0501
20
10
0
-10
-20
-30
-40
-50
-60
-70
-80
-90
-100
-110
-120
-130
-140
-150
-160
-170
-180
-190
f1 (ppm)

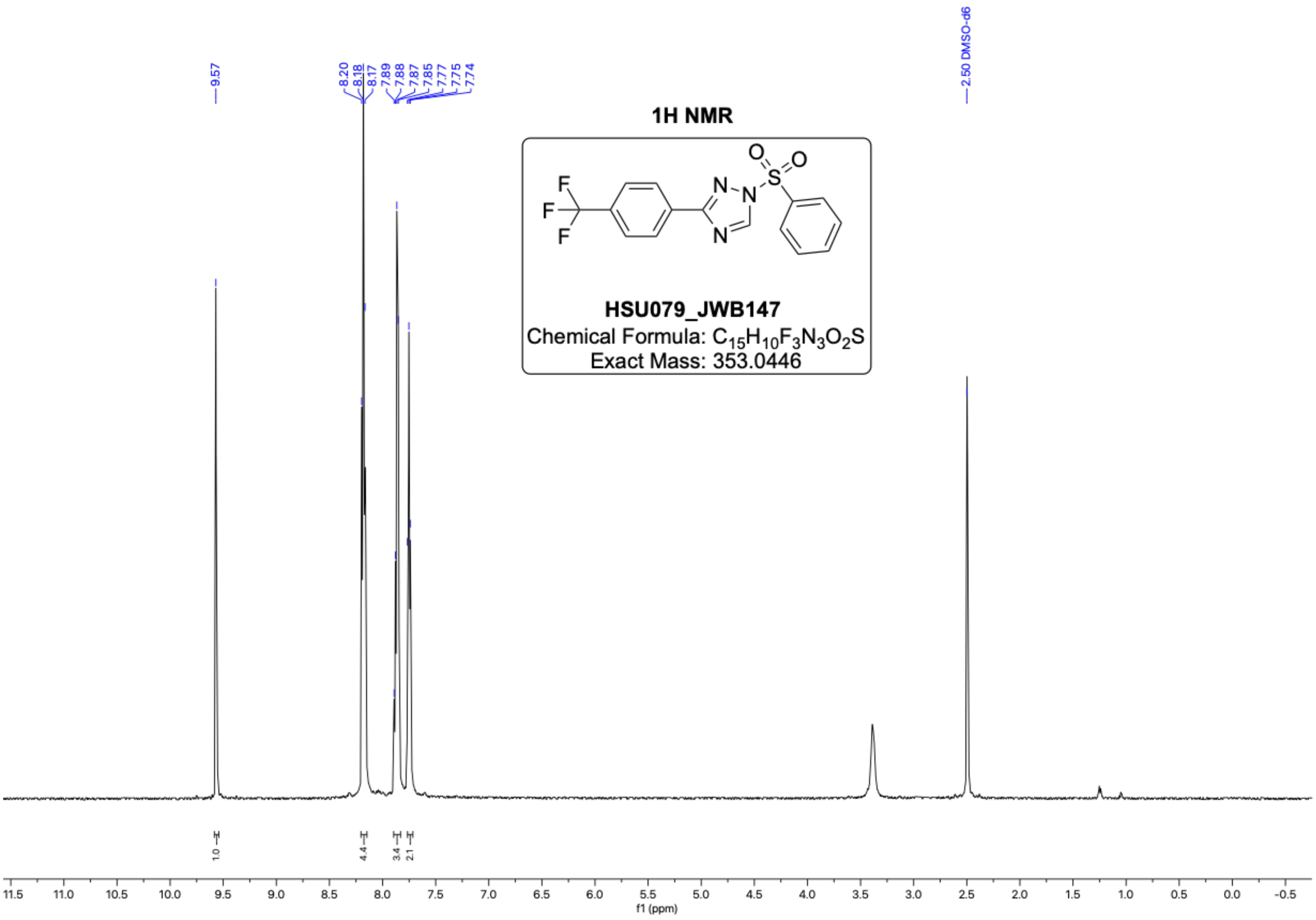
1H NMR
HSU079_JWB147
Chemical Formula: C15H10F3N3O2S
Exact Mass: 353.0446
9.57
8.20
8.18
8.17
7.89
7.88
7.87
7.85
7.77
7.75
7.74
2.50 DMSO-d6
1.0
4.4
3.4
2.1
f1 (ppm)

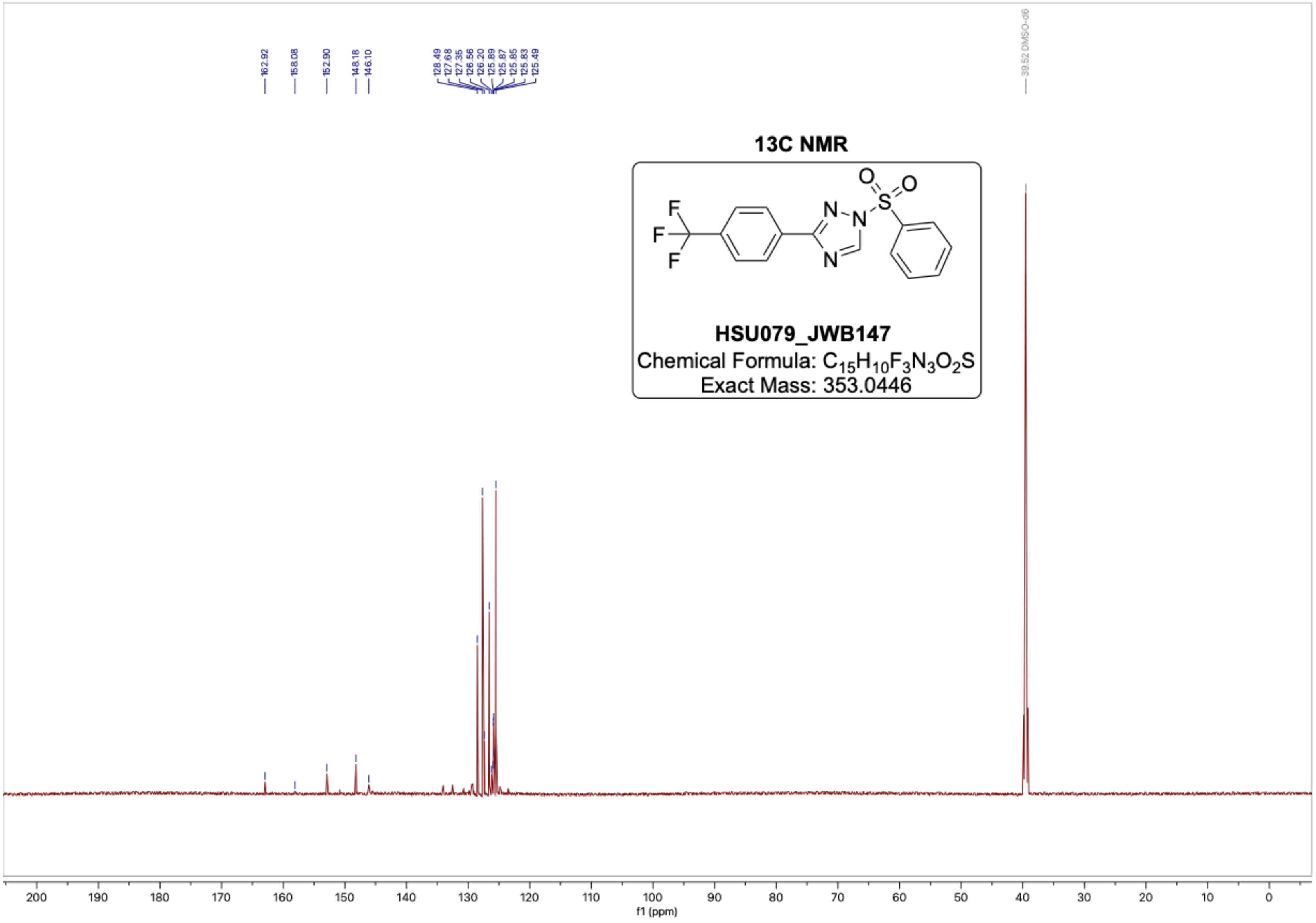
162.92
158.08
152.90
148.18
146.10
128.49
127.68
127.35
126.56
126.20
125.89
125.87
125.85
125.83
125.49
39.52 DMSO-d6
13C NMR
HSU079_JWB147
Chemical Formula: C15H10F3N3O2S
Exact Mass: 353.0446
f1 (ppm)

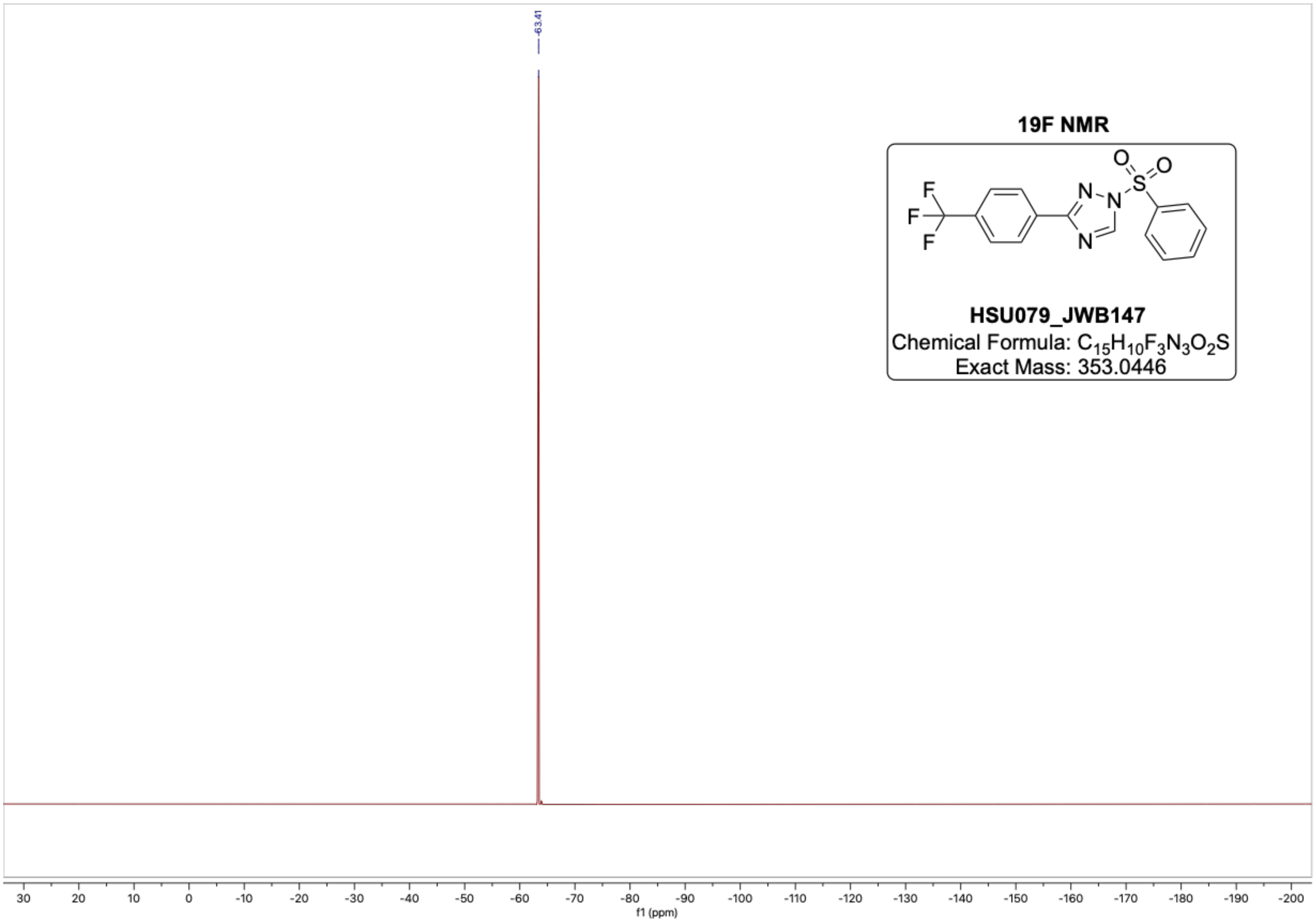

-63.41
19F NMR
HSU079_JWB147
Chemical Formula: $C_{15}H_{10}F_3N_3O_2S$
Exact Mass: 353.0446
30 20 10 0 -10 -20 -30 -40 -50 -60 -70 -80 -90 -100 -110 -120 -130 -140 -150 -160 -170 -180 -190 -200
f1 (ppm)

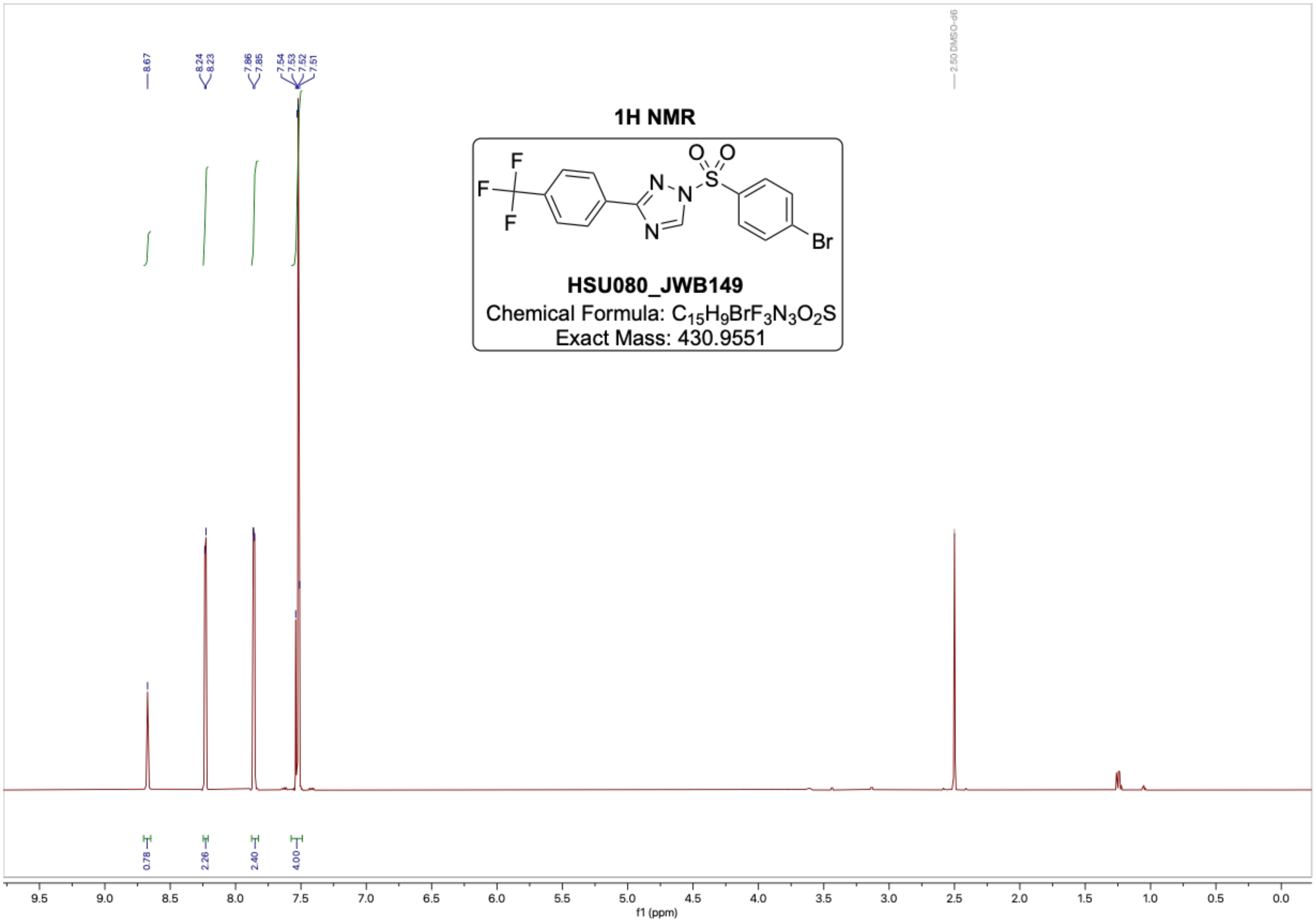
1H NMR
HSU080_JWB149
Chemical Formula: C15H9BrF3N3O2S
Exact Mass: 430.9551
8.67
8.24
8.23
7.86
7.85
7.54
7.53
7.52
7.51
2.50 DMSO-d6
0.78
2.26
2.40
4.00
9.5
9.0
8.5
8.0
7.5
7.0
6.5
6.0
5.5
5.0
4.5
4.0
3.5
3.0
2.5
2.0
1.5
1.0
0.5
0.0
f1 (ppm)

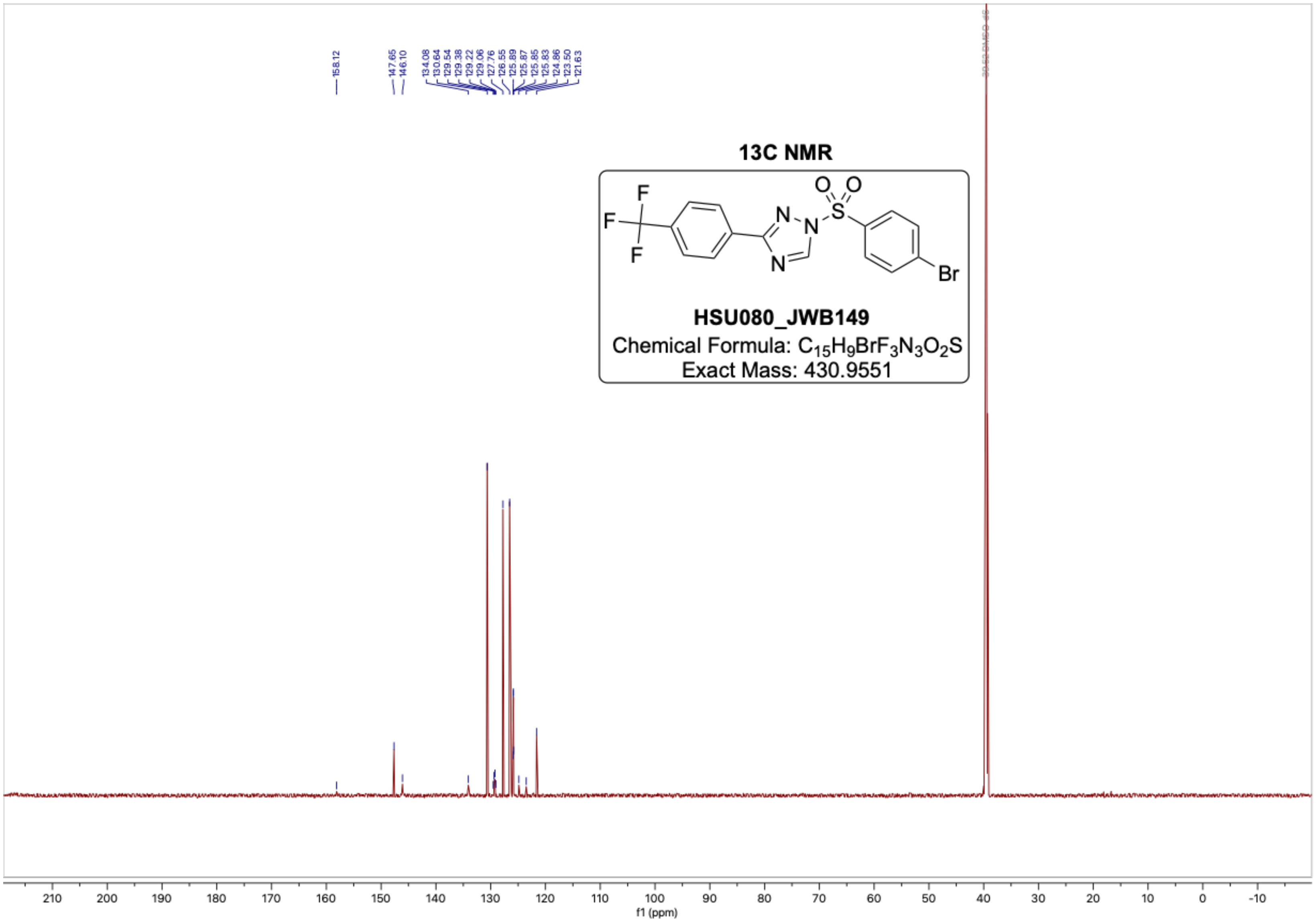
158.12
147.65
146.10
134.08
130.64
129.54
129.38
129.22
129.06
127.76
126.55
125.89
125.87
125.85
125.83
124.86
123.50
121.63
13C NMR
F
F
F
N
N
N
O
O
S
Br
HSU080_JWB149
Chemical Formula: C15H9BrF3N3O2S
Exact Mass: 430.9551
210 200 190 180 170 160 150 140 130 120 110 100 90 80 70 60 50 40 30 20 10 0 -10
f1 (ppm)

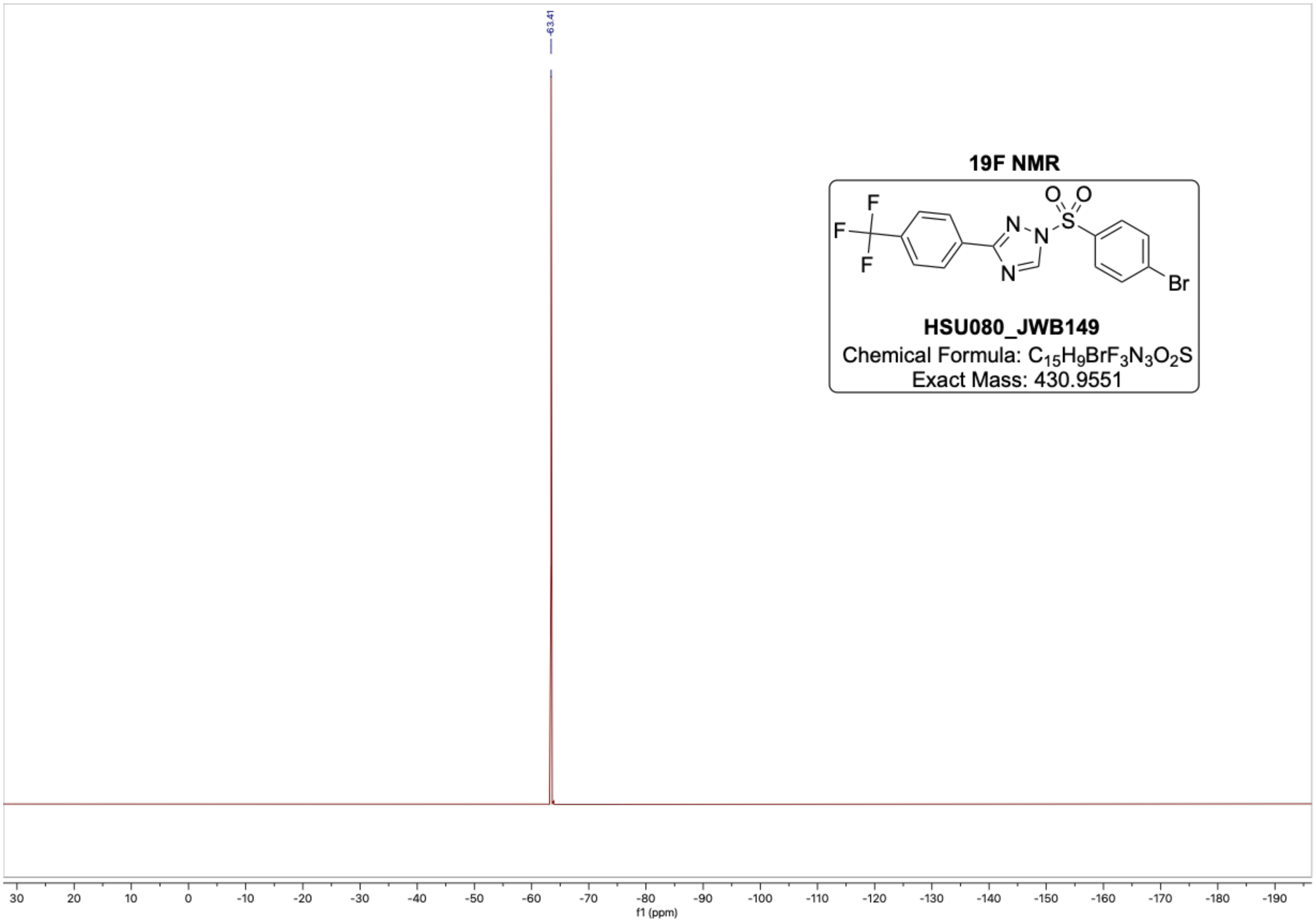
-63.41
19F NMR
HSU080_JWB149
Chemical Formula: C15H9BrF3N3O2S
Exact Mass: 430.9551
f1 (ppm)

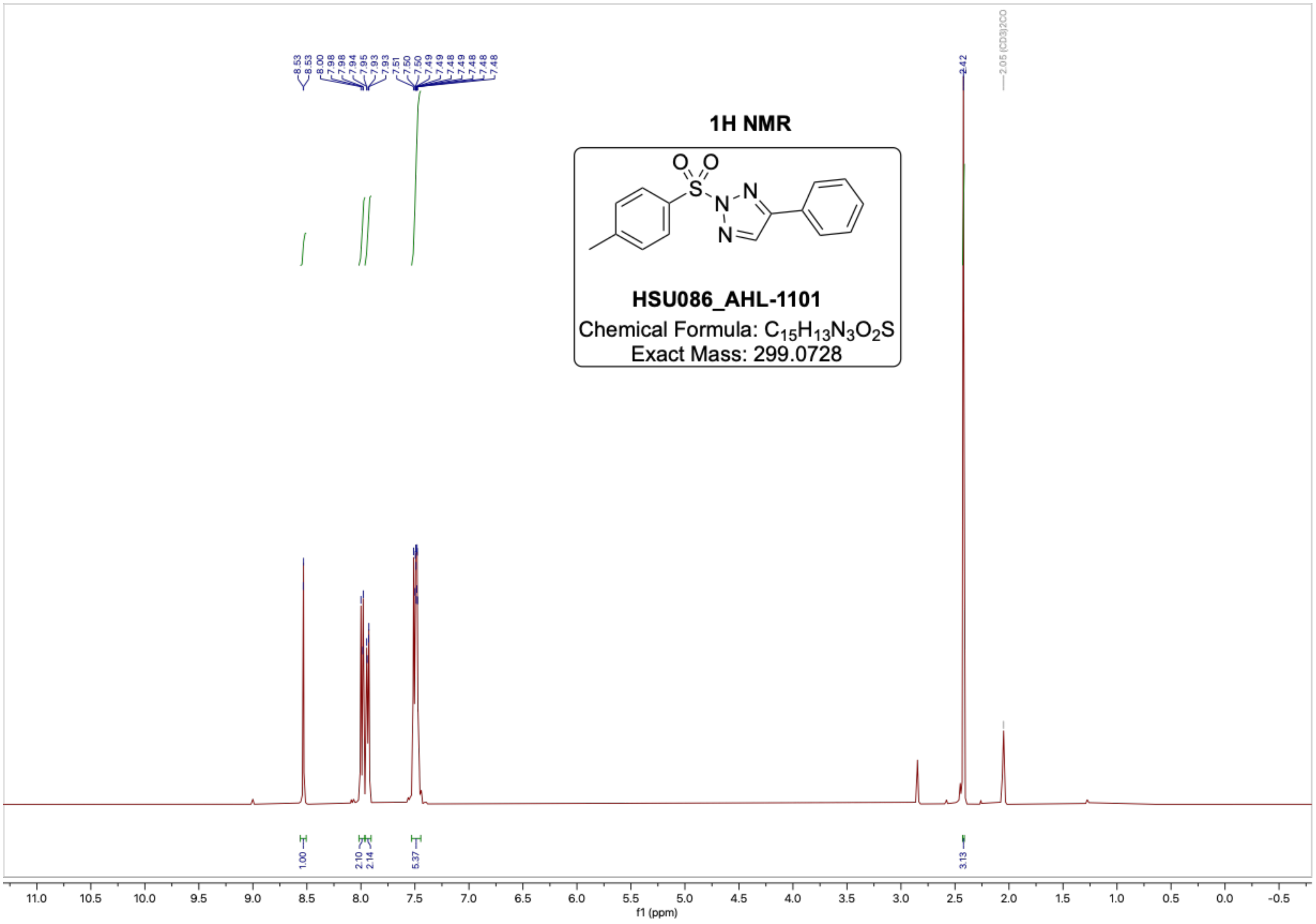
1H NMR
HSU086_AHL-1101
Chemical Formula: $C_{15}H_{13}N_3O_2S$
Exact Mass: 299.0728
f1 (ppm)
2.05 (CD3)2CO
2.42
1.00
2.10
2.14
5.37
3.13

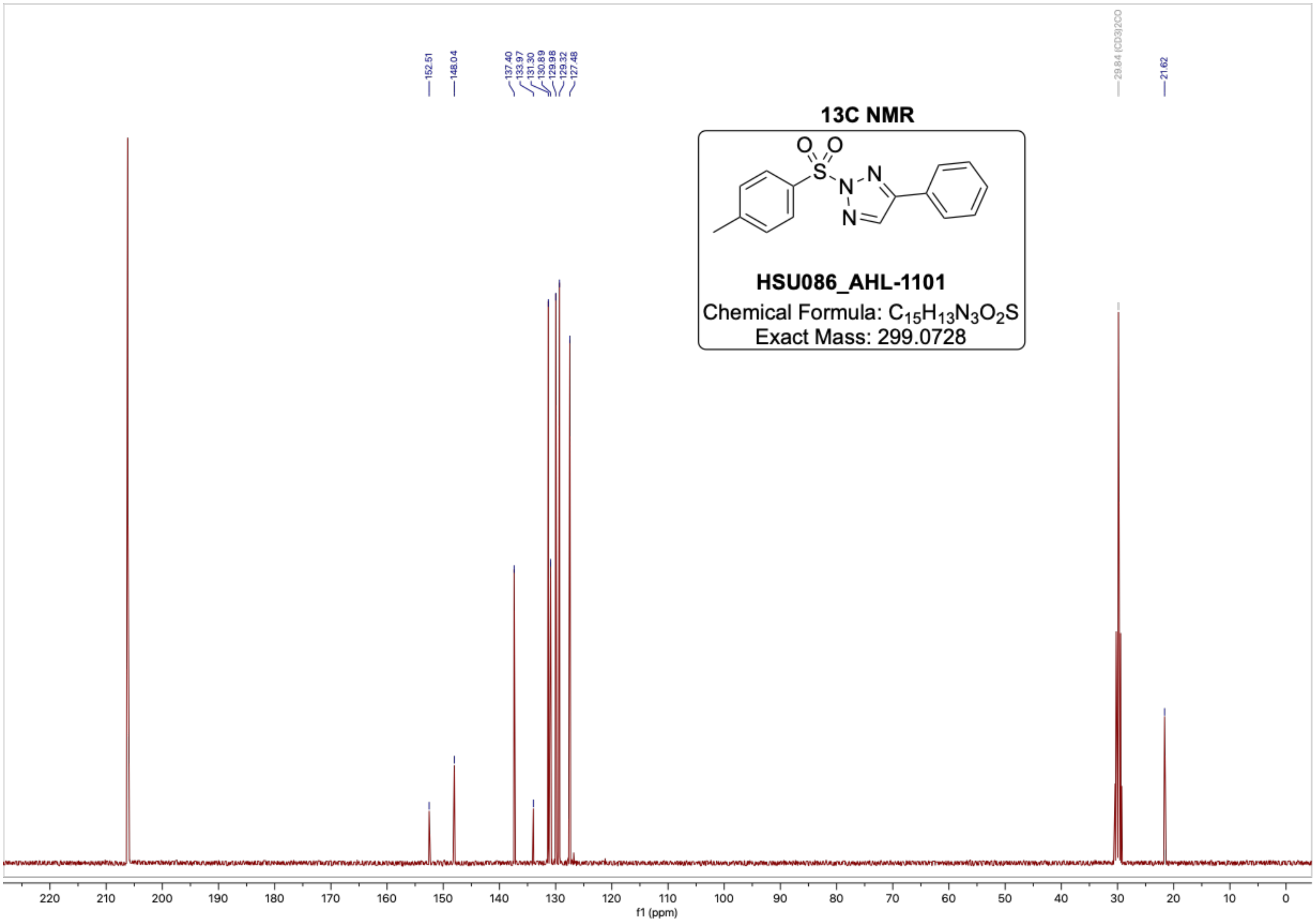
13C NMR
HSU086_AHL-1101
Chemical Formula: C15H13N3O2S
Exact Mass: 299.0728
152.51
148.04
137.40
133.97
131.30
130.89
129.98
129.32
127.48
29.84 (CD3)2CO
21.62
f1 (ppm)

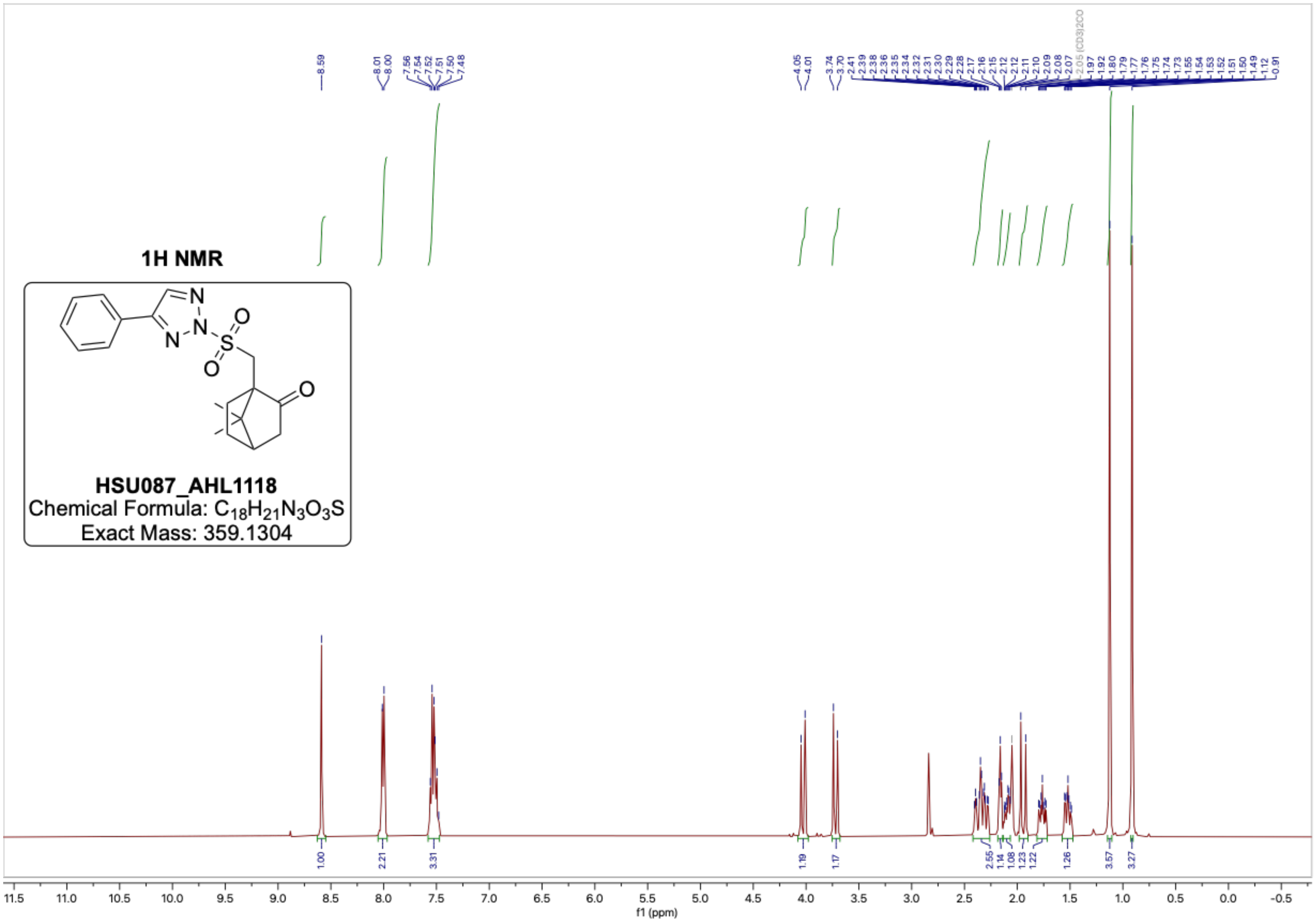

1H NMR
HSU087_AHL1118
Chemical Formula: C18H21N3O3S
Exact Mass: 359.1304
f1 (ppm)

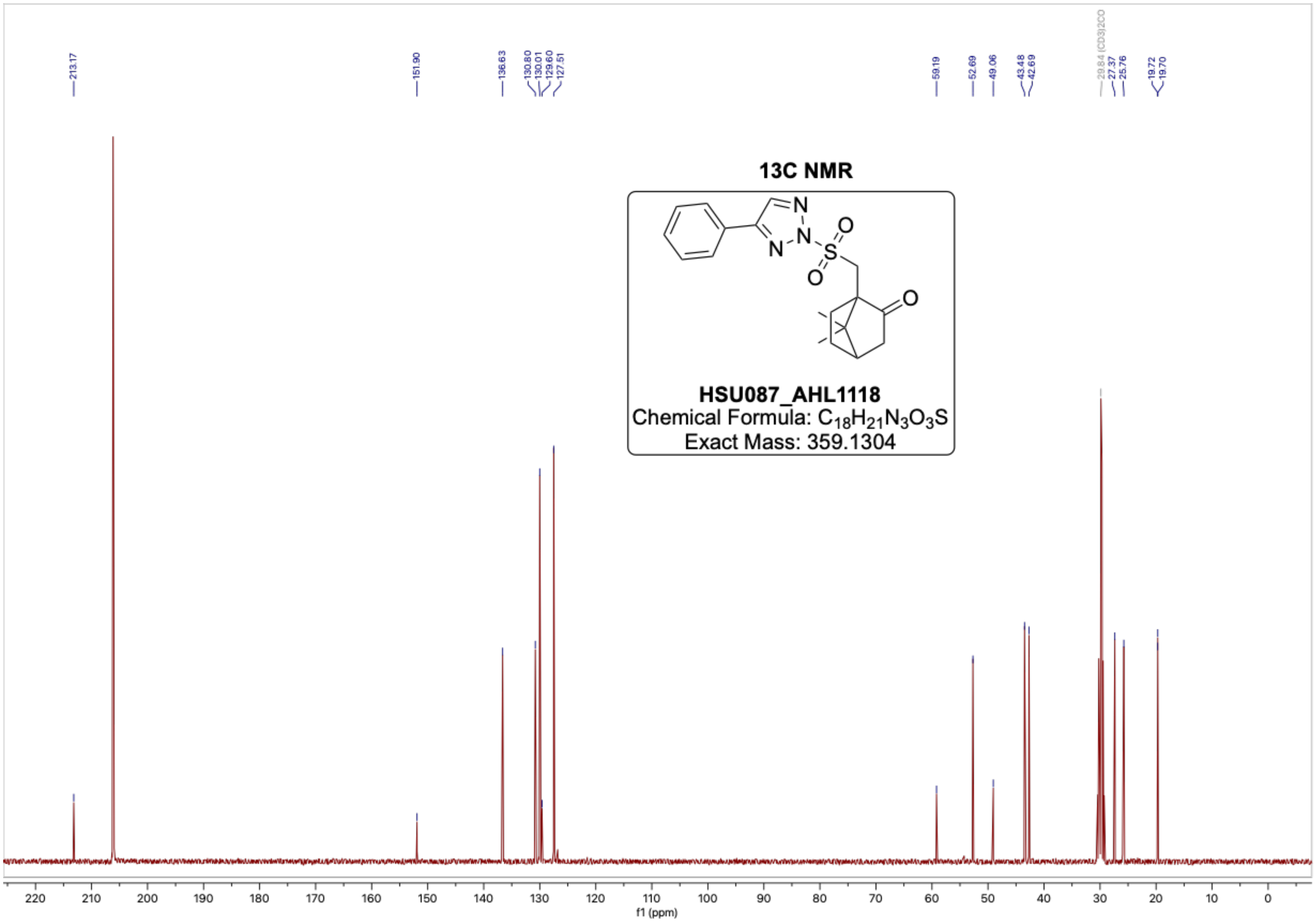

213.17
151.90
136.63
130.80
130.01
129.60
127.51
59.19
52.69
49.06
43.48
42.69
29.84 (CD3)2CO
27.37
25.76
19.72
19.70
13C NMR
HSU087_AHL1118
Chemical Formula: C18H21N3O3S
Exact Mass: 359.1304
f1 (ppm)

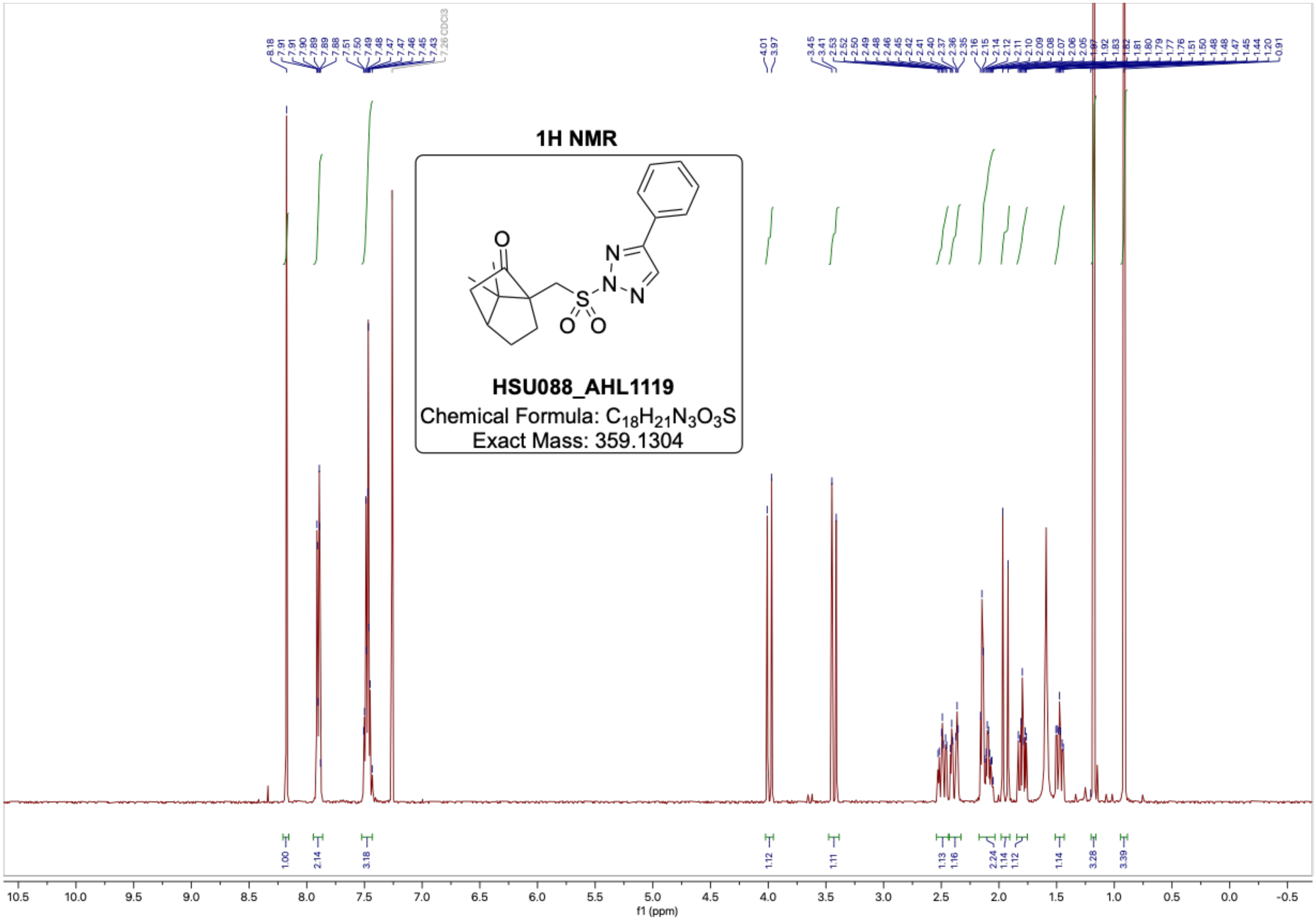

1H NMR
HSU088_AHL1119
Chemical Formula: C18H21N3O3S
Exact Mass: 359.1304
f1 (ppm)

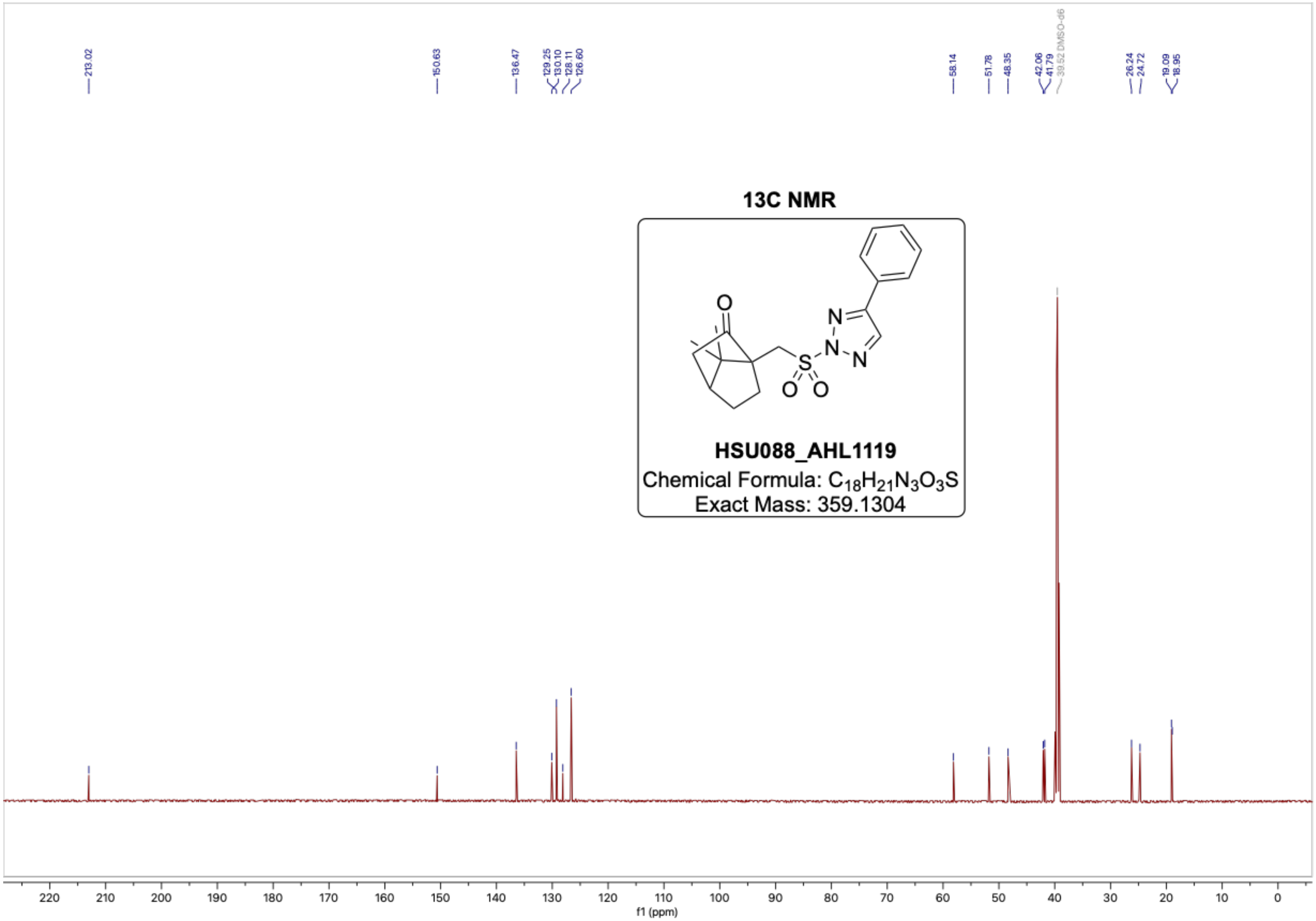
213.02
150.63
136.47
129.25
130.10
128.11
126.60
58.14
51.78
48.35
42.06
41.79
39.52 DMSO-d6
26.24
24.72
19.09
18.95
13C NMR
O
S
O
O
N
N
N
HSU088_AHL1119
Chemical Formula: C18H21N3O3S
Exact Mass: 359.1304
220
210
200
190
180
170
160
150
140
130
120
110
100
90
80
70
60
50
40
30
20
10
0
f1 (ppm)

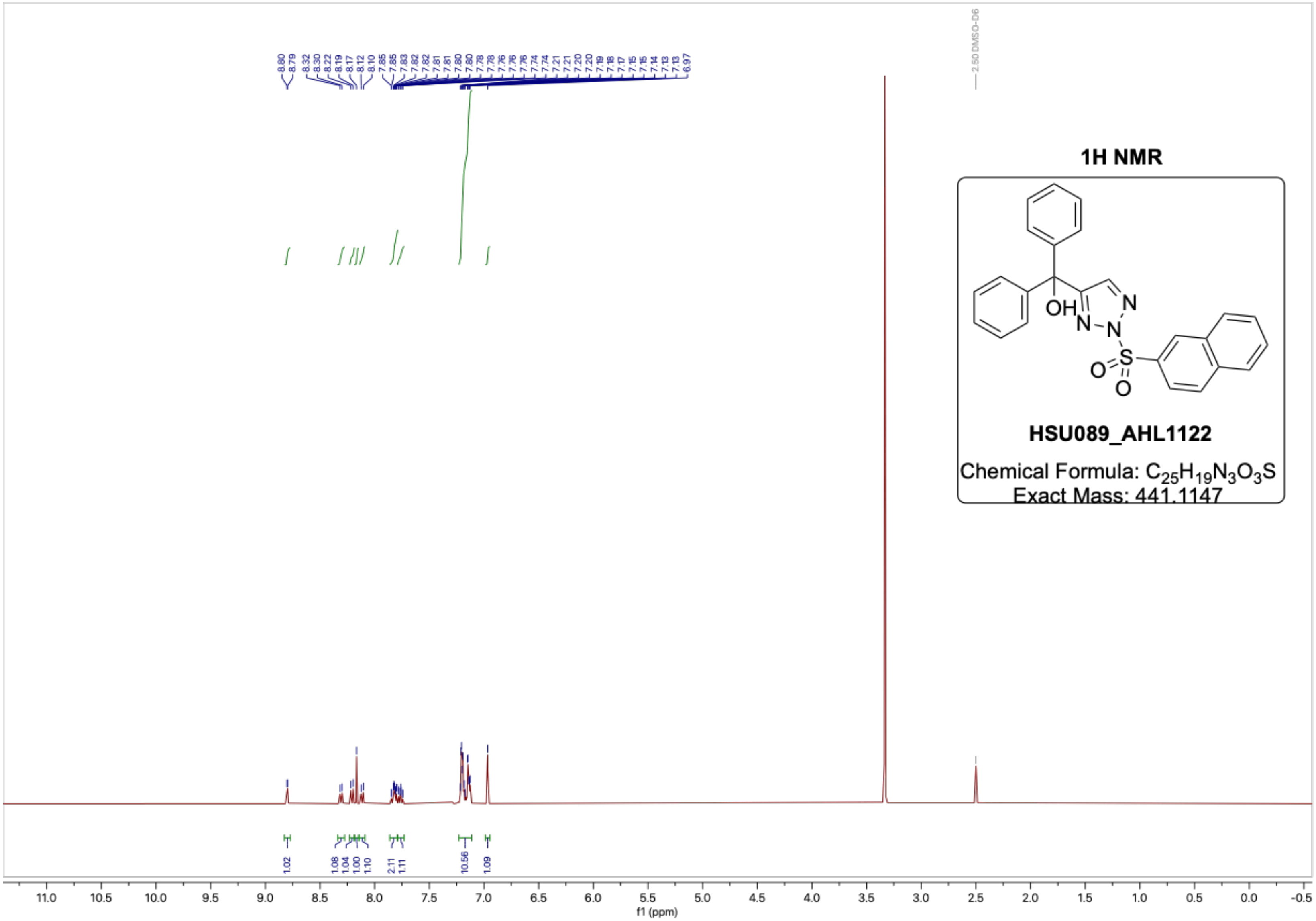
1H NMR
OH
N
N
N
S
O
O
HSU089_AHL1122
Chemical Formula: $C_{25}H_{19}N_3O_3S$
Exact Mass: 441.1147
2.50 DMSO-D6
1.02
1.08
1.04
1.00
1.10
2.11
1.11
10.56
1.09
f1 (ppm)

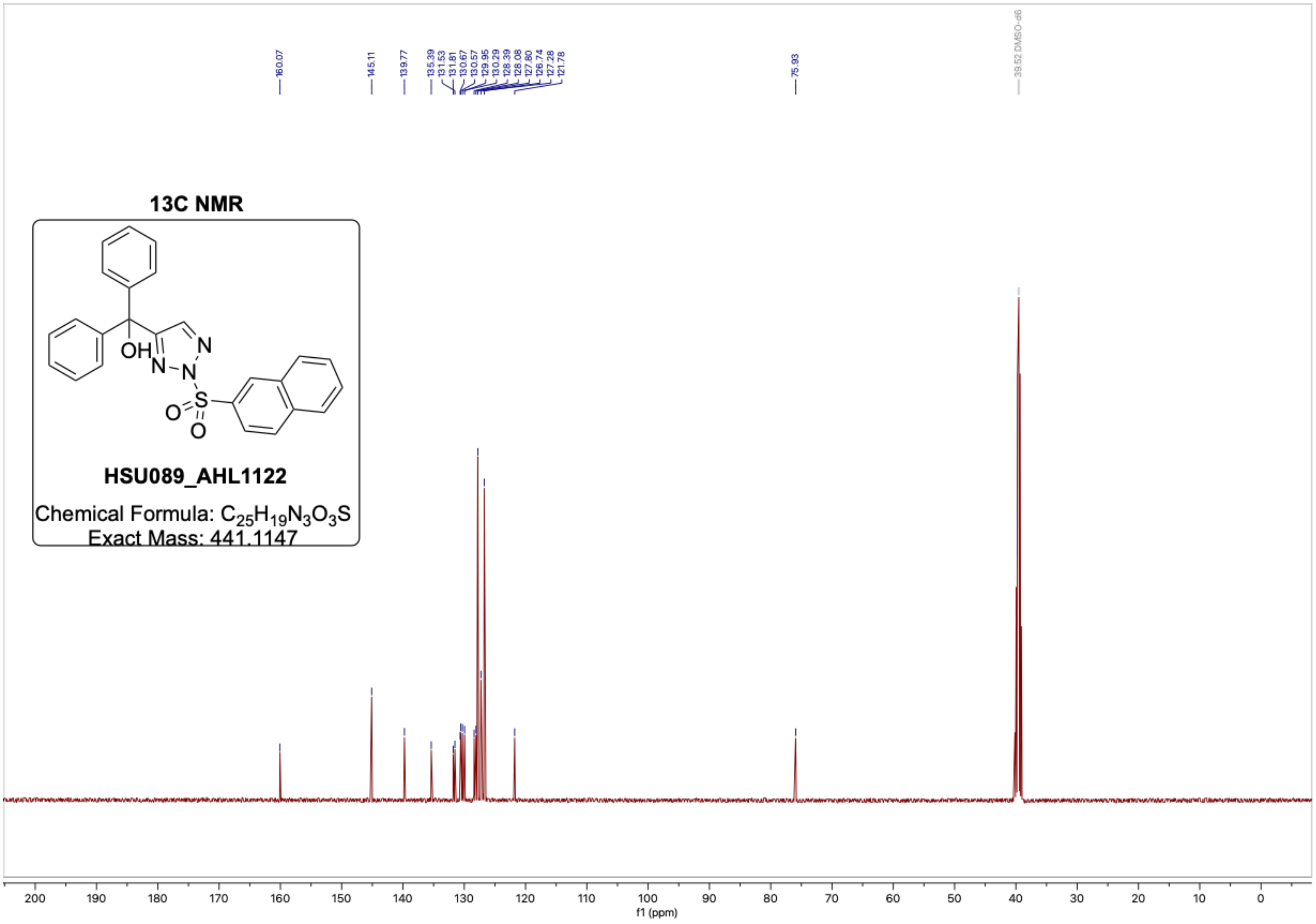

13C NMR
OH
N
N
N
S
O
O
HSU089_AHL1122
Chemical Formula: $C_{25}H_{19}N_3O_3S$
Exact Mass: 441.1147
160.07
145.11
139.77
135.39
131.53
131.81
130.67
130.57
129.95
130.29
128.39
128.08
127.80
126.74
127.28
121.78
75.93
39.52 DMSO-d6
200
190
180
170
160
150
140
130
120
110
100
90
80
70
60
50
40
30
20
10
0
f1 (ppm)

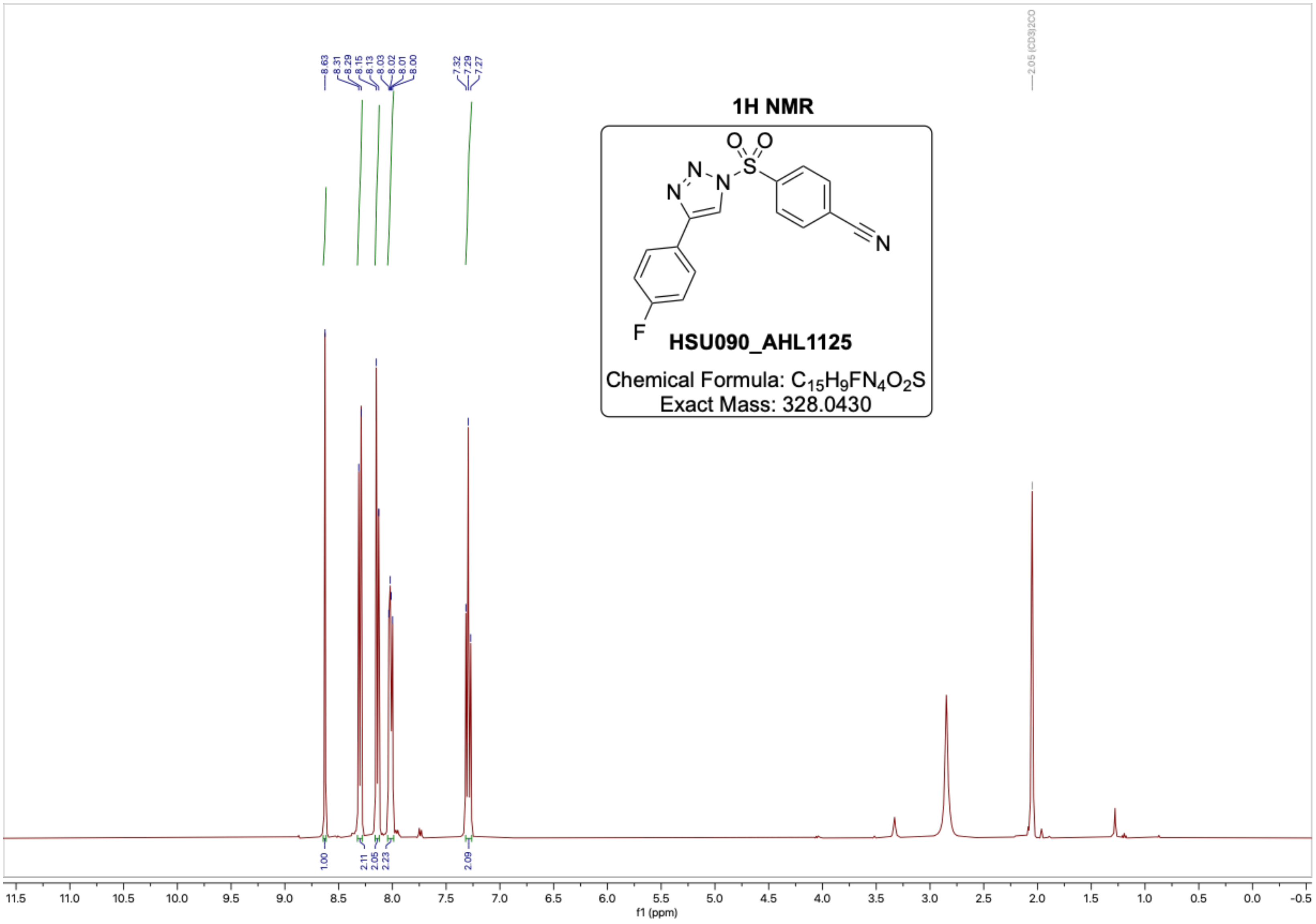
1H NMR
HSU090_AHL1125
Chemical Formula: C15H9FN4O2S
Exact Mass: 328.0430
8.63
8.31
8.29
8.15
8.13
8.03
8.02
8.01
8.00
7.32
7.29
7.27
2.05 (CD3)2CO
1.00
2.11
2.05
2.23
2.09
f1 (ppm)

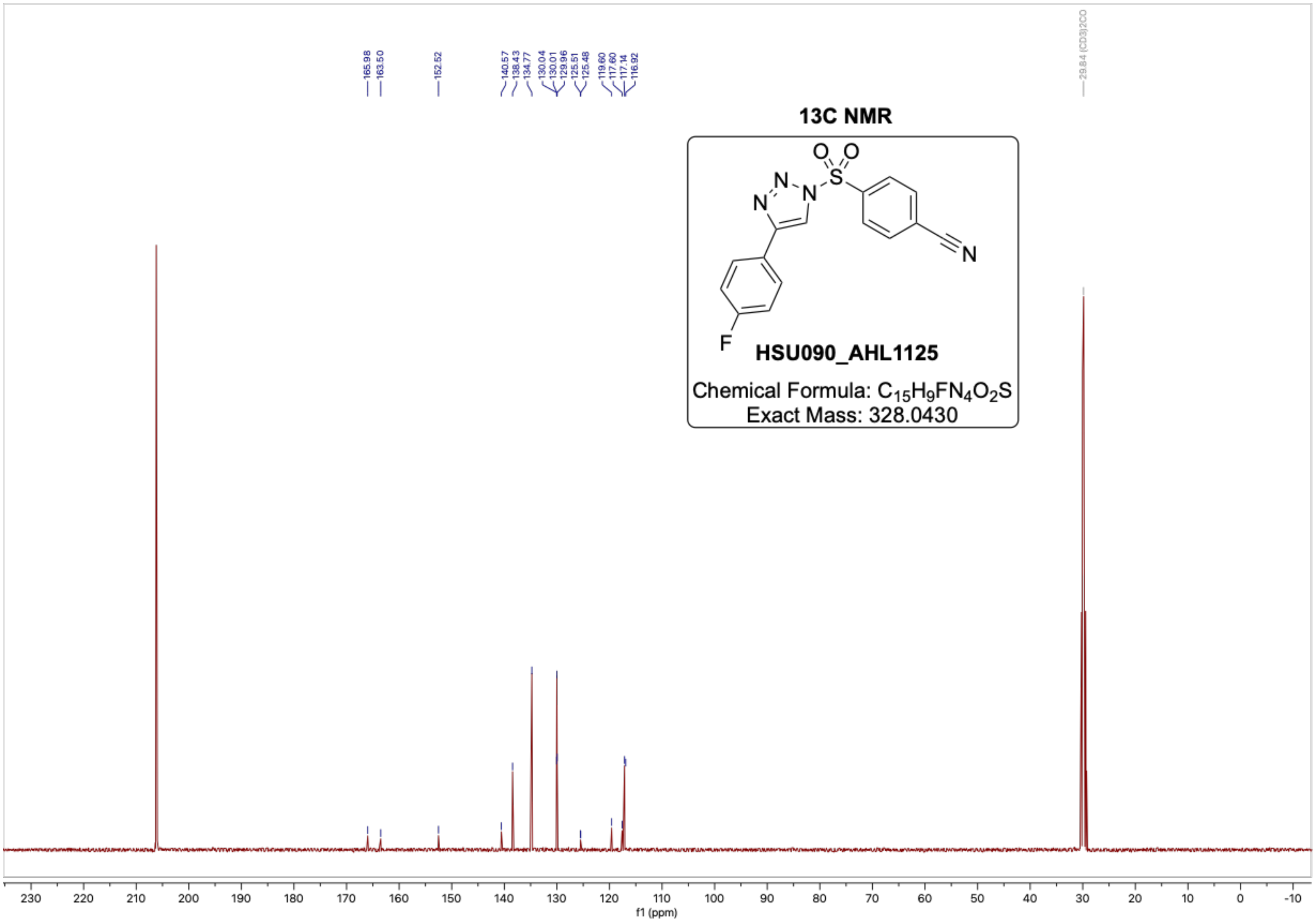

165.98
163.50
152.52
140.57
138.43
134.77
130.04
130.01
129.96
125.51
125.48
119.60
117.60
117.14
116.92
29.84 (CD3)2CO
13C NMR
O
O
N
N
N
N
S
N
F
HSU090_AHL1125
Chemical Formula: C15H9FN4O2S
Exact Mass: 328.0430
230
220
210
200
190
180
170
160
150
140
130
120
110
100
90
80
70
60
50
40
30
20
10
0
-10
f1 (ppm)

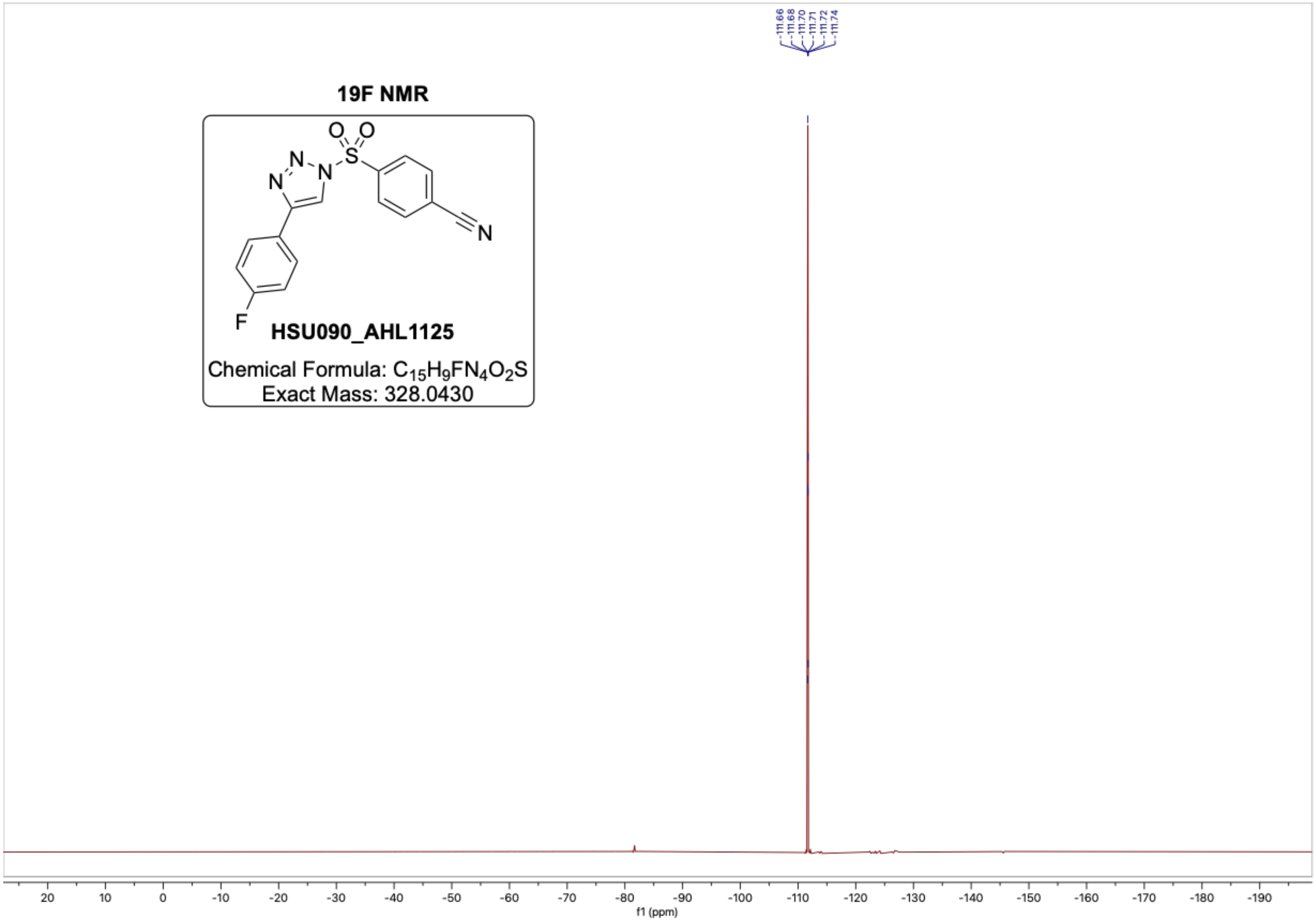

-111.66
-111.68
-111.70
-111.71
-111.72
-111.74
19F NMR
HSU090_AHL1125
Chemical Formula: $C_{15}H_9FN_4O_2S$
Exact Mass: 328.0430
20 10 0 -10 -20 -30 -40 -50 -60 -70 -80 -90 -100 -110 -120 -130 -140 -150 -160 -170 -180 -190
f1 (ppm)

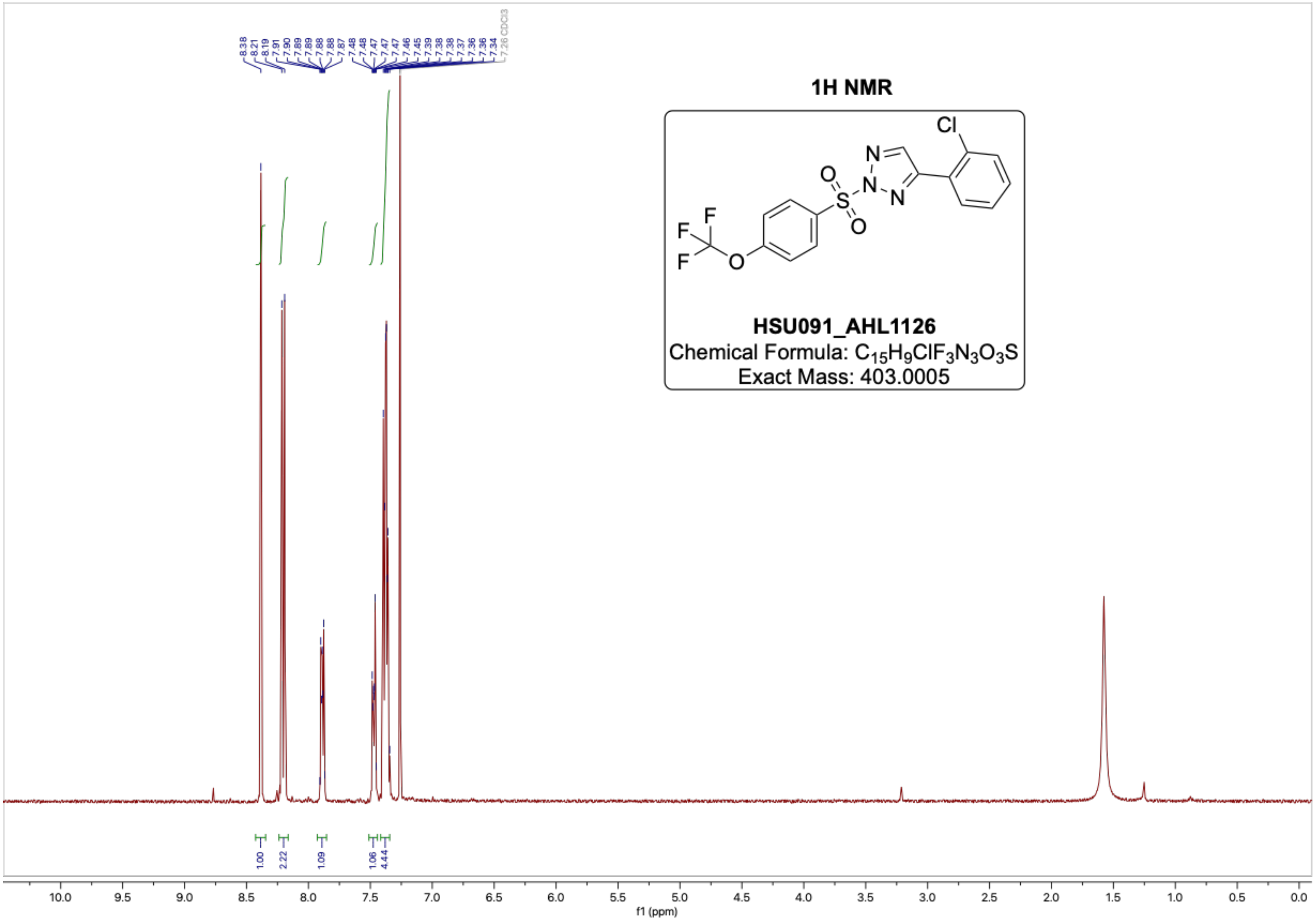

1H NMR
Cl
N
O
S
N
N
F
F
F
O
O
HSU091_AHL1126
Chemical Formula: C15H9ClF3N3O3S
Exact Mass: 403.0005
8.38
8.21
8.19
7.91
7.90
7.89
7.89
7.88
7.88
7.87
7.48
7.48
7.47
7.47
7.47
7.46
7.45
7.39
7.38
7.38
7.37
7.36
7.36
7.34
7.26 CDCl3
1.00
2.22
1.09
1.06
4.44
10.0
9.5
9.0
8.5
8.0
7.5
7.0
6.5
6.0
5.5
5.0
4.5
4.0
3.5
3.0
2.5
2.0
1.5
1.0
0.5
0.0
f1 (ppm)

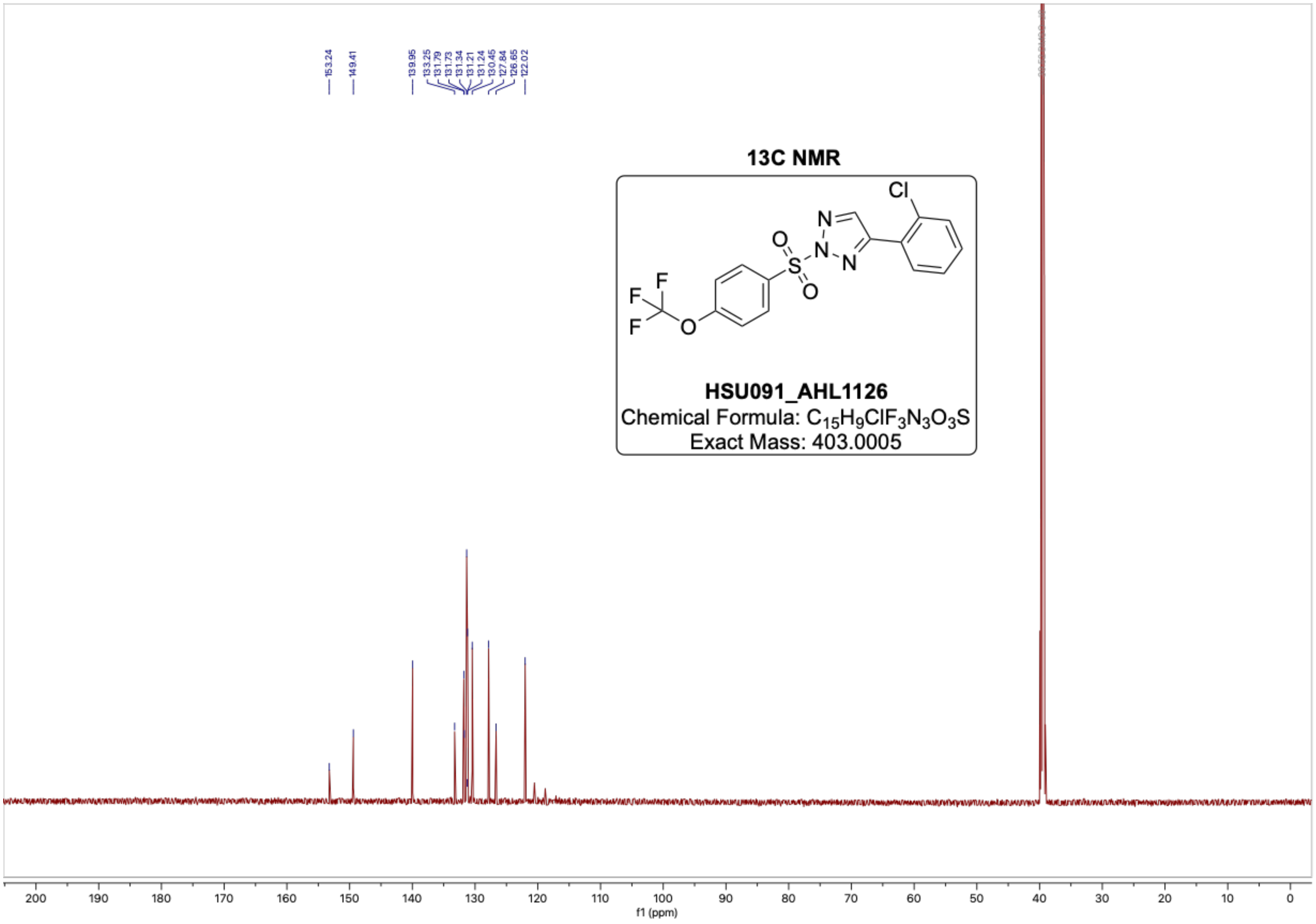

153.24
149.41
139.95
133.25
131.79
131.73
131.34
131.21
131.24
130.45
127.84
126.65
122.02
13C NMR
HSU091_AHL1126
Chemical Formula: C15H9ClF3N3O3S
Exact Mass: 403.0005
f1 (ppm)

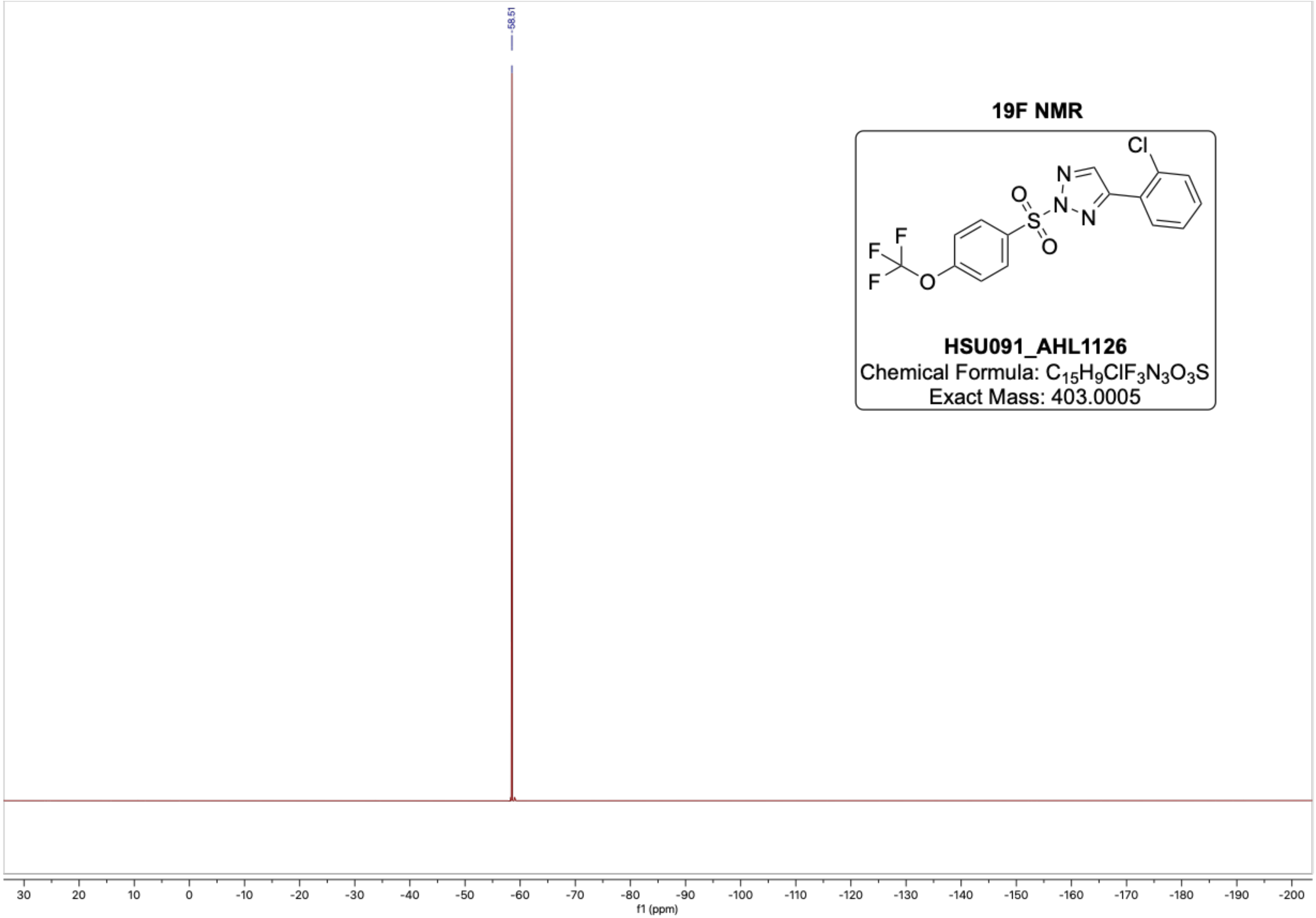

-58.51
19F NMR
Cl
N
N
N
O
S
O
F
F
F
O
HSU091_AHL1126
Chemical Formula: C15H9ClF3N3O3S
Exact Mass: 403.0005
30
20
10
0
-10
-20
-30
-40
-50
-60
-70
-80
-90
-100
-110
-120
-130
-140
-150
-160
-170
-180
-190
-200
f1 (ppm)

1H NMR

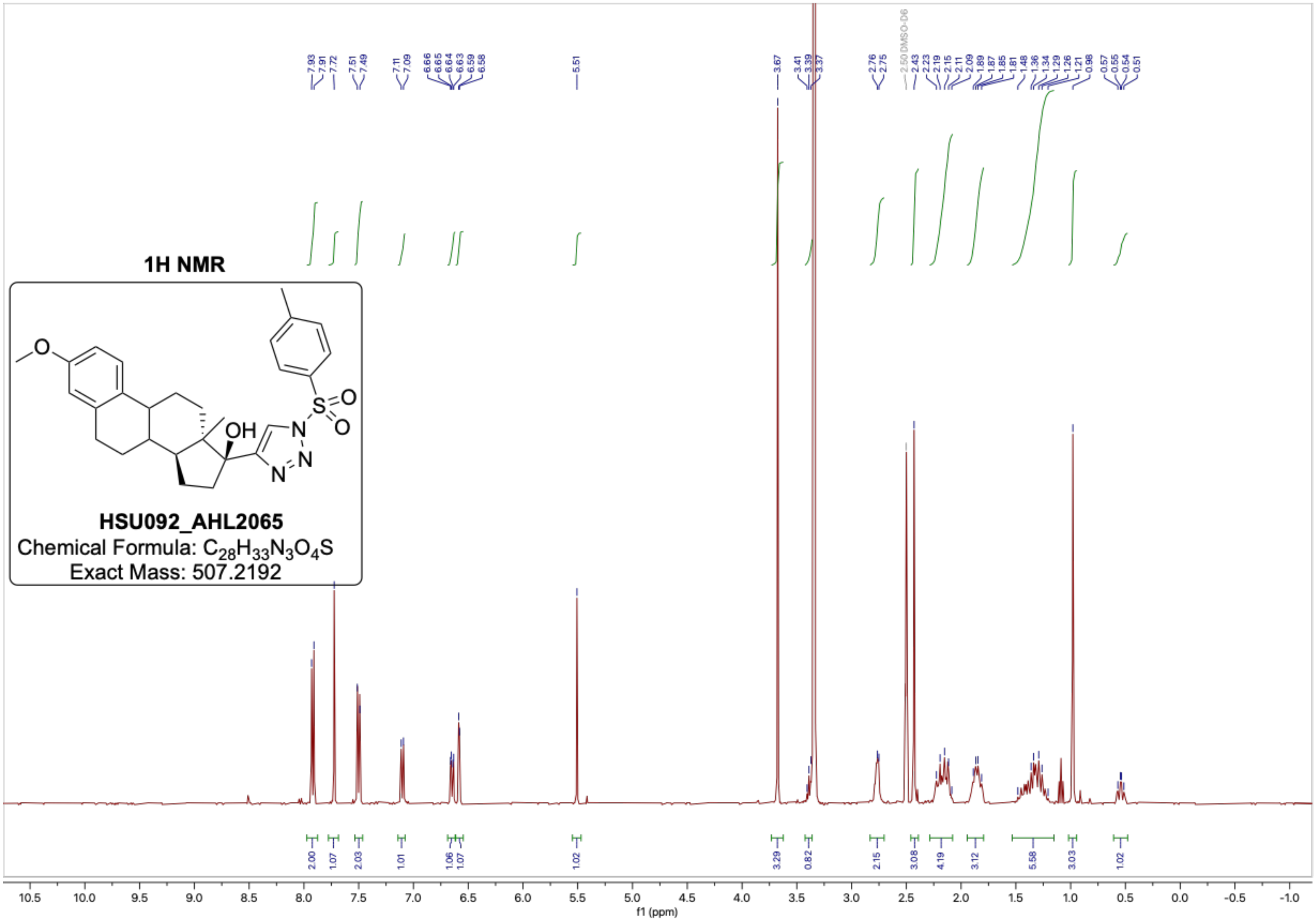

HSU092_AHL2065
Chemical Formula: $C_{28}H_{33}N_3O_4S$
Exact Mass: 507.2192

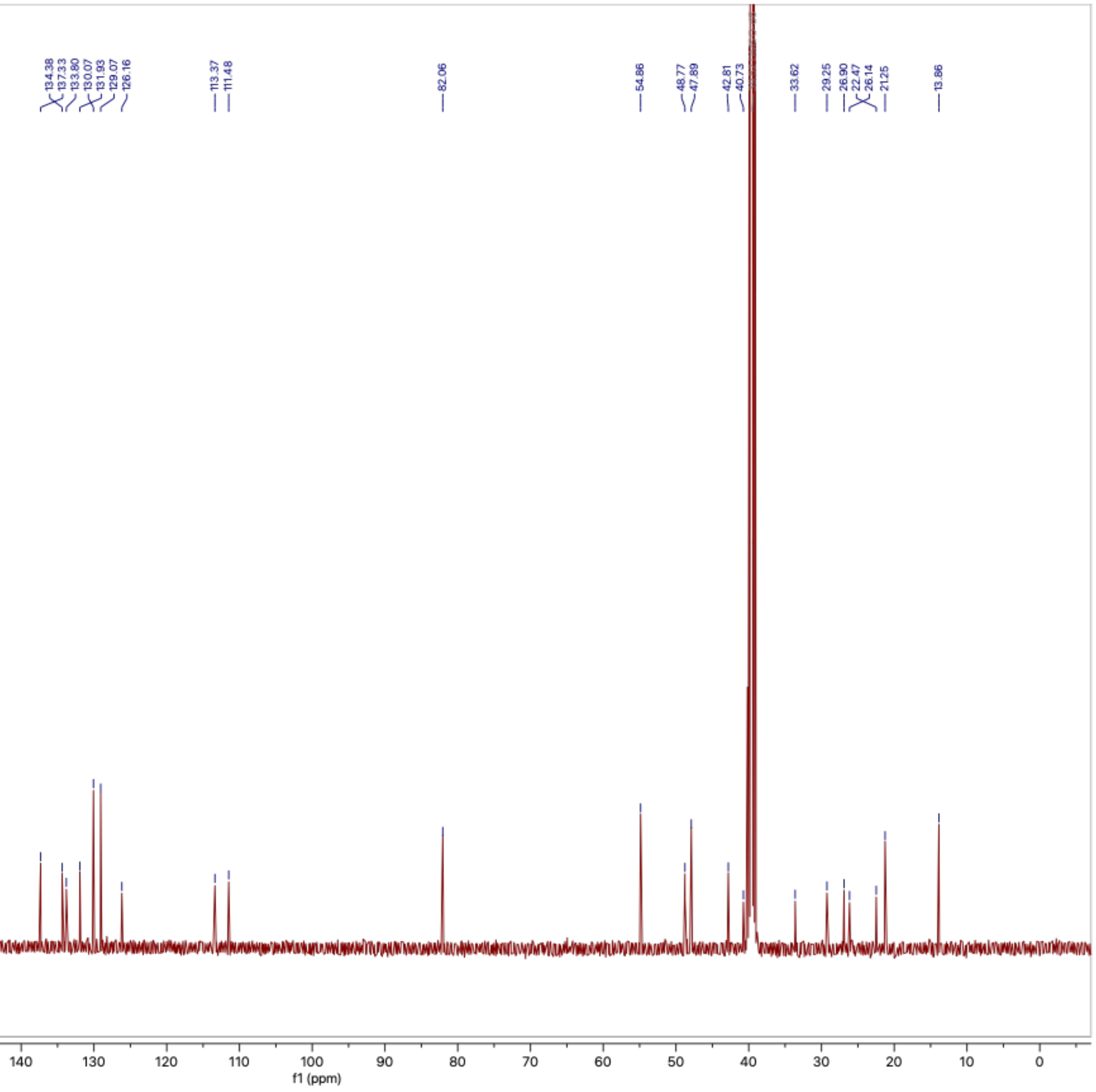

13C NMR

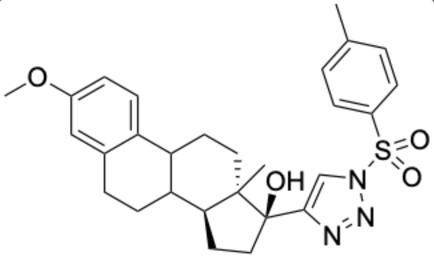

HSU092_AHL2065

Chemical Formula: $C_{28}H_{33}N_3O_4S$

Exact Mass: 507.2192

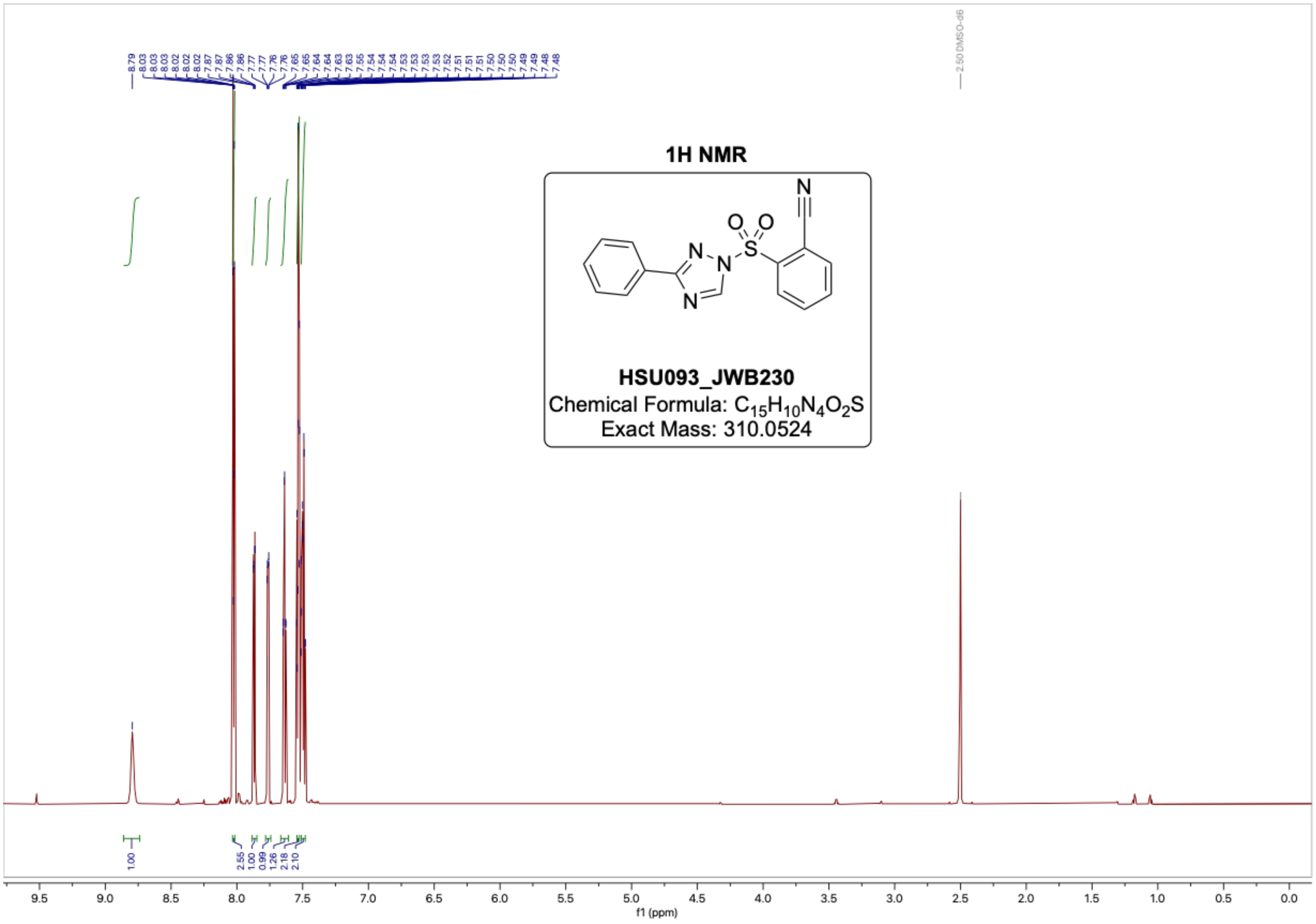
1H NMR
HSU093_JWB230
Chemical Formula: $C_{15}H_{10}N_4O_2S$
Exact Mass: 310.0524
2.50 DMSO-d6
f1 (ppm)

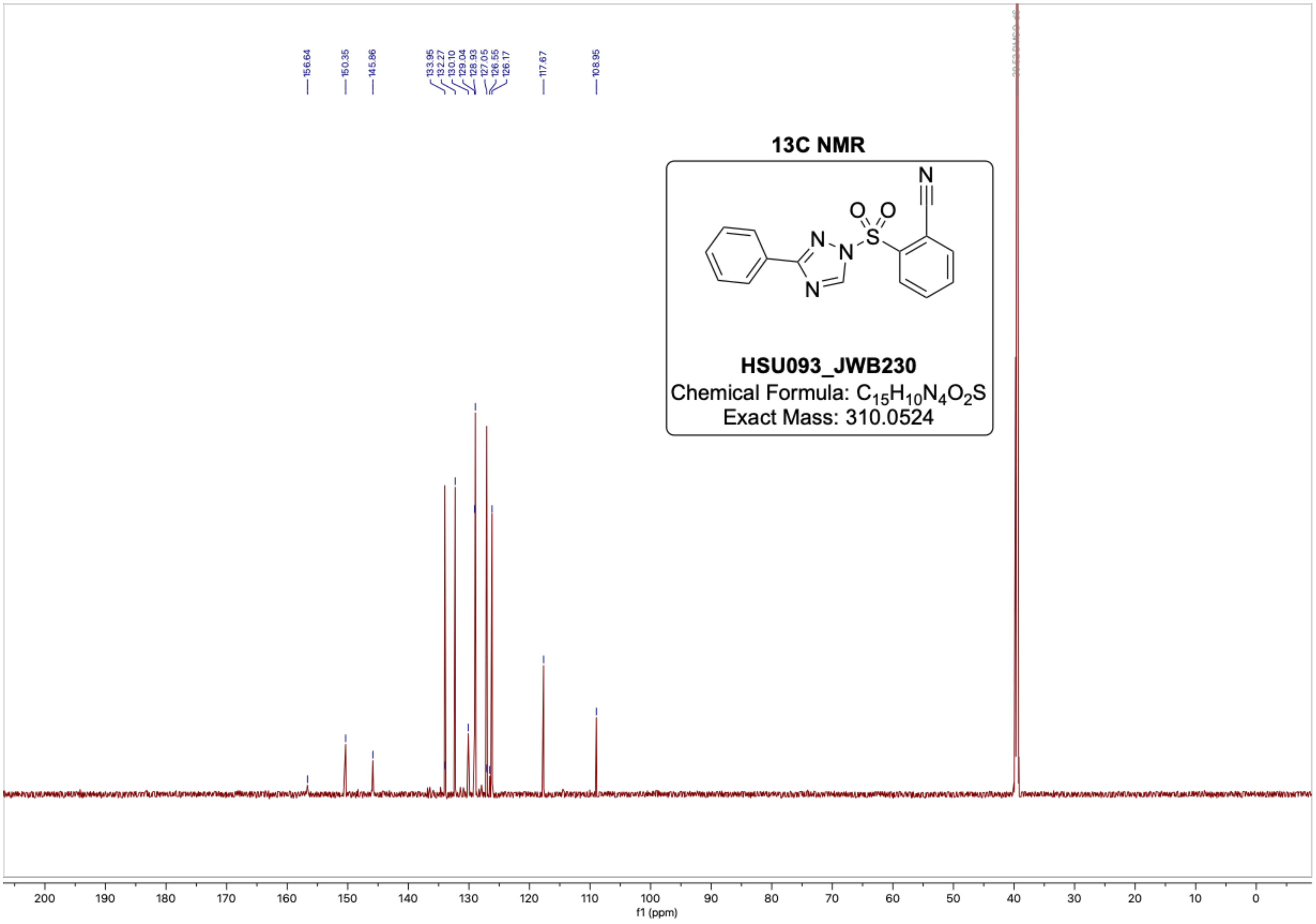

156.64
150.35
145.86
133.95
132.27
130.10
129.04
128.93
127.05
126.55
126.17
117.67
108.95
13C NMR
HSU093_JWB230
Chemical Formula: C15H10N4O2S
Exact Mass: 310.0524
200
190
180
170
160
150
140
130
120
110
100
90
80
70
60
50
40
30
20
10
0
f1 (ppm)

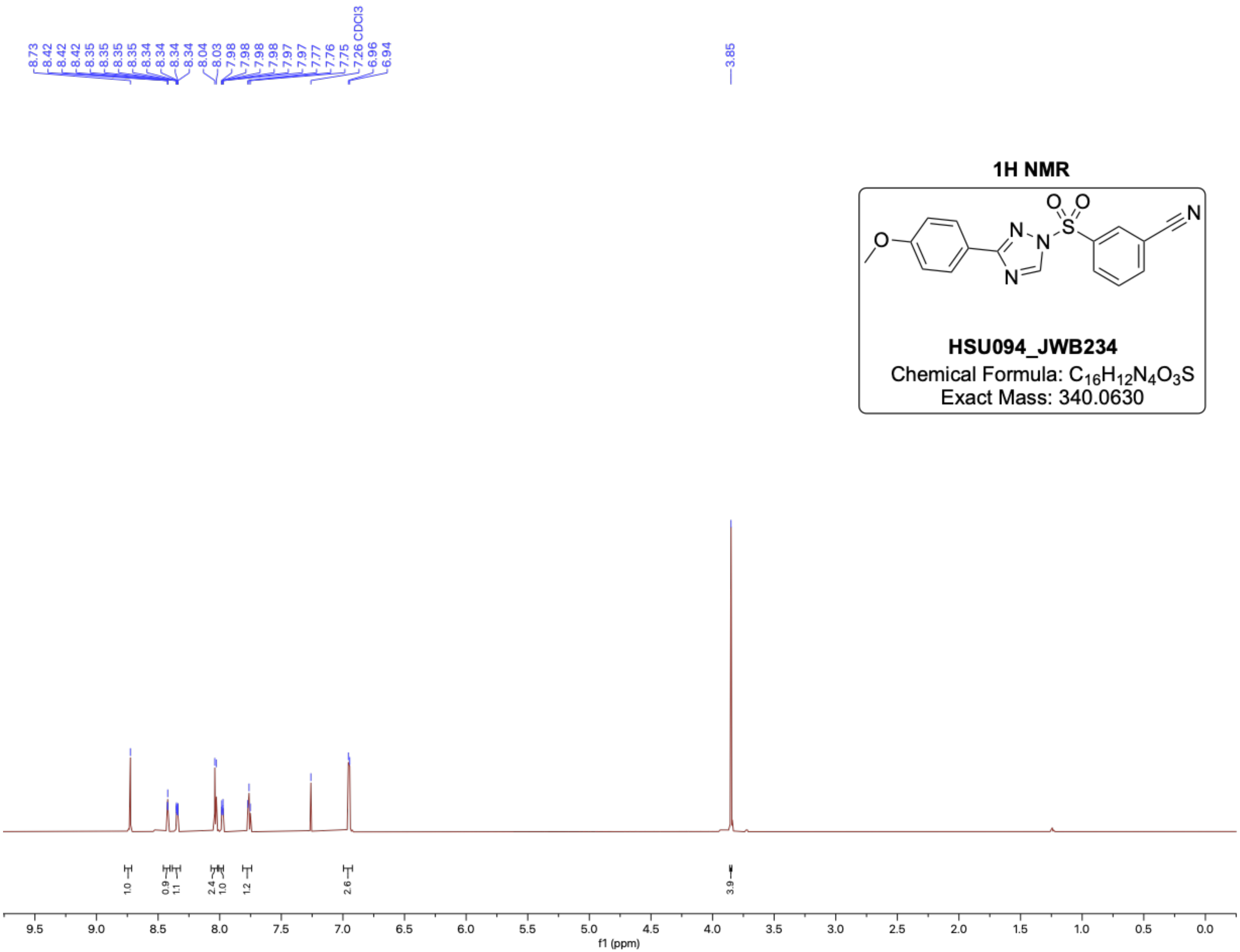
8.73
8.42
8.42
8.42
8.35
8.35
8.35
8.35
8.34
8.34
8.34
8.04
8.03
7.98
7.98
7.98
7.98
7.97
7.97
7.77
7.76
7.75
7.26 CDCl3
6.96
6.94
3.85
1H NMR
HSU094_JWB234
Chemical Formula: C16H12N4O3S
Exact Mass: 340.0630
1.0
0.9
1.1
2.4
1.0
1.2
2.6
3.9
9.5
9.0
8.5
8.0
7.5
7.0
6.5
6.0
5.5
5.0
4.5
4.0
3.5
3.0
2.5
2.0
1.5
1.0
0.5
0.0
f1 (ppm)

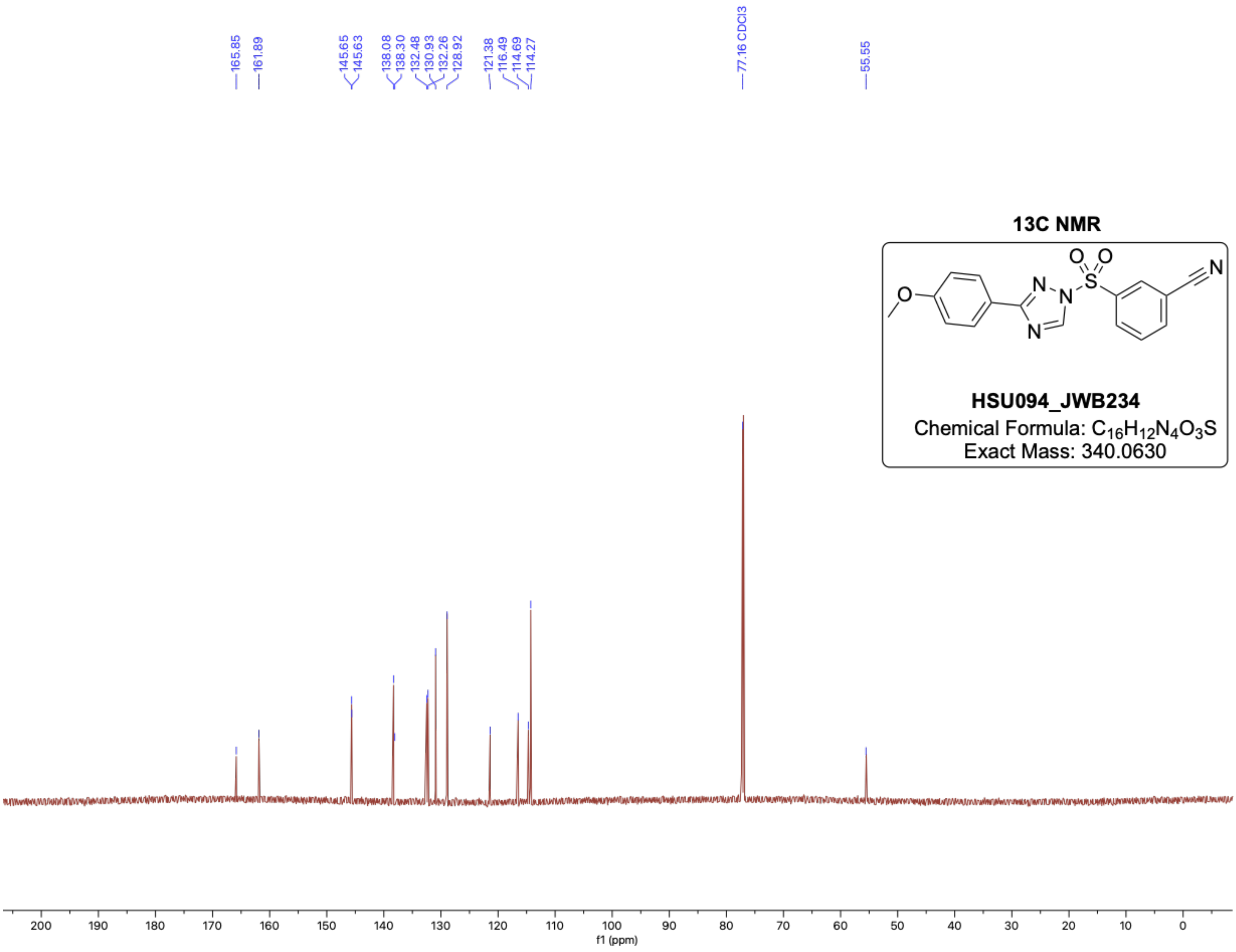
165.85
161.89
145.65
145.63
138.08
138.30
132.48
130.93
132.26
128.92
121.38
116.49
114.69
114.27
77.16 CDCl3
55.55
13C NMR
HSU094_JWB234
Chemical Formula: $C_{16}H_{12}N_4O_3S$
Exact Mass: 340.0630
200
190
180
170
160
150
140
130
120
110
100
90
80
70
60
50
40
30
20
10
0
f1 (ppm)

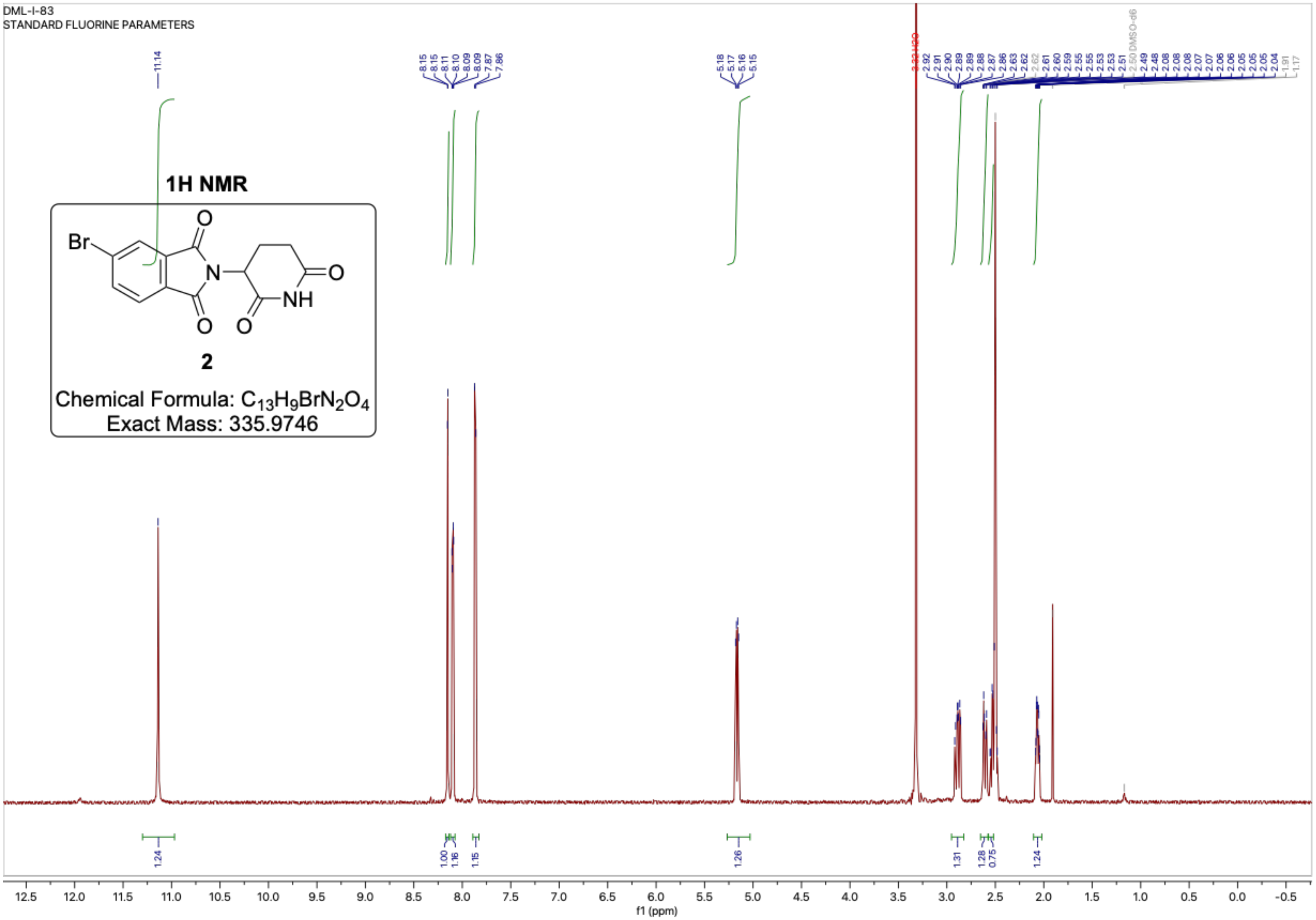
DML-I-83
STANDARD FLUORINE PARAMETERS
1H NMR
Br
N
NH
O
2
Chemical Formula: C13H9BrN2O4
Exact Mass: 335.9746
11.14
1.24
1.00
1.16
1.15
1.26
1.31
1.28
0.75
1.24
f1 (ppm)

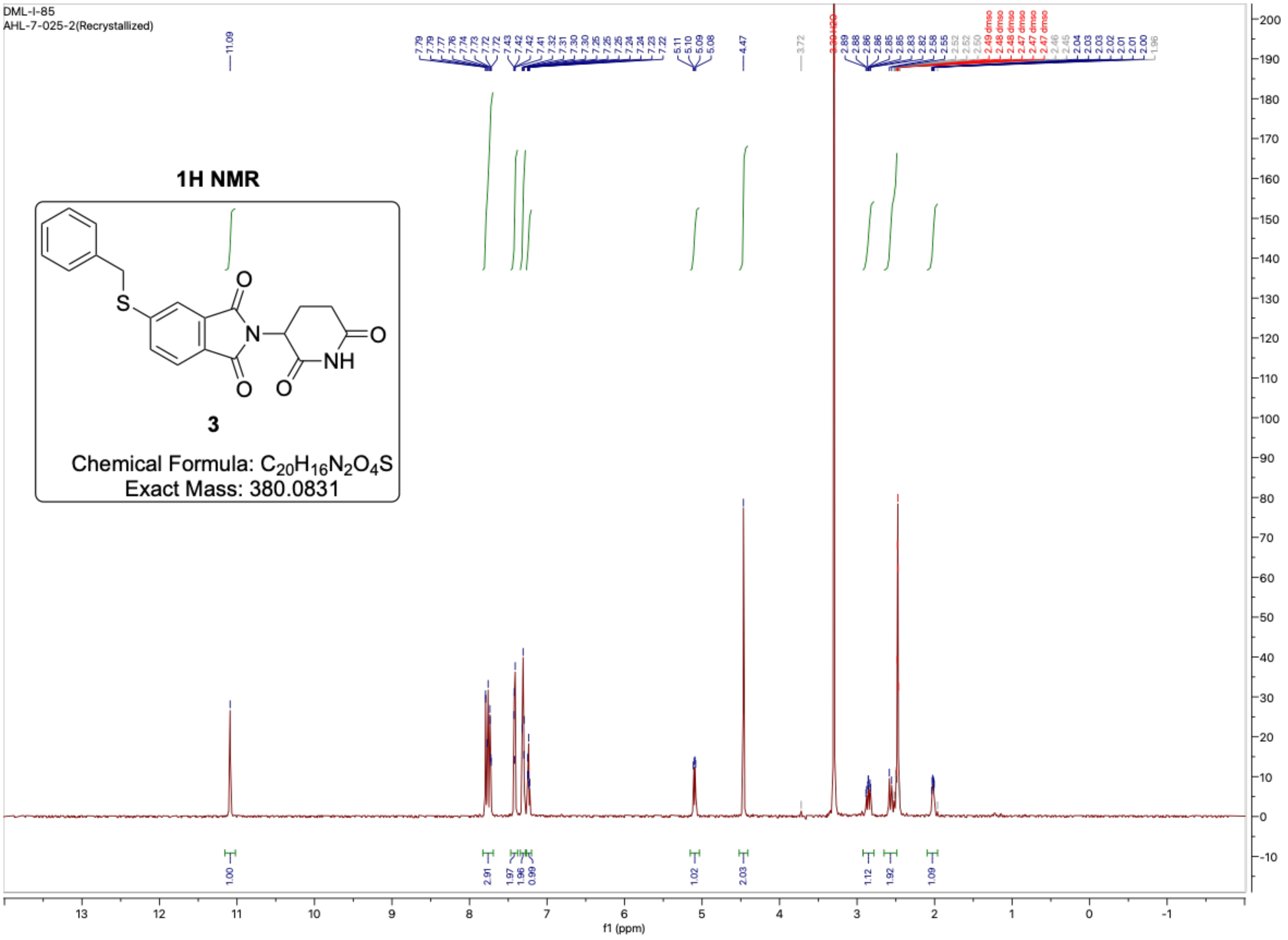

DML-I-85
AHL-7-025-2(Recrystallized)
1H NMR
3
Chemical Formula: C20H16N2O4S
Exact Mass: 380.0831
f1 (ppm)

DML-I-87
STANDARD FLUORINE PARAMETERS

1H NMR

4

Chemical Formula: $C_{13}H_9ClN_2O_6S$
Exact Mass: 355.9870

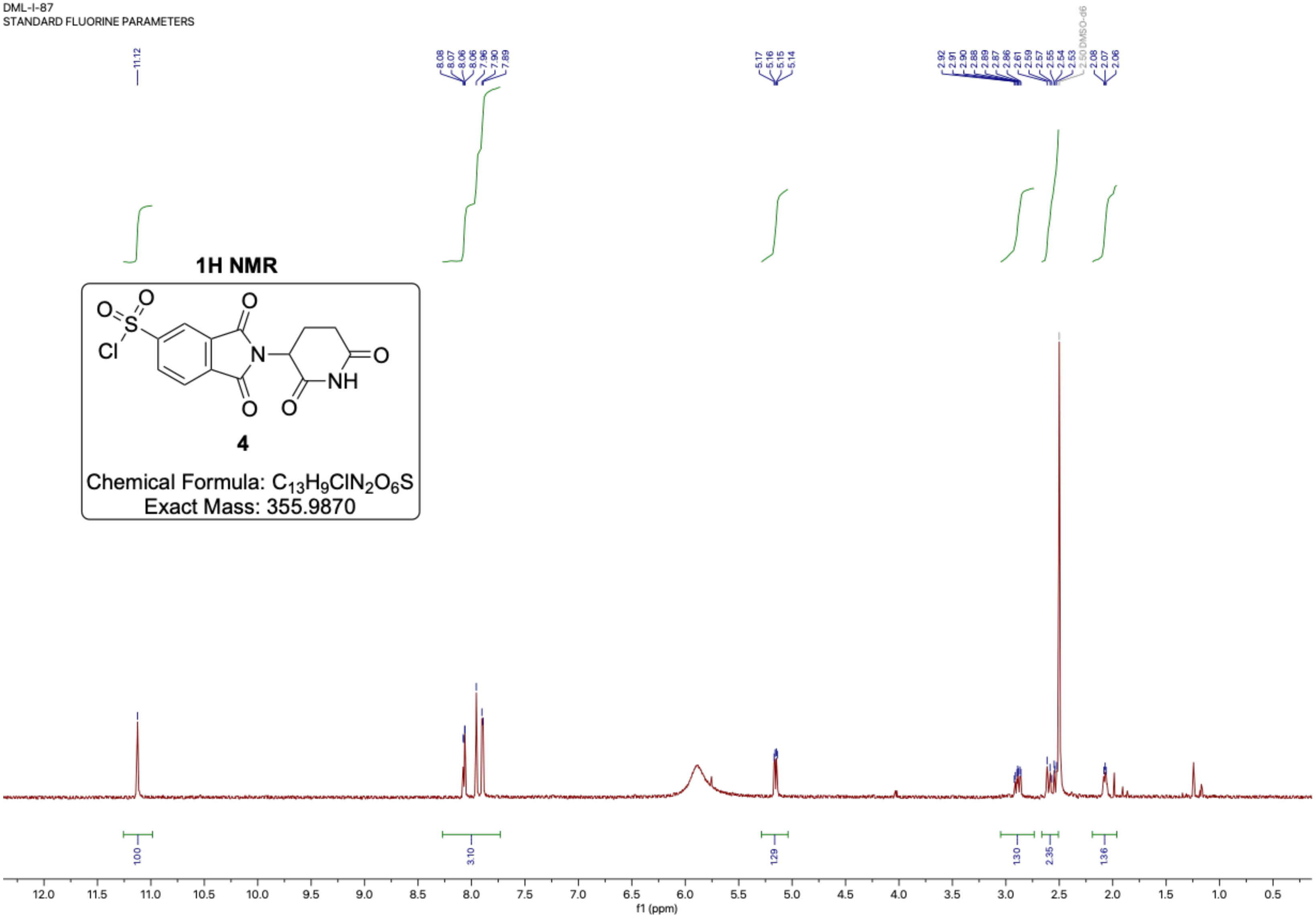

Target Compound Screening Report

Results Acquired by The University of Texas at Austin Mass Spectrometry Facility

**Data File**	MSF25-2063(JWB179)_hrESIpos1.d	**Sample Name**	2063(JWB179)	**Comment**	2063(JWB179)
**Position**	P1-D1	**Instrument Name**	6530	**User Name**	
**Acq Method**	FIA_pos.m	**Acquired Time**	12/4/2025 10:43:53 AM	**DA Method**	MSF.m

MS Zoomed Spectrum

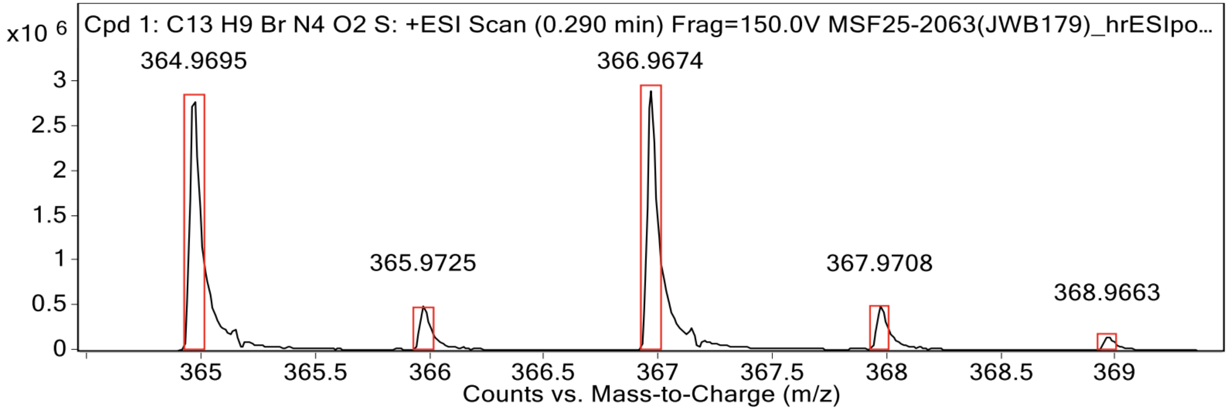

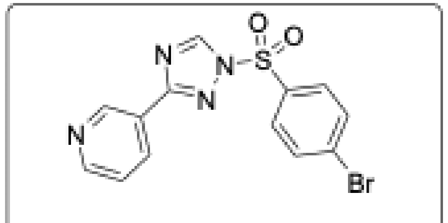

MS Spectrum Peak List

Obs. m/z	Calc. m/z	Charge	Abundance	Formula	Ion Species	Tgt Mass Error (ppm)
364.9695	364.9702	1	2836650	C13H9BrN4O2S	(M+H)+	2.1
365.9725	365.9729	1	501572	C13H9BrN4O2S	(M+H)+	1.08
366.9674	366.9682	1	2928898	C13H9BrN4O2S	(M+H)+	2.13
367.9708	367.9708	1	497794	C13H9BrN4O2S	(M+H)+	0.07
368.9663	368.9666	1	170136	C13H9BrN4O2S	(M+H)+	0.66

--- End Of Report ---

Target Compound Screening Report

Results Acquired by The University of Texas at Austin Mass Spectrometry Facility

**Data File**	MSF25-2042(JWB-222)_hrESIpos1.d	**Sample Name**	2042(JWB-222)	**Comment**	2042(JWB-222)
**Position**	P1-A9	**Instrument Name**	6530	**User Name**	
**Acq Method**	FIA_pos.m	**Acquired Time**	11/24/2025 2:05:59 PM	**DA Method**	MSF.m

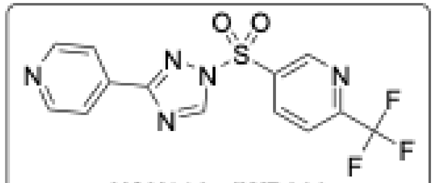

MS Zoomed Spectrum

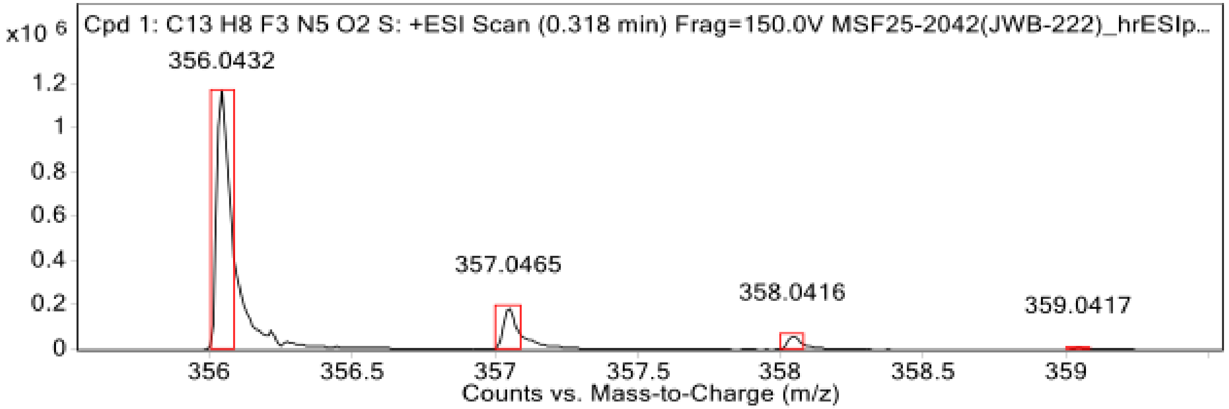

MS Spectrum Peak List

Obs. m/z	Calc. m/z	Charge	Abundance	Formula	Ion Species	Tgt Mass Error (ppm)
356.0432	356.0424	1	1173525	C13H8F3N5O2S	(M+H)+	-2.26
357.0465	357.0449	1	192606	C13H8F3N5O2S	(M+H)+	-4.47
358.0416	358.0407	1	67591	C13H8F3N5O2S	(M+H)+	-2.58
359.0417	359.0422	1	9398	C13H8F3N5O2S	(M+H)+	1.33

--- End Of Report ---

**Sample Name**	CPT03	**Position**	P1-A1	**Instrument Name**	Instrument 1
**User Name**		**Inj Vol**	0.1	**InjPosition**	
**Sample Type**	Sample	**IRM Calibration Status**	Success	**Data Filename**	CPT03.d
**ACQ Method**	Acq 100 ACN.m	**Comment**		**Acquired Time**	2/17/2022 10:19:34 AM (UTC-05:00)

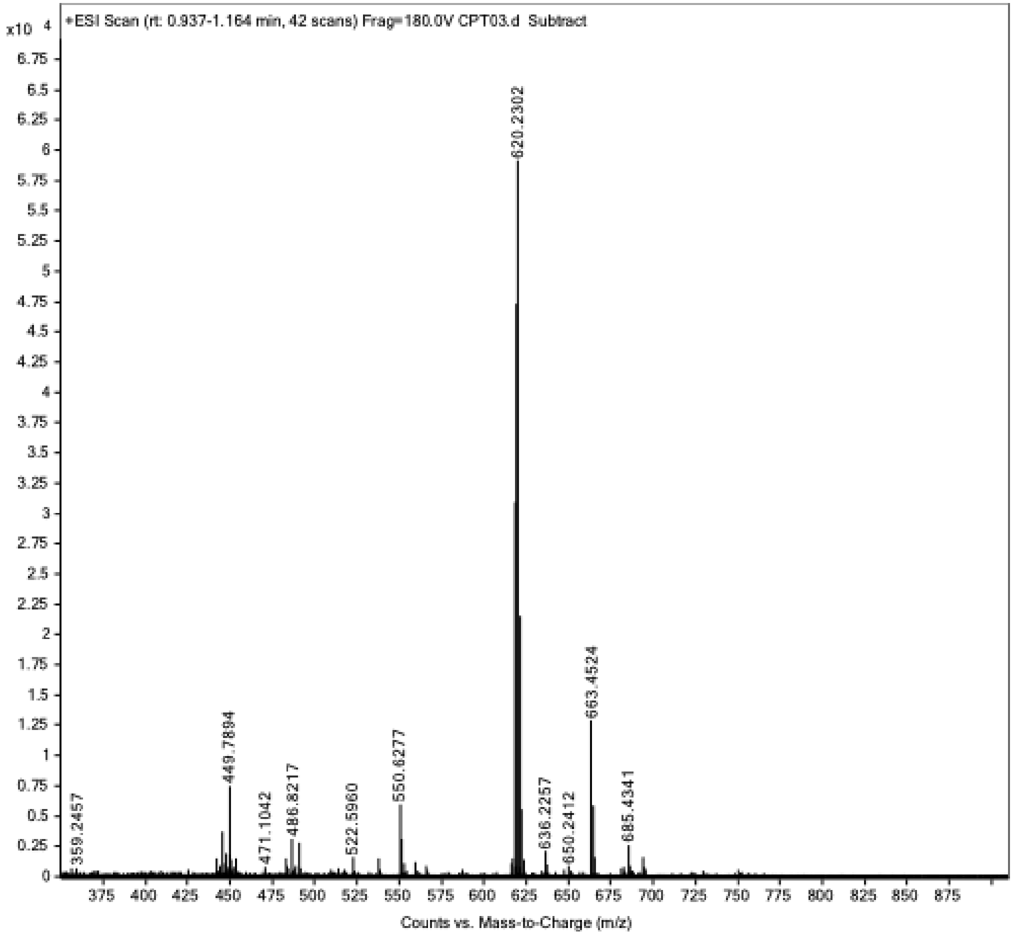

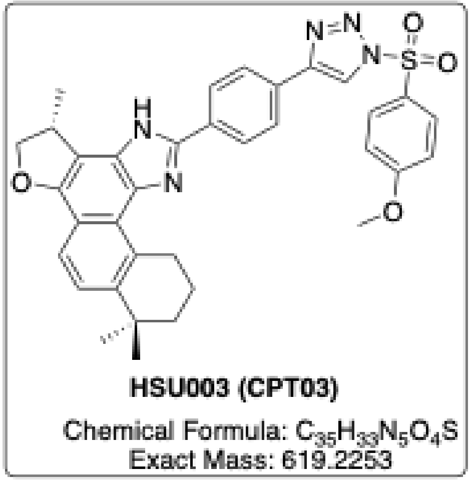

Target Compound Screening Report

Results Acquired by The University of Texas at Austin Mass Spectrometry Facility

**Data File**	MSF25-2044(JWB-226)_hrESIpos1.d	**Sample Name**	2044(JWB-226)	**Comment**	2044(JWB-226)
**Position**	P1-B2	**Instrument Name**	6530	**User Name**	
**Acq Method**	FIA_pos.m	**Acquired Time**	11/24/2025 2:10:01 PM	**DA Method**	MSF.m

MS Zoomed Spectrum

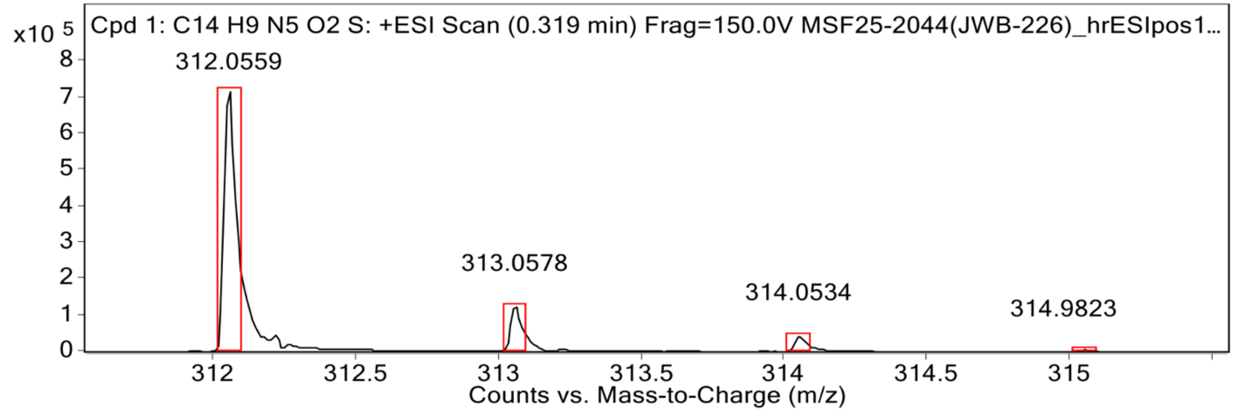

MS Spectrum Peak List

Obs. m/z	Calc. m/z	Charge	Abundance	Formula	Ion Species	Tgt Mass Error (ppm)
312.0559	312.0550	1	724030	C14H9N5O2S	(M+H)+	-2.87
313.0578	313.0575	1	126193	C14H9N5O2S	(M+H)+	-0.72
314.0534	314.0535	1	45243	C14H9N5O2S	(M+H)+	0.4
315.9266	316.0561	1	599	C14H9N5O2S	(M+H)+	409.74
316.8873	317.0575	1	484	C14H9N5O2S	(M+H)+	537.24

--- End Of Report ---

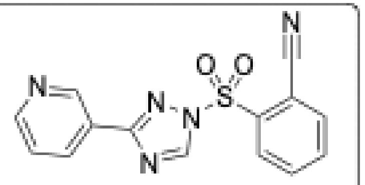

HSU006_JWB226

Chemical Formula: $C_{14}H_{10}N_5O_2S$

Exact Mass: 312.0555

Target Compound Screening Report

Results Acquired by The University of Texas at Austin Mass Spectrometry Facility

**Data File**	MSF25-2063(JWB228)_hrESIpos1.d	**Sample Name**	2063(JWB228)	**Comment**	2063(JWB228)
**Position**	P1-D3	**Instrument Name**	6530	**User Name**	
**Acq Method**	FIA_pos.m	**Acquired Time**	12/4/2025 10:47:54 AM	**DA Method**	MSF.m

MS Zoomed Spectrum

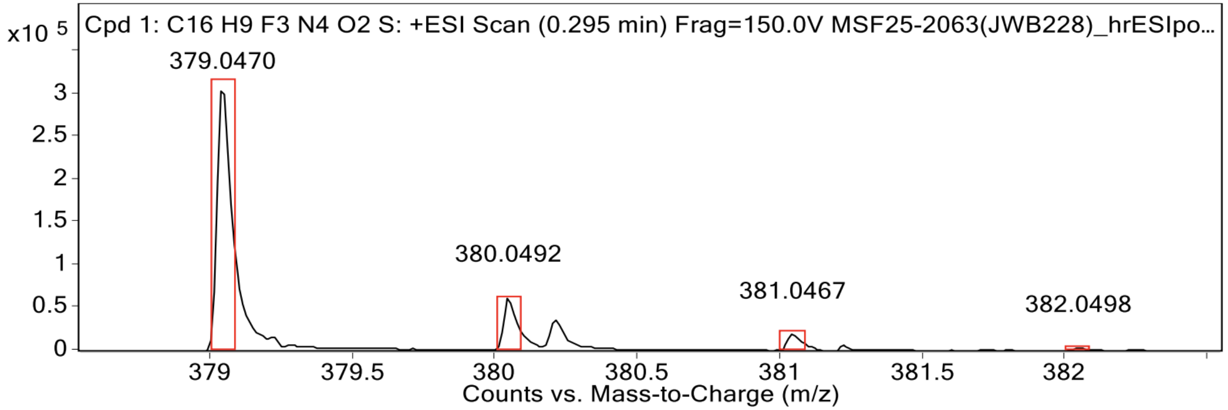

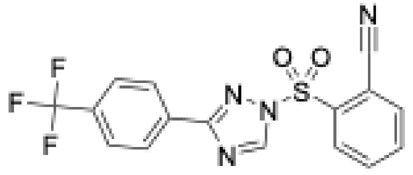

HSU010_JWB228

Chemical Formula: $C_{16}H_9F_3N_4O_2S$

Exact Mass: 378.0398

MS Spectrum Peak List

Obs. m/z	Calc. m/z	Charge	Abundance	Formula	Ion Species	Tgt Mass Error (ppm)
302.1529			1704604			
379.0470	379.0471	1	315264	C16H9F3N4O2S	(M+H)+	0.19
380.0492	380.0499	1	62977	C16H9F3N4O2S	(M+H)+	1.74
381.0467	381.0461	1	19793	C16H9F3N4O2S	(M+H)+	-1.64
382.0498	382.0474	1	4104	C16H9F3N4O2S	(M+H)+	-6.17

--- End Of Report ---

Target Compound Screening Report

Results Acquired by The University of Texas at Austin Mass Spectrometry Facility

**Data File**	MSF25-2037(AHL-041)_hrESIposLC1.d	**Sample Name**	2037(AHL-041)	**Comment**	2037(AHL-041)
**Position**	P1-A4	**Instrument Name**	6530	**User Name**	
**Acq Method**	LC_C18_pos_jl.m	**Acquired Time**	11/24/2025 3:33:43 PM	**DA Method**	MSF.m

MS Zoomed Spectrum

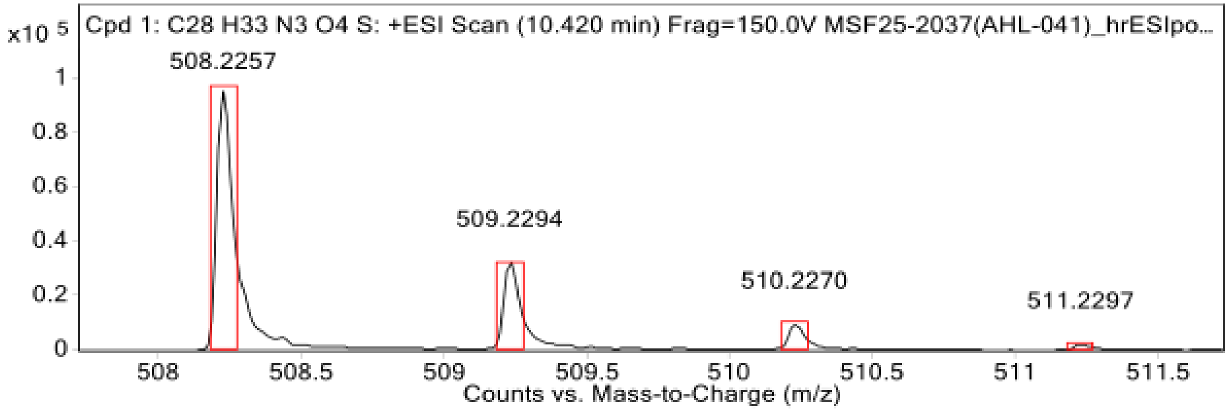

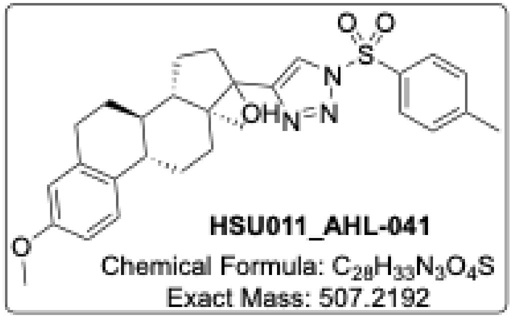

HSU011_AHL-041

Chemical Formula: $C_{28}H_{33}N_3O_4S$

Exact Mass: 507.2192

MS Spectrum Peak List

Obs. m/z	Calc. m/z	Charge	Abundance	Formula	Ion Species	Tgt Mass Error (ppm)
508.2257	508.2265	1	95809	C28H33N3O4S	(M+H)+	1.44
509.2294	509.2295	1	32568	C28H33N3O4S	(M+H)+	0.2
510.2270	510.2280	1	9952	C28H33N3O4S	(M+H)+	2.08
511.2297	511.2289	1	2526	C28H33N3O4S	(M+H)+	-1.59
512.2447	512.2303	1	442	C28H33N3O4S	(M+H)+	-28.12

--- End Of Report ---

Target Compound Screening Report

Results Acquired by The University of Texas at Austin Mass Spectrometry Facility

**Data File**	MSF25-2087(AHL011)_hrESIposLC1.d	**Sample Name**	2087(AHL011)	**Comment**	2087(AHL011)
**Position**	P1-C5	**Instrument Name**	6530	**User Name**	
**Acq Method**	LC_C18_pos_jl.m	**Acquired Time**	12/4/2025 5:48:51 PM	**DA Method**	MSF.m

MS Zoomed Spectrum

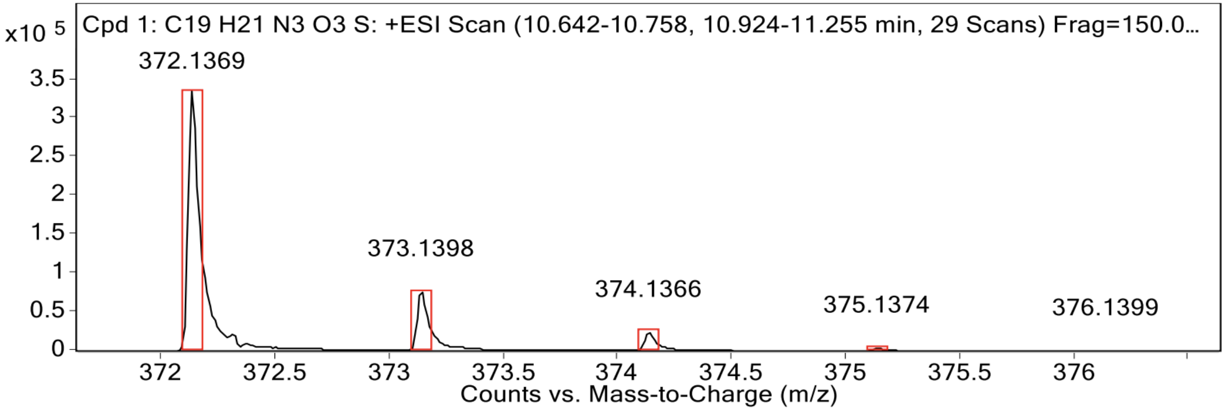

MS Spectrum Peak List

Obs. m/z	Calc. m/z	Charge	Abundance	Formula	Ion Species	Tgt Mass Error (ppm)
372.1369	372.1376	1	335642	C19H21N3O3S	(M+H)+	2.04
373.1398	373.1406	1	76837	C19H21N3O3S	(M+H)+	2.21
374.1366	374.1374	1	24236	C19H21N3O3S	(M+H)+	2.16
375.1374	375.1387	1	4089	C19H21N3O3S	(M+H)+	3.41
376.1399	376.1401	1	497	C19H21N3O3S	(M+H)+	0.66

--- End Of Report ---

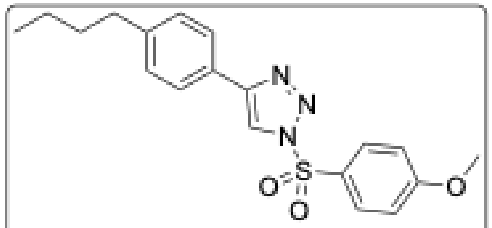

HSU012_AHL-011

Chemical Formula: $C_{19}H_{21}N_3O_3S$

Exact Mass: 371.1304

Target Compound Screening Report

Results Acquired by The University of Texas at Austin Mass Spectrometry Facility

**Data File**	MSF25-2063(AHL015)_hrESIpos1.d	**Sample Name**	2063(AHL015)	**Comment**	2063(AHL015)
**Position**	P1-E2	**Instrument Name**	6530	**User Name**	
**Acq Method**	FIA_pos.m	**Acquired Time**	12/4/2025 11:04:01 AM	**DA Method**	MSF.m

MS Zoomed Spectrum

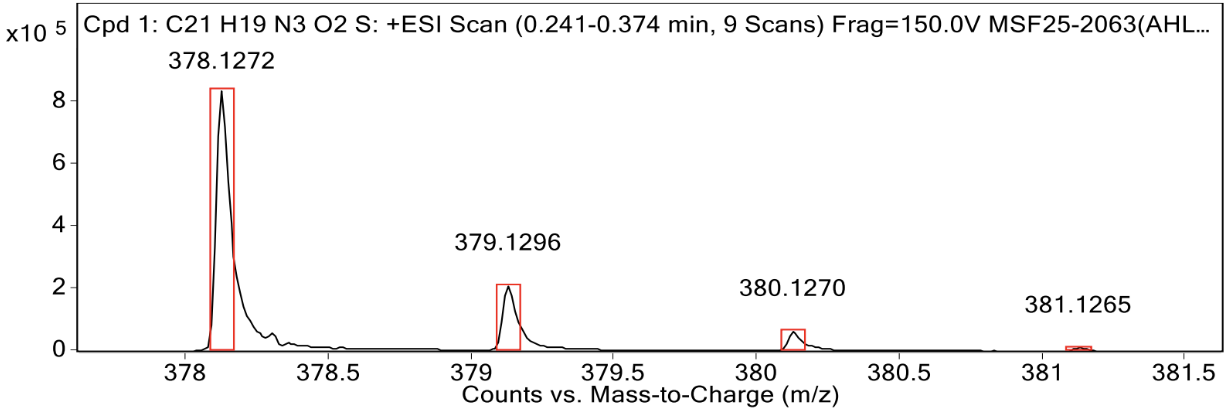

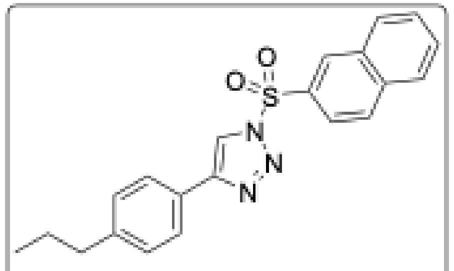

MS Spectrum Peak List

Obs. m/z	Calc. m/z	Charge	Abundance	Formula	Ion Species	Tgt Mass Error (ppm)
372.1022			3559317			
378.1272	378.1271	1	834563	C21H19N3O2S	(M+H)+	-0.35
379.1296	379.1301	1	210880	C21H19N3O2S	(M+H)+	1.26
380.1270	380.1272	1	64437	C21H19N3O2S	(M+H)+	0.35
381.1265	381.1282	1	10923	C21H19N3O2S	(M+H)+	4.28

--- End Of Report ---

Target Screening Report

Results Acquired by The University of Texas at Austin Mass Spectrometry Facility

**Data File**	MSF25-2099(EKT116)_hrESIpos1.d	**Sample Name**	2099(EKT116)	**Comment**	2099(EKT116)
**Position**	P1-F3	**Instrument Name**	Instrument 1		
**Acquisition Method**	FIA_Pos.m	**Acquired Time**	12/15/2025 5:16:48 PM (UTC-06:00)	**DA Method**	MSF.m

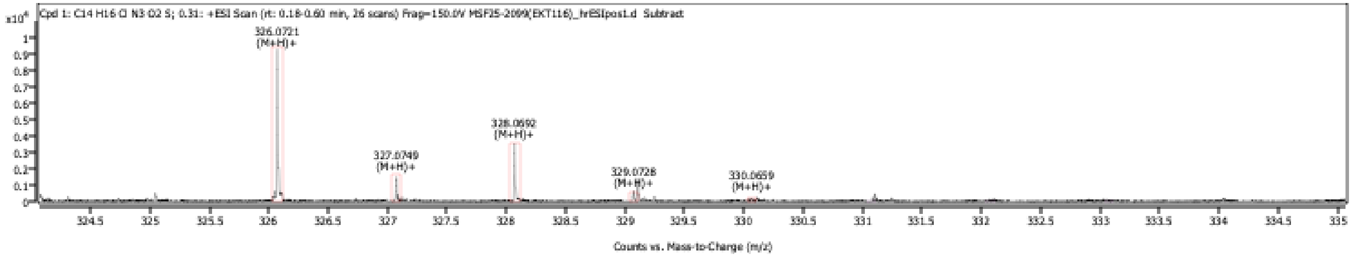

Spectrum Peaks

Obs. m/z	Calc. m/z	Charge	Abund	Formula	Ion Species	Tgt Mass Error (PPM)
326.0721	326.0725	1	9354	C14H16ClN3O2S	(M+H)+	-1.17
327.0749	327.0753	1	1485	C14H16ClN3O2S	(M+H)+	-1.20
328.0692	328.0697	1	3574	C14H16ClN3O2S	(M+H)+	-1.57
329.0728	329.0724	1	632	C14H16ClN3O2S	(M+H)+	1.39
330.0659	330.0683	1	197	C14H16ClN3O2S	(M+H)+	-7.27
130.1590			8001873			

MassHunter Qual 10.0
(End of Report)

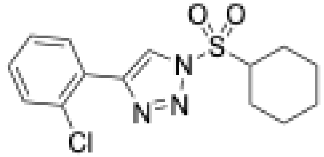

HSU014_EKT116

Chemical Formula: $C_{14}H_{16}ClN_3O_2S$
Exact Mass: 325.0652

Target Compound Screening Report

Results Acquired by The University of Texas at Austin Mass Spectrometry Facility

**Data File**	MSF25-2063(EKT151)_hrESIposLC1.d	**Sample Name**	2063(EKT151)	**Comment**	2063(EKT151)
**Position**	P1-D8	**Instrument Name**	6530	**User Name**	
**Acq Method**	LC_C18_pos_jl.m	**Acquired Time**	12/4/2025 10:01:33 PM	**DA Method**	MSF.m

MS Zoomed Spectrum

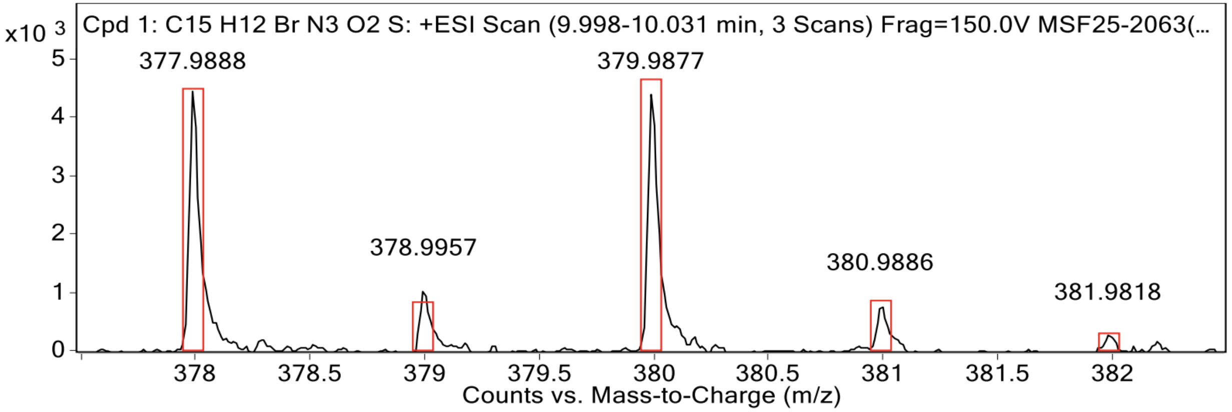

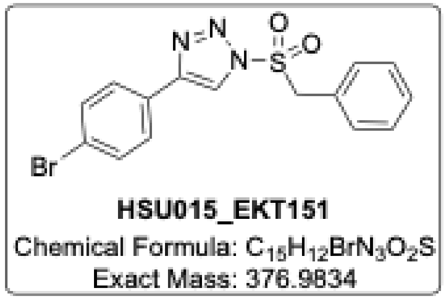

MS Spectrum Peak List

Obs. m/z	Calc. m/z	Charge	Abundance	Formula	Ion Species	Tgt Mass Error (ppm)
377.9888	377.9906	1	4475	C15H12BrN3O2S	(M+H)+	4.78
378.9957	378.9935	1	1048	C15H12BrN3O2S	(M+H)+	-5.96
379.9877	379.9886	1	4426	C15H12BrN3O2S	(M+H)+	2.36
380.9886	380.9914	1	794	C15H12BrN3O2S	(M+H)+	7.42
381.9818	381.9874	1	287	C15H12BrN3O2S	(M+H)+	14.61
922.0021			59582			

--- End Of Report ---

Target Compound Screening Report

Results Acquired by The University of Texas at Austin Mass Spectrometry Facility

**Data File**	MSF25-2039(EKT-165)_hrESIpos1.d	**Sample Name**	2039(EKT-165)	**Comment**	2039(EKT-165)
**Position**	P1-A6	**Instrument Name**	6530	**User Name**	
**Acq Method**	FIA_pos.m	**Acquired Time**	11/24/2025 1:59:51 PM	**DA Method**	MSF.m

MS Zoomed Spectrum

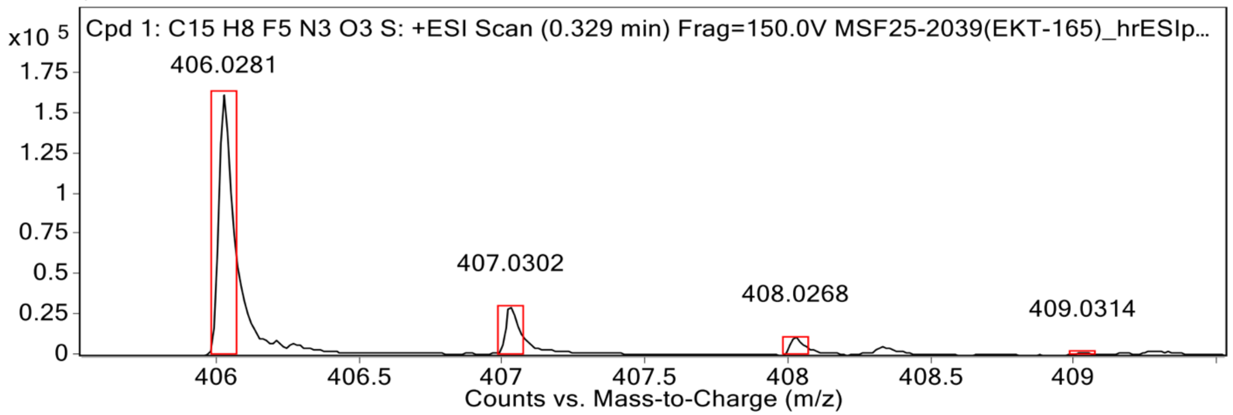

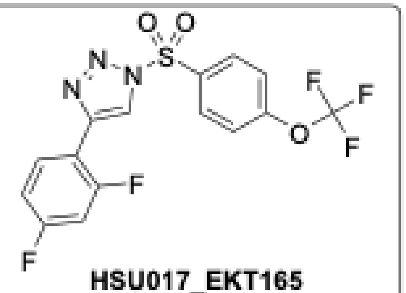

MS Spectrum Peak List

Obs. m/z	Calc. m/z	Charge	Abundance	Formula	Ion Species	Tgt Mass Error (ppm)
406.0281	406.0279	1	161689	C15H8F5N3O3S	(M+H)+	-0.33
407.0302	407.0308	1	30496	C15H8F5N3O3S	(M+H)+	1.44
408.0268	408.0268	1	11438	C15H8F5N3O3S	(M+H)+	0.17
409.0314	409.0285	1	2202	C15H8F5N3O3S	(M+H)+	-7.02
409.9521	410.0297	1	261	C15H8F5N3O3S	(M+H)+	189.15

--- End Of Report ---

Target Compound Screening Report

Results Acquired by The University of Texas at Austin Mass Spectrometry Facility

**Data File**	MSF25-2063(EKT122)_hrESIposLC1.d	**Sample Name**	2063(EKT122)	**Comment**	2063(EKT122)
**Position**	P1-D7	**Instrument Name**	6530	**User Name**	
**Acq Method**	LC_C18_pos_jl.m	**Acquired Time**	12/4/2025 1:36:37 PM	**DA Method**	MSF.m

MS Zoomed Spectrum

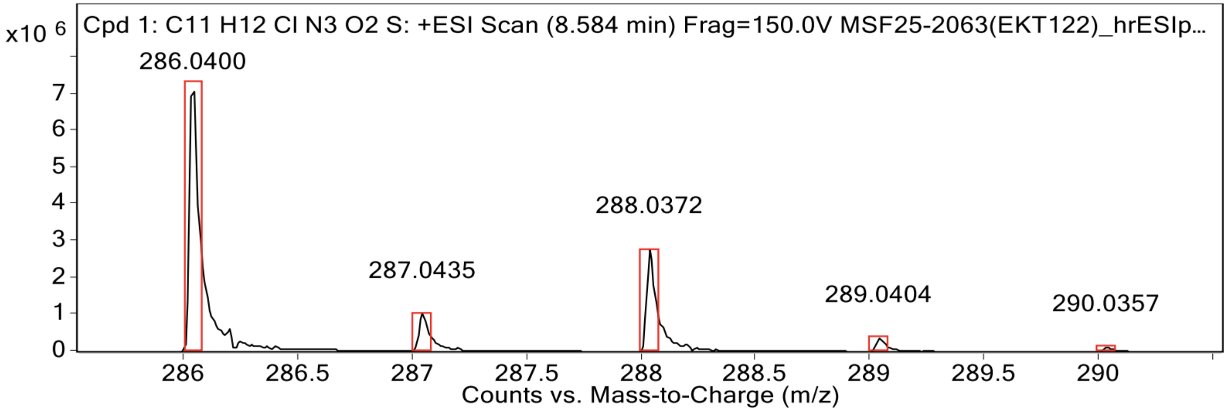

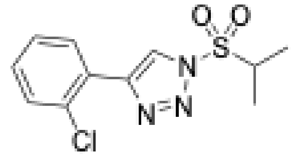

MS Spectrum Peak List

Obs. m/z	Calc. m/z	Charge	Abundance	Formula	Ion Species	Tgt Mass Error (ppm)
180.0313			9057772			
286.0400	286.0412	1	7222646	C11H12ClN3O2S	(M+H)+	3.99
287.0435	287.0438	1	1044582	C11H12ClN3O2S	(M+H)+	1.01
288.0372	288.0383	1	2811036	C11H12ClN3O2S	(M+H)+	4.1
289.0404	289.0409	1	388823	C11H12ClN3O2S	(M+H)+	1.73
290.0357	290.0363	1	141438	C11H12ClN3O2S	(M+H)+	1.97

--- End Of Report ---

Target Compound Screening Report

Results Acquired by The University of Texas at Austin Mass Spectrometry Facility

**Data File**	MSF25-2063(EKT080)_hrESIpos1.d	**Sample Name**	2063(EKT080)	**Comment**	2063(EKT080)
**Position**	P1-D6	**Instrument Name**	6530	**User Name**	
**Acq Method**	FIA_pos.m	**Acquired Time**	12/4/2025 10:53:57 AM	**DA Method**	MSF.m

MS Zoomed Spectrum

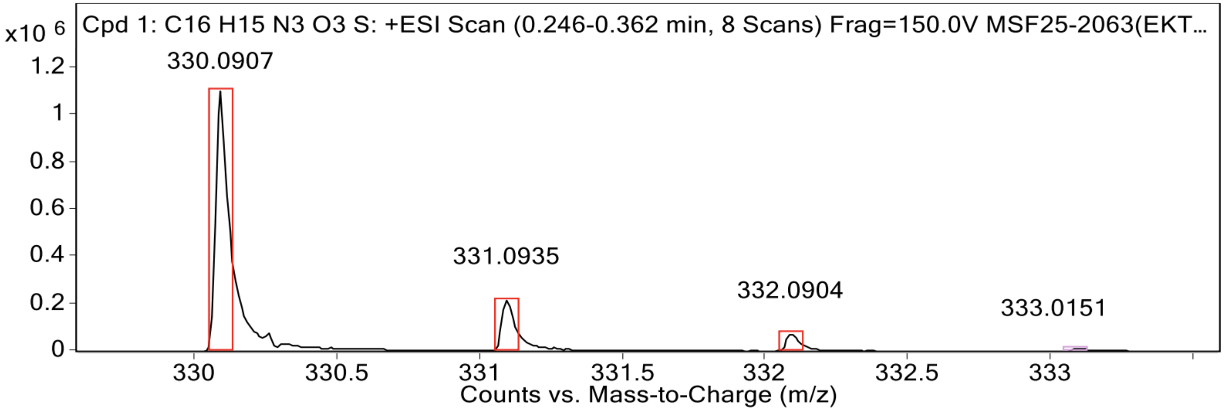

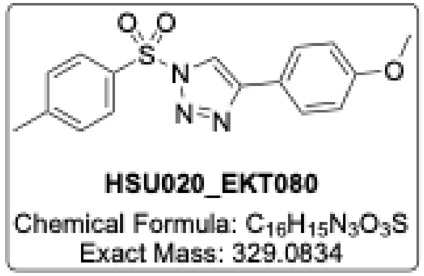

MS Spectrum Peak List

Obs. m/z	Calc. m/z	Charge	Abundance	Formula	Ion Species	Tgt Mass Error (ppm)
330.0907	330.0907	1	1106115	C16H15N3O3S	(M+H)+	-0.07
331.0935	331.0936	1	220098	C16H15N3O3S	(M+H)+	0.11
332.0904	332.0898	1	75988	C16H15N3O3S	(M+H)+	-1.58
333.0151	333.0914	1	825	C16H15N3O3S	(M+H)+	228.98

--- End Of Report ---

Target Compound Screening Report

Results Acquired by The University of Texas at Austin Mass Spectrometry Facility

**Data File**	MSF25-2038(EKT-101)_hrESIpos1.d	**Sample Name**	2038(EKT-101)	**Comment**	2038(EKT-101)
**Position**	P1-A5	**Instrument Name**	6530	**User Name**	
**Acq Method**	FIA_pos.m	**Acquired Time**	11/24/2025 1:57:48 PM	**DA Method**	MSF.m

MS Zoomed Spectrum

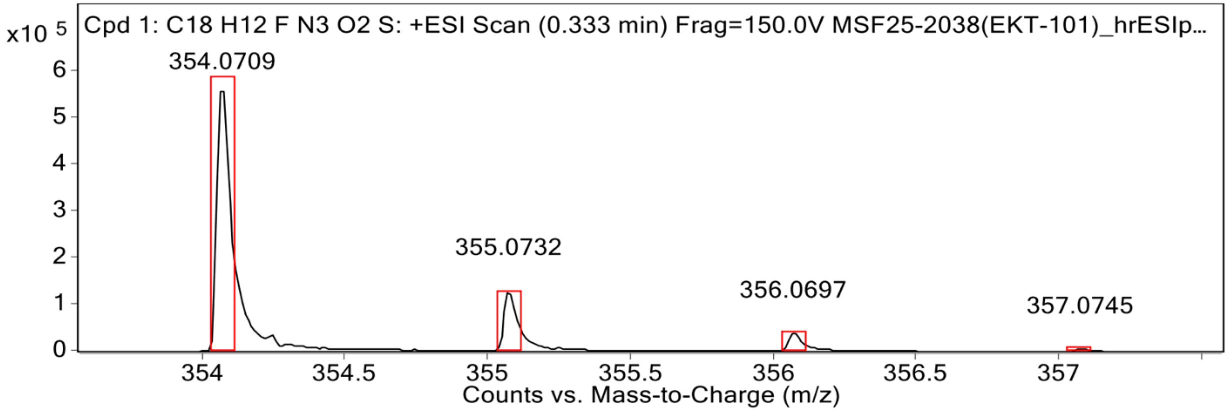

MS Spectrum Peak List

Obs. m/z	Calc. m/z	Charge	Abundance	Formula	Ion Species	Tgt Mass Error (ppm)
354.0709	354.0707	1	581540	C18H12FN3O2S	(M+H)+	-0.48
355.0732	355.0736	1	131547	C18H12FN3O2S	(M+H)+	1.07
356.0697	356.0701	1	40099	C18H12FN3O2S	(M+H)+	1.19
357.0745	357.0714	1	6318	C18H12FN3O2S	(M+H)+	-8.55
364.0203			1881949			

--- End Of Report ---

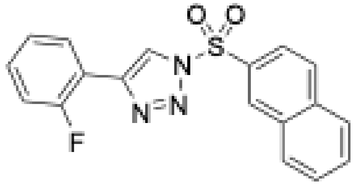

HSU022_EKT101
Chemical Formula: $C_{18}H_{12}FN_3O_2S$
Exact Mass: 353.0634

Target Screening Report

Results Acquired by The University of Texas at Austin Mass Spectrometry Facility

**Data File**	MSF25-2099(AHL009)_hrESIpos1.d	**Sample Name**	2099(AHL009)	**Comment**	2099(AHL009)
**Position**	P1-E6	**Instrument Name**	Instrument 1		
**Acquisition Method**	FIA_Pos.m	**Acquired Time**	12/15/2025 5:07:02 PM (UTC-06:00)	**DA Method**	MSF.m

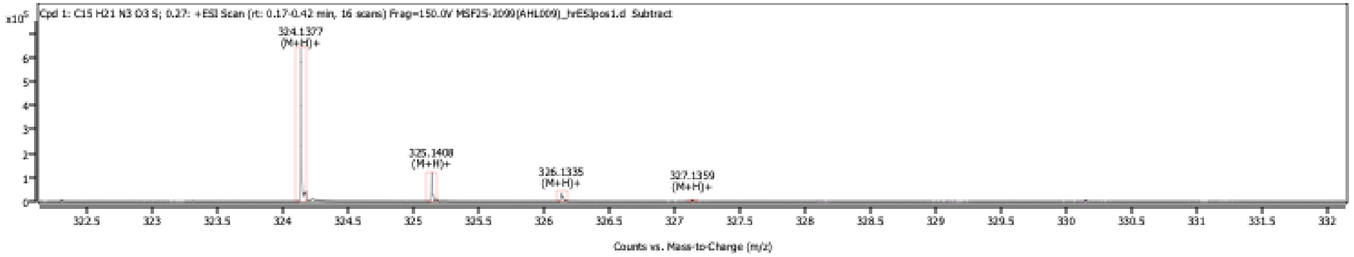

Spectrum Peaks

Obs. m/z	Calc. m/z	Charge	Abund	Formula	Ion Species	Tgt Mass Error (PPM)
324.1377	324.1376	1	640805	C15H21N3O3S	(M+H)+	0.15
325.1408	325.1405	1	122866	C15H21N3O3S	(M+H)+	0.81
326.1335	326.1366	1	34437	C15H21N3O3S	(M+H)+	-9.34
327.1359	327.1383	1	5528	C15H21N3O3S	(M+H)+	-7.10
130.1590			11077853			

MassHunter Qual 10.0
(End of Report)

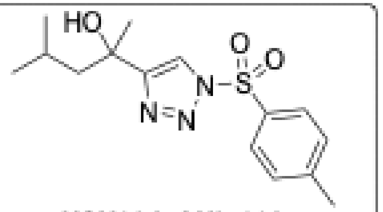

HSU024_AHL-009
Chemical Formula: $C_{15}H_{21}N_3O_3S$
Exact Mass: 323.1304

Target Compound Screening Report

Results Acquired by The University of Texas at Austin Mass Spectrometry Facility

**Data File**	MSF25-2063(JWB164)_hrESIposLC1.d	**Sample Name**	2063(JWB164)	**Comment**	2063(JWB164)
**Position**	P1-C9	**Instrument Name**	6530	**User Name**	
**Acq Method**	LC_C18_pos_jl.m	**Acquired Time**	12/4/2025 12:50:41 PM	**DA Method**	MSF.m

MS Zoomed Spectrum

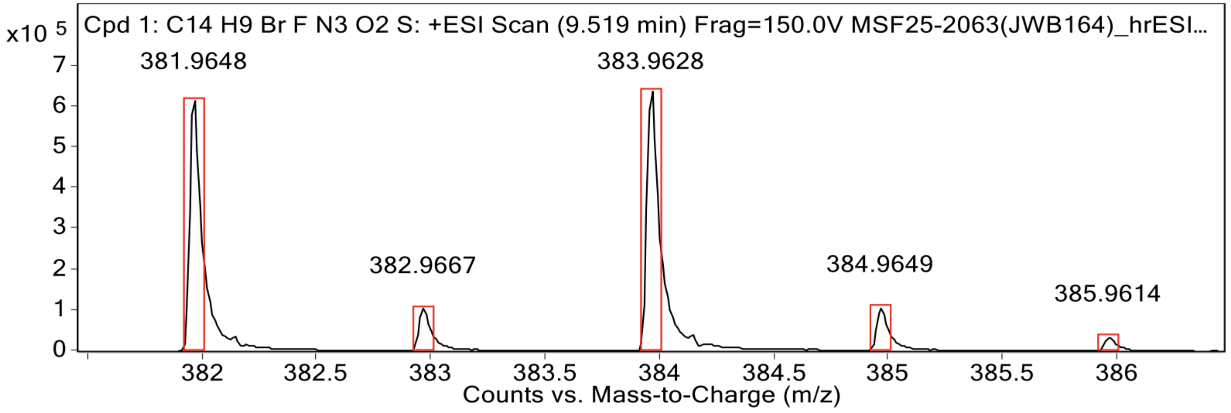

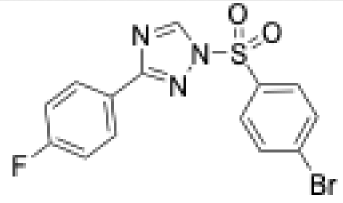

HSU025_JWB164
Chemical Formula: $C_{14}H_{10}BrFN_3O_2S$
Exact Mass: 381.9661

MS Spectrum Peak List

Obs. m/z	Calc. m/z	Charge	Abundance	Formula	Ion Species	Tgt Mass Error (ppm)
381.9648	381.9656	1	619445	C14H9BrFN3O2S	(M+H)+	1.99
382.9667	382.9684	1	105565	C14H9BrFN3O2S	(M+H)+	4.22
383.9628	383.9636	1	642132	C14H9BrFN3O2S	(M+H)+	1.92
384.9649	384.9663	1	108586	C14H9BrFN3O2S	(M+H)+	3.5
385.9614	385.9621	1	36185	C14H9BrFN3O2S	(M+H)+	1.79
386.9641	386.9638	1	4732	C14H9BrFN3O2S	(M+H)+	-0.84

--- End Of Report ---

Target Compound Screening Report

Results Acquired by The University of Texas at Austin Mass Spectrometry Facility

**Data File**	MSF25-2041(JWB-154)_hrESIpos1.d	**Sample Name**	2041(JWB-154)	**Comment**	2041(JWB-154)
**Position**	P1-A8	**Instrument Name**	6530	**User Name**	
**Acq Method**	FIA_pos.m	**Acquired Time**	11/24/2025 2:03:59 PM	**DA Method**	MSF.m

MS Zoomed Spectrum

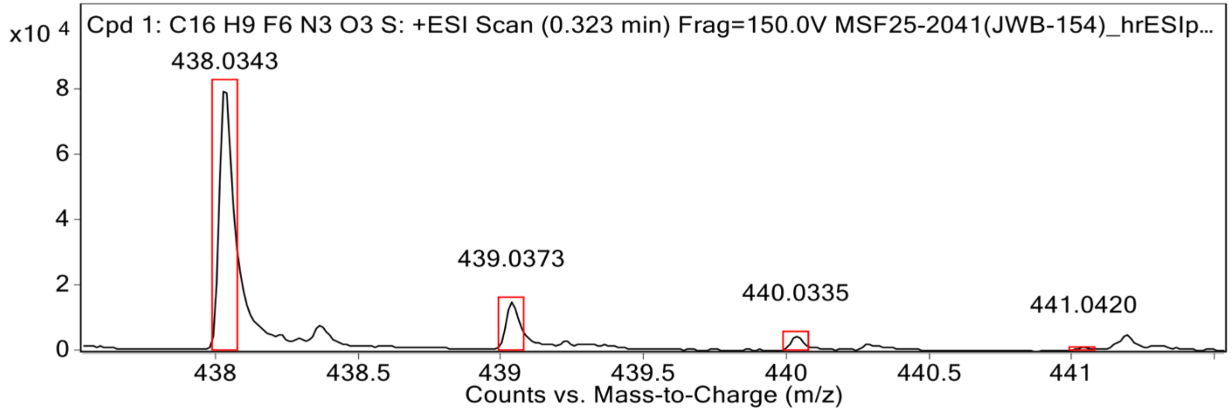

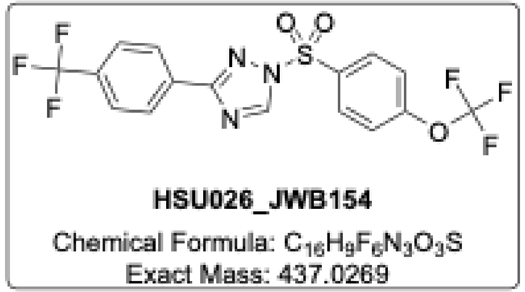

MS Spectrum Peak List

Obs. m/z	Calc. m/z	Charge	Abundance	Formula	Ion Species	Tgt Mass Error (ppm)
436.3627			98431			
438.0343	438.0342	1	82450	C16H9F6N3O3S	(M+H)+	-0.35
439.0373	439.0370	1	15163	C16H9F6N3O3S	(M+H)+	-0.66
440.0335	440.0333	1	4733	C16H9F6N3O3S	(M+H)+	-0.41
441.0420	441.0348	1	1091	C16H9F6N3O3S	(M+H)+	-16.3

--- End Of Report ---

Target Screening Report

Results Acquired by The University of Texas at Austin Mass Spectrometry Facility

**Data File**	MSF25-2099(JWB167)_hrESIpos1.d	**Sample Name**	2099(JWB167)	**Comment**	2099(JWB167)
**Position**	P1-F5	**Instrument Name**	Instrument 1		
**Acquisition Method**	FIA_Pos.m	**Acquired Time**	12/15/2025 5:20:03 PM (UTC-06:00)	**DA Method**	MSF.m

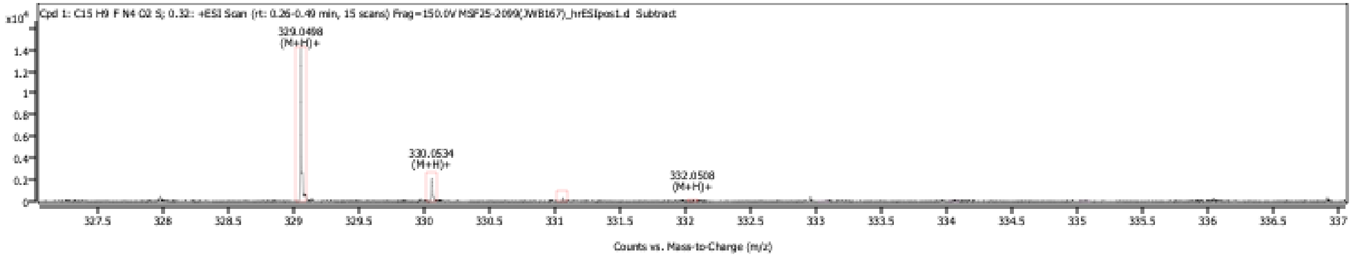

Spectrum Peaks

Obs. m/z	Calc. m/z	Charge	Abund	Formula	Ion Species	Tgt Mass Error (PPM)
329.0498	329.0503	1	14190	C15H9FN4O2S	(M+H)+	-1.62
330.0534	330.0530	1	2114	C15H9FN4O2S	(M+H)+	1.16
332.0508	332.0505	1	51	C15H9FN4O2S	(M+H)+	0.93
130.1590			6756261			

MassHunter Qual 10.0
(End of Report)

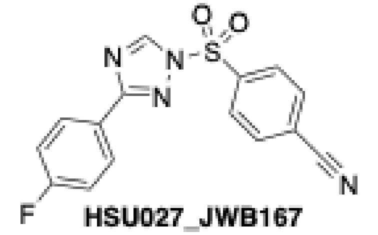

HSU027_JWB167

Chemical Formula: $C_{15}H_9FN_4O_2S$

Exact Mass: 328.0430

Target Compound Screening Report

Results Acquired by The University of Texas at Austin Mass Spectrometry Facility

**Data File**	MSF25-2043(JWB-224)_hrESIpos1.d	**Sample Name**	2043(JWB-224)	**Comment**	2043(JWB-224)
**Position**	P1-B1	**Instrument Name**	6530	**User Name**	
**Acq Method**	FIA_pos.m	**Acquired Time**	11/24/2025 2:08:00 PM	**DA Method**	MSF.m

MS Zoomed Spectrum

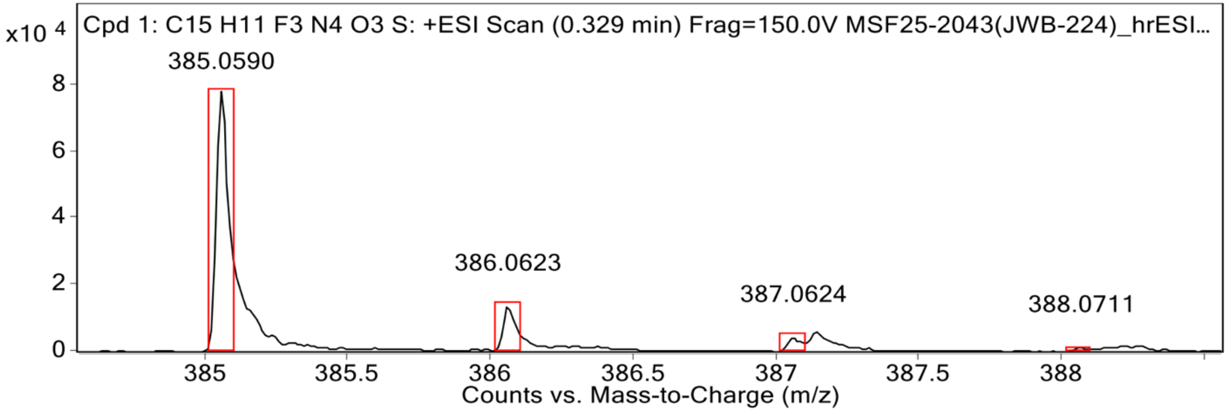

MS Spectrum Peak List

Obs. m/z	Calc. m/z	Charge	Abundance	Formula	Ion Species	Tgt Mass Error (ppm)
385.0590	385.0577	1	78326	C15H11F3N4O3S	(M+H)+	-3.57
386.0623	386.0604	1	13764	C15H11F3N4O3S	(M+H)+	-4.87
387.0624	387.0566	1	4353	C15H11F3N4O3S	(M+H)+	-15.07
388.0711	388.0581	1	1326	C15H11F3N4O3S	(M+H)+	-33.48
389.1370	389.0593	1	1675	C15H11F3N4O3S	(M+H)+	-199.73

--- End Of Report ---

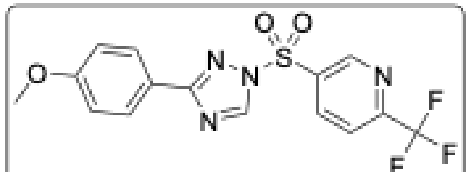

HSU028_JWB224

Chemical Formula: $C_{15}H_{11}F_3N_4O_3S$

Target Compound Screening Report

Results Acquired by The University of Texas at Austin Mass Spectrometry Facility

**Data File**	MSF25-2063(JWB221)_hrESIpos1.d	**Sample Name**	2063(JWB221)	**Comment**	2063(JWB221)
**Position**	P1-D2	**Instrument Name**	6530	**User Name**	
**Acq Method**	FIA_pos.m	**Acquired Time**	12/4/2025 10:45:54 AM	**DA Method**	MSF.m

MS Zoomed Spectrum

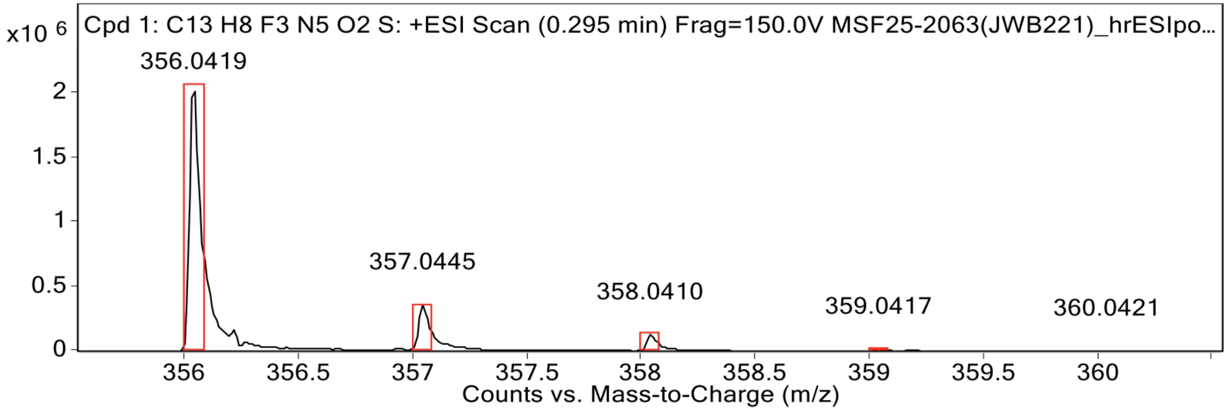

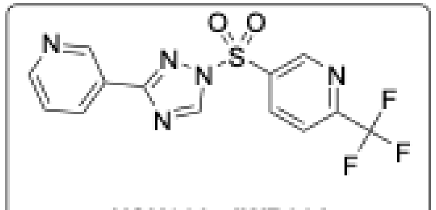

Chemical Formula: $C_{13}H_8F_3N_5O_2S$
Exact Mass: 355.0351

MS Spectrum Peak List

Obs. m/z	Calc. m/z	Charge	Abundance	Formula	Ion Species	Tgt Mass Error (ppm)
356.0419	356.0424	1	2054974	C13H8F3N5O2S	(M+H)+	1.31
357.0445	357.0449	1	360844	C13H8F3N5O2S	(M+H)+	0.89
358.0410	358.0407	1	127975	C13H8F3N5O2S	(M+H)+	-0.94
359.0417	359.0422	1	16257	C13H8F3N5O2S	(M+H)+	1.35
360.0421	360.0433	1	2193	C13H8F3N5O2S	(M+H)+	3.32

--- End Of Report ---

Target Compound Screening Report

Results Acquired by The University of Texas at Austin Mass Spectrometry Facility

**Data File**	MSF25-2040(JWB-123)_hrESIposLC1.d	**Sample Name**	Unavailable	**Comment**	Sample information is unavailable
**Position**	Unavailable	**Instrument Name**	Unavailable	**User Name**	Unavailable
**Acq Method**		**Acquired Time**	Unavailable	**DA Method**	MSF.m

MS Zoomed Spectrum

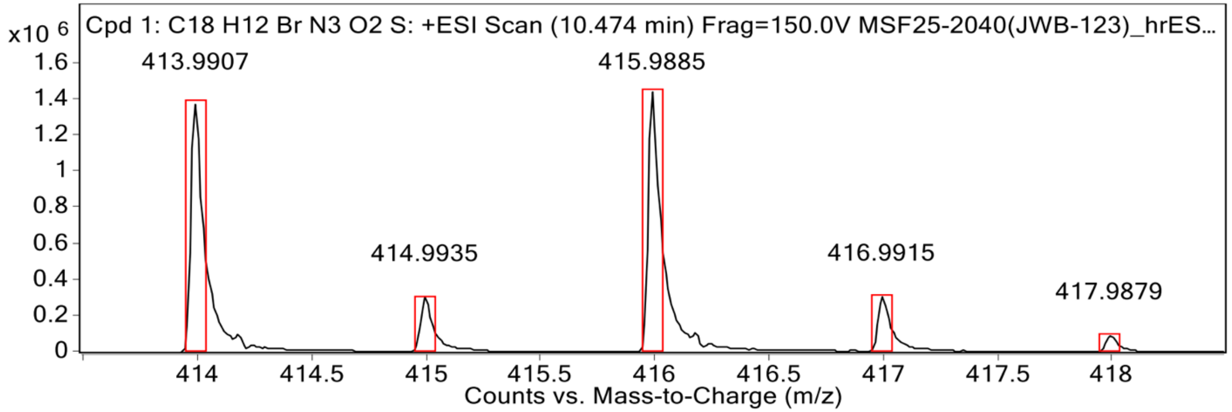

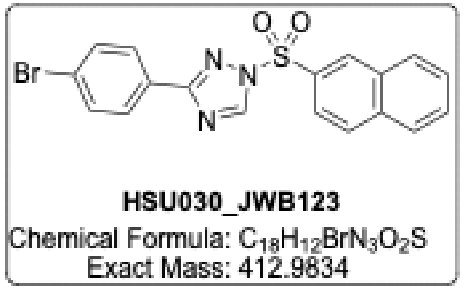

MS Spectrum Peak List

Obs. m/z	Calc. m/z	Charge	Abundance	Formula	Ion Species	Tgt Mass Error (ppm)
413.9907	413.9906	1	1377179	C18H12BrN3O2S	(M+H)+	-0.06
414.9935	414.9935	1	311013	C18H12BrN3O2S	(M+H)+	0.14
415.9885	415.9887	1	1446529	C18H12BrN3O2S	(M+H)+	0.37
416.9915	416.9915	1	314084	C18H12BrN3O2S	(M+H)+	0.05
417.9879	417.9881	1	99280	C18H12BrN3O2S	(M+H)+	0.58

--- End Of Report ---

Target Screening Report

Results Acquired by The University of Texas at Austin Mass Spectrometry Facility

**Data File**	MSF25-2099(JWB209)_hrESIpos1.d	**Sample Name**	2099(JWB209)	**Comment**	2099(JWB209)
**Position**	P1-F6	**Instrument Name**	Instrument 1		
**Acquisition Method**	FIA_Pos.m	**Acquired Time**	12/15/2025 5:21:40 PM (UTC-06:00)	**DA Method**	MSF.m

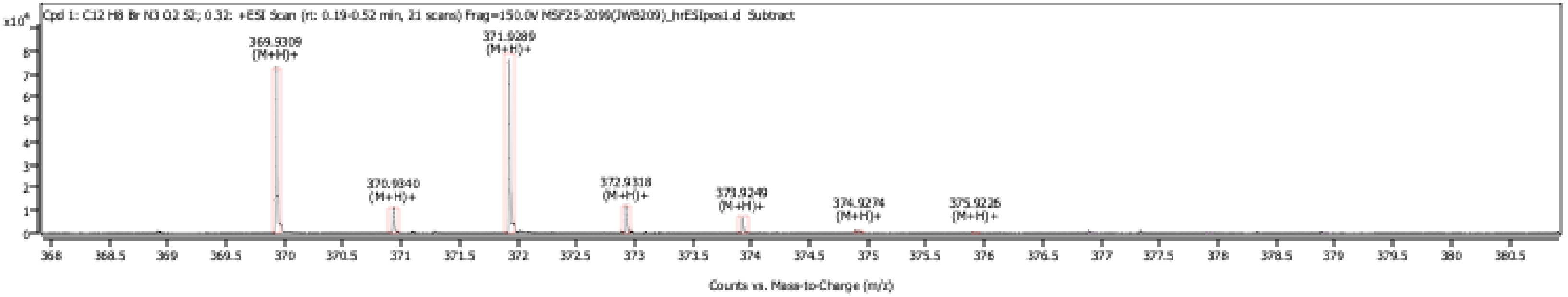

Spectrum Peaks

Obs. m/z	Calc. m/z	Charge	Abund	Formula	Ion Species	Tgt Mass Error (PPM)
369.9309	369.9314	1	74221	C12H8BrN3O2S2	(M+H)+	-1.48
370.9340	370.9340	1	11603	C12H8BrN3O2S2	(M+H)+	0.05
371.9289	371.9293	1	77349	C12H8BrN3O2S2	(M+H)+	-1.03
372.9318	372.9318	1	12417	C12H8BrN3O2S2	(M+H)+	-0.07
373.9249	373.9265	1	6998	C12H8BrN3O2S2	(M+H)+	-4.35
374.9274	374.9286	1	1213	C12H8BrN3O2S2	(M+H)+	-3.03
375.9226	375.9249	1	216	C12H8BrN3O2S2	(M+H)+	-6.13
130.1589			10468549			

MassHunter Qual 10.0
(End of Report)

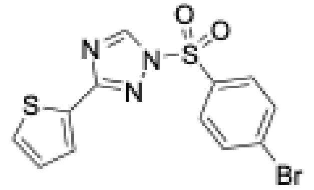

HSU032_JWB209

Chemical Formula: $C_{12}H_8BrN_3O_2S_2$
Exact Mass: 368.9241

Target Screening Report

Results Acquired by The University of Texas at Austin Mass Spectrometry Facility

**Data File**	MSF25-2143(AHL-014-2)_hrESIposLC1.d	**Sample Name**		**Comment**	
**Position**		**Instrument Name**			
**Acquisition Method**		**Acquired Time**	12/19/2025 2:56:13 PM (UTC-06:00)	**DA Method**	MSF.m

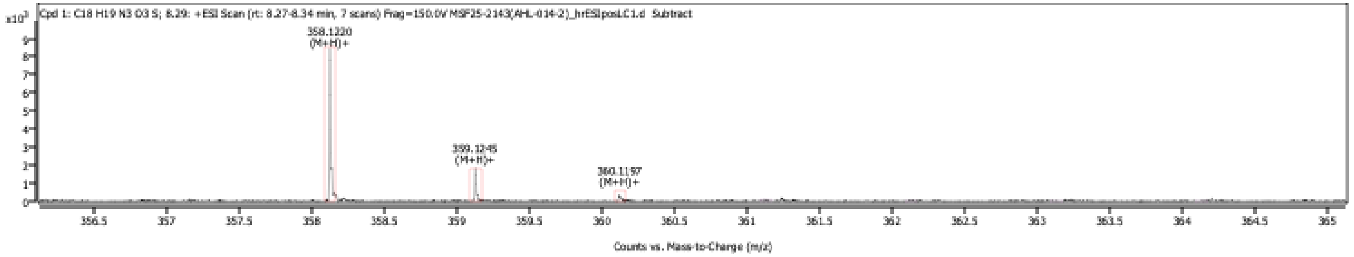

Spectrum Peaks

Obs. m/z	Calc. m/z	Charge	Abund	Formula	Ion Species	Tgt Mass Error (PPM)
358.1220	358.1220	1	8499	C18H19N3O3S	(M+H)+	0.01
359.1245	359.1249	1	1859	C18H19N3O3S	(M+H)+	-1.18
360.1197	360.1216	1	377	C18H19N3O3S	(M+H)+	-5.17

MassHunter Qual 10.0
(End of Report)

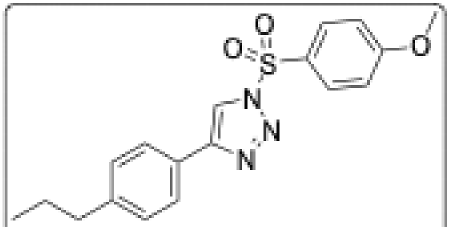

HSU037_AHL-014
Chemical Formula: $C_{18}H_{19}N_3O_3S$
Exact Mass: 357.1147

Target Compound Screening Report

Results Acquired by The University of Texas at Austin Mass Spectrometry Facility

**Data File**	MSF25-2063(AHL017)_hrESIpos1.d	**Sample Name**	2063(AHL017)	**Comment**	2063(AHL017)
**Position**	P1-E3	**Instrument Name**	6530	**User Name**	
**Acq Method**	FIA_pos.m	**Acquired Time**	12/4/2025 11:06:02 AM	**DA Method**	MSF.m

MS Zoomed Spectrum

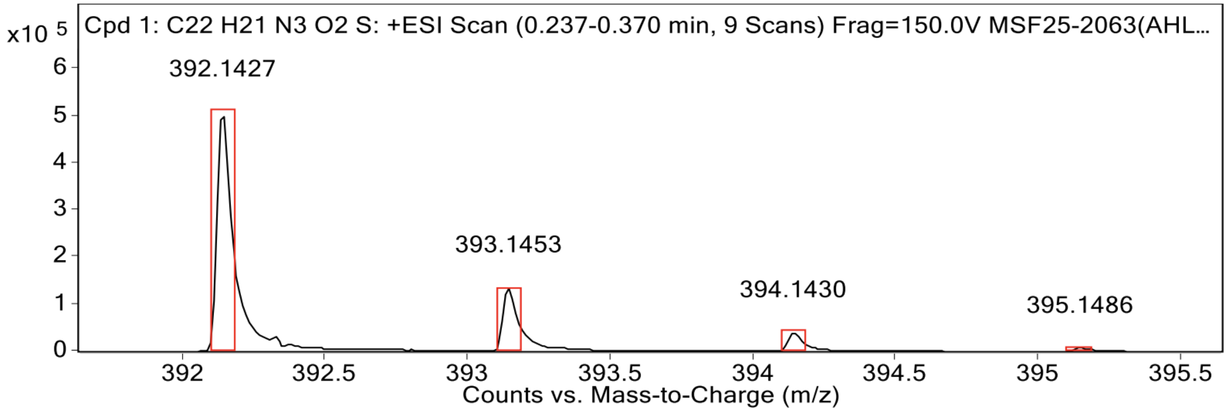

MS Spectrum Peak List

Obs. m/z	Calc. m/z	Charge	Abundance	Formula	Ion Species	Tgt Mass Error (ppm)
386.1182			1176102			
392.1427	392.1427	1	510087	C22H21N3O2S	(M+H)+	0.15
393.1453	393.1457	1	136366	C22H21N3O2S	(M+H)+	1.18
394.1430	394.1430	1	40763	C22H21N3O2S	(M+H)+	0
395.1486	395.1440	1	7586	C22H21N3O2S	(M+H)+	-11.75

--- End Of Report ---

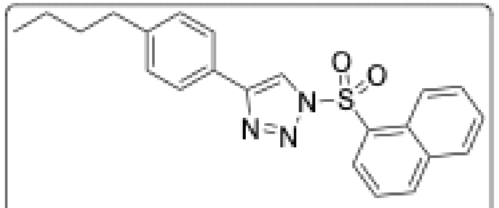

HSU038_AHL-017

Chemical Formula: $C_{22}H_{21}N_3O_2S$

Exact Mass: 391.1354

Target Screening Report

Results Acquired by The University of Texas at Austin Mass Spectrometry Facility

**Data File**	MSF25-2099(AHL021)_hrESIpos1.d	**Sample Name**	2099(AHL021)	**Comment**	2099(AHL021)
**Position**	P1-E7	**Instrument Name**	Instrument 1		
**Acquisition Method**	FIA_Pos.m	**Acquired Time**	12/15/2025 5:08:39 PM (UTC-06:00)	**DA Method**	MSF.m

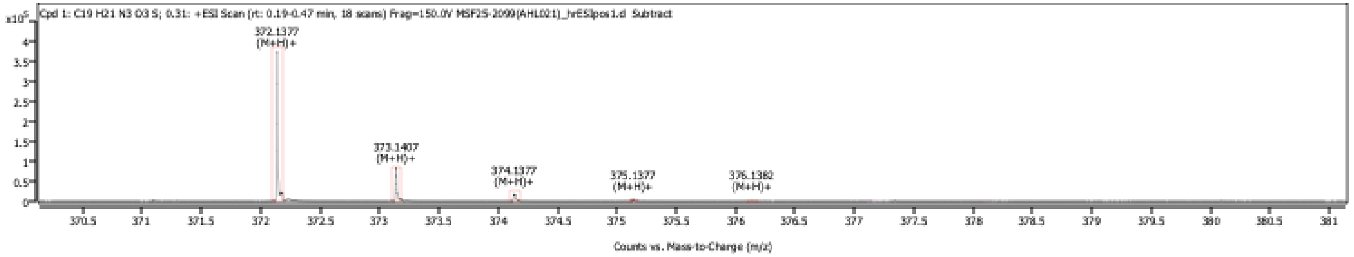

Spectrum Peaks

Obs. m/z	Calc. m/z	Charge	Abund	Formula	Ion Species	Tgt Mass Error (PPM)
372.1377	372.1376	1	385128	C19H21N3O3S	(M+H)+	0.05
373.1407	373.1406	1	86521	C19H21N3O3S	(M+H)+	0.36
374.1377	374.1374	1	19401	C19H21N3O3S	(M+H)+	0.61
375.1377	375.1387	1	4561	C19H21N3O3S	(M+H)+	-2.62
376.1382	376.1401	1	749	C19H21N3O3S	(M+H)+	-5.04
130.1590			9895309			

MassHunter Qual 10.0
(End of Report)

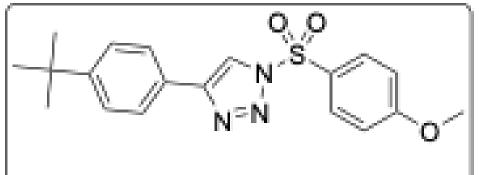

HSU041_AHL-021

Chemical Formula: $C_{19}H_{21}N_3O_3S$

Exact Mass: 371.1304

Target Screening Report

Results Acquired by The University of Texas at Austin Mass Spectrometry Facility

**Data File**	MSF25-2099(DML189)_hrESIpos1.d	**Sample Name**	2099(DML189)	**Comment**	2099(DML189)
**Position**	P1-F4	**Instrument Name**	Instrument 1		
**Acquisition Method**	FIA_Pos.m	**Acquired Time**	12/15/2025 5:18:26 PM (UTC-06:00)	**DA Method**	MSF.m

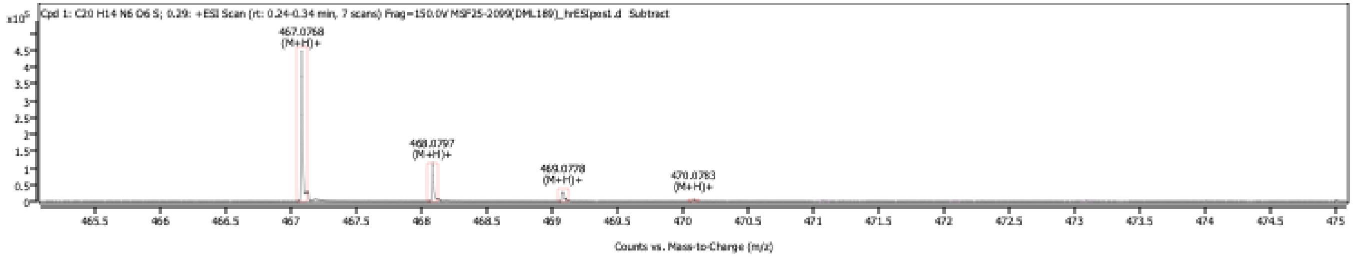

Spectrum Peaks

Obs. m/z	Calc. m/z	Charge	Abund	Formula	Ion Species	Tgt Mass Error (PPM)
467.0768	467.0768	1	458293	C20H14N6O6S	(M+H)+	-0.09
468.0797	468.0795	1	116254	C20H14N6O6S	(M+H)+	0.40
469.0778	469.0771	1	26809	C20H14N6O6S	(M+H)+	1.42
470.0783	470.0784	1	5894	C20H14N6O6S	(M+H)+	-0.09
130.1589			11053639			

MassHunter Qual 10.0
(End of Report)

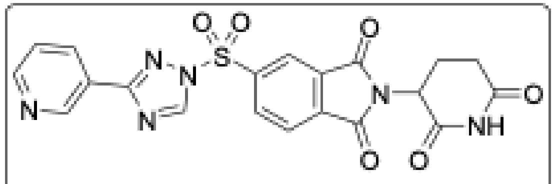

HSU044_DML 189

Chemical Formula: $C_{20}H_{14}N_6O_6S$
Exact Mass: 466.0696

Target Compound Screening Report

Results Acquired by The University of Texas at Austin Mass Spectrometry Facility

**Data File**	MSF25-2087(DML367)_hrESIposLC1.d	**Sample Name**	2087(DML367)	**Comment**	2087(DML367)
**Position**	P1-D5	**Instrument Name**	6530	**User Name**	
**Acq Method**	LC_C18_pos_jl.m	**Acquired Time**	12/4/2025 9:15:35 PM	**DA Method**	MSF.m

MS Zoomed Spectrum

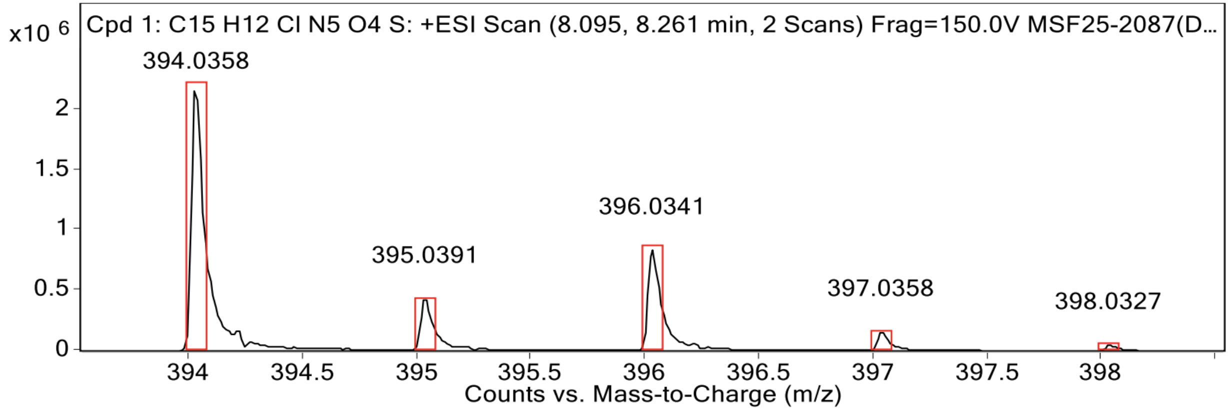

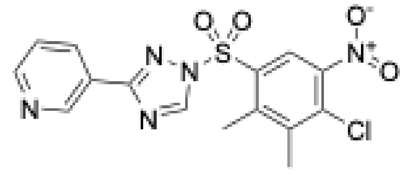

HSU050_DML367

Chemical Formula: $C_{15}H_{12}ClN_5O_4S$

Exact Mass: 393.0299

MS Spectrum Peak List

Obs. m/z	Calc. m/z	Charge	Abundance	Formula	Ion Species	Tgt Mass Error (ppm)
394.0358	394.0371	1	2211590	C15H12ClN5O4S	(M+H)+	3.32
395.0391	395.0397	1	437564	C15H12ClN5O4S	(M+H)+	1.76
396.0341	396.0345	1	840389	C15H12ClN5O4S	(M+H)+	1.19
397.0358	397.0369	1	162511	C15H12ClN5O4S	(M+H)+	2.95
398.0327	398.0336	1	53848	C15H12ClN5O4S	(M+H)+	2.23

--- End Of Report ---

Target Compound Screening Report

Results Acquired by The University of Texas at Austin Mass Spectrometry Facility

Data File MSF25-2036(AHL-001)_hrESIpos1.d **Sample Name** 2036(AHL-001) **Comment** 2036(AHL-001)
Position P1-A3 **Instrument Name** 6530 **User Name**
Acq Method FIA_pos.m **Acquired Time** 11/24/2025 1:53:43 PM **DA Method** MSF.m

MS Zoomed Spectrum

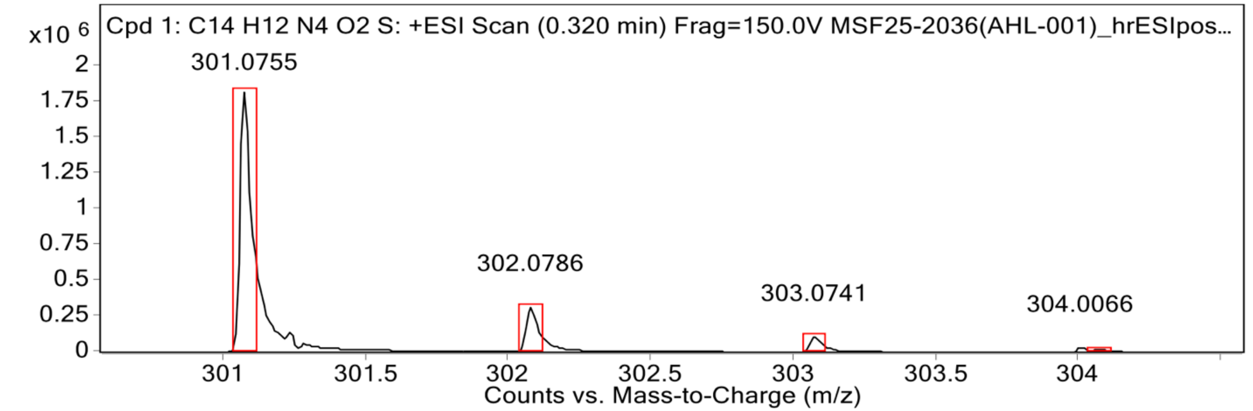

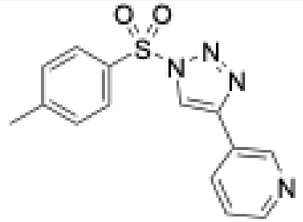

HSU053_AHL-001
Chemical Formula: $C_{14}H_{12}N_4O_2S$
Exact Mass: 300.0681

MS Spectrum Peak List

Obs. m/z	Calc. m/z	Charge	Abundance	Formula	Ion Species	Tgt Mass Error (ppm)
301.0755	301.0754	1	1815759	C14H12N4O2S	(M+H)+	-0.28
302.0786	302.0781	1	320167	C14H12N4O2S	(M+H)+	-1.76
303.0741	303.0739	1	110800	C14H12N4O2S	(M+H)+	-0.62
304.0066	304.0755	1	35131	C14H12N4O2S	(M+H)+	226.51
305.0077	305.0766	1	6387	C14H12N4O2S	(M+H)+	225.89

--- End Of Report ---

Target Compound Screening Report

Results Acquired by The University of Texas at Austin Mass Spectrometry Facility

**Data File**	MSF25-2063(AHL010)_hrESIpos1.d	**Sample Name**	2063(AHL010)	**Comment**	2063(AHL010)
**Position**	P1-D9	**Instrument Name**	6530	**User Name**	
**Acq Method**	FIA_pos.m	**Acquired Time**	12/4/2025 11:00:01 AM	**DA Method**	MSF.m

MS Zoomed Spectrum

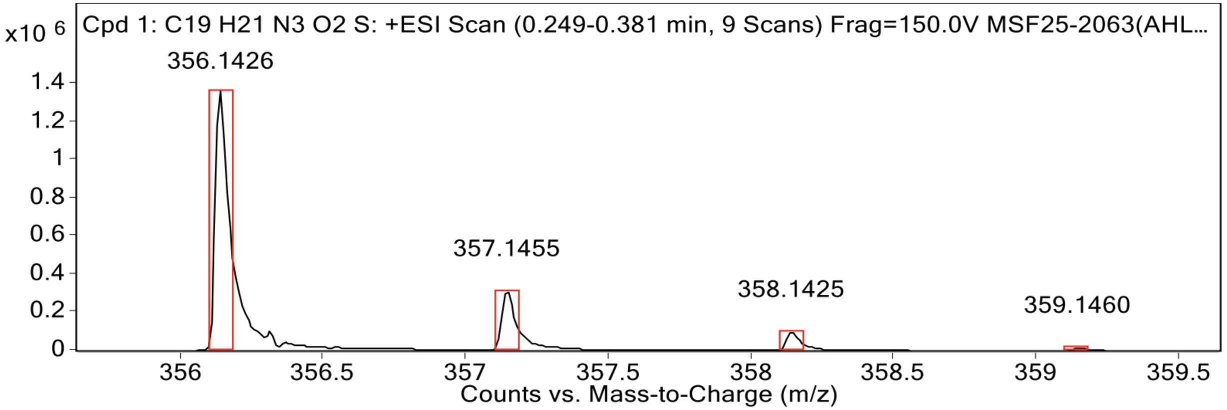

MS Spectrum Peak List

Obs. m/z	Calc. m/z	Charge	Abundance	Formula	Ion Species	Tgt Mass Error (ppm)
350.1180			2843358			
356.1426	356.1427	1	1362227	C19H21N3O2S	(M+H)+	0.32
357.1455	357.1457	1	311766	C19H21N3O2S	(M+H)+	0.45
358.1425	358.1424	1	97903	C19H21N3O2S	(M+H)+	-0.37
359.1460	359.1436	1	15085	C19H21N3O2S	(M+H)+	-6.87

--- End Of Report ---

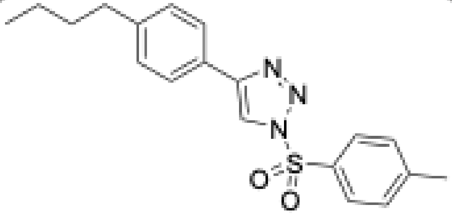

HSU054_AHL-010
Chemical Formula: $C_{19}H_{21}N_3O_2S$
Exact Mass: 355.1354

Target Compound Screening Report

Results Acquired by The University of Texas at Austin Mass Spectrometry Facility

**Data File**	MSF25-2063(AHL020)_hrESIpos1.d	**Sample Name**	2063(AHL020)	**Comment**	2063(AHL020)
**Position**	P1-E4	**Instrument Name**	6530	**User Name**	
**Acq Method**	FIA_pos.m	**Acquired Time**	12/4/2025 11:08:03 AM	**DA Method**	MSF.m

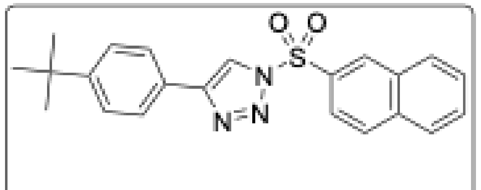

MS Zoomed Spectrum

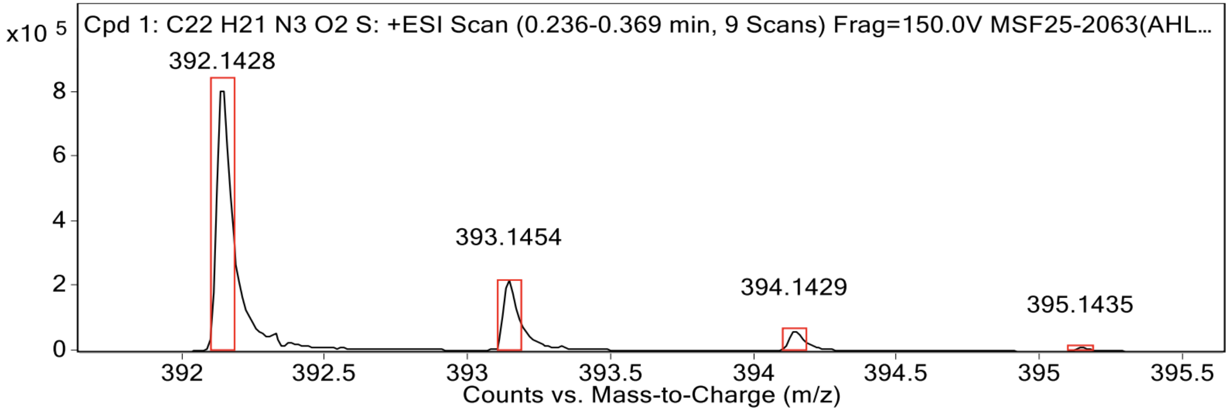

MS Spectrum Peak List

Obs. m/z	Calc. m/z	Charge	Abundance	Formula	Ion Species	Tgt Mass Error (ppm)
386.1178			2835460			
392.1428	392.1427	1	839543	C22H21N3O2S	(M+H)+	-0.2
393.1454	393.1457	1	220208	C22H21N3O2S	(M+H)+	0.83
394.1429	394.1430	1	65407	C22H21N3O2S	(M+H)+	0.34
395.1435	395.1440	1	11697	C22H21N3O2S	(M+H)+	1.21

--- End Of Report ---

Target Screening Report

Results Acquired by The University of Texas at Austin Mass Spectrometry Facility

**Data File**	MSF25-2099(AHL032)_hrESIpos1.d	**Sample Name**	2099(AHL032)	**Comment**	2099(AHL032)
**Position**	P1-E8	**Instrument Name**	Instrument 1		
**Acquisition Method**	FIA_Pos.m	**Acquired Time**	12/15/2025 5:10:18 PM (UTC-06:00)	**DA Method**	MSF.m

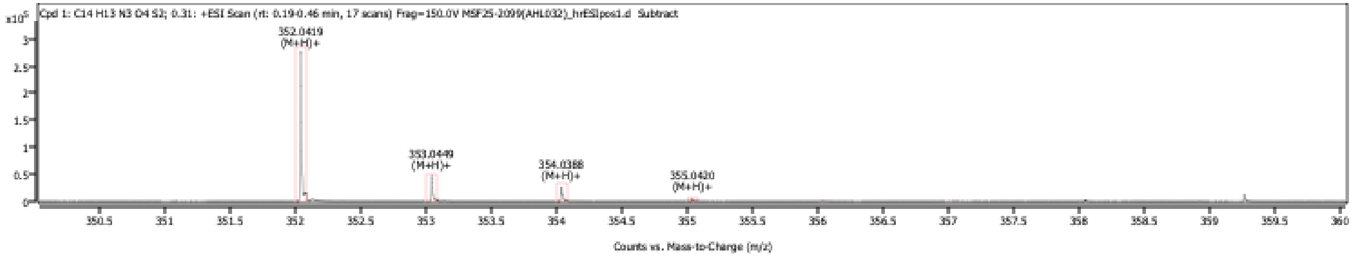

Spectrum Peaks

Obs. m/z	Calc. m/z	Charge	Abund	Formula	Ion Species	Tgt Mass Error (PPM)
352.0419	352.0420	1	284062	C14H13N3O4S2	(M+H)+	-0.33
353.0449	353.0447	1	49068	C14H13N3O4S2	(M+H)+	0.55
354.0388	354.0397	1	27153	C14H13N3O4S2	(M+H)+	-2.72
355.0420	355.0418	1	3976	C14H13N3O4S2	(M+H)+	0.77
130.1590			9651942			

MassHunter Qual 10.0
(End of Report)

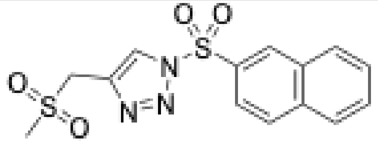

HSU058_AHL-032

Chemical Formula: $C_{14}H_{13}N_3O_4S_2$

Exact Mass: 351.0347

Target Screening Report

Results Acquired by The University of Texas at Austin Mass Spectrometry Facility

**Data File**	MSF25-2129(AHL033)_hrESIposLC1.d	**Sample Name**	2129(AHL033)	**Comment**	2129(AHL033)
**Position**	P1-C3	**Instrument Name**	Instrument 1		
**Acquisition Method**	LC_C18_CH3CN_JL.m	**Acquired Time**	12/18/2025 8:37:03 PM (UTC-06:00)	**DA Method**	MSF.m

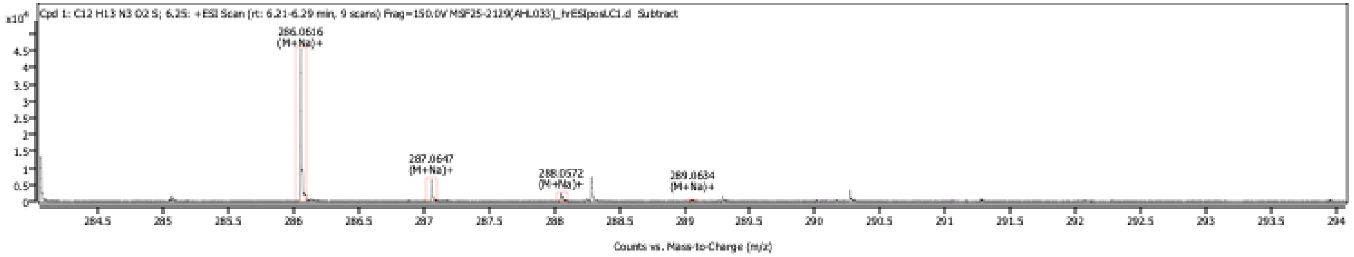

Spectrum Peaks

Obs. m/z	Calc. m/z	Charge	Abund	Formula	Ion Species	Tgt Mass Error (PPM)
286.0616	286.0621	1	45887	C12H13N3O2S	(M+Na)+	-1.75
287.0647	287.0648	1	6431	C12H13N3O2S	(M+Na)+	-0.30
288.0572	288.0602	1	2512	C12H13N3O2S	(M+Na)+	-10.44
289.0634	289.0620	1	351	C12H13N3O2S	(M+Na)+	4.81
200.1182			5713466			

MassHunter Qual 10.0
(End of Report)

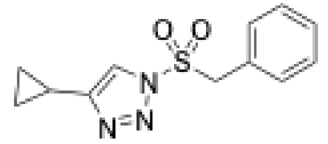

HSU059_AHL-033
Chemical Formula: $C_{12}H_{13}N_3O_2S$
Exact Mass: 263.0728

Target Compound Screening Report

Results Acquired by The University of Texas at Austin Mass Spectrometry Facility

**Data File**	MSF25-2087(AHL035)_hrESIposLC1.d	**Sample Name**	2087(AHL035)	**Comment**	2087(AHL035)
**Position**	P1-C6	**Instrument Name**	6530	**User Name**	
**Acq Method**	LC_C18_pos_jl.m	**Acquired Time**	12/4/2025 6:11:50 PM	**DA Method**	MSF.m

MS Zoomed Spectrum

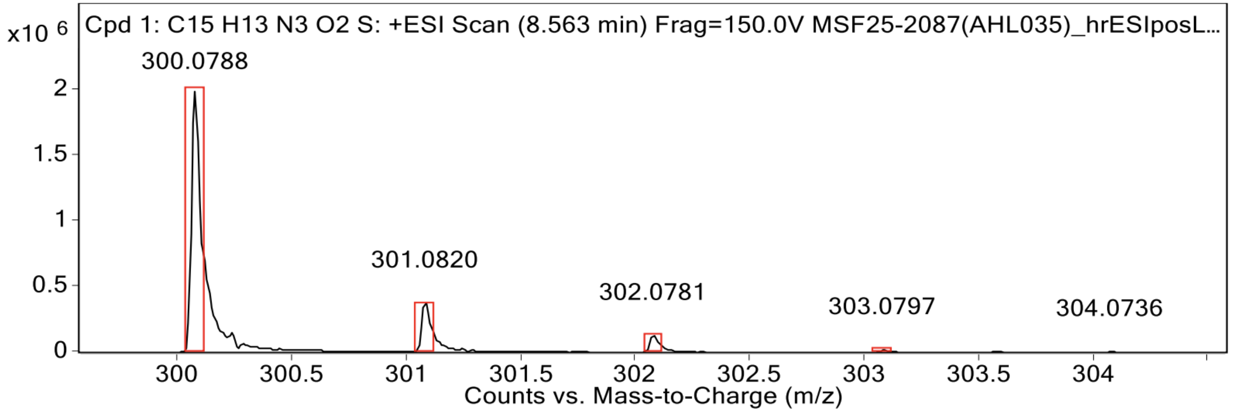

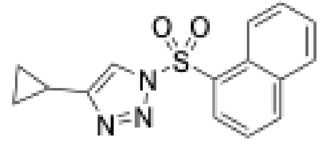

HSU060_AHL-035
Chemical Formula: $C_{15}H_{13}N_3O_2S$
Exact Mass: 299.0728

MS Spectrum Peak List

Obs. m/z	Calc. m/z	Charge	Abundance	Formula	Ion Species	Tgt Mass Error (ppm)
300.0788	300.0801	1	1995820	C15H13N3O2S	(M+H)+	4.34
301.0820	301.0830	1	378066	C15H13N3O2S	(M+H)+	3.25
302.0781	302.0789	1	130701	C15H13N3O2S	(M+H)+	2.68
303.0797	303.0804	1	21773	C15H13N3O2S	(M+H)+	2.34
304.0736	304.0817	1	7650	C15H13N3O2S	(M+H)+	26.61
305.0780	305.0833	1	1398	C15H13N3O2S	(M+H)+	17.4

--- End Of Report ---

Target Compound Screening Report

Results Acquired by The University of Texas at Austin Mass Spectrometry Facility

**Data File**	MSF25-2087(AHL038)_hrESIposLC1.d	**Sample Name**	2087(AHL038)	**Comment**	2087(AHL038)
**Position**	P1-C7	**Instrument Name**	6530	**User Name**	
**Acq Method**	LC_C18_pos_jl.m	**Acquired Time**	12/4/2025 6:34:49 PM	**DA Method**	MSF.m

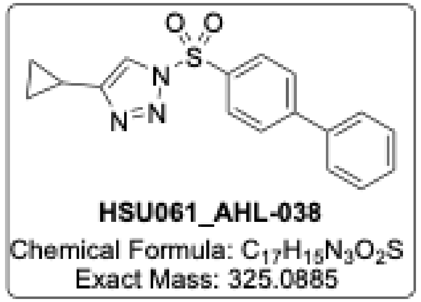

MS Zoomed Spectrum

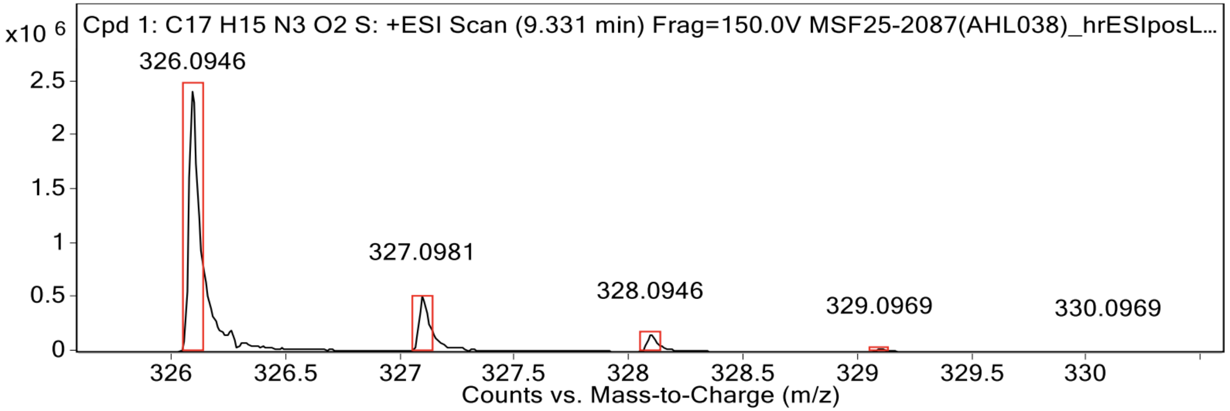

MS Spectrum Peak List

Obs. m/z	Calc. m/z	Charge	Abundance	Formula	Ion Species	Tgt Mass Error (ppm)
326.0946	326.0958	1	2479350	C17H15N3O2S	(M+H)+	3.72
327.0981	327.0987	1	524298	C17H15N3O2S	(M+H)+	1.68
328.0946	328.0950	1	164961	C17H15N3O2S	(M+H)+	1.03
329.0969	329.0964	1	25706	C17H15N3O2S	(M+H)+	-1.78
330.0969	330.0977	1	2744	C17H15N3O2S	(M+H)+	2.54

--- End Of Report ---

Target Compound Screening Report

Results Acquired by The University of Texas at Austin Mass Spectrometry Facility

**Data File**	MSF25-2087(AHL7004A)_hrESIposLC1.d	**Sample Name**	2087(AHL7004A)	**Comment**	2087(AHL7004A)
**Position**	P1-D2	**Instrument Name**	6530	**User Name**	
**Acq Method**	LC_C18_pos_jl.m	**Acquired Time**	12/4/2025 8:06:41 PM	**DA Method**	MSF.m

MS Zoomed Spectrum

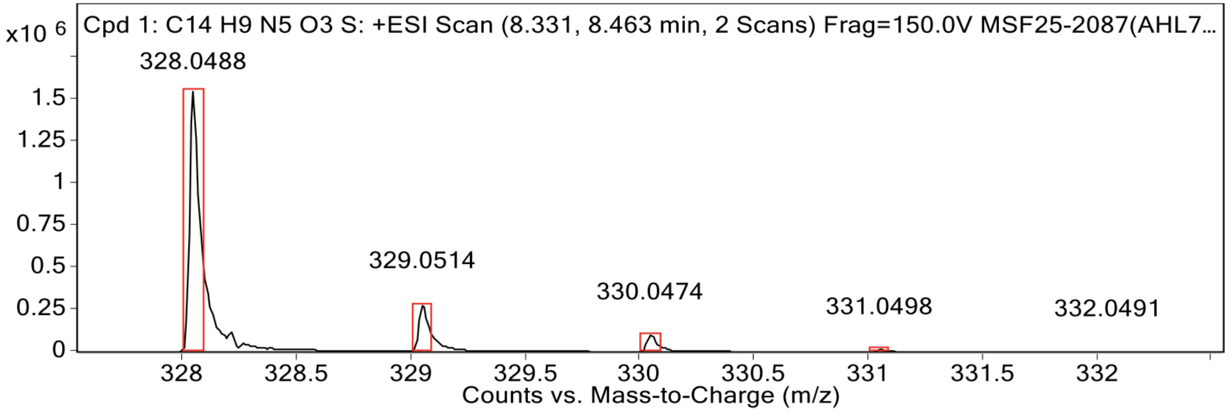

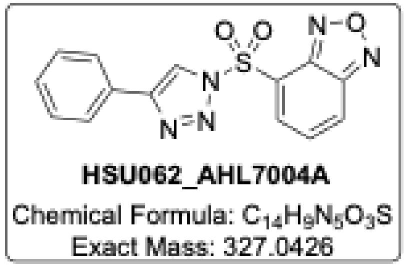

MS Spectrum Peak List

Obs. m/z	Calc. m/z	Charge	Abundance	Formula	Ion Species	Tgt Mass Error (ppm)
328.0488	328.0499	1	1550059	C14H9N5O3S	(M+H)+	3.46
329.0514	329.0524	1	287820	C14H9N5O3S	(M+H)+	3.25
330.0474	330.0486	1	102204	C14H9N5O3S	(M+H)+	3.74
331.0498	331.0501	1	14026	C14H9N5O3S	(M+H)+	1.05
332.0491	332.0512	1	1889	C14H9N5O3S	(M+H)+	6.3

--- End Of Report ---

Target Compound Screening Report

Results Acquired by The University of Texas at Austin Mass Spectrometry Facility

**Data File**	MSF25-2087(AHL7004B)_hrESIposLC1.d	**Sample Name**	2087(AHL7004B)	**Comment**	2087(AHL7004B)
**Position**	P1-D3	**Instrument Name**	6530	**User Name**	
**Acq Method**	LC_C18_pos_jl.m	**Acquired Time**	12/4/2025 8:29:36 PM	**DA Method**	MSF.m

MS Zoomed Spectrum

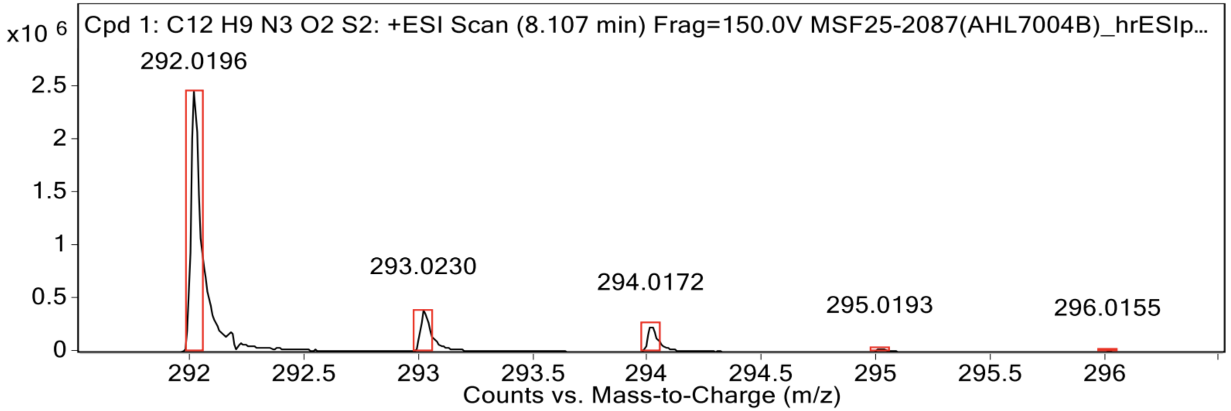

MS Spectrum Peak List

Obs. m/z	Calc. m/z	Charge	Abundance	Formula	Ion Species	Tgt Mass Error (ppm)
292.0196	292.0209	1	2463582	C12H9N3O2S2	(M+H)+	4.5
293.0230	293.0234	1	400507	C12H9N3O2S2	(M+H)+	1.52
294.0172	294.0181	1	251014	C12H9N3O2S2	(M+H)+	2.88
295.0193	295.0201	1	34946	C12H9N3O2S2	(M+H)+	2.81
296.0155	296.0164	1	8654	C12H9N3O2S2	(M+H)+	2.96

--- End Of Report ---

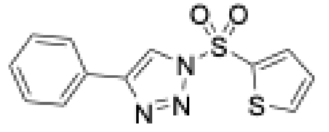

HSU063_AHL7004B

Chemical Formula: $C_{12}H_{10}N_3O_2S_2$

Exact Mass: 291.0136

Target Compound Screening Report

Results Acquired by The University of Texas at Austin Mass Spectrometry Facility

**Data File**	MSF25-2087(AHL7004C)_hrESIposLC1.d	**Sample Name**	2087(AHL7004C)	**Comment**	2087(AHL7004C)
**Position**	P1-D4	**Instrument Name**	6530	**User Name**	
**Acq Method**	LC_C18_pos_jl.m	**Acquired Time**	12/4/2025 8:52:35 PM	**DA Method**	MSF.m

MS Zoomed Spectrum

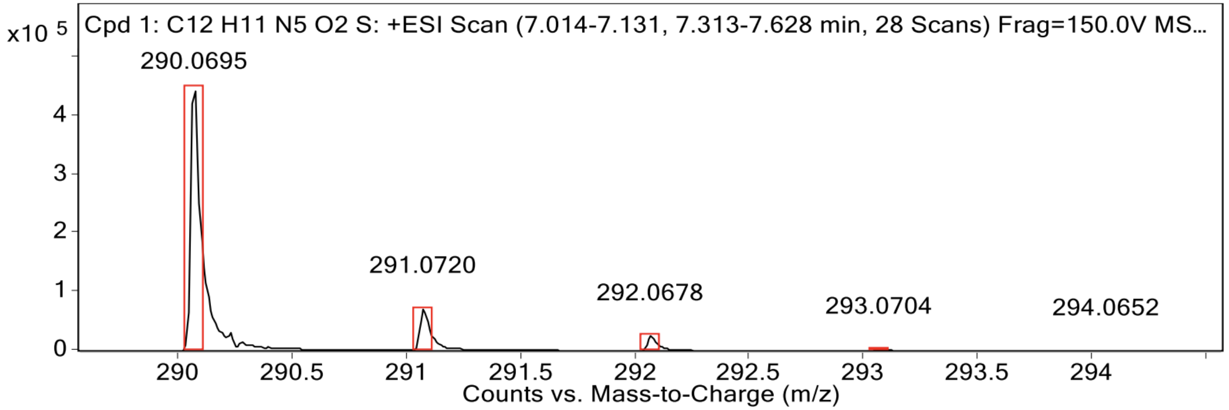

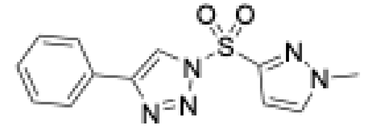

HSU064_AHL7004C

Chemical Formula: $C_{12}H_{11}N_5O_2S$

Exact Mass: 289.0633

MS Spectrum Peak List

Obs. m/z	Calc. m/z	Charge	Abundance	Formula	Ion Species	Tgt Mass Error (ppm)
290.0695	290.0706	1	449080	C12H11N5O2S	(M+H)+	3.72
291.0720	291.0731	1	72894	C12H11N5O2S	(M+H)+	3.55
292.0678	292.0687	1	26001	C12H11N5O2S	(M+H)+	3.26
293.0704	293.0703	1	3242	C12H11N5O2S	(M+H)+	-0.45
294.0652	294.0713	1	487	C12H11N5O2S	(M+H)+	20.67

--- End Of Report ---

Target Compound Screening Report

Results Acquired by The University of Texas at Austin Mass Spectrometry Facility

**Data File**	MSF25-2063(JWB101)_hrESIpos1.d	**Sample Name**	2063(JWB101)	**Comment**	2063(JWB101)
**Position**	P1-C1	**Instrument Name**	6530	**User Name**	
**Acq Method**	FIA_pos.m	**Acquired Time**	12/4/2025 10:25:37 AM	**DA Method**	MSF.m

MS Zoomed Spectrum

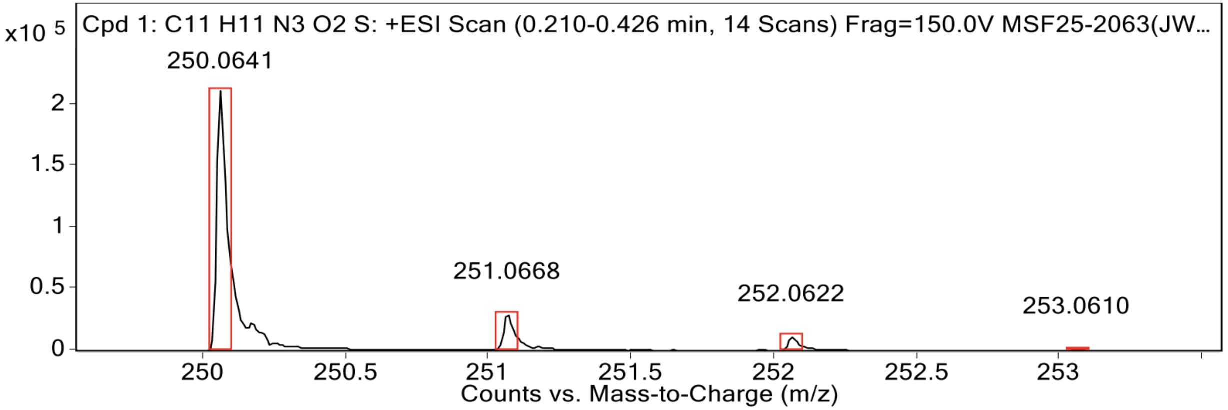

MS Spectrum Peak List

Obs. m/z	Calc. m/z	Charge	Abundance	Formula	Ion Species	Tgt Mass Error (ppm)
250.0641	250.0645	1	212953	C11H11N3O2S	(M+H)+	1.55
251.0668	251.0671	1	29722	C11H11N3O2S	(M+H)+	1.31
252.0622	252.0624	1	11501	C11H11N3O2S	(M+H)+	0.54
253.0610	253.0643	1	1470	C11H11N3O2S	(M+H)+	13.2
867.0737			212500			

--- End Of Report ---

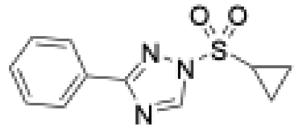

HSU071_JWB101

Chemical Formula: $C_{11}H_{11}N_3O_2S$

Exact Mass: 249.0572

Target Compound Screening Report

Results Acquired by The University of Texas at Austin Mass Spectrometry Facility

**Data File**	MSF25-2063(JWB106)_hrESIpos1.d	**Sample Name**	2063(JWB106)	**Comment**	2063(JWB106)
**Position**	P1-C2	**Instrument Name**	6530	**User Name**	
**Acq Method**	FIA_pos.m	**Acquired Time**	12/4/2025 10:27:38 AM	**DA Method**	MSF.m

MS Zoomed Spectrum

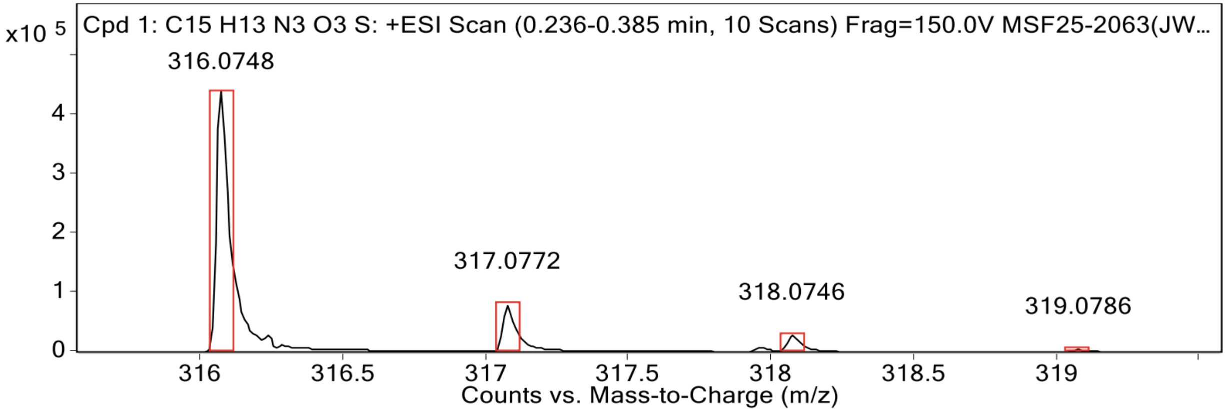

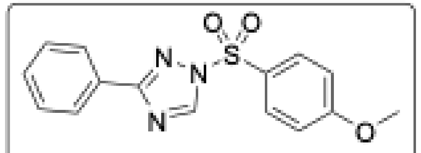

MS Spectrum Peak List

Obs. m/z	Calc. m/z	Charge	Abundance	Formula	Ion Species	Tgt Mass Error (ppm)
316.0748	316.0750	1	440410	C15H13N3O3S	(M+H)+	0.81
317.0772	317.0779	1	80937	C15H13N3O3S	(M+H)+	2.12
318.0746	318.0740	1	28563	C15H13N3O3S	(M+H)+	-1.94
319.0786	319.0756	1	4504	C15H13N3O3S	(M+H)+	-9.46
320.0772	320.0768	1	811	C15H13N3O3S	(M+H)+	-1.29

--- End Of Report ---

Target Compound Screening Report

Results Acquired by The University of Texas at Austin Mass Spectrometry Facility

**Data File**	MSF25-2063(JWB117)_hrESIpos1.d	**Sample Name**	2063(JWB117)	**Comment**	2063(JWB117)
**Position**	P1-C3	**Instrument Name**	6530	**User Name**	
**Acq Method**	FIA_pos.m	**Acquired Time**	12/4/2025 10:29:40 AM	**DA Method**	MSF.m

MS Zoomed Spectrum

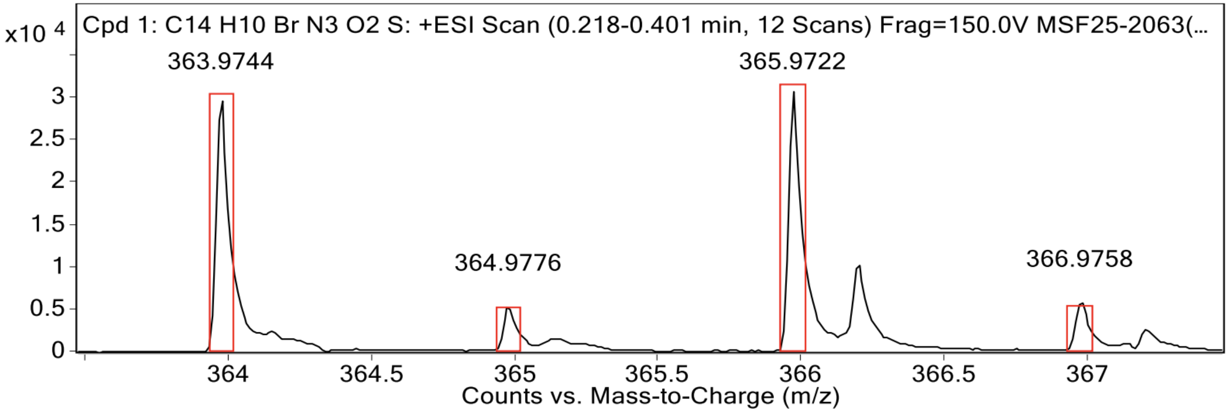

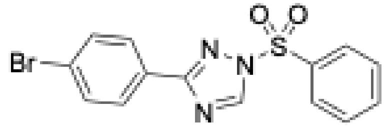

HSU073_JWB117
Chemical Formula: $C_{14}H_{10}BrN_3O_2S$
Exact Mass: 362.9677

MS Spectrum Peak List

Obs. m/z	Calc. m/z	Charge	Abundance	Formula	Ion Species	Tgt Mass Error (ppm)
130.1591			422236			
363.9744	363.9750	1	29876	C14H10BrN3O2S	(M+H)+	1.66
364.9776	364.9778	1	5489	C14H10BrN3O2S	(M+H)+	0.47
365.9722	365.9730	1	30849	C14H10BrN3O2S	(M+H)+	2.12
366.9758	366.9757	1	5953	C14H10BrN3O2S	(M+H)+	-0.37

--- End Of Report ---

Target Compound Screening Report

Results Acquired by The University of Texas at Austin Mass Spectrometry Facility

**Data File**	MSF25-2063(JWB134)_hrESIpos1.d	**Sample Name**	2063(JWB134)	**Comment**	2063(JWB134)
**Position**	P1-C6	**Instrument Name**	6530	**User Name**	
**Acq Method**	FIA_pos.m	**Acquired Time**	12/4/2025 10:35:47 AM	**DA Method**	MSF.m

MS Zoomed Spectrum

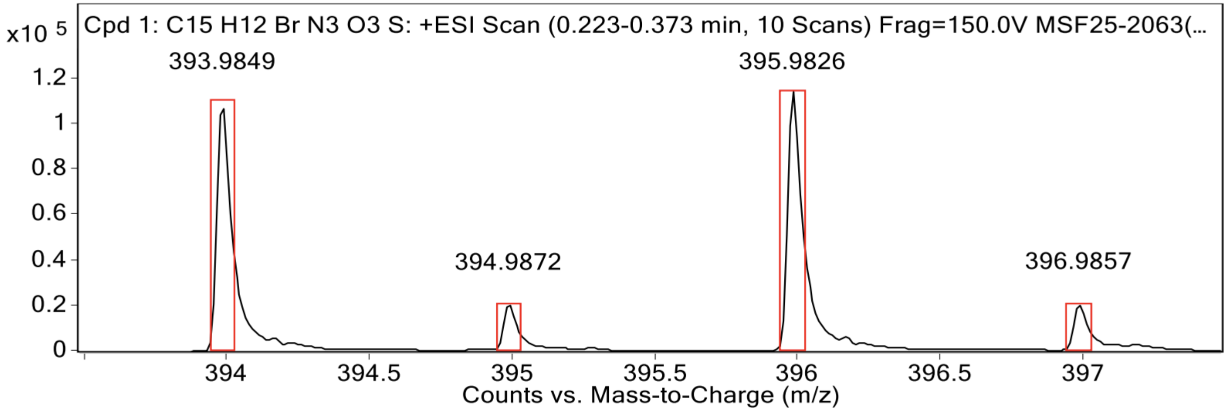

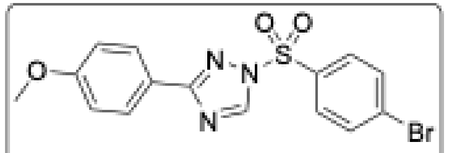

MS Spectrum Peak List

Obs. m/z	Calc. m/z	Charge	Abundance	Formula	Ion Species	Tgt Mass Error (ppm)
393.9849	393.9856	1	109046	C15H12BrN3O3S	(M+H)+	1.69
394.9872	394.9884	1	20545	C15H12BrN3O3S	(M+H)+	2.99
395.9826	395.9836	1	114568	C15H12BrN3O3S	(M+H)+	2.43
396.9857	396.9863	1	20906	C15H12BrN3O3S	(M+H)+	1.67
867.0743			319119			

--- End Of Report ---

Target Compound Screening Report

Results Acquired by The University of Texas at Austin Mass Spectrometry Facility

**Data File**	MSF25-2063(JWB124)_hrESIposLC1.d	**Sample Name**	2063(JWB124)	**Comment**	2063(JWB124)
**Position**	P1-C5	**Instrument Name**	6530	**User Name**	
**Acq Method**	LC_C18_pos_jl.m	**Acquired Time**	12/4/2025 12:04:43 PM	**DA Method**	MSF.m

MS Zoomed Spectrum

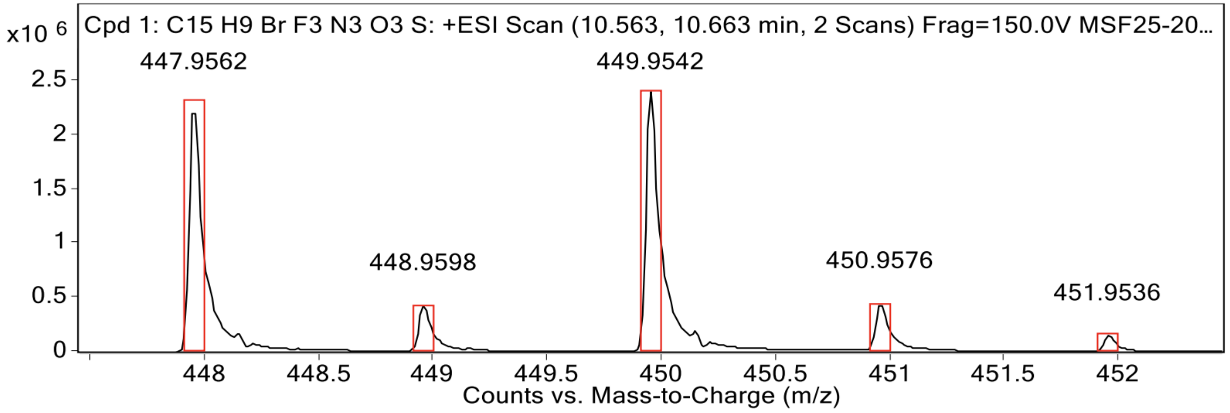

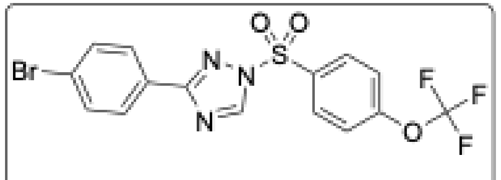

HSU076_JWB124
Chemical Formula: $C_{15}H_9BrF_3N_3O_3S$
Exact Mass: 446.9500

MS Spectrum Peak List

Obs. m/z	Calc. m/z	Charge	Abundance	Formula	Ion Species	Tgt Mass Error (ppm)
447.9562	447.9573	1	2289222	C15H9BrF3N3O3S	(M+H)+	2.33
448.9598	448.9601	1	430820	C15H9BrF3N3O3S	(M+H)+	0.73
449.9542	449.9553	1	2400483	C15H9BrF3N3O3S	(M+H)+	2.36
450.9576	450.9581	1	449016	C15H9BrF3N3O3S	(M+H)+	1.07
451.9536	451.9542	1	152213	C15H9BrF3N3O3S	(M+H)+	1.46

--- End Of Report ---

Target Compound Screening Report

Results Acquired by The University of Texas at Austin Mass Spectrometry Facility

**Data File**	MSF25-2063(JWB119)_hrESIposLC1.d	**Sample Name**	2063(JWB119)	**Comment**	2063(JWB119)
**Position**	P1-C4	**Instrument Name**	6530	**User Name**	
**Acq Method**	LC_C18_pos_jl.m	**Acquired Time**	12/4/2025 5:25:51 PM	**DA Method**	MSF.m

MS Zoomed Spectrum

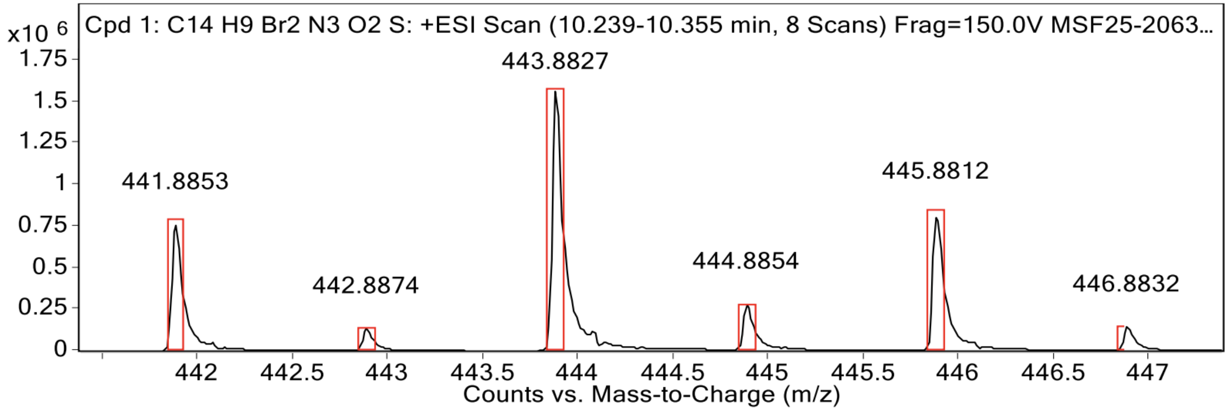

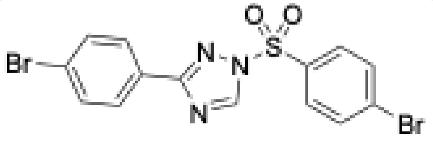

HSU077_JWB119

Chemical Formula: $C_{14}H_9Br_2N_3O_2S$
Exact Mass: 440.8782

MS Spectrum Peak List

Obs. m/z	Calc. m/z	Charge	Abundance	Formula	Ion Species	Tgt Mass Error (ppm)
441.8853	441.8855	1	763367	C14H9Br2N3O2S	(M+H)+	0.55
442.8874	442.8883	1	136331	C14H9Br2N3O2S	(M+H)+	2.04
443.8827	443.8835	1	1575727	C14H9Br2N3O2S	(M+H)+	1.84
444.8854	444.8862	1	284008	C14H9Br2N3O2S	(M+H)+	1.83
445.8812	445.8815	1	826850	C14H9Br2N3O2S	(M+H)+	0.64
446.8832	446.8841	1	146646	C14H9Br2N3O2S	(M+H)+	2.22

--- End Of Report ---

Target Compound Screening Report

Results Acquired by The University of Texas at Austin Mass Spectrometry Facility

**Data File**	MSF25-2063(JWB139)_hrESIpos1.d	**Sample Name**	2063(JWB139)	**Comment**	2063(JWB139)
**Position**	P1-C7	**Instrument Name**	6530	**User Name**	
**Acq Method**	FIA_pos.m	**Acquired Time**	12/4/2025 10:37:50 AM	**DA Method**	MSF.m

MS Zoomed Spectrum

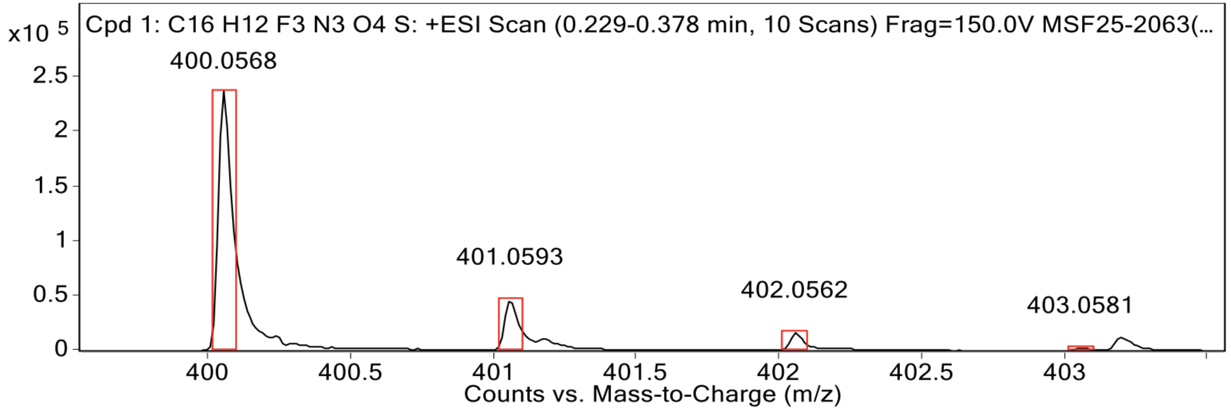

MS Spectrum Peak List

Obs. m/z	Calc. m/z	Charge	Abundance	Formula	Ion Species	Tgt Mass Error (ppm)
400.0568	400.0573	1	237845	C16H12F3N3O4S	(M+H)+	1.23
401.0593	401.0602	1	46701	C16H12F3N3O4S	(M+H)+	2.2
402.0562	402.0566	1	16432	C16H12F3N3O4S	(M+H)+	1.04
403.0581	403.0583	1	2353	C16H12F3N3O4S	(M+H)+	0.35

--- End Of Report ---

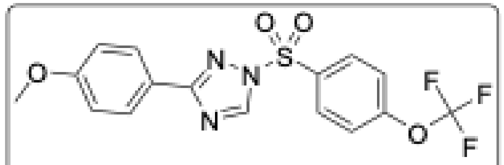

HSU078_JWB139
Chemical Formula: $C_{16}H_{12}F_3N_3O_4S$
Exact Mass: 399.0501

Target Screening Report

Results Acquired by The University of Texas at Austin Mass Spectrometry Facility

**Data File**	MSF25-2143(JWB147)_hrESIposLC1.d	**Sample Name**	2143(JWB147)	**Comment**	2143(JWB147)
**Position**	P1-C8	**Instrument Name**	Instrument 1		
**Acquisition Method**	LC_C18_CH3CN_JL.m	**Acquired Time**	12/19/2025 2:34:05 PM (UTC-06:00)	**DA Method**	MSF.m

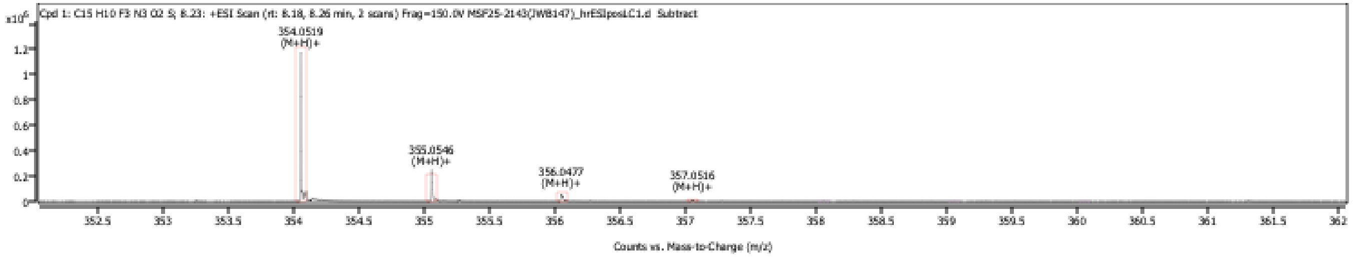

Spectrum Peaks

Obs. m/z	Calc. m/z	Charge	Abund	Formula	Ion Species	Tgt Mass Error (PPM)
354.0519	354.0519	1	1207995	C15H10F3N3O2S	(M+H)+	0.04
355.0546	355.0547	1	251557	C15H10F3N3O2S	(M+H)+	-0.12
356.0477	356.0506	1	55689	C15H10F3N3O2S	(M+H)+	-8.13
357.0516	357.0522	1	11552	C15H10F3N3O2S	(M+H)+	-1.49

MassHunter Qual 10.0
(End of Report)

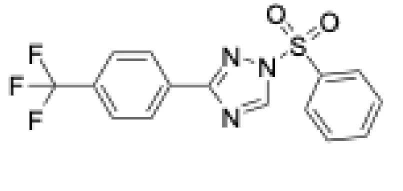

HSU079_JWB147
Chemical Formula: $C_{15}H_{10}F_3N_3O_2S$
Exact Mass: 353.0446

Target Compound Screening Report

Results Acquired by The University of Texas at Austin Mass Spectrometry Facility

**Data File**	MSF25-2063(JWB149)_hrESIposLC1.d	**Sample Name**	2063(JWB149)	**Comment**	2063(JWB149)
**Position**	P1-C8	**Instrument Name**	6530	**User Name**	
**Acq Method**	LC_C18_pos_jl.m	**Acquired Time**	12/4/2025 12:27:42 PM	**DA Method**	MSF.m

MS Zoomed Spectrum

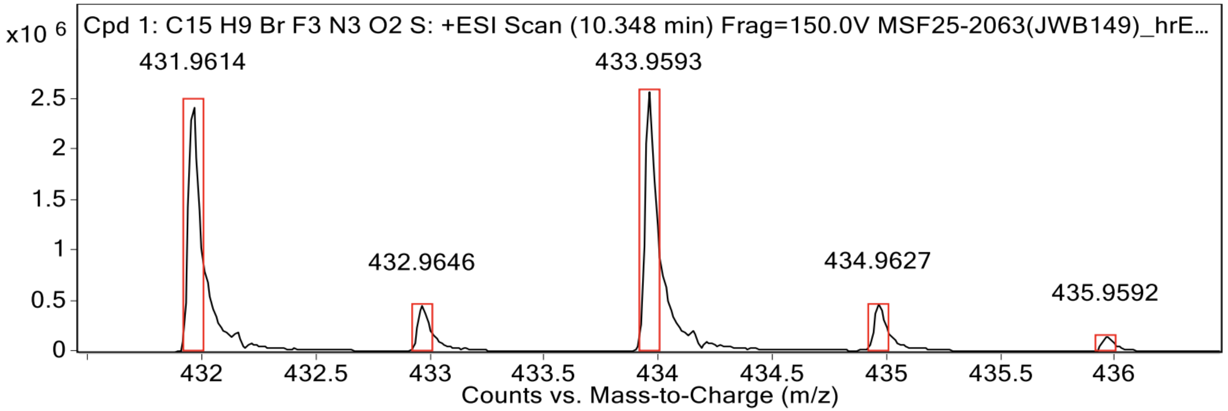

MS Spectrum Peak List

Obs. m/z	Calc. m/z	Charge	Abundance	Formula	Ion Species	Tgt Mass Error (ppm)
431.9614	431.9624	1	2447313	C15H9BrF3N3O2S	(M+H)+	2.28
432.9646	432.9652	1	463283	C15H9BrF3N3O2S	(M+H)+	1.42
433.9593	433.9604	1	2586893	C15H9BrF3N3O2S	(M+H)+	2.53
434.9627	434.9631	1	478074	C15H9BrF3N3O2S	(M+H)+	0.97
435.9592	435.9591	1	160057	C15H9BrF3N3O2S	(M+H)+	-0.19
436.9607	436.9607	1	20499	C15H9BrF3N3O2S	(M+H)+	-0.01

--- End Of Report ---

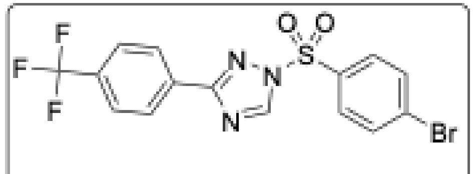

HSU080_JWB149

Chemical Formula: $C_{15}H_9BrF_3N_3O_2S$

Exact Mass: 430.9551

Target Compound Screening Report

Results Acquired by The University of Texas at Austin Mass Spectrometry Facility

**Data File**	MSF25-2087(AHL1101)_hrESIposLC1.d	**Sample Name**	2087(AHL1101)	**Comment**	2087(AHL1101)
**Position**	P1-C8	**Instrument Name**	6530	**User Name**	
**Acq Method**	LC_C18_pos_jl.m	**Acquired Time**	12/4/2025 6:57:49 PM	**DA Method**	MSF.m

MS Zoomed Spectrum

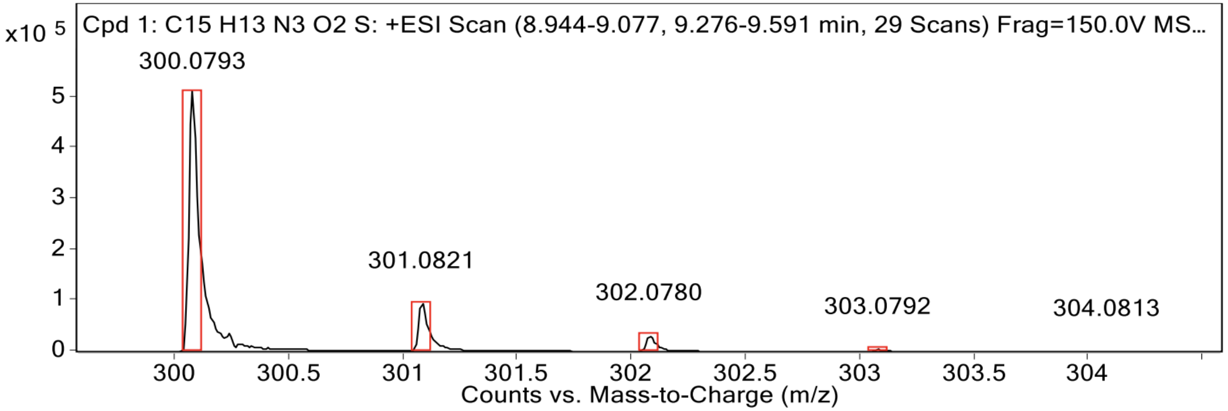

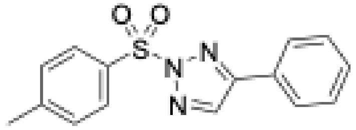

HSU086_AHL-1101

Chemical Formula: $C_{15}H_{13}N_3O_2S$

Exact Mass: 299.0728

MS Spectrum Peak List

Obs. m/z	Calc. m/z	Charge	Abundance	Formula	Ion Species	Tgt Mass Error (ppm)
300.0793	300.0801	1	512406	C15H13N3O2S	(M+H)+	2.81
301.0821	301.0830	1	94533	C15H13N3O2S	(M+H)+	2.8
302.0780	302.0789	1	31690	C15H13N3O2S	(M+H)+	2.79
303.0792	303.0804	1	4518	C15H13N3O2S	(M+H)+	4.28
304.0813	304.0817	1	560	C15H13N3O2S	(M+H)+	1.48

--- End Of Report ---

Target Compound Screening Report

Results Acquired by The University of Texas at Austin Mass Spectrometry Facility

**Data File**	MSF25-2087(AHL1118)_hrESIposLC1.d	**Sample Name**	2087(AHL1118)	**Comment**	2087(AHL1118)
**Position**	P1-C9	**Instrument Name**	6530	**User Name**	
**Acq Method**	LC_C18_pos_jl.m	**Acquired Time**	12/4/2025 7:20:48 PM	**DA Method**	MSF.m

MS Zoomed Spectrum

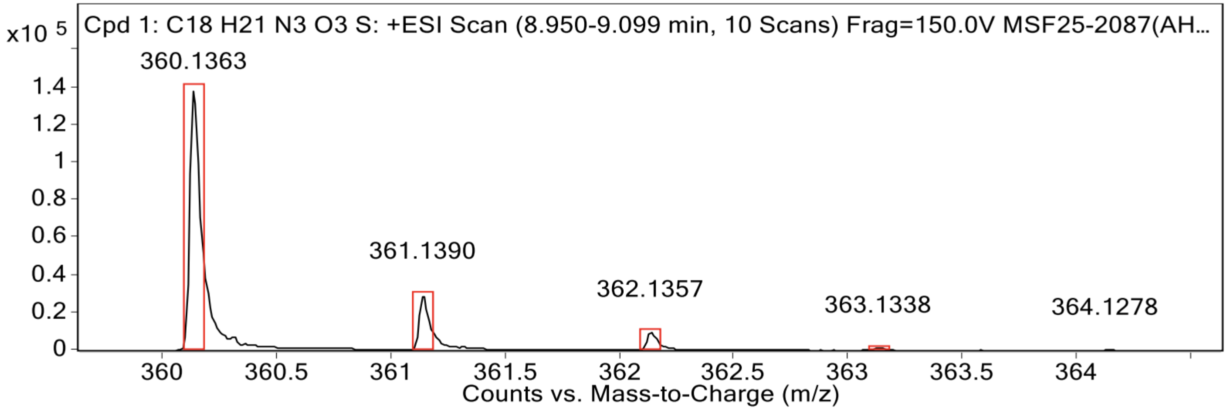

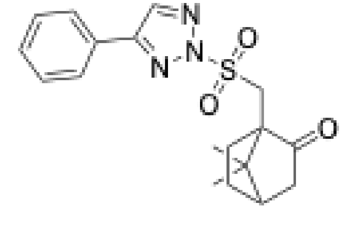

HSU087_AHL1118
Chemical Formula: $C_{18}H_{21}N_3O_3S$
Exact Mass: 359.1304

MS Spectrum Peak List

Obs. m/z	Calc. m/z	Charge	Abundance	Formula	Ion Species	Tgt Mass Error (ppm)
146.0701			4780333			
360.1363	360.1376	1	141517	C18H21N3O3S	(M+H)+	3.69
361.1390	361.1406	1	30287	C18H21N3O3S	(M+H)+	4.26
362.1357	362.1372	1	9962	C18H21N3O3S	(M+H)+	4.17
363.1338	363.1386	1	1560	C18H21N3O3S	(M+H)+	13.17

--- End Of Report ---

Target Compound Screening Report

Results Acquired by The University of Texas at Austin Mass Spectrometry Facility

**Data File**	MSF25-2087(AHL1119)_hrESIposLC1.d	**Sample Name**	2087(AHL1119)	**Comment**	2087(AHL1119)
**Position**	P1-D1	**Instrument Name**	6530	**User Name**	
**Acq Method**	LC_C18_pos_jl.m	**Acquired Time**	12/4/2025 7:43:46 PM	**DA Method**	MSF.m

MS Zoomed Spectrum

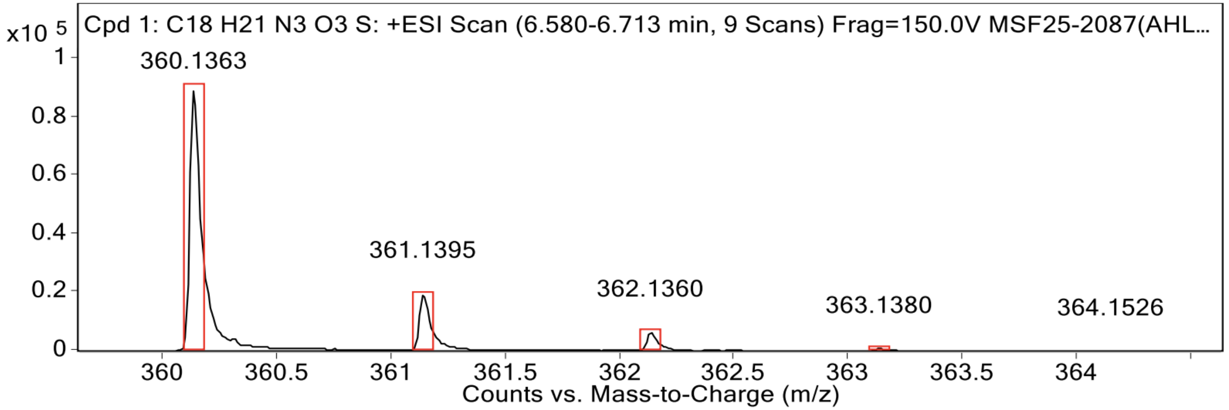

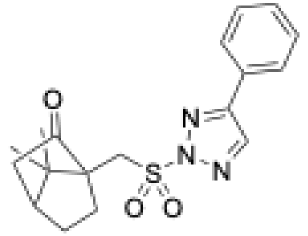

HSU088_AHL1119
Chemical Formula: $C_{18}H_{21}N_3O_3S$
Exact Mass: 359.1304

MS Spectrum Peak List

Obs. m/z	Calc. m/z	Charge	Abundance	Formula	Ion Species	Tgt Mass Error (ppm)
360.1363	360.1376	1	91013	C18H21N3O3S	(M+H)+	3.61
361.1395	361.1406	1	19815	C18H21N3O3S	(M+H)+	2.98
362.1360	362.1372	1	6552	C18H21N3O3S	(M+H)+	3.29
363.1380	363.1386	1	929	C18H21N3O3S	(M+H)+	1.57
364.1526	364.1400	1	101	C18H21N3O3S	(M+H)+	-34.75

--- End Of Report ---

Target Compound Screening Report

Results Acquired by The University of Texas at Austin Mass Spectrometry Facility

**Data File**	MSF25-2063(AHL1122)_hrESIpos1.d	**Sample Name**	2063(AHL1122)	**Comment**	2063(AHL1122)
**Position**	P1-E6	**Instrument Name**	6530	**User Name**	
**Acq Method**	FIA_pos.m	**Acquired Time**	12/4/2025 11:12:08 AM	**DA Method**	MSF.m

MS Zoomed Spectrum

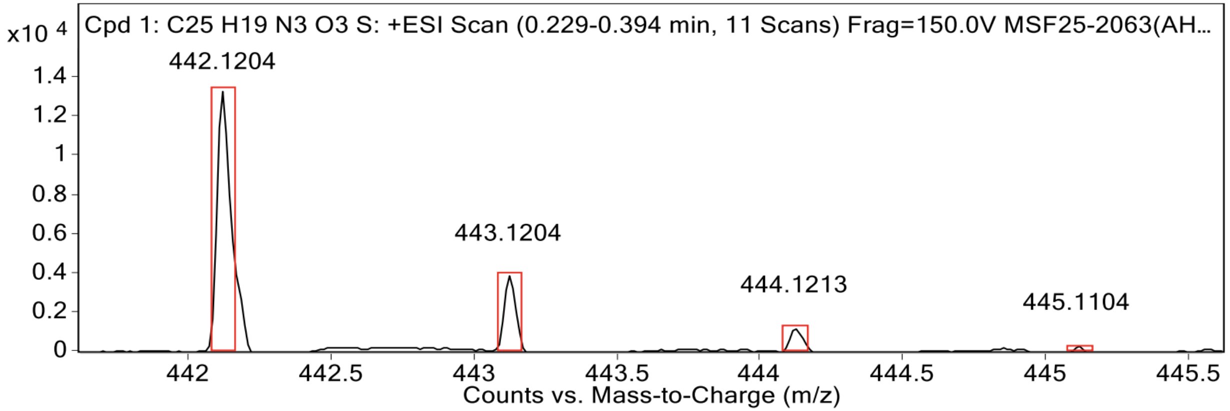

MS Spectrum Peak List

Obs. m/z	Calc. m/z	Charge	Abundance	Formula	Ion Species	Tgt Mass Error (ppm)
442.1204	442.1220	1	13323	C25H19N3O3S	(M+H)+	3.7
443.1204	443.1250	1	3886	C25H19N3O3S	(M+H)+	10.41
444.1213	444.1229	1	1240	C25H19N3O3S	(M+H)+	3.65
445.1104	445.1238	1	344	C25H19N3O3S	(M+H)+	30.11
905.2183			6399613			

--- End Of Report ---

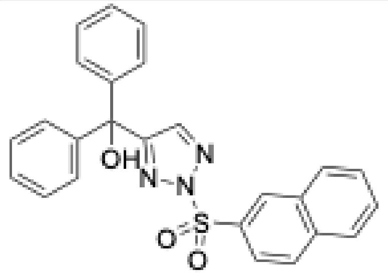

HSU089_AHL1122

Chemical Formula: $C_{25}H_{19}N_3O_3S$

Exact Mass: 441.1147

Target Compound Screening Report

Results Acquired by The University of Texas at Austin Mass Spectrometry Facility

**Data File**	MSF25-2063(AHL1125)_hrESIposLC1.d	**Sample Name**	Unavailable	**Comment**	Sample information is unavailable
**Position**	Unavailable	**Instrument Name**	Unavailable	**User Name**	Unavailable
**Acq Method**		**Acquired Time**	Unavailable	**DA Method**	MSF.m

MS Zoomed Spectrum

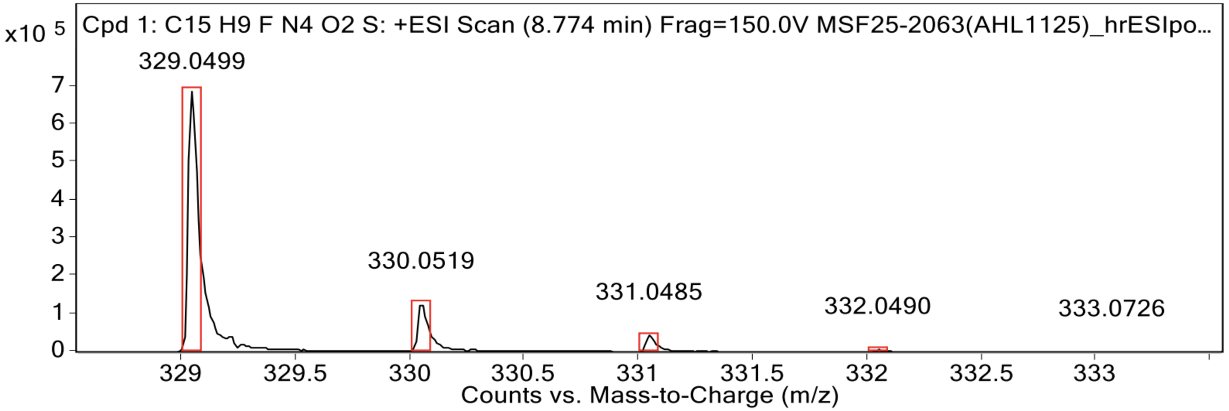

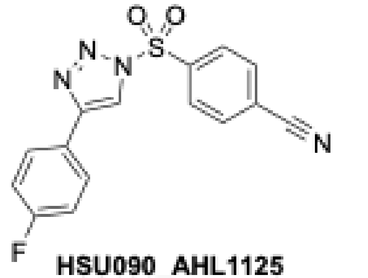

HSU090_AHL1125

Chemical Formula: $C_{15}H_9FN_4O_2S$
Exact Mass: 328.0430

MS Spectrum Peak List

Obs. m/z	Calc. m/z	Charge	Abundance	Formula	Ion Species	Tgt Mass Error (ppm)
329.0499	329.0503	1	695013	C15H9FN4O2S	(M+H)+	1.19
330.0519	330.0530	1	126472	C15H9FN4O2S	(M+H)+	3.49
331.0485	331.0490	1	44544	C15H9FN4O2S	(M+H)+	1.68
332.0490	332.0505	1	6993	C15H9FN4O2S	(M+H)+	4.49
333.0726	333.0517	1	643	C15H9FN4O2S	(M+H)+	-62.64
334.0612	334.0533	1	495	C15H9FN4O2S	(M+H)+	-23.8

--- End Of Report ---

Target Screening Report

Results Acquired by The University of Texas at Austin Mass Spectrometry Facility

**Data File**	MSF25-2129(AHL1126)_hrESIposLC1.d	**Sample Name**	2129(AHL1126)	**Comment**	2129(AHL1126)
**Position**	P1-C4	**Instrument Name**	Instrument 1		
**Acquisition Method**	LC_C18_CH3CN_JL.m	**Acquired Time**	12/18/2025 8:59:02 PM (UTC-06:00)	**DA Method**	MSF.m

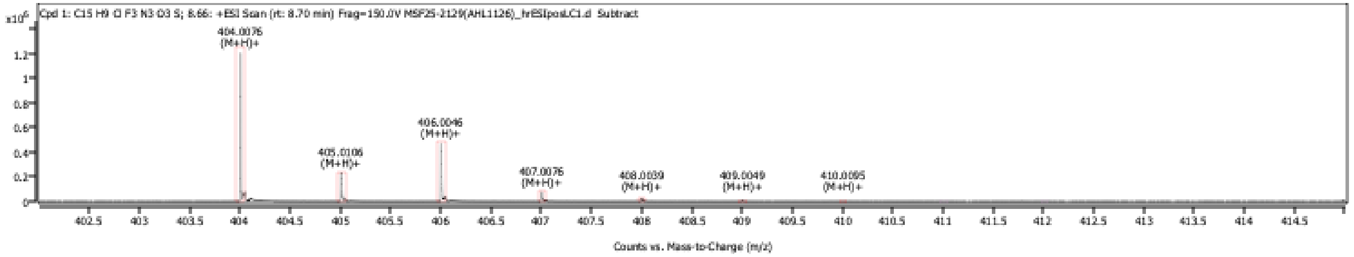

Spectrum Peaks

Obs. m/z	Calc. m/z	Charge	Abund	Formula	Ion Species	Tgt Mass Error (PPM)
404.0076	404.0078	1	1250670	C15H9ClF3N3O3S	(M+H)+	-0.56
405.0106	405.0106	1	240559	C15H9ClF3N3O3S	(M+H)+	-0.12
406.0046	406.0052	1	477045	C15H9ClF3N3O3S	(M+H)+	-1.49
407.0076	407.0078	1	78114	C15H9ClF3N3O3S	(M+H)+	-0.46
408.0039	408.0041	1	19458	C15H9ClF3N3O3S	(M+H)+	-0.30
409.0049	409.0056	1	4511	C15H9ClF3N3O3S	(M+H)+	-1.71
410.0095	410.0067	1	517	C15H9ClF3N3O3S	(M+H)+	6.74

MassHunter Qual 10.0
(End of Report)

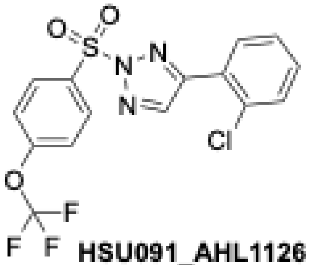

HSU091_AHL1126
Chemical Formula: $C_{15}H_9ClF_3N_3O_3S$
Exact Mass: 403.0005

Target Screening Report

Results Acquired by The University of Texas at Austin Mass Spectrometry Facility

**Data File**	MSF25-2129(AHL2065)_hrESIposLC1.d	**Sample Name**	2129(AHL2065)	**Comment**	2129(AHL2065)
**Position**	P1-C5	**Instrument Name**	Instrument 1		
**Acquisition Method**	LC_C18_CH3CN_JL.m	**Acquired Time**	12/18/2025 9:21:00 PM (UTC-06:00)	**DA Method**	MSF.m

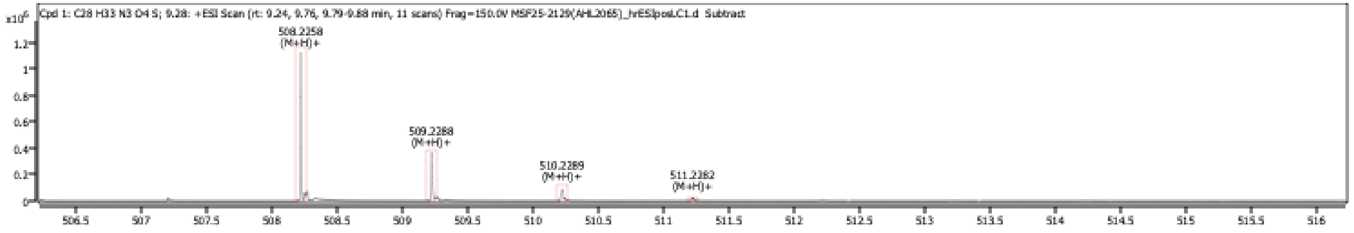

Spectrum Peaks

Obs. m/z	Calc. m/z	Charge	Abund	Formula	Ion Species	Tgt Mass Error (PPM)
508.2258	508.2265	1	1165402	C28H33N3O4S	(M+H)+	-1.25
509.2288	509.2295	1	369669	C28H33N3O4S	(M+H)+	-1.55
510.2289	510.2280	1	88122	C28H33N3O4S	(M+H)+	1.77
511.2282	511.2289	1	20306	C28H33N3O4S	(M+H)+	-1.32
336.2066			2485220			

MassHunter Qual 10.0
(End of Report)

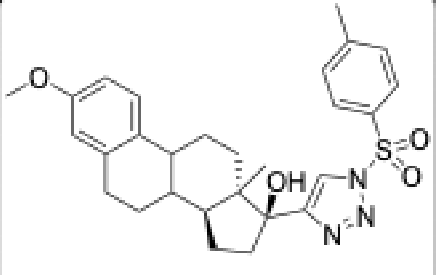

HSU092_AHL2065
Chemical Formula: $C_{28}H_{33}N_3O_4S$
Exact Mass: 507.2192

Target Compound Screening Report

Results Acquired by The University of Texas at Austin Mass Spectrometry Facility

**Data File**	MSF25-2063(JWB230)_hrESIpos1.d	**Sample Name**	2063(JWB230)	**Comment**	2063(JWB230)
**Position**	P1-D4	**Instrument Name**	6530	**User Name**	
**Acq Method**	FIA_pos.m	**Acquired Time**	12/4/2025 10:49:55 AM	**DA Method**	MSF.m

MS Zoomed Spectrum

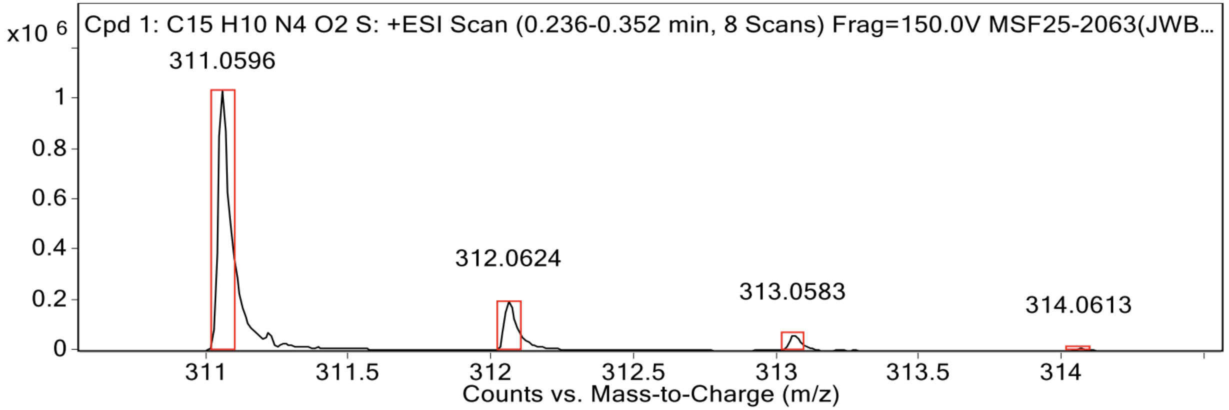

MS Spectrum Peak List

Obs. m/z	Calc. m/z	Charge	Abundance	Formula	Ion Species	Tgt Mass Error (ppm)
311.0596	311.0597	1	1034641	C15H10N4O2S	(M+H)+	0.28
312.0624	312.0624	1	196895	C15H10N4O2S	(M+H)+	0.21
313.0583	313.0585	1	64033	C15H10N4O2S	(M+H)+	0.69
314.0613	314.0599	1	10785	C15H10N4O2S	(M+H)+	-4.5
333.0414			1629377			

--- End Of Report ---

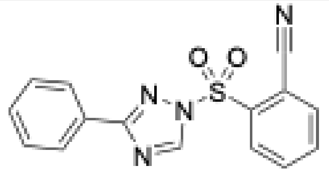

HSU093_JWB230
Chemical Formula: $C_{15}H_{10}N_4O_2S$
Exact Mass: 310.0524

Target Compound Screening Report

Results Acquired by The University of Texas at Austin Mass Spectrometry Facility

**Data File**	MSF25-2063(JWB234)_hrESIposLC1.d	**Sample Name**	2063(JWB234)	**Comment**	2063(JWB234)
**Position**	P1-D5	**Instrument Name**	6530	**User Name**	
**Acq Method**	LC_C18_pos_jl.m	**Acquired Time**	12/4/2025 1:13:39 PM	**DA Method**	MSF.m

MS Zoomed Spectrum

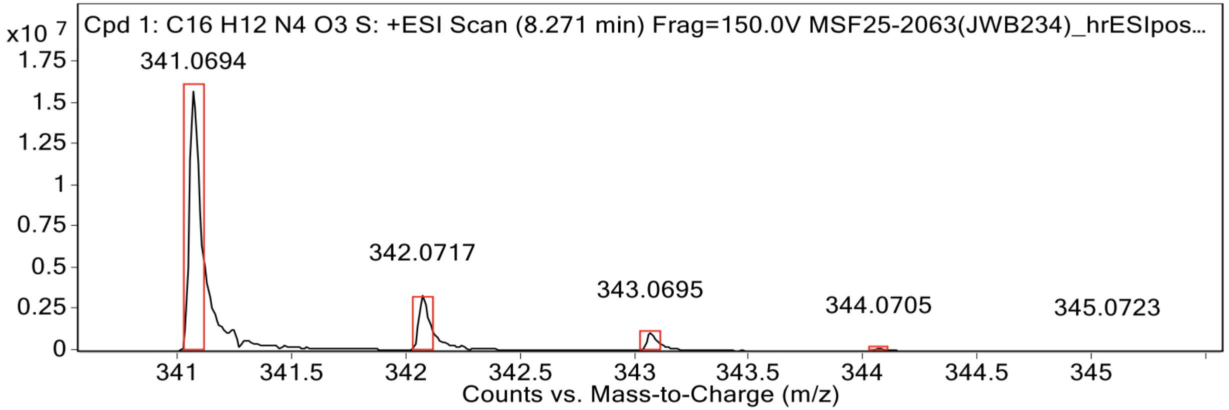

MS Spectrum Peak List

Obs. m/z	Calc. m/z	Charge	Abundance	Formula	Ion Species	Tgt Mass Error (ppm)
341.0694	341.0703	1	16007025	C16H12N4O3S	(M+H)+	2.53
342.0717	342.0730	1	3361506	C16H12N4O3S	(M+H)+	4.05
343.0695	343.0694	1	1111244	C16H12N4O3S	(M+H)+	-0.08
344.0705	344.0709	1	165984	C16H12N4O3S	(M+H)+	0.99
345.0723	345.0721	1	20594	C16H12N4O3S	(M+H)+	-0.61

--- End Of Report ---

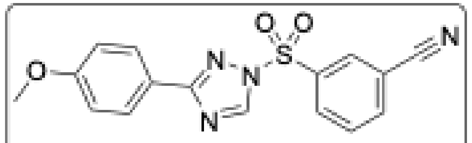

HSU094_JWB234

Chemical Formula: $C_{16}H_{12}N_4O_3S$

Exact Mass: 340.0630
