# Supplementary material for: Protein tyrosine phosphatase inactivation by electrophilic tyrosine modification
Source: Chem Sci. 2026 Jan 19;17(10):5163–71. doi: 10.1039/d5sc07398g (PMC12814959; doi:10.1039/d5sc07398g)
Supplement: SC-017-D5SC07398G-s003 [file SC-017-D5SC07398G-s003.pdf]

This journal is © The Royal Society of Chemistry 2026

Table S1. % PTP activity after SuTEx ligand treatment. Compounds are ordered by activity against SHP2.

Table S2. PTP peptides detected after DML189 treatment and tryptic digest (reducing)

Table S3. Fitness list for DML189 docking with SHP2 at Y279

Table S4. SHP peptides detected after DML189 treatment, tryptic digest, and EThcD (non-reducing)
